# Association between antipsychotic medication and clinically relevant weight change: meta-analysis

**DOI:** 10.1192/bjo.2022.619

**Published:** 2023-01-18

**Authors:** Bea Campforts, Marjan Drukker, Joost Crins, Therese van Amelsvoort, Maarten Bak

**Affiliations:** Department of Psychiatry and Neuropsychology, School for Mental Health and Neuroscience, Maastricht University, Maastricht, The Netherlands; Faculty of Health Medicine and Life Science, Maastricht University, Maastricht, The Netherlands

**Keywords:** Antipychotics, weight gain, weight loss, clinically relevant, antipsychotic-naive

## Abstract

**Background:**

Previous meta-analyses have shown that almost all antipsychotics are associated with weight gain. However, mean weight gain is not informative about clinically relevant weight gain or weight loss.

**Aims:**

To provide further insight into the more severe body weight changes associated with antipsychotic use, we assessed the proportion of patients with clinically relevant weight gain (CRWG) and clinically relevant weight loss (CRWL), defined as ≥7% weight gain and ≥7% weight loss.

**Method:**

We searched PubMed, Embase and PsycInfo for randomised controlled trials of antipsychotics that reported CRWG and CRWL in study populations aged 15 years or older. We conducted meta-analyses stratified by antipsychotic and study duration using a random-effects model. We performed meta-regression analyses to assess antipsychotic-naive status and psychiatric diagnosis as modifiers for CRWG. PROSPERO: CRD42020204734.

**Results:**

We included 202 articles (201 studies). Almost all included antipsychotics were associated with CRWG. For CRWL, available data were too limited to draw firm conclusions. For some antipsychotics, CRWG was more pronounced in individuals who were antipsychotic-naive than in individuals switching to another antipsychotic. Moreover, a longer duration of antipsychotic use was associated with more CRWG, but not CRWL. For some antipsychotics, CRWG was higher in people diagnosed with schizophrenia, but this was inconsistent.

**Conclusions:**

Switching antipsychotic medication is associated with both weight gain and weight loss, but the level of CRWG is higher than CRWL in antipsychotic-switch studies. CRWG was more pronounced in antipsychotic-naive patients, highlighting their vulnerability to weight gain. The impact of diagnosis on CRWG remains inconclusive.

The life expectancy of people with severe mental disorders is 20 years lower and mortality is higher than in the general population, partly explained by the metabolic side-effects of antipsychotic medication.^1–3^ Side-effects and their comorbidities include weight gain, obesity, hyperglycaemia, dyslipidaemia, metabolic syndrome, cardiovascular disease and stroke.^4,5^ These side-effects increase healthcare consumption, reduce the quality of life and lead to lower medication adherence, with an increased risk of acute psychiatric illness.^2,6–9^ The underlying mechanisms of antipsychotic-induced weight gain are still not fully understood. A complex interplay of neurotransmitters, hormones, neuropeptides and genetic and epigenetic factors contribute to it.^10–16^ However, it remains impossible to predict antipsychotic-induced weight gain in individual patients, as no definite predictive factors are known.^13,15,16^

A recent meta-analysis showed that antipsychotics were associated with weight gain over time.^17^ Other reviews and meta-analyses showed similar results.^16,18–25^ In general, olanzapine and clozapine are associated with the most severe weight gain.^16–25^ Ziprasidone,^17,18,20–25^ aripiprazole^17,20,23,25^ and lurasidone^21,24,25^ show a more benign profile. However, looking at weight as a continuous variable has limitations, as some patients gain body weight whereas others lose body weight. The average outcome of weight change depends on a balance between weight gain and weight loss. This average may also be determined by outliers in a small proportion of patients, leading to an overestimation of the mean weight gain or loss. Therefore, assessing the proportion of patients with clinically relevant weight gain (CRWG) and clinically relevant weight loss (CRWL), defined as ≥7% weight gain and ≥7% weight loss,^26,27^ may provide further insight into the more severe body weight changes associated with antipsychotic use.

To date, only a handful of systematic reviews and meta-analyses have addressed CRWG and even fewer CRWL. Over the short term (≤13 weeks), evidence indicated that most antipsychotics were associated with a significantly higher risk of CRWG compared with placebo; the exceptions were aripiprazole,^28^ cariprazine,^24^ lurasidone,^24,28,29^ quetiapine XR^28^ and ziprasidone.^24^ In these meta-analyses, olanzapine was associated with the highest risk of CRWG.^24,28^ In a recent comparison, in mainly short-term studies, olanzapine was associated with a higher risk of CRWG than risperidone and paliperidone. Risperidone, in turn, had a higher chance of CRWG than paliperidone.^30^ In a meta-analysis of first-episode psychosis studies, all antipsychotics except ziprasidone showed a significantly higher risk of CRWG compared with placebo.^31^

Evidence for the long term is particularly scarce. A comprehensive database analysis showed that in long-term studies (≥52 weeks), a higher proportion of patients taking olanzapine or risperidone had CRWG than patients taking ziprasidone or a placebo.^22^ For ziprasidone and placebo, the proportion of patients with CRWL was higher than that with CRWG, suggesting that ziprasidone could be weight-neutral.^22^ In a recent review, most antipsychotics were associated with a significantly higher risk of CRWG compared with placebo; the exception was aripiprazole.^32^

A previous meta-analysis, stratified by study duration, showed that the duration of antipsychotic use was associated with a clinically significant proportion of people experiencing CRWG on almost all antipsychotics.^20^ It suggests a time effect, with a longer duration of antipsychotic use associated with weight gain.^17,20^ Also, a statistically significant proportion experienced CRWL after starting an antipsychotic, but no duration–response pattern was found.^20^ To date, no other systematic review or meta-analysis has assessed CRWG or CRWL in longer-term (≥13 weeks) studies.

Recent systematic reviews and network meta-analyses either focused mainly on short-term weight gain^24,28,29^ or did not distinguish between short-term and long-term studies.^24,25,30–32^ They also included mainly studies involving people with schizophrenia, and they did not clearly distinguish between antipsychotic-naive patients (i.e. those with no history of antipsychotic use) and antipsychotic-switching patients (i.e. those switching from one antipsychotic to another).^24,25^ This distinction is relevant because antipsychotic-naive patients are more likely to develop antipsychotic-induced weight gain, and there is no effect of prior antipsychotic use on body weight, as previous reviews and meta-analyses^15,17,20,33^ have shown.

## Aims

With this meta-analysis, we aimed to update the Bak et al meta-analysis from 2014.^20^ This update was limited to data on CRWG and CRWL, and included the new antipsychotics introduced from 2012 onwards. As in the earlier meta-analysis, we stratified analyses by study duration and distinguished four time frames by dividing the number of days in the study by 7: short-term (<6 weeks), medium short-term (6–16 weeks, where the cutoff result is 16 inclusive), medium-term (16–38 weeks, where the starting result is measured from 16.1) and long-term studies (>38 weeks). We distinguished between antipsychotic-naive and antipsychotic-switching patients and reported CRWG and CRWL regardless of diagnosis.

Second, to enable future prediction, we investigated whether several factors were associated with weight gain. We expected that the duration of antipsychotic use would be associated with a higher proportion of individuals with CRWG and CRWL. Furthermore, we expected CRWG to be more pronounced in the antipsychotic-naive group than in the antipsychotic-switching group. Finally, we investigated whether a diagnosis of schizophrenia was associated with more CRWG compared with other psychiatric diagnoses.

## Method

### Protocol

The meta-analysis was performed and reported according to the guidelines of the Preferred Reporting Items for Systematic Reviews and Meta-Analyses (PRISMA) statement.^34^ The review protocol was registered with the International Prospective Register of Systematic Reviews (PROSPERO: CRD42020204734).

### Search strategy

We performed a literature search assessing CRWG and CRWL associated with the use of antipsychotic medication in randomised controlled trials (RCTs).

We conducted an electronic database search in PubMed, Embase and PsycInfo for articles that reported CRWG or CRWL in patients taking antipsychotic medication. The search terms used for PubMed were ((change OR gain OR clinically relevant OR body) AND weight)), combined with the various antipsychotic names. The search terms were similar for each database; the complete search strategy is provided in Supplementary Appendix 2, available at https://doi.org/10.1192/bjo.2022.619. We searched databases from their respective inception dates until 21 July 2021 and used no language filters. Hand-searching reference lists of previously published reviews and meta-analyses supplemented the database search.

### Study selection

We designed the search to identify RCTs with patients receiving an antipsychotic medication in placebo-controlled or active-controlled trial conditions. The outcome measures of interest were CRWG and CRWL, defined as ≥7% weight gain or ≥7% weight loss. We defined the following inclusion and exclusion criteria.

The inclusion criteria were:

- study populations aged 15 years or older taking an antipsychotic drug, all diagnoses included, except eating disorders
- a randomised placebo-controlled or active-controlled design
- available CRWG or CRWL data per antipsychotic (gain and/or loss).

The exclusion criteria were:

- studies that did not apply an intention-to-treat (ITT) analysis
- a study population with at least 1 participant younger than 15 years
- a study population diagnosed with eating disorders (e.g. anorexia nervosa or bulimia nervosa)
- a study population receiving adjunctive therapy (medication, behavioural therapy, lifestyle interventions) that might influence body weight during the study
- a study population using antipsychotic medication to treat nausea or vomiting in, for example, people with cancer or pregnant women
- studies with a duration of less than 1 week, for example studies assessing brain changes after antipsychotic intake, rapid tranquillisation studies, acute antipsychotic intervention studies and treatment of delirium: these studies are too short in duration and interventions will not substantially affect body weight in a week
- systematic reviews; meta-analyses; pooled data-sets; *post hoc* and subgroup analyses; case reports; expert opinions; narrative reviews; poster presentations
- non-human studies
- studies not published in English: although we are multilingual, we chose English as it is the most accessible to all researchers and the most transparent and traceable.

At least two reviewers (B.C., J.C. and M.B.) per article independently performed all the steps of the selection process. First, we controlled for double entries and checked titles and abstracts for relevance and eligibility. Then, we screened potentially relevant full-text articles for the inclusion and exclusion criteria. In case of disagreement between the reviewers, they discussed the articles until they reached a consensus.

We extracted data only from studies that performed an ITT analysis because drop-out due to weight problems could otherwise bias our results. We excluded extension studies if they had different baseline data than the original study or reported a subgroup analysis. Also, we included only studies that reported weight change per individual antipsychotic or group of antipsychotics (i.e. first- or second-generation antipsychotics).

### Data extraction

Two reviewers independently extracted the data (B.C., J.C.), with these two reviewers checking each other's data extraction. In case of disagreement, a third reviewer (M.B.) made the final decision.

Characteristics of included studies to be extracted were: author, year of publication, study design, number of participants, the proportion of male/female participants, mean age (with standard deviation), diagnosis, antipsychotic treatment, antipsychotic-naive/switching population, study duration (in weeks), baseline body mass index (BMI), the proportion of participants with CRWG and proportion of participants with CRWL. We extracted data for every randomised study group separately (either using antipsychotic or placebo).

### Outcome measures

The primary outcome measures were: the proportion of patients with (a) CRWG or (b) CRWL after starting or switching an antipsychotic.

We defined four categories of antipsychotic exposure: short-term (<6 weeks), medium short-term (6–16 weeks), medium-term (16–38 weeks) and long-term (>38 weeks).

### Risk of bias assessment

Risk of bias assessment for the primary outcomes was done by two independent reviewers (B.C., M.B.) using the Cochrane risk of bias tool.^35^ As we had already started piloting our review before a newer version of the tool became available, we decided to continue using the original instrument for our assessment. We assessed the following domains: (a) random sequence generation, (b) allocation concealment, (c) masking (‘blinding’), (d) incomplete outcome data and (e) selective outcome reporting; and classified individual risk of bias items as low, high or unclear risk (Supplementary Appendix 11). Any disagreements among reviewers were resolved by consulting a third reviewer (M.D.). The Grading of Recommendations Assessment, Development and Evaluation (GRADE) system^36^ was used to evaluate the certainty of evidence as high, moderate, low or very low (Supplementary Appendix 11).

### Statistical analysis

We performed all statistical analyses using Stata for Mac version 13.1.^37^ We performed meta-analyses using a random-effects model since we assumed included studies to be heterogeneous. Forest plots, including pooled estimates with their corresponding 95% confidence intervals (95% CI), were generated for all antipsychotics stratified by study duration using the *metaprop* command via the Freeman–Tukey double arcsine transformation method.^38^ The analyses aim to test whether the proportions of patients with CRWG and CRWL are significantly different from zero. Meta-analyses were performed only if data from at least two studies per antipsychotic could be included. Summary estimates were calculated under the DerSimonian and Laird method. We calculated the 95% CI of the pooled estimates using the Wald method. We further explored heterogeneity using the *I*-squared test, which estimates the proportion of variability in the summary estimates resulting from heterogeneity among the true effects. *I*-squared values of more than 50% were considered to indicate heterogeneity.

In the subset of antipsychotics with sufficient data, we performed meta-regression analyses to assess whether the duration of antipsychotic use was a modifier for CRWG or CRWL and whether there was a difference in weight gain between antipsychotic-naive and switched patients. To this end, we transformed proportional data using a natural logarithm. Furthermore, we performed meta-regression analyses to investigate whether other psychiatric diagnoses were associated with less CRWG compared with schizophrenia. Schizophrenia was used as the reference category in these analyses for reasons of power, since people with schizophrenia are by far the most included in RCTs assessing antipsychotics and body-weight change. Analyses were stratified by antipsychotic and duration of exposure.

Since the inclusion of various study populations could have contributed to heterogeneity, we performed sensitivity analyses by excluding studies in antipsychotic-naive populations and studies in populations aged over 65 years. We also performed a sensitivity analysis by excluding studies with a high risk of bias for at least one domain according to the Cochrane risk of bias tool.

Finally, we assessed publication bias stratified by antipsychotic and duration. We obtained funnel plots and performed Egger tests. We performed trim-and-fill analyses to estimate the effects of publication bias.

## Results

### Search results

The database search yielded a total of 2363 articles: 918 in PubMed, 1393 in Embase and 52 in PsycInfo. Manual searches of reference lists of previously published reviews and meta-analyses, pooled analyses, *post hoc* analyses and conference abstracts yielded another 47 potentially eligible articles.

After removing duplicates, 1822 articles remained. Checking titles and abstracts led to the exclusion of a further 1265 articles. The PRISMA flow diagram presents an overview of the selection process (Supplementary Appendix 1). Pooled data-sets and *post hoc* analyses were included only when the original published articles did not report CRWG or CRWL.^39–43^ RCTs that allowed co-medication were included only if a stable dose was maintained throughout the study period.

Full-text screening resulted in the exclusion of a further 355 articles (Supplementary Appendix 4). Two articles reported data on one study, with one paper reporting data on CRWG and the other on CRWL in the same study population.^44,45^ We included 202 articles (201 studies) with a total of 80 372 participants in the meta-analysis (Supplementary Appendix 3).

### CRWG

Of the included studies, 200 studies had usable data on CRWG (557 records in the meta-analysis data). Two studies reported on CRWL only.^45,46^ Most studies reported on CRWG in antipsychotic-switch patients (193 studies); only 7 studies investigated CRWG in antipsychotic-naive patients. The proportion of participants showing CRWG was significant for all antipsychotics and for placebo and varied between 1.7% (blonanserin <6 weeks) 1.9% (brexpiprazole 6–16 weeks) and 2.0% (placebo <6 week) on the one hand and 47% (clozapine 16–38 weeks), 49% (first-generation antipsychotics >38 weeks) and 76% (clozapine >38 weeks) on the other. Full details and forest plots can be found in Supplementary Appendix 5. Results for antipsychotics for which data for only one exposure period were available are presented in Supplementary Appendix 7. The *I*-squared for the included studies ranged from 0.0 to 94.16%, indicating no heterogeneity to strong heterogeneity (Supplementary Appendix 5.1).

### CRWG and duration of antipsychotic use

**Fig. 1:**
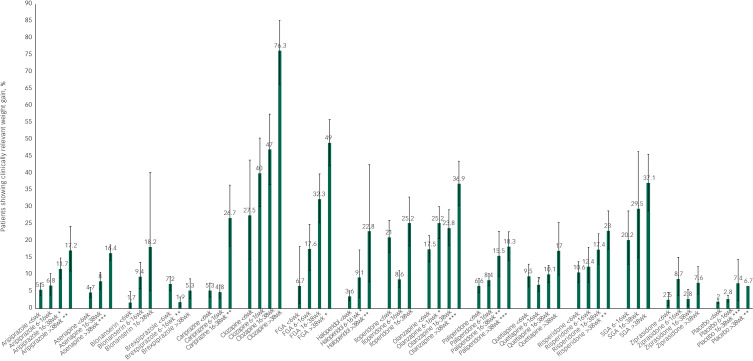
Clinically relevant weight gain per antipsychotic per time period. Only antipsychotics with ≥3 data points are displayed. Significant differences from the reference period <6 weeks are marked: *P < 0.05, **P < 0.01, ***P < 0.001. wk, weeks; FGA, first-generation antipsychotics; SGA, second-generation antipsychotics.

CRWG by treatment duration is shown in Fig. 1. Only antipsychotics with data for three or more exposure periods are shown. Visual inspection shows that for most antipsychotics, the proportions of patients with CRWG continue to increase with longer exposure; the exceptions are ziprasidone and brexpiprazole.

Meta-regression analyses showed that the proportions of patients with CRWG were significantly higher for aripiprazole (16–38 weeks, >38 weeks), asenapine (>38 weeks), cariprazine (16–38 weeks), first-generation antipsychotics (>38 weeks), haloperidol (6–16 weeks, >38 weeks), olanzapine (6–16 weeks, >38 weeks), paliperidone (16–38 weeks, >38 weeks) and risperidone (>38 weeks) compared with the exposure period <6 weeks. For brexpiprazole, CRWG was significantly lower in period 2 (6–16 weeks) than in the exposure period <6 weeks. Full results are presented in Table 1.

**Table 1:** Meta-regression analysis: effect of treatment duration on clinically relevant weight gain

Antipsychotic	Period, weeks	*B*	95% CI	*P*
Amisulpride	6–16^a^	0	0	0
	16–38	0.47	−0.29 to 1.23	0.145
Aripiprazole	<6^a^	0	0	0
	6–16	0.09	−0.44 to 0.62	0.725
	16–38	0.69	0.12 to 1.26	**0**.**019**
	>38	1.14	0.47 to 1.80	**0**.**001**
Asenapine	<6	0	0	0
	16–38	0.50	−0.02 to 1.01	0.057
	>38	1.67	0.98 to 2.36	**<0**.**001**
Blonanserin	<6	0	0	0
	6–16	1.03	−2.66 to 4.71	0.353
	16–38	1.91	−2.73 to 6.54	0.219
Brexpiprazole	<6	0	0	0
	6–16	−1.47	−2.41 to −0.53	**0**.**006**
	>38	−0.40	−1.38 to 0.57	0.384
Cariprazine	<6	0	0	0
	6–16	−0.11	−0.86 to 0.63	0.751
	16–38	1.82	0.63 to 3.01	**0**.**006**
Clozapine	<6	0	0	0
	6–16	0.33	−4.61 to 5.27	0.843
	16–38	0.92	−4.04 to 5.87	0.598
	>38	2.15	−2.81 to 7.10	0.262
First-generation antipsychotics	<6	0	0	0
	6–16	1.38	−0.76 to 3.52	0.133
	16–38	1.92	−016 to 4.00	0.061
	>38	2.57	0.52 to 4.62	**0**.**028**
Haloperidol	<6	0	0	0
	6–16	1.07	0.08 to 2.06	**0**.**036**
	>38	1.88	0.82 to 2.95	**0**.**002**
Lurasidone	<6	0	0	0
	>38	0.59	−0.53 to 1.70	0.287
Olanzapine	<6	0	0	0
	6–16	0.41	0.02 to 0.79	**0**.**038**
	16–38	0.33	−0.08 to 0.75	0.116
	>38	0.96	0.51 to 1.41	**<0**.**001**
Paliperidone	<6	0	0	0
	6–16	0.24	−0.24 to 0.72	0.322
	16–38	0.87	0.36 to 1.38	**0**.**002**
	>38	1.07	0.59 to 1.55	**<0**.**001**
Quetiapine	<6	0	0	0
	6–16	−0.24	−0.73 to 0.25	0.327
	16–38	0.14	−0.76 to 1.05	0.751
	>38	0.78	−0.10 to 1.66	0.082
Risperidone	<6	0	0	0
	6–16	0.33	−0.26 to 0.92	0.266
	16–38	0.58	−0.18 to 1.34	0.129
	>38	0.90	0.29 to 1.51	**0**.**005**
Second-generation antipsychotics	6–16	0	0	0
	16–38	0.47	−1.97 to 2.90	0.586
	>38	0.99	−1.65 to 3.62	0.319
Ziprasidone	<6	0	0	0
	6–16	0.26	−2.00 to 2.52	0.778
	16–38	−0.33	−2.93 to 2.28	0.759
	>38	0.07	−2.77 to 2.92	0.949
Placebo	<6	0	0	0
	6–16	0.09	−0.31 to 0.49	0.644
	16–38	1.14	0.54 to 1.74	**<0**.**001**
	>38	0.85	0.24 to 1.45	**0**.**007**

a. The period <6 weeks was the reference period. If no data were available for this period, the period 6–16 weeks was used as reference period instead. Significant P-values are marked in bold.

Only outcomes that could be analysed are noted here.

### CRWL in antipsychotic-switch studies

Of the 202 studies included in this meta-analysis, only 45 reported CRWL (116 records in the meta-analysis data). Data were available for amisulpride, aripiprazole, asenapine, haloperidol, lurasidone, olanzapine, paliperidone, quetiapine, risperidone, second-generation antipsychotics, ziprasidone and placebo. Overall, the proportion of patients who lost ≥7% of body weight was rather modest. After 38 weeks of treatment, CRWL was apparent for aripiprazole (9.1%, 95% CI 4.8–14.4), olanzapine (10.8%, 95% CI 4.1–20.0), lurasidone (12.6%, 95% CI 9.6–16.2) and ziprasidone (28.6%, 95% CI 13.2–48.7). In the placebo condition, 12.0% (95% CI 7.0–1.8) of the patients lost ≥7% of their body weight after 38 weeks of treatment. Further details and forest plots can be found in Supplementary Appendix 6. Results for antipsychotics for which data were available for only one exposure period are presented in Supplementary Appendix 7. The *I*-squared for the included studies ranged from 0.0 to 94.86%, indicating little to strong heterogeneity (Supplementary Appendix 6.1).

### CRWL in antipsychotic-switch studies and duration of antipsychotic use

**Fig. 2:**
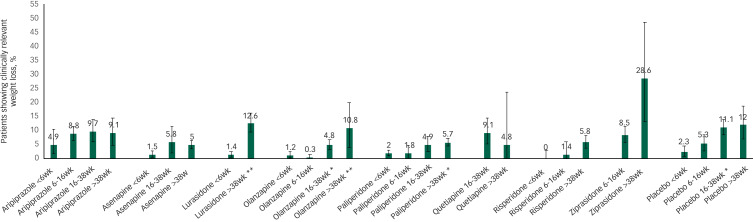
Clinically relevant weight loss per antipsychotic per time period. Only antipsychotics with ≥2 data points are displayed. Significant differences from the reference period <6 weeks are marked: *P < 0.05 **P < 0.01. wk, weeks.

Figure 2 shows CRWL by treatment duration. Because available data are limited, we could perform meta-regression analyses for only a limited subset of antipsychotics: aripiprazole, asenapine, lurasidone, olanzapine and paliperidone. Only in lurasidone (>38 weeks), olanzapine (>38 weeks) and paliperidone (>38 weeks) the proportion of CRWL was significantly higher than in the exposure period <6 weeks. No other findings were significant. Full details are presented in Table 2.

**Table 2:** Meta-regression analysis: effect of treatment duration on clinically relevant weight loss

Antipsychotic	Period, weeks	*B*	95% CI	*P*
Aripiprazole	<6^a^	0	0	0
	6–16	0.64	−0.74 to 2.01	0.345
	16–38	0.80	−0.61 to 2.20	0.248
	>38	0.64	−1.02 to 2.31	0.427
Asenapine	<6	0	0	0
	16–38	1.29	−0.08 to 2.67	0.061
	>38	1.12	−0.60 to 2.84	0.155
Lurasidone	<6	0	0	0
	>38	2.11	1.17 to 3.04	**0**.**003**
Olanzapine	<6	0	0	0
	6–16	0.37	−1.76 to 2.50	0.709
	16–38	1.05	−0.00 to 2.10	**0**.**050**
	>38	1.82	0.73 to 2.92	**0**.**004**
Paliperidone	<6	0	0	0
	6–16	−0.18	−1.64 to 1.28	0.786
	16–38	0.67	−0.28 to 1.63	0.145
	>38	0.83	0.09 to 1.57	**0**.**032**
Placebo	<6	0	0	0
	6–16	0.30	−0.50 to 1.11	0.435
	16–38	1.14	0.29 to 1.98	**0**.**011**
	>38	1.17	−0.21 to 2.55	0.092

a. The period <6 weeks was the reference period. Significant P-values are marked in bold.

Only outcomes that could be analysed are noted here.

### Antipsychotic-naive versus antipsychotic-switch

Only seven of the included studies^47–53^ reported on CRWG in antipsychotic-naive patients (19 records) and no studies reported on CRWL in that population. For aripiprazole (6–16 weeks), olanzapine (<6 weeks, 6–16 weeks, >38 weeks) and quetiapine (6–16 weeks) more antipsychotic-naive patients than switch patients had CRWG (Table 3). Differences for haloperidol, risperidone and ziprasidone were not statistically significant. The remaining antipsychotics could not be analysed.

**Table 3:** Meta-regression: difference in proportion showing clinically relevant weight gain (CRWG) between antipsychotic-naive and antipsychotic-switch patients

Period	Aripiprazole, OR (95% CI)	Haloperidol, OR (95% CI)	Olanzapine, OR (95% CI)	Quetiapine, OR (95% CI)	Risperidone, OR (95% CI)	Ziprasidone, OR (95% CI)
≤6 weeks	1.42 (0.37–5.39)	n.a.	2.43 (1.07–5.54)*	n.a.	1.18 (0.39–3.55)	n.a.
6–16 weeks	7.33 (2.47–21.76)***	3.14 (0.09–108.26)	4.49 (2.31–8.72)***	6.69 (1.98–22.64)**	3.03 (0.44–20.63)	2.42 (0.26–22.50)
16–38 weeks	0.75 (0.11–5.20)	n.a.	n.a.	n.a.	n.a.	n.a.
≥38 weeks	n.a.	3.72 (0.24–58.20)	4.98 (1.70–14.60)**	n.a.	n.a.	n.a.

n.a., no data available for antipsychotic-naive CRWG. The outcomes in bold indicate significant differences in CRWG in antipsychotic-naive compared with the reference group (antipsychotic-switch).

*P < 0.05, **P < 0.01, ***P < 0.001.

### CRWG and association with diagnosis

Table 4 presents associations between CRWG and diagnostic category, stratified by antipsychotic and duration of exposure. People with bipolar disorder receiving aripiprazole, cariprazine or olanzapine experienced significantly less CRWG in the short term (<6 weeks) compared with those with schizophrenia. In addition, people with dementia receiving olanzapine or risperidone experienced significantly less CRWG in the medium term (6–16 weeks) compared with those with schizophrenia. Finally, people with major depressive disorder, borderline personality disorder or generalised anxiety disorder receiving quetiapine had significantly less CRWG (<6 weeks, 6–16 weeks) compared with those with schizophrenia. All other results were not significant (Table 4 and Supplementary Appendix 8).

**Table 4:** Meta-regression: clinically relevant weight gain per diagnosis per period

Antipsychotic	Period, weeks	Diagnosis	B	95% CI	*P*
Aripiprazole		Schizophrenia^a^	0		
	<6	Schizophrenia spectrum	0.27	−0.22–0.75	0.261
		Bipolar disorder	−1.72	−2.62–-0.82	**0**.**001**
		Other diagnosis	0.29	−0.21–0.78	0.238
	6–16	Schizophrenia spectrum	−0.26	−1.57–1.05	0.679
		Bipolar disorder	−0.68	−2.19–0.82	0.346
		Dementia	−0.86	−2.18–0.45	0.182
	16–38	Bipolar disorder	0.13	−1.17–1.43	0.829
	>38	Bipolar disorder	0.20	−1.17–1.58	0.719
Asenapine		Schizophrenia	0		
	<6	Schizophrenia spectrum	−0.60	−2.26–1.06	0.434
		Bipolar disorder	0.05	−0.47–0.57	0.835
	16–38	Bipolar disorder	0.53	−0.23–1.29	0.141
Brexpiprazole		Schizophrenia	0		
	<6	Other diagnosis	−0.60	−1.41–0.21	0.124
Cariprazine		Schizophrenia	0		
	<6	Bipolar disorder	−1.37	−2.07–−0.66	**0**.**002**
Haloperidol		Schizophrenia	0		
	<6	Schizophrenia spectrum	0.32	−1.06–1.70	0.577
		Bipolar disorder	−0.20	−2.04–1.65	0.794
Lurasidone		Schizophrenia	0		
	<6	Bipolar disorder	−0.95	− 1.79–−0.12	**0**.**028**
Olanzapine		Schizophrenia	0		
	<6	Schizophrenia spectrum	−0.43	−1.30–0.44	0.312
		Bipolar disorder	−0.71	−1.26–−0.16	**0**.**014**
	6–16	Schizophrenia spectrum	−0.29	−0.90–0.33	0.346
		Bipolar disorder	−0.19	−1.07–0.69	0.663
		Other diagnosis	0.02	−0.91–0.87	0.967
		Dementia	−1.79	−3.29–−0.29	**0**.**021**
	16–38	Schizophrenia spectrum	−0.49	−1.45–0.46	0.288
		Dementia	−1.01	−2.42–0.40	0.150
	>38	Schizophrenia spectrum	0.51	−0.43–1.46	0.256
		Bipolar disorder	−0.17	−0.99–0.65	0.658
Paliperidone		Schizophrenia	0		
	<6	Schizophrenia spectrum	−0.50	− 1.43–0.42	0.262
		Bipolar disorder	−0.71	−1.74–0.32	0.158
	6–16	Bipolar disorder	−0.16	−0.94–0.61	0.644
	16–38	Bipolar disorder	1.00	−0.61–2.61	0.161
	>38	Schizophrenia spectrum	−0.46	−1.66–0.75	0.376
Quetiapine		Schizophrenia	0		
	<6	Bipolar disorder	−0.92	−2.74–0.89	0.302
		Other diagnosis	−1.14	−2.09–−0.20	**0**.**021**
	6–16	Schizophrenia spectrum	0.67	−0.36–1.69	0.189
		Bipolar disorder	−0.09	−0.90–0.72	0.816
		Other diagnosis	−1.25	−2.17–−0.34	**0**.**010**
Risperidone		Schizophrenia	0		
	<6	Schizophrenia spectrum	−0.07	−1.29–1.14	0.892
	6–16	Schizophrenia spectrum	−0.13	−1.41–1.15	0.828
		Bipolar disorder	−0.48	−2.43–1.46	0.601
		Dementia	−3.58	−6.35–−0.81	**0**.**015**
	16–38	Dementia	−0.73	−1.78–0.31	0.123
	>38	Schizophrenia spectrum	−0.49	−1.60–0.62	0.342
		Bipolar disorder	−0.08	−1.10–0.93	0.86
Placebo		Schizophrenia	0		
	<6	Schizophrenia spectrum	−0.27	−1.03–0.49	0.482
		Bipolar disorder	−0.55	−1.11–−0.01	0.053
		Other diagnosis	−0.95	−1.68–−0.23	**0**.**011**
	6–16	Bipolar disorder	−0.95	−1.58–−0.32	**0**.**004**
		Other diagnosis	−1.34	−2.20–−0.49	**0**.**003**
		Dementia	−0.67	−1.49–−0.15	0.104
	16–38	Bipolar disorder	0.72	−2.44–3.88	0.599
		Dementia	−0.79	−5.07–3.49	0.668
	>38	Schizophrenia spectrum	0.27	−3.03–3.58	0.847
		Bipolar disorder	0.52	−1.66–2.70	0.580

a. Schizophrenia was the reference diagnosis. Other diagnosis groups were schizophrenia spectrum disorder, bipolar disorder, dementia and other diagnosis. Significant P-values are marked in bold.

Only outcomes that could be analysed are noted here.

### Sensitivity analyses

After excluding studies examining antipsychotic-naive patients (seven studies), results were essentially unchanged and in the same direction. For blonanserin (16–38 weeks), analyses could not be repeated as data were available only for antipsychotic-naive patients. After excluding studies that examined patients older than 65 years (nine studies), the results were also unchanged, except for brexpiprazole (6–16 weeks). Here, data were available only for patients older than 65 years.

After the removal of studies with at least one domain with a high risk of bias (five studies), the results for CRWG changed only for ziprasidone (<6 weeks: 0%, 95% CI 0.0–2.8). After excluding the high risk of bias studies, for iloperidone available data were not sufficient to perform an analysis (<6 weeks). For CRWL, the results did not change. For quetiapine (>38 weeks) and risperidone (<6 weeks), no more data were available. In the analyses of the other antipsychotics, the exclusion of these studies did not significantly affect our results (Supplementary Appendix 9).

### Publication bias

Funnel plots and Egger tests showed some evidence of publication bias, specifically for antipsychotics with fewer studies (Supplementary Appendix 10). For CRWG, trim-and-fill added only a single study for asenapine (>38 weeks), chlorpromazine (<6 weeks), clozapine (16–38 weeks, >38 weeks), risperidone (>38 weeks), second-generation antipsychotics (>38 weeks) and ziprasidone (16–38 weeks). Pooled estimates and *P*-values were similar to the original analyses (Supplementary Files 10.1 and 10.3). For clozapine, the trim-and-fill analysis was unable to calculate pooled estimates. Funnel plots with low numbers of studies could not be interpreted. For CRWL, trim-and-fill added two studies for aripiprazole (6–16 weeks) and one study for asenapine (<6 weeks), risperidone (6–16 weeks) and placebo (16–38 weeks) with similar pooled estimates and *P*-values as the original analyses (Supplementary Files 10.2 and 10.4).

## Discussion

The present results showed that almost all included antipsychotics were associated with CRWG. In addition, among the few antipsychotics with sufficient data, some were associated with CRWL. For some antipsychotics, CRWG was greater in antipsychotic-naive than in antipsychotic-switch patients. Moreover, a longer duration of antipsychotic use was associated with more CRWG, but not with CRWL. For some antipsychotics, CRWG was greater for patients diagnosed with schizophrenia, but this was not consistent.

### CRWG

Because we analysed a wide variety of antipsychotics, CRWG per antipsychotic can be assessed. However, with the current methods it was impossible to compare CRWG between different antipsychotics. The reader should keep this in mind when reading different percentages for antipsychotics below. Network meta-analysis using the present data-set can enable those conclusions in the future (also see the Methodological issues section below). CRWG was severe for clozapine and olanzapine, with respectively 76.3% and 36.9% of patients gaining ≥7% body weight after 38 weeks of use. After prolonged use, paliperidone (18.3%), quetiapine (17.0%) and risperidone (23.0%) showed moderate percentages of CRWG, as did so-called metabolically friendly antipsychotics: aripiprazole (17.2%), amisulpride (20.6%), asenapine (16.4%) and haloperidol (22.8%). This may seem counterintuitive, as most studies and systematic reviews report the average weight gain in a study population, which may obscure the true magnitude of antipsychotic-induced weight gain. An average weight change is calculated by averaging weight gain in some patients and weight loss in others and may also be distorted by outliers in a small proportion of patients. When focusing on average weight change, some antipsychotics may appear metabolically friendly, i.e. on average do not lead to much weight gain, but this average does not apply to all patients. Thus, even if an antipsychotic is generally considered metabolically friendly and associated with limited weight gain or even CRWL in some patients, other patients using it may still experience CRWG. Proportions showing CRWG were relatively low for lurasidone (7.4%) and ziprasidone (7.6%) after 38 weeks of use. Proportions showing CRWG were similar to placebo (6.7%), but available data for lurasidone and ziprasidone were limited. Of the newer antipsychotics, brexpiprazole and cariprazine were promising for the short term, but little or no data were available for the long term (Table 1).

### CRWG and duration of antipsychotic use

Visual inspection of Fig. 1 shows that CRWG continues to increase with longer antipsychotic use, except for ziprasidone and brexpiprazole. In a subset of antipsychotics with sufficient data, meta-regression analyses showed that a longer duration of use was associated with more CRWG for most antipsychotics (Table 2). This is also the case for so-called metabolically friendly antipsychotics such as aripiprazole, asenapine and haloperidol, as well as the newer compound cariprazine. The result for cariprazine should be interpreted with caution as it was based on a single study. However, placebo was also associated with CRWG in studies exceeding 16 weeks.

Previous systematic reviews and meta-analyses focused mainly on short-term weight gain or did not distinguish between short-term and long-term studies.^24,25,28–32^ In contrast, our findings suggest that time is a relevant factor in antipsychotic-induced weight gain. Unfortunately, a majority of studies included in this meta-analysis focused on the short term (<6 weeks; 6–16 weeks), and only about 15% of the studies focused on the long-term. The availability of long-term data is limited and suggests that the true impact of long-term antipsychotic use on weight gain remains hidden. Conducting long-term studies or longitudinal studies in this patient population presents methodological and other challenges but is essential to obtain a complete picture of antipsychotic-induced weight gain. We believe that non-randomised studies will better answer this question. We plan to conduct a systematic review and meta-analysis on such studies in the near future. However, non-randomised studies also have methodological shortcomings.

Our findings suggest that the duration of antipsychotic use is an important determinant of weight gain, highlighting the importance of monitoring weight gain and weight management strategies from the start and throughout antipsychotic treatment.^17,28,32^

### CRWL after antipsychotic switch

CRWL was noticeable for aripiprazole (9.1%), lurasidone (12.6%) and ziprasidone (28.6%), but also olanzapine (10.8%) after 38 weeks of treatment. Except for ziprasidone, CRWL for these antipsychotic was similar to CRWL for placebo (12.0%). Data, however, are too limited to draw firm conclusions. For lurasidone and ziprasidone, long-term results were based on a single study (Table 3). The effect of treatment duration on weight loss could be tested for only a limited subset of antipsychotics: aripiprazole, asenapine, olanzapine and paliperidone. For olanzapine and paliperidone, a longer duration of antipsychotic use was associated with more CRWL (Table 4).

Recently, much research has been done on strategies to prevent or counteract antipsychotic-induced weight gain. Behavioural therapies, exercise programmes, dietary counselling and pharmacological interventions such as metformin and topiramate may have a moderate effect on reducing weight gain.^2,7,54–56^ A switch to so-called metabolically friendly antipsychotics (amisulpride, aripiprazole, haloperidol, lurasidone, ziprasidone) might also result in weight loss.^7,23–25^ In a recent meta-analysis of antipsychotic-switch studies, switching to aripiprazole and ziprasidone was associated with weight loss, but a switch to amisulpride, paliperidone, risperidone, quetiapine or lurasidone was not.^57^ However, switching to a metabolically friendly antipsychotic should be done with caution and always involves uncertainties, for instance about the effect on symptoms and side-effects. In addition, there is evidence that weight gain and other metabolic side-effects appear to be associated with more clinical improvement.^25,58^ Our meta-analysis shows modest CRWL for aripiprazole, lurasidone and olanzapine, comparable with placebo. Surprisingly, although olanzapine is known to be associated with weight gain, and the present data showed that the proportion of patients showing CRWG increased, in other patients CRWL increased over time. However, this result was strongly influenced by one long-term study with a 2-year follow-up, comparing long-acting injection and oral administration, but with no significant differences between both groups.^59^ Ziprasidone appears promising for long-term CRWL, but these results were based on one study^46^ and need replication in larger patient populations to draw firm conclusions. Based on these results, there is insufficient evidence to recommend switching antipsychotic as a weight management strategy.

In contrast to current guidelines that recommend switching to a metabolically friendly antipsychotic as the first step in controlling antipsychotic-induced weight gain, we strongly recommend considering alternative strategies for losing body weight. Focus first on individual lifestyle counselling and exercise interventions, possibly supplemented with metformin, topiramate or bupropion, before switching to another antipsychotic.^15,54–56^

### Antipsychotic-naive versus antipsychotic-switch populations

Recent network meta-analyses did not distinguish between antipsychotic-naive patients and antipsychotic-switch patients.^24,25^ However, this distinction is relevant because antipsychotic-naive patients are more likely to develop antipsychotic-induced weight gain, as previous meta-analyses^20,31^ have shown. Moreover, weight gain in antipsychotic-naive patients is much more indicative of the effect of a specific antipsychotic on weight, whereas in switch studies the effect of a previous antipsychotic can certainly not be excluded for the short term and therefore leads to bias.^60^ We could include only a few studies that assessed CRWG and no studies that assessed CRWL in an antipsychotic-naive population. We excluded multiple studies that had data on 7% weight change but were either not randomised, used a per-protocol analysis or included patients younger than 15 years. Antipsychotic-naive patients taking aripiprazole, olanzapine and quetiapine showed significantly more CRWG than switch patients (Table 3). Results for haloperidol, risperidone and ziprasidone were not statistically significant. These results are consistent with previous findings indicating that antipsychotic-naive patients are particularly vulnerable to excessive weight gain.^20,31^ This is probably because the antipsychotic-naive patients were not overweight at baseline as there is no effect of prior antipsychotic use on body weight, and because they are younger.^33,61–63^

Because weight gain is a predictor of non-adherence as well as metabolic and cardiovascular dysregulation,^5,64^ assessment of the effect of antipsychotics on weight gain is necessary and requires long-term RCTs and prospective studies. Until recently, few pharmacological studies had investigated the efficacy and side-effects of antipsychotics exclusively in populations with first-episode psychosis.^60^ In addition, many people with first-episode psychosis struggle with substance misuse or depression or use co-medications such as benzodiazepines or antidepressants, which often exclude them from participation in an RCT.^62,63^ A recent analysis of two national patient registries estimated that only about 20% of patients with schizophrenia spectrum disorders may be represented in RCTs.^65^ Studies with a naturalistic design then have the advantage of investigating antipsychotic-induced weight gain in a realistic setting and may help us to explain underlying mechanisms that influence it.^60^

### Diagnosis

We found some evidence that diagnosis moderates CRWG (Table 4). People with bipolar disorder taking aripiprazole, cariprazine, lurasidone or olanzapine showed less CRWG compared with those with schizophrenia (<6 weeks). People with major depressive disorder, generalised anxiety disorder or borderline personality disorder treated with quetiapine showed less CRWG than whose with schizophrenia (<16 weeks). People with dementia taking olanzapine or risperidone also showed less CRWG (6–16 weeks). These differences in CRWG between schizophrenia and other diagnoses were visible only in the short term.

Previously, we found no evidence that diagnosis moderates weight gain.^17^ However, in this study, there is weak evidence that diagnosis is of influence. This may be explained by the fact that only the extremes of a spectrum, i.e. individuals who gain or lose ≥7% of their body weight, were examined here. Even if an antipsychotic is, on average, associated with no or minimal weight gain, some individuals may still experience CRWG. For example, a subgroup of individuals with schizophrenia may experience more short-term CRWG than individuals with other psychiatric diagnoses, despite the fact that there was no significant difference in weight gain as a continuous measure between diagnoses.^17^

Previous research has been inconclusive about the role of diagnosis in antipsychotic-induced weight gain. Some studies concluded that people with schizophrenia were at greater risk of developing metabolic syndrome or diabetes mellitus than people with bipolar disorder.^66^ Other studies found no differences in weight gain between the diagnoses.^67–69^ Several studies have investigated the possibility of a shared genetic predisposition between schizophrenia and weight gain, but the results are still inconclusive.^70^

Regardless of the influence of diagnosis on antipsychotic-induced weight gain, the results of our meta-analysis indicate that a substantial proportion of patients will experience CRWG with all antipsychotics.

### Methodological issues

Our comprehensive meta-analysis included 202 papers addressing the use of antipsychotics and clinically relevant weight change. Another strength is that all RCTs irrespective of study duration or psychiatric diagnosis were included. However, several limitations should be kept in mind when interpreting the results of this meta-analysis.

First, our meta-analysis focused exclusively on CRWG or CRWL, but relevant studies did not systematically report CRWG and CRWL. Therefore, we excluded multiple studies that met all other inclusion criteria. Nevertheless, we consider this meta-analysis to be a useful and valuable addition to previous analyses as it provides information on the severe body weight changes associated with antipsychotic use and as such may guide clinical decision-making. Despite the representation problems in RCTs,^65^ we assume that the studies that reported CRWG and CRWL are sufficiently representative and generalisable to real-world patient populations.

Second, this meta-analysis was intended to give an overview of individual antipsychotic drugs, not to compare them. With the analysis method we used, comparisons between interventions (including placebo) were not possible. Direct comparison of weight change between antipsychotics would require novel analysis methods such as a network meta-analysis. Given the stratified structure of the data, i.e. studies were included with multiple time points and multiple antipsychotics, and the heterogeneity of the included studies and patient groups, this was not possible within the scope of the current meta-analysis.^71^ Previous network meta-analyses examining CRWG included only short-term studies (≤13 weeks)^24^ or analysed data at the individual patient level.^30^

Third, we included only randomised studies in this meta-analysis because this design has the lowest risk of bias and the highest methodological quality at the expense of ecological validity. We excluded multiple cohort studies, which often follow large study populations for a prolonged period. The majority of studies included in this meta-analysis were of relatively short duration, whereas our results clearly showed that weight change continues even after 38 weeks of treatment. Cohort studies also include patients with comorbidities and co-medication often excluded in RCTs.^28^ A systematic review or meta-analysis based on cohort studies could therefore provide a better understanding of the long-term effects of antipsychotics on weight gain in large study populations. Meta-analyses such as the present one including only RCTs and meta-analyses including observational studies both have their own merits. Since both types of meta-analysis are needed to obtain a complete overview, a meta-analysis including observational studies is needed in the near future.

Fourth, in this meta-analysis we could include only a handful of studies that examined clinically relevant weight change in antipsychotic-naive patients. Studies in antipsychotic-naive patients were more often excluded. Therefore, results should be interpreted with caution. It is clear that more research is needed in the antipsychotic-naive population. Research on antipsychotic-induced weight gain in this population is highly relevant because, as first-time users, previous use of an antipsychotic does not affect weight gain. Moreover, they are more vulnerable to excessive weight gain because of their lower age and lower BMI.^62^

Furthermore, a meta-analysis using data reported in original papers can assess only what the studies analysed. Results controlled for confounders (such as age and baseline BMI) and results stratified by a modifier (such as antipsychotic dose) can be extracted only when the original studies reported them. For example, antipsychotic dose differed within one arm of RCTs and therefore antipsychotic dose could not be analysed as a modifier. Only when the RCTs included study arms with different dosages or when the dosage differed between studies could this be analysed in our meta-analysis. For some antipsychotics (olanzapine, clozapine), preliminary evidence indicates a dose–response relationship between serum concentrations and metabolic outcomes; for other antipsychotics, the evidence for such a dose–response relationship is limited.^72^ New original research could shed more light on this. We could not correct for antipsychotic drug use before participation in the trials. As most studies included here are antipsychotic-switch studies, a recording of the pre-switch antipsychotic would have been important. However, these pre-switch antipsychotics were not given or were listed as a percentage of which antipsychotic was previously prescribed. Except for a recent meta-analysis on antipsychotic switching,^57^ pre-switch medication is usually not included in meta-analyses. Interpreting the data is arduous^32^ and underscores the relevance of investigating antipsychotic-induced weight gain in an antipsychotic-naive population.

The present meta-analysis aimed to be comprehensive and thus to include all RCTs. The RCTs were not restricted to a specific diagnosis, age group or study duration. Despite the fact that all antipsychotics were analysed separately, the data showed a high degree of heterogeneity and results should therefore be interpreted with caution. However, because of the heterogeneity, the data were analysed using the random-effects option.

We included only articles written in English because it would be difficult to judge the methodological and outcome properties otherwise. A few studies from China or other Asian countries were therefore excluded. A meta-analysis found Asian patients to have a lower BMI and lower antipsychotic-induced weight gain than Western patients.^31^ Another meta-analysis excluded Chinese studies because of a higher risk of methodological shortcomings.^24^ We did include studies from China written in English. We did evaluate those articles as methodologically sound and they had all been published in peer-reviewed journals.

Finally, we did not include unpublished work in our meta-analysis, and studies with null findings tend to remain unpublished. We checked poster presentations and conference abstracts to partly overcome this. We found some evidence of publication bias, particularly for antipsychotics with few available studies. Where possible, *post hoc* trim-and-fill analyses were performed to control for this, but the pooled estimates and *P*-values were similar to the original analyses.

### Clinical implications

Switching antipsychotics can lead to both clinically relevant weight gain and weight loss, although weight gain is more prominent. Switching to a metabolically friendly antipsychotic may help patients to lose some weight, but it does not appear to be very effective in substantially reducing body weight and carries the risk of worsening symptoms.^15,57^ Clinically relevant weight gain is more pronounced in antipsychotic-naive patients, which emphasises the importance of immediate follow-up. Prevention of antipsychotic-induced weight gain and subsequent complications ultimately appears to be the best strategy and should be the focus of treatment. Careful consideration of possible side-effects of antipsychotics and individual risk factors for increased vulnerability to weight gain is crucial before initiating antipsychotic treatment.

## Data Availability

The data that support the findings of this study are available from the corresponding author (B.C.) on reasonable request.

## Supplementary material

For supplementary material accompanying this paper visit http://doi.org/10.1192/bjo.2022.619. click here to view supplementary material
