# Supplementary material for: Association between antipsychotic medication and clinically relevant weight change: meta-analysis
Source: BJPsych Open. 2023 Jan 18;9(1):e18. doi: 10.1192/bjo.2022.619 (PMC9885350; doi:10.1192/bjo.2022.619)
Supplement: Supplementary file 1 [file S2056472422006196sup001.docx]

Supplementary Materials

[Appendix 1: PRISMA 3](#_Toc101811634)

[Appendix 1.1: PRISMA 2020 Flow Diagram 3](#_Toc101811635)

[Appendix 2: Search Terms 4](#_Toc101811636)

[Appendix 2.1: Pubmed Search Terms 4](#_Toc101811637)

[Appendix 2.2: Embase Search Terms 4](#_Toc101811638)

[Appendix 2.3: PsycINFO Search Terms 4](#_Toc101811639)

[Appendix 3: Included studies 5](#_Toc101811640)

[Appendix 3.1: Table of included studies 5](#_Toc101811641)

[Appendix 3.2: References of included studies 23](#_Toc101811642)

[Appendix 4: Excluded studies 34](#_Toc101811643)

[Appendix 4.1: Table of excluded studies after full text assessment 34](#_Toc101811644)

[Appendix 4.2: References of excluded studies 43](#_Toc101811645)

[Appendix 5: CRWG 61](#_Toc101811646)

[Appendix 5.1: Results meta-analysis CRWG 61](#_Toc101811647)

[Appendix 5.2: Forest plots of CRWG 63](#_Toc101811648)

[Supplementary file 5.2.1: Amisulpride 63](#_Toc101811649)

[Supplementary file 5.2.2: Aripiprazole 64](#_Toc101811650)

[Supplementary file 5.2.3: Asenapine 65](#_Toc101811651)

[Supplementary file 5.2.4: Blonanserin 66](#_Toc101811652)

[Supplementary file 5.2.5: Brexpiprazole 67](#_Toc101811653)

[Supplementary file 5.2.6: Cariprazine 68](#_Toc101811654)

[Supplementary file 5.2.7: Chlorpromazine 69](#_Toc101811655)

[Supplementary file 5.2.8: Clozapine 70](#_Toc101811656)

[Supplementary file 5.2.9: FGA 71](#_Toc101811657)

[Supplementary file 5.2.10: Haloperidol 72](#_Toc101811658)

[Supplementary file 5.2.11: Iloperidone 73](#_Toc101811659)

[Supplementary file 5.2.12: Lurasidone 74](#_Toc101811660)

[Supplementary file 5.2.13: Olanzapine 75](#_Toc101811661)

[Supplementary file 5.2.14: Paliperidone 76](#_Toc101811662)

[Supplementary file 5.2.15: Placebo 77](#_Toc101811663)

[Supplementary file 5.2.16: Quetiapine 78](#_Toc101811664)

[Supplementary file 5.2.17: Risperidone 79](#_Toc101811665)

[Supplementary file 5.2.18: Sertindole 80](#_Toc101811666)

[Supplementary file 5.2.19: SGA 81](#_Toc101811667)

[Supplementary file 5.2.20: Ziprasidone 82](#_Toc101811668)

[Appendix 6: CRWL 83](#_Toc101811669)

[Appendix 6.1: Results meta-analysis CRWL 83](#_Toc101811670)

[Appendix 6.2: Forest plots of CRWL 84](#_Toc101811671)

[Supplementary file 6.2.1: Aripiprazole 84](#_Toc101811672)

[Supplementary file 6.2.2: Asenapine 85](#_Toc101811673)

[Supplementary file 6.2.3: Lurasidone 86](#_Toc101811674)

[Supplementary file 6.2.4: Olanzapine 87](#_Toc101811675)

[Supplementary file 6.2.5: Paliperidone 88](#_Toc101811676)

[Supplementary file 6.2.6: Placebo 89](#_Toc101811677)

[Supplementary file 6.2.7: Quetiapine 90](#_Toc101811678)

[Supplementary file 6.2.8: Risperidone 91](#_Toc101811679)

[Supplementary file 6.2.9: Ziprasidone 92](#_Toc101811680)

[Appendix 7. Results for AP with data for only 1 period 93](#_Toc101811681)

[Supplementary table 7.1. CRWG for AP with data for only 1 period 93](#_Toc101811682)

[Supplementary table 7.2. CRWL for AP with data for only 1 period 93](#_Toc101811683)

[Appendix 8: Diagnosis 94](#_Toc101811684)

[Supplementary table 8.1: Overview psychiatric diagnoses of included studies and grouped diagnoses 94](#_Toc101811685)

[Appendix 9: Sensitivity analyses 95](#_Toc101811686)

[Supplementary table 9.1: Sensitivity meta-analysis CRWG without AP-naive population 95](#_Toc101811687)

[Supplementary table 9.2: Sensitivity meta-analysis CRWG without population >65 years 97](#_Toc101811688)

[Supplementary table 9.3: Sensitivity analysis meta-regression CRWG without AP-naive population and population >65 years 99](#_Toc101811689)

[Supplementary table 9.4: Sensitivity meta-analysis CRWL without population > 65 years 101](#_Toc101811690)

[Supplementary table 9.5: Sensitivity analysis meta-regression CRWL without population > 65 years 102](#_Toc101811691)

[Supplementary table 9.6: Sensitivity meta-analysis CRWG without studies with high Risk of Bias 103](#_Toc101811692)

[Supplementary table 9.7: Sensitivity analysis meta-regression CRWG without studies with high Risk of Bias 105](#_Toc101811693)

[Supplementary table 9.8: Sensitivity meta-analysis CRWL without studies with high Risk of Bias 107](#_Toc101811694)

[Supplementary table 9.9: Sensitivity analysis meta-regression CRWL without studies with high Risk of Bias 108](#_Toc101811695)

[Appendix 10: Publication Bias 109](#_Toc101811696)

[Supplementary file 10.1: Funnel plots CRWG stratified by AP and study duration 109](#_Toc101811697)

[Supplementary file10.2: Funnel plots CRWL stratified by AP and study duration 113](#_Toc101811698)

[Supplementary file 10.3: CRWG Egger tests coefficient and p-value and added studies by trim-and-fill procedure 115](#_Toc101811699)

[Supplementary file 10.4: CRWL Egger tests coefficient and p-value and added studies by trim-and-fill procedure 117](#_Toc101811700)

[Appendix 11: Risk of Bias Assessment 118](#_Toc101811701)

[Supplementary table 11.1: Risk of Bias assessment for the individual domains 118](#_Toc101811702)

[Supplementary table 11.2: Risk of Bias assessment for the individual studies 119](#_Toc101811703)

[Supplementary table 11.3: Certainty of evidence assessment (GRADE) 125](#_Toc101811704)

# Appendix 1: PRISMA

## Appendix 1.1: PRISMA 2020 Flow Diagram

**Identification of studies via databases and registers**

Records removed *before screening*:

Duplicate records removed (n = 588)

Records marked as ineligible by automation tools (n = 0)

Records removed for other reasons (n = 0)

Records identified from*:

Databases (n = 2363)

Pubmed (n = 918)

Embase (n = 1393)

PsycINFO (n = 52)

Registers (n = 0)

Other sources (n = 47)

**Identification**

Records excluded**:

(n = 1265)

No randomized design (n = 413)

Adjunctive therapy (n = 301)

No AP (n = 163)

Pooled dataset/ post-hoc analyses (n = 125)

Age <15 (n = 192)

Review/ meta-analysis (n = 23)

Eating disorders (n = 21)

Study duration < 1 week (n = 14)

Language (n = 8)

Other reasons (n = 5)

)

- Adjunctive therapy (298)
- No AP (143)
- Pooled dataset/ post-hoc analyses (134)
- Age <15 (194)
- Review/ meta-analysis (24)
- Eating disorders (23)
- Study duration < 1 week (12)
- Language (8)
- Other reasons (6)

Records screened

(n = 1822)

**Screening**

Reports sought for retrieval

(n = 557)

Reports not retrieved

(n = 0)

Reports excluded: (n = 355)

No 7% weight data (n = 329)

No ITT analysis (n = 18)

Pooled dataset/ post-hoc analyses (n = 3)

Studies already included (n = 3)

Language (n = 2)

Reports assessed for eligibility

(n = 557)

Studies included in review

(n = 202)

Reports of included studies

(n = 202)

**Included**

*Consider. if feasible to do so. reporting the number of records identified from each database or register searched (rather than the total number across all databases/registers).

**If automation tools were used. indicate how many records were excluded by a human and how many were excluded by automation tools.

*From:*  Page MJ. McKenzie JE. Bossuyt PM. Boutron I. Hoffmann TC. Mulrow CD. et al. The PRISMA 2020 statement: an updated guideline for reporting systematic reviews. BMJ 2021;372:n71. doi: 10.1136/bmj.n71

For more information. visit: <http://www.prisma-statement.org/>

# Appendix 2: Search Terms

## Appendix 2.1: Pubmed Search Terms

1. **(((((change OR gain OR clinically relevant OR body) AND weight)) AND (((((((((((((((((((((((((((((((((((amisulpride) OR amoxapine) OR aripiprazole) OR asenapine) OR blonanserin) OR brexpiprazole) OR bromperidol) OR cariprazine) OR chlorpromazine) OR clozapine) OR flupentixol) OR fluphenazine) OR haloperidol) OR iloperidone) OR levopromazine) OR lurasidone) OR melperone) OR olanzapine) OR paliperidone) OR penfluridol) OR zotepine) OR periciazine) OR perphenazine) OR pimavanserin) OR pimozide) OR quetiapine) OR risperidone) OR sertindole) OR sulpiride) OR ziprasidone) OR zuclopenthixol) OR FGA) OR first generation antipsychotic) OR second generation antipsychotic) OR SGA))) NOT ((((((((metformin) OR topiramate) OR ramelteon) OR rimonabant) OR modafinil) OR sibutramine) OR steroids) OR ropinirole) AND ((Clinical Trial[ptyp] OR Clinical Trial. Phase III[ptyp] OR Clinical Trial. Phase IV[ptyp] OR Controlled Clinical Trial[ptyp] OR Randomized Controlled Trial[ptyp]) AND Humans[Mesh])**
2. **Filters: Adolescent: 13-18 years. Adult: 19+ years**

## Appendix 2.2: Embase Search Terms

1. (((change or gain or clinically relevant or body) and weight and (amisulpride or amoxapine or aripiprazole or asenapine or blonanserin or brexpiprazole or bromperidol or cariprazine or chlorpromazine or clozapine or flupentixol or fluphenazine or haloperidol or iloperidone or levopromazine or lurasidone or melperone or olanzapine or paliperidone or penfluridol or zotepine or periciazine or perphenazine or pimavanserin or pimozide or quetiapine or risperidone or sertindole or sulpiride or ziprasidone or zuclopenthixol or FGA or first generation antipsychotic or second generation antipsychotic or SGA)) not (metformin or topiramate or ramelteon or rimonabant or modafinil or sibutramine or steroids or ropinirole)).mp. [mp=title. abstract. heading word. drug trade name. original title. device manufacturer. drug manufacturer. device trade name. keyword. floating subheading word. candidate term word]
2. limit 1 to (human and (clinical trial or randomized controlled trial or controlled clinical trial or phase 3 clinical trial or phase 4 clinical trial) and (article or article in press) and (adolescent <13 to 17 years> or adult <18 to 64 years> or aged <65+ years>))

## Appendix 2.3: PsycINFO Search Terms

1. (((((change OR gain OR clinically relevant OR body) AND weight)) AND (((((((((((((((((((((((((((((((((((amisulpride) OR amoxapine) OR aripiprazole) OR asenapine) OR blonanserin) OR brexpiprazole) OR bromperidol) OR cariprazine) OR chlorpromazine) OR clozapine) OR flupentixol) OR fluphenazine) OR haloperidol) OR iloperidone) OR levopromazine) OR lurasidone) OR melperone) OR olanzapine) OR paliperidone) OR penfluridol) OR zotepine) OR periciazine) OR perphenazine) OR pimavanserin) OR pimozide) OR quetiapine) OR risperidone) OR sertindole) OR sulpiride) OR ziprasidone) OR zuclopenthixol) OR FGA) OR first generation antipsychotic) OR second generation antipsychotic) OR SGA))) NOT ((((((((metformin) OR topiramate) OR ramelteon) OR rimonabant) OR modafinil) OR sibutramine) OR steroids) OR ropinirole)
2. Limiters:
   1. Age Groups: Adolescence (13-17 yrs). Adulthood (18 yrs & older)
   2. Population Group: Human
   3. Document Type: Journal Article
   4. Methodology: CLINICAL TRIAL

# Appendix 3: Included studies

## Appendix 3.1: Table of included studies

| Study. year (N=202; n=80.372) | Antipsychotic/ placebo | Diagnosis | AP-naive | Sample size | % Male | Age: mean (SD) | Duration (weeks) | % with CRWG | % with CRWL |
| --- | --- | --- | --- | --- | --- | --- | --- | --- | --- |
| Adams 2013 (1) | LY2140023 | Schizophrenia | _ | 130 | 65.4 | 38.7 (10.9) | 24 | 8.2 | 14.8 |
|  | SGA | Schizophrenia | _ | 131 | 66.4 | 39.5 (12.5) | 24 | 21.4 | 2.3 |
| Adams 2014 (2) | Aripiprazole | Schizophrenia | _ | 161 | 65.8 | 43.0 (11.0) | 24 | 7.1 | 3.2 |
|  | LY2140023 | Schizophrenia | _ | 511 | 63.8 | 42.3 (10.9) | 24 | 4.1 | 13.1 |
| Addington 2004 (3) | Risperidone | Schizophrenia, Schizoaffective | _ | 147 | 71.4 | 33.9(-) | 8 | 16.0 | 2.4 |
|  | Ziprasidone | Schizophrenia, Schizoaffective | _ | 149 | 73.8 | 35.1(-) | 8 | 8.2 | 7.4 |
| Alphs 2015 (4) | Paliperidone IM | Schizophrenia | _ | 226 | 85.4 | 37.7 (10.6) | 38 | 32.4 | _ |
| Alvarez 2012 (5) | Olanzapine | Schizophrenia | _ | 23 | 65.2 | 35.6 (13.5) | 24 | 47.8 | _ |
|  | Ziprasidone | Schizophrenia | _ | 27 | 74.1 | 40.8 (9.2) | 24 | 11.1 | _ |
| Arvanitis 1997 (6) | Haloperidol | Schizophrenia | _ | 52 | 80.8 | 37.0 (10.0) | 6 | 4.0 | _ |
|  | Placebo | Schizophrenia | _ | 51 | 80.4 | 36.0 (8.0) | 6 | 6.0 | _ |
|  | Quetiapine 75 mg | Schizophrenia | _ | 53 | 73.6 | 37.0 (10.0) | 6 | 11.0 | _ |
|  | Quetiapine 150 mg | Schizophrenia | _ | 48 | 81.3 | 38.0 (9.0) | 6 | 17.0 | _ |
|  | Quetiapine 300 mg | Schizophrenia | _ | 52 | 71.2 | 38.0 (9.0) | 6 | 10.0 | _ |
|  | Quetiapine 600 mg | Schizophrenia | _ | 51 | 74.5 | 39.0 (8.0) | 6 | 16.0 | _ |
|  | Quetiapine 750 mg | Schizophrenia | _ | 54 | 70.4 | 35.0 (10.0) | 6 | 13.0 | _ |
| Ascher-Svanum 2005 (7) | Olanzapine | Schizophrenia, Schizoaffective | _ | 1283 | 65.1 | 38.7 (11.6) | 6 | 13.6 | _ |
|  | Haloperidol | Schizophrenia, Schizoaffective | _ | 622 | 64.6 | 38.3 (11.1) | 6 | 3.1 | _ |
| Bauer 2013(8) | Quetiapine | MDD | _ | 228 | - | - | 6 | 7.6 | _ |
| Berwaerts 2012a (9) | Paliperidone 3 mg | Bipolar I | _ | 107 | 51.0 | 39.0 (10.2) | 3 | 5.6 | _ |
|  | Paliperidone 6 mg | Bipolar I | _ | 112 | 53.0 | 39.0 (11.3) | 3 | 5.4 | _ |
|  | Paliperidone 12 mg | Bipolar I | _ | 109 | 55.0 | 39.0 (11) | 3 | 0.9 | _ |
|  | Placebo | Bipolar I | _ | 115 | 55.0 | 41.0 (11.2) | 3 | 0.9 | _ |
| Berwaerts 2012b (10) | Olanzapine | Bipolar I | _ | 82 | 41.0 | 39.0 (11.9) | 62 | 41.0 | _ |
|  | Paliperidone | Bipolar I | _ | 146 | 49.0 | 40.0 (12.7) | 33 | 29.0 | _ |
|  | Placebo | Bipolar I | _ | 144 | 44.0 | 40.0 (12.6) | 22 | 21.0 | _ |
| Berwaerts 2015 (11) | Paliperidone IM | Schizophrenia | _ | 160 | 74.0 | 37.1 (10.9) | 24 | 10.0 | _ |
|  | Placebo | Schizophrenia | _ | 145 | 76.0 | 38.5 (11.2) | 21 | 1.0 | _ |
| Borison 1996 (12) | Placebo | Schizophrenia | _ | 55 | 91.0 | 37.0 (8.0) | 4 | 4.0 | _ |
|  | Quetiapine | Schizophrenia | _ | 54 | 89.0 | 36.0 (9.0) | 4 | 25.0 | _ |
| Bortnick 2011 (13) | Placebo | MDD | _ | 155 | 35.5 | 42.6 (11.7) | 8 | 1.3 | _ |
|  | Quetiapine | MDD | _ | 152 | 35.4 | 43.3 (10.5) | 8 | 2.6 | _ |
| Buchanan 2012 (14) | Asenapine (Study 1) | Schizophrenia | _ | 241 | 68.0 | 40.7 (12.7) | 26 | 7.9 | 11.6 |
|  | Asenapine (Study 2) | Schizophrenia | _ | 244 | 72.1 | 43.1 (11.4) | 26 | 8.0 | 4.2 |
|  | Olanzapine (Study 1) | Schizophrenia | _ | 240 | 68.3 | 40.3 (11.7) | 26 | 24.6 | 3.3 |
|  | Olanzapine (Study 2) | Schizophrenia | _ | 224 | 75.9 | 42.8 (11.3) | 26 | 18.6 | 3.2 |
| Bushe 2010 (15) | Olanzapine | Schizophrenia, Schizoaffective | _ | 171 | 66.7 | 41.7 (9.5) | 24 | 19.2 | 9.6 |
|  | Quetiapine | Schizophrenia, Schizoaffective | _ | 175 | 65.1 | 40.5 (9.6) | 24 | 13.2 | 9.0 |
| Calabrese 2005 (16) | Placebo | Bipolar | _ | 169 | 37.9 | 38.3 (11.1) | 8 | 1.7 | _ |
|  | Quetiapine 600 mg | Bipolar | _ | 170 | 41.8 | 37.3 (11.4) | 8 | 9.0 | _ |
|  | Quetiapine 300 mg | Bipolar | _ | 172 | 45.9 | 36.6 (11.2) | 8 | 8.5 | _ |
| Calabrese 2017 (17) | Aripiprazole | Bipolar I | _ | 133 | 37.6 | 40.6 (10.8) | 52 | 18.0 | 9.4 |
|  | Placebo | Bipolar I | _ | 133 | 47.4 | 40.6 (11.2) | 52 | 12.9 | 12.1 |
| Cantillon 2017 (18) | Aripiprazole | Schizophrenia, Schizoaffective | _ | 20 | 90.0 | 35.0 (10.0) | 4 | 14.0 | _ |
|  | Placebo | Schizophrenia, Schizoaffective | _ | 38 | 71.1 | 36.0 (12.0) | 4 | 11.0 | _ |
|  | RP5063 15 mg | Schizophrenia, Schizoaffective | _ | 58 | 70.7 | 36.0 (10.0) | 4 | 7.0 | _ |
|  | RP5063 30 mg | Schizophrenia, Schizoaffective | _ | 59 | 84.7 | 37.0 (12.0) | 4 | 6.0 | _ |
|  | RP5063 50 mg | Schizophrenia, Schizoaffective | _ | 58 | 72.4 | 35.0 (9.0) | 4 | 7.0 | _ |
| Canuso 2010a (19) | Paliperidone (lower dose) | Schizoaffective | _ | 105 | 66.7 | 38.1 (10.0) | 6 | 2.9 | _ |
|  | Paliperidone (higher dose) | Schizoaffective | _ | 98 | 65.3 | 36.5 (10.5) | 6 | 7.1 | _ |
|  | Placebo | Schizoaffective | _ | 107 | 62.6 | 37.1 (11.1) | 6 | 0.9 | _ |
| Canuso 2010b (20) | Paliperidone ER | Schizoaffective | _ | 214 | 55.9 | 37.6 (9.2) | 6 | 5.6 | _ |
|  | Placebo | Schizoaffective | _ | 95 | 55.9 | 37.6 (9.2) | 6 | 2.1 | _ |
| Casey 2003 (21) | Aripiprazole  (immediate switch) | Schizophrenia, Schizoaffective | _ | 103 | 65.0 | 39.7 (10.3) | 8 | 3.0 | 7.0 |
|  | Aripiprazole  (immediate initiation/ taper off) | Schizophrenia. Schizoaffective | _ | 104 | 69.0 | 37.1 (9.5) | 8 | 5.0 | 15.0 |
|  | Aripiprazole  (titrating dose/ taper off) | Schizophrenia, Schizoaffective | _ | 103 | 73.0 | 39.3 (10.9) | 8 | 3.0 | 8.0 |
| Casey 2008 (22) | Placebo | Schizophrenia | _ | 114 | 77.0 | 40.8 (9.4) | 6 | 5.0 | 3.0 |
|  | Risperidone | Schizophrenia | _ | 116 | 81.0 | 41.1 (8.6) | 6 | 13.0 | 0.0 |
| Chan 2007 (23) | Aripiprazole | Schizophrenia | _ | 49 | 88.0 | 35.2 (10.9) | 4 | 4.0 | _ |
|  | Risperidone | Schizophrenia | _ | 34 | 64.7 | 35.1 (8.6) | 4 | 12.0 | _ |
| Chan 2010 (24) | Olanzapine | Schizophrenia | _ | 35 | 45.7 | 40.8 (11.5) | 8 | 25.7 | _ |
|  | Risperidone | Schizophrenia | _ | 35 | 45.7 | 41.1 (11.3) | 8 | 17.1 | _ |
| Chen 2010 (25) | Placebo | Schizophrenia | _ | 89 | 46.1 | 24.9 (7.3) | 52 | 10.1 | _ |
|  | Quetiapine | Schizophrenia | _ | 89 | 43.8 | 23.5 (5.2) | 52 | 10.1 | _ |
| Chen2012a (26) | Aripiprazole | Schizophrenia, Schizoaffective, Bipolar | _ | 24 | 45.8 | 45.3 (8.7) | 52 | _ | 8.3 |
|  | Ziprasidone | Schizophrenia, Schizoaffective, Bipolar | _ | 28 | 35.7 | 45.2 (9.2) | 52 | _ | 28.6 |
| Chen 2012b (27) | FGA | Schizophrenia | _ | 16 | 81.3 | 36.5 (11.0) | 8 | 0.0 | _ |
|  | Olanzapine | Schizophrenia | _ | 16 | 43.8 | 37.9 (9.4) | 8 | 31.0 | _ |
|  | Risperidone | Schizophrenia | _ | 16 | 50.0 | 36.7 (14.0) | 8 | 19.0 | _ |
| Cheng 2019 (28) | Risperidone | Schizophrenia | yes | 161 | 51.6 | 24.9 (7.1) | 4 | 13.0 | _ |
|  | Olanzapine | Schizophrenia | yes | 165 | 50.6 | 24.6 (7.8) | 4 | 23.6 | _ |
|  | Aripiprazole | Schizophrenia | yes | 161 | 46.0 | 24.8 (7.2) | 4 | 8.7 | _ |
|  | Risperidone | Schizophrenia | yes | 152 | 51.6 | 24.9 (7.1) | 8 | 30.4 | _ |
|  | Olanzapine | Schizophrenia | yes | 149 | 50.6 | 24.6 (7.8) | 8 | 44.2 | _ |
|  | Aripiprazole | Schizophrenia | yes | 153 | 46.0 | 24.8 (7.2) | 8 | 16.2 | _ |
| Chengappa 2010 (29) | Risperidone | Bipolar | _ | 23 | 30.4 | 40.0 (9.2) | 64 | 38.1 | _ |
|  | SGA | Bipolar | _ | 25 | 28.0 | 39.4 (10.9) | 64 | 50.0 | _ |
| Chrzanowski 2006 (30) | Aripiprazole | Schizophrenia | _ | 104 | 57.0 | 41.7 (12.2) | 52 | 10.0 | _ |
|  | Olanzapine | Schizophrenia | _ | 110 | 52.0 | 41.3 (13.6) | 52 | 24.0 | _ |
| Citrome 2012 (31) | Lurasidone | Schizophrenia, Schizoaffective | _ | 419 | 72.1 | 41.7 (11.3) | 52 | 7.3 | 12.7 |
|  | Risperidone | Schizophrenia, Schizoaffective | _ | 202 | 62.4 | 41.6 (11.3) | 52 | 13.7 | 5.6 |
| Conley 2001 (32) | Olanzapine | Schizophrenia, Schizoaffective | _ | 189 | 73.0 | 38.9 (10.5) | 8 | 27.3 | 0.0 |
|  | Risperidone | Schizophrenia, Schizoaffective | _ | 188 | 72.3 | 41.0 (11.0) | 8 | 11.6 | 0.0 |
| Coppola 2011 (33) | Paliperidone XR 1.5 mg | Schizophrenia | _ | 58 | 78.0 | 41.1 (11.6) | 6 | 7.0 | 0.0 |
|  | Paliperidone XR 6 mg | Schizophrenia | _ | 64 | 69.0 | 40.7 (12.2) | 6 | 3.0 | 6.0 |
|  | Placebo | Schizophrenia | _ | 56 | 74.0 | 36.4 (10.7) | 6 | 5.0 | 4.0 |
| Correll 2015 (34) | Brexpiprazole 2 mg | Schizophrenia | _ | 182 | 61.0 | 39.6 (10.2) | 6 | 8.8 | _ |
|  | Brexpiprazole 4 mg | Schizophrenia | _ | 180 | 61.7 | 40.8 (11.0) | 6 | 9.0 | _ |
|  | Placebo | Schizophrenia | _ | 184 | 64.1 | 39.7 (10.8) | 6 | 4.4 | _ |
| Correll 2020 (35) | Lumateperone 42 mg | Schizophrenia | _ | 150 | 73.3 | 42.4 (10.3) | 4 | 8.4 | 2.1 |
|  | Lumateperone 28 mg | Schizophrenia | _ | 150 | 75.3 | 43.5 (10.1) | 4 | 4.3 | 0.7 |
|  | Placebo | Schizophrenia | _ | 149 | 82.6 | 41.4 (10.3) | 4 | 3.8 | 0.0 |
| Correll 2020 (36) | Olanzapine | Schizophrenia | _ | 276 | 75.0 | 40.1 (10.0) | 24 | 42.7 | _ |
| Cutler 2008 (37) | Iloperidone | Schizophrenia | _ | 300 | 83.1 | 39.5 (10.4) | 4 | 21.0 | _ |
|  | Placebo | Schizophrenia | _ | 147 | 76.5 | 40.7 (10.4) | 4 | 3.0 | _ |
|  | Ziprasidone | Schizophrenia | _ | 150 | 75.8 | 40.0 (9.9) | 4 | 7.0 | _ |
| Cutler 2009 (38) | Placebo | MDD | _ | 157 | 35.5 | 42.3 (11.5) | 8 | 0.0 | _ |
|  | Quetiapine XR 150 mg | MDD | _ | 152 | 36.7 | 40.9 (12.3) | 8 | 2.1 | _ |
|  | Quetiapine XR 300 mg | MDD | _ | 152 | 49.0 | 41.6 (12.0) | 8 | 3.4 | _ |
| Cutler 2011a (39) | Placebo | Bipolar I | _ | 160 | 58.5 | 40.8 (10.7) | 3 | 0.0 | _ |
|  | Quetiapine | Bipolar I | _ | 151 | 61.7 | 41.3 (10.3) | 3 | 5.1 | _ |
| Cutler 2011b (40) | Placebo | Schizophrenia | _ | 107 | 69.4 | 42.5 (10.8) | 6 | 5.6 | _ |
|  | Quetiapine XR 400 mg | Schizophrenia | _ | 107 | 69.9 | 42.1 (10.1) | 6 | 6.5 | _ |
|  | Quetiapine XR 600 mg | Schizophrenia | _ | 108 | 81.2 | 41.2 (10.8) | 6 | 14.1 | _ |
|  | Quetiapine XR 800 mg | Schizophrenia | _ | 99 | 74.5 | 40.2 (9.1) | 6 | 6.5 | _ |
|  | Quetiapine IR 800 mg | Schizophrenia | _ | 108 | 63.3 | 40.8 (10.4) | 6 | 12.1 | _ |
| Davidson 2007 (41) | Olanzapine | Schizophrenia | _ | 126 | 76.0 | 36.5 (10.2) | 6 | 22.0 | 2.0 |
|  | Paliperidone XR 3 mg | Schizophrenia | _ | 123 | 63.0 | 36.3 (11.0) | 6 | 7.0 | 2.0 |
|  | Paliperidone XR 9 mg | Schizophrenia | _ | 123 | 65.0 | 37.6 (9.8) | 6 | 11.0 | 2.0 |
|  | Paliperidone XR 15 mg | Schizophrenia | _ | 113 | 64.0 | 37.3 (10.9) | 6 | 18.0 | 2.0 |
|  | Placebo | Schizophrenia | _ | 120 | 68.0 | 36.8 (10.6) | 6 | 6.0 | 12.0 |
| De Deyn 2005 (42) | Aripriprazole | Alzheimer | _ | 106 | 28.0 | 81.5(-) | 10 | 5.0 | _ |
|  | Placebo | Alzheimer | _ | 102 | 28.0 | 81.5(-) | 10 | 3.0 | _ |
| De Hert 2011 (43) | Risperidone | Schizophrenia | _ | 130 | 46.2 | 37.0(-) | 60 | 16.0 | _ |
|  | Sertindole | Schizophrenia | _ | 131 | 48.9 | 35.0(-) | 60 | 17.0 | _ |
| Deberdt 2005 (44) | Olanzapine | Dementia | _ | 204 | 30.9 | 77.9 (7.7) | 10 | 6.2 | _ |
|  | Placebo | Dementia | _ | 94 | 36.2 | 79.8 (7.2) | 10 | 1.1 | _ |
|  | Risperidone | Dementia | _ | 196 | 37.2 | 78.0 (6.9) | 10 | 0.5 | _ |
| Detke 2014 (45) | Olanzapine IM | Schizophrenia | _ | 264 | 66.3 | 41.7 (10.9) | 104 | 40.8 | 20.0 |
|  | Olanzapine oral | Schizophrenia | _ | 260 | 68.1 | 40.1 (10.8) | 104 | 38.0 | 17.3 |
| Dossenbach 2007 (46) | Chlorpromazine | Schizophrenia | _ | 37 | 72.5 | 33.4 (8.5) | 6 | 24.3 | _ |
|  | Olanzapine | Schizophrenia | _ | 80 | 74.7 | 30.9 (7.8) | 6 | 26.3 | _ |
| Dubovsky 2012 (47) | Asenapine (slow dose escalation) | Schizophrenia, Schizoaffective | _ | 61 | 32.8 | 70.5 (4.6) | 6 | 0.0 | _ |
|  | Asenapine (rapid dose escalation) | Schizophrenia, Schizoaffective | _ | 61 | 23.0 | 72.0 (5.8) | 6 | 3.3 | _ |
| Durgam 2014 (48) | Cariprazine 1.5 mg | Schizophrenia | _ | 145 | 64.1 | 36.8 (9.6) | 6 | 9.0 | _ |
|  | Cariprazine 3 mg | Schizophrenia | _ | 146 | 73.3 | 37.1 (10.4) | 6 | 11.0 | _ |
|  | Cariprazine 4.5 mg | Schizophrenia | _ | 147 | 70.1 | 35.8 (10.8) | 6 | 5.0 | _ |
|  | Risperidone | Schizophrenia | _ | 140 | 70.0 | 36.5 (11.1) | 6 | 16.8 | _ |
| Durgam 2015a (49) | Cariprazine | Bipolar I | _ | 118 | 67.8 | 38.0 (10.3) | 3 | 2.5 | _ |
|  | Placebo | Bipolar I | _ | 118 | 65.3 | 38.7 (11.0) | 3 | 0.0 | _ |
| Durgam 2015b (50) | Aripiprazole | Schizophrenia | _ | 152 | 61.8 | 39.3 (10.8) | 6 | 6.0 | _ |
|  | Cariprazine 3 mg | Schizophrenia | _ | 155 | 63.9 | 37.9 (10.6) | 6 | 6.0 | _ |
|  | Cariprazine 6 mg | Schizophrenia | _ | 157 | 63.7 | 38.6 (10.6) | 6 | 5.0 | _ |
|  | Placebo | Schizophrenia | _ | 153 | 63.4 | 38.2 (11.3) | 6 | 3.0 | _ |
| Durgam 2016a (51) | Cariprazine 0.75 mg | Bipolar I | _ | 141 | 35.5 | 40.1 (11.2) | 8 | 2.1 | _ |
|  | Cariprazine 1.5 mg | Bipolar I | _ | 146 | 37.0 | 40.9 (11.4) | 8 | 6.9 | _ |
|  | Cariprazine 3 mg | Bipolar I | _ | 146 | 39.7 | 42.8 (10.8) | 8 | 4.8 | _ |
|  | Placebo | Bipolar I | _ | 145 | 38.6 | 43.6 (12.0) | 8 | 3.5 | _ |
| Durgam 2016b (52) | Cariprazine | Schizophrenia | _ | 101 | 61.4 | 39.2 (10.9) | 37 | 27.0 | _ |
|  | Placebo | Schizophrenia | _ | 99 | 70.7 | 37.7 (10.1) | 29 | 32.2 | _ |
| Durgam 2017 (53) | Asenapine 2.5 mg | Schizophrenia | _ | 31 | 58.1 | 41.1 (9.8) | 26 | 6.5 | _ |
|  | Asenapine 5 mg | Schizophrenia | _ | 42 | 59.5 | 39.5 (10.0) | 26 | 12.2 | _ |
|  | Olanzapine | Schizophrenia | _ | 16 | 62.5 | 37.4 (12.5) | 26 | 26.7 | _ |
| Earley 2019 (54) | Cariprazine 1.5 mg | Bipolar I | _ | 167 | 64.1 | 42.2 (12.0) | 5 | 2.4 | _ |
|  | Cariprazine 3.mg | Bipolar I | _ | 158 | 65.2 | 43.9 (11.8) | 5 | 1.9 | _ |
|  | Placebo | Bipolar I | _ | 165 | 58.8 | 44.6 (11.5) | 6 | 0.0 | _ |
| Fleischhacker 2009 (55) | Aripiprazole | Schizophrenia | _ | 317 | 57.0 | 35.9(-) | 26 | 21.0 | _ |
|  | Aripiprazole | Schizophrenia | _ | 317 | 57.0 | 35.9(-) | 52 | 21.0 | _ |
|  | Olanzapine | Schizophrenia | _ | 313 | 56.0 | 37.3(-) | 26 | 40.0 | _ |
|  | Olanzapine | Schizophrenia | _ | 313 | 56.0 | 37.3(-) | 52 | 43.0 | _ |
| Fleischhacker 2012 (56) | Paliperidone | Schizophrenia | _ | 379 | 57.0 | 40.7 (11.8) | 53 | 14.0 | _ |
|  | Risperidone | Schizophrenia | _ | 368 | 62.0 | 40.6 (12.1) | 53 | 15.0 | _ |
| Fleischhacker 2014 (57) | Aripiprazole IM 400 mg | Schizophrenia | _ | 264 | 60.4 | 41.7 (10.4) | 38 | 15.9 | 15.2 |
|  | Aripiprazole oral 10-30 mg | Schizophrenia | _ | 266 | 63.2 | 41.2 (10.8) | 38 | 16.2 | 10.2 |
|  | Aripiprazole IM 50 mg | Schizophrenia | _ | 131 | 59.5 | 40.2 (9.6) | 38 | 6.1 | 13.7 |
| Fleischhacker 2017 (58) | Brexpiprazole | Schizophrenia | _ | 264 | 59.8 | 38.8 (10.7) | 52 | 5.2 | _ |
|  | Placebo | Schizophrenia | _ | 104 | 61.9 | 41.6 (10.6) | 52 | 1.0 | _ |
| Fu 2015 (59) | Paliperidone | Schizoaffective | _ | 164 | 51.8 | 39.3(-) | 64 | 13.0 | _ |
|  | Placebo | Schizoaffective | _ | 170 | 49.4 | 38.0(-) | 64 | 6.0 | _ |
| Garcia 2009 (60) | Blonanserin 2.5 mg | Schizophrenia | _ | 61 | 54.1 | 38.3 (10.6) | 6 | 3.3 | _ |
|  | Blonanserin 5 mg | Schizophrenia | _ | 58 | 69.0 | 37.0 (11.4) | 6 | 0.0 | _ |
|  | Blonanserin 10 mg | Schizophrenia | _ | 64 | 54.7 | 38.4 (10.8) | 6 | 3.1 | _ |
|  | Haloperidol | Schizophrenia | _ | 60 | 58.3 | 38.1 (11.4) | 6 | 3.3 | _ |
|  | Placebo | Schizophrenia | _ | 64 | 62.5 | 38.6 (11.5) | 6 | 3.1 | _ |
| Gopal 2010 (61) | Paliperidone IM 50 mg eq. | Schizophrenia | _ | 94 | 70.0 | 39.0 (10.6) | 13 | 12.0 | _ |
|  | Paliperidone IM 100 mg eq. | Schizophrenia | _ | 97 | 65.0 | 39.0 (10.7) | 13 | 10.0 | _ |
|  | Paliperidone IM 150 mg eq. | Schizophrenia | _ | 30 | 73.0 | 41.0 (11.1) | 13 | 4.0 | _ |
|  | Placebo | Schizophrenia | _ | 136 | 71.0 | 41.0 (11.0) | 13 | 2.0 | _ |
| Green 2006 (62) | Haloperidol | Schizophrenia, Schizoaffective, Schizofreniform | yes | 132 | 84.1 | 24.0 (4.9) | 104 | 42.0 | _ |
|  | Olanzapine | Schizophrenia, Schizoaffective, Schizofreniform | yes | 131 | 79.4 | 25.5 (4.6) | 104 | 72.0 | _ |
| Grootens 2011 (63) | Olanzapine | Schizophrenia, Schizoaffective | yes | 34 | 86.0 | 23.1 (4.4) | 8 | 64.5 | _ |
|  | Ziprasidone | Schizophrenia, Schizoaffective | yes | 39 | 79.0 | 24.3 (4.5) | 8 | 3.3 | _ |
| Grossberg 2020 (64) | Brexpiprazole 0.5/1 mg fixed dose (Study 1) | Alzheimer | _ | 157 | 43.1 | 73.8 (8.8) | 12 | 1.9 | 3.8 |
|  | Brexpiprazole 2 mg fixed dose(Study 1) | Alzheimer | _ | 140 | 43.6 | 73.7 (8.1) | 12 | 2.1 | 2.9 |
|  | Brexpiprazole 0.5-2 mg flexible dose (Study 2) | Alzheimer | _ | 133 | 38.3 | 73.5 (8.5) | 12 | 1.5 | 0.0 |
|  | Placebo (Study 1) | Alzheimer | _ | 136 | 48.5 | 74.1 (8.0) | 12 | 0.7 | 4.5 |
|  | Placebo (Study 2) | Alzheimer | _ | 137 | 35.8 | 74.0 (7.8) | 12 | 1.5 | 2.9 |
| Higuchi 2019a (65) | Lurasidone 40 mg | Schizophrenia | _ | 150 | 54.7 | 42.1 (13.0) | 6 | 5.3 | _ |
|  | Lurasidone 80 mg | Schizophrenia | _ | 154 | 52.6 | 43.6 (13.8) | 6 | 1.3 | _ |
|  | Placebo | Schizophrenia | _ | 151 | 58.9 | 42.6 (13.4) | 6 | 1.4 | _ |
| Higuchi 2019b (66) | Lurasidone 40 mg | Schizophrenia | _ | 125 | 59.1 | 45.6(-) | 6 | 2.4 | _ |
|  | Lurasidone 80 mg | Schizophrenia | _ | 129 | 59.1 | 45.6(-) | 6 | 1.5 | _ |
|  | Placebo | Schizophrenia | _ | 129 | 59.1 | 45.6(-) | 6 | 2.3 | _ |
|  | Risperidone | Schizophrenia | _ | 64 | 59.1 | 45.6(-) | 6 | 6.2 | _ |
| Hill 2011 (67) | Olanzapine IM 300 mg | Schizophrenia | _ | 140 | 60.0 | 37.7 (10.5) | 24 | 12.1 | 6.4 |
|  | Olanzapine IM 405 mg | Schizophrenia | _ | 318 | 66.7 | 39.0 (11.3) | 24 | 10.8 | 4.1 |
|  | Olanzapine IM 600 mg | Schizophrenia | _ | 141 | 67.4 | 39.5 (11.2) | 24 | 17.1 | 5.0 |
| Hobart 2018a (68) | Brexpiprazole | MDD | _ | 197 | 35.0 | 43.6 (11.5) | 6 | 5.7 | _ |
|  | Placebo | MDD | _ | 206 | 17.7 | 41.8 (11.7) | 6 | 2.4 | _ |
|  | Quetiapine | MDD | _ | 100 | 34.0 | 44.6 (11.6) | 6 | 5.1 | _ |
| Hobart 2018b (69) | Brexpiprazole | MDD | _ | 192 | 23.4 | 43.0 (12.7) | 6 | 4.2 | _ |
|  | Placebo | MDD | _ | 202 | 28.7 | 42.7 (12.5) | 6 | 1.0 | _ |
| Honer 2012 (70) | Quetiapine 800 mg | Schizophrenia, Schizoaffective | _ | 43 | 74.4 | 37.9 (10.9) | 8 | 2.3 | _ |
|  | Quetiapine > 800 mg | Schizophrenia, Schizoaffective | _ | 88 | 65.9 | 40.6 (12.5) | 8 | 10.2 | _ |
| Hough 2010 (71) | Paliperidone | Schizophrenia | _ | 205 | 53.2 | 38.8 (11.4) | 24 | 6.0 | _ |
|  | Placebo | Schizophrenia | _ | 203 | 54.7 | 39.4 (10.8) | 15 | 3.0 | _ |
| Ishigooka 2018 (72) | Brexpiprazole 1 mg | Schizophrenia | _ | 115 | 44.3 | 44.7 (11.5) | 6 | 4.3 | _ |
|  | Brexpiprazole 2 mg | Schizophrenia | _ | 115 | 53.0 | 43.3 (12.0) | 6 | 5.3 | _ |
|  | Brexpiprazole 4 mg | Schizophrenia | _ | 113 | 48.7 | 44.1 (11.9) | 6 | 2.7 | _ |
|  | Placebo | Schizophrenia | _ | 116 | 44.0 | 45.0 (11.9) | 6 | 0.9 | _ |
| Iyo 2021 (73) | Lurasidone | Schizophrenia | _ | 247 | 48.6 | 41.0 (11.0) | 6 | 3.3 | _ |
|  | Placebo | Schizophrenia | _ | 235 | 51.5 | 39.3 (11.4) | 6 | 0.9 | _ |
| Jeste 2003 (74) | Olanzapine | Schizophrenia | _ | 88 | 31.8 | 71.4 (5.6) | 8 | 14.8 | _ |
|  | Risperidone | Schizophrenia | _ | 87 | 39.1 | 70.9 (5.6) | 8 | 5.1 | _ |
| Jindal 2013 (75) | Aripiprazole | Schizophrenia | _ | 30 | 63.3 | - | 6 | 7.7 | _ |
|  | Olanzapine | Schizophrenia | _ | 30 | 50.0 | - | 6 | 22.2 | _ |
| Kamijima 2013 (76) | Aripiprazole 3mg fixed dose | MDD | _ | 197 | 62.9 | 39.2 (9.1) | 6 | 8.1 | _ |
|  | Aripiprazole 3-15 mg flexible dose | MDD | _ | 194 | 52.1 | 38.1 (9.6) | 6 | 10.4 | _ |
|  | Placebo | MDD | _ | 195 | 59.0 | 38.7 (9.2) | 6 | 1.6 | _ |
| Kanba 2014 (77) | Aripiprazole | Bipolar I | _ | 122 | 42.6 | 37.5 (12.5) | 3 | 0.8 | 5.0 |
|  | Placebo | Bipolar I | _ | 125 | 40.0 | 37.8 (12.7) | 3 | 0.0 | 6.6 |
| Kane 2002 (78) | Aripiprazole 15 mg | Schizophrenia, Schizoaffective | _ | 102 | 74.5 | 37.8(-) | 4 | 7.0 | _ |
|  | Aripiprazole 30 mg | Schizophrenia, Schizoaffective | _ | 102 | 68.6 | 39.3(-) | 4 | 4.0 | _ |
|  | Haloperidol | Schizophrenia, Schizoaffective | _ | 104 | 65.4 | 38.9(-) | 4 | 10.0 | _ |
|  | Placebo | Schizophrenia, Schizoaffective | _ | 106 | 69.8 | 38.5(-) | 4 | 1.0 | _ |
| Kane 2006 (79) | Chlorpromazine | Schizophrenia | _ | 154 | 79.0 | 34.4 (8.2) | 6 | 13.8 | 1.8 |
|  | Ziprasidone | Schizophrenia | _ | 152 | 67.0 | 35.6 (9.5) | 6 | 5.1 | 10.2 |
| Kane 2007 (80) | Olanzapine | Schizophrenia | _ | 128 | 47.0 | 36.3 (11.2) | 6 | 13.0 | 1.0 |
|  | Paliperidone ER 6 mg | Schizophrenia | _ | 123 | 50.0 | 37.0 (10.2) | 6 | 5.0 | 2.0 |
|  | Paliperidone ER 9 mg | Schizophrenia | _ | 122 | 59.0 | 38.5 (11.4) | 6 | 7.0 | 3.0 |
|  | Paliperidone ER 12 mg | Schizophrenia | _ | 129 | 53.0 | 36.0 (10.6) | 6 | 3.0 | 2.0 |
|  | Placebo | Schizophrenia | _ | 126 | 52.0 | 37.9 (10.9) | 6 | 2.0 | 5.0 |
| Kane 2009a (81) | Aripiprazole | Schizophrenia | _ | 861 | 59.3 | 37.3 (11.0) | 52 | 25.4 | _ |
|  | Haloperidol | Schizophrenia | _ | 433 | 57.0 | 36.8 (10.8) | 52 | 16.6 | _ |
| Kane 2009b (82) | Aripiprazole | Schizophrenia | _ | 285 | 66.7 | 37.3 (10.4) | 28 | 16.4 | _ |
|  | Olanzapine | Schizophrenia | _ | 281 | 69.0 | 38.3 (10.5) | 28 | 40.3 | _ |
| Kane 2010 (83) | Asenapine 5 mg | Schizophrenia | _ | 114 | - | - | 6 | 5.0 | 1.0 |
|  | Asenapine 10 mg | Schizophrenia | _ | 106 | - | - | 6 | 4.0 | 2.0 |
|  | Haloperidol | Schizophrenia | _ | 115 | - | - | 6 | 4.0 | 0.0 |
|  | Placebo | Schizophrenia | _ | 123 | - | - | 6 | 2.0 | 2.0 |
| Kane 2011a (84) | Asenapine | Schizophrenia | _ | 194 | 54.1 | 39.2 (12.5) | 26 | 3.7 | 3.2 |
|  | Placebo | Schizophrenia | _ | 192 | 60.4 | 38.7 (11.6) | 26 | 0.5 | 9.6 |
| Kane 2011b (85) | Risperidone | Schizophrenia | _ | 105 | 78.0 | 38.7 (7.3) | 12 | 16.0 | _ |
|  | Sertindole | Schizophrenia | _ | 205 | 78.0 | 38.9 (9.0) | 12 | 25.0 | _ |
| Kane 2012 (86) | Aripiprazole | Schizophrenia | _ | 269 | 60.2 | 40.1 (11.0) | 52 | 6.4 | _ |
|  | Placebo | Schizophrenia | _ | 134 | 59.0 | 41.7 (10.5) | 52 | 5.2 | _ |
| Kane 2014 (87) | Aripiprazole | Schizophrenia | _ | 168 | 77.4 | 42.1 (11.0) | 12 | 21.5 | _ |
|  | Placebo | Schizophrenia | _ | 172 | 80.8 | 42.7 (10.9) | 12 | 8.5 | _ |
| Kane 2015a (88) | Cariprazine 3-6 mg | Schizophrenia | _ | 151 | 78.1 | 36.6 (10.5) | 6 | 8.0 | _ |
|  | Cariprazine 6-9 mg | Schizophrenia | _ | 148 | 76.4 | 35.5 (9.3) | 6 | 11.0 | _ |
|  | Placebo | Schizophrenia | _ | 147 | 74.8 | 36.7 (11.3) | 6 | 4.0 | _ |
| Kane 2015b (89) | Brexpiprazole 1 mg | Schizophrenia | _ | 120 | 64.2 | 39.1 (11.9) | 6 | 10.0 | _ |
|  | Brexpiprazole 2 mg | Schizophrenia | _ | 186 | 65.6 | 36.9 (10.9) | 6 | 12.2 | _ |
|  | Brexpiprazole 4 mg | Schizophrenia | _ | 184 | 61.4 | 38.6 (11.0) | 6 | 11.4 | _ |
|  | Placebo | Schizophrenia | _ | 184 | 60.3 | 39.3 (10.8) | 6 | 3.9 | _ |
| Karagianis 2009 (90) | Olanzapine oral | Schizophrenia, Schizoaffective, other psychotic disorder, Bipolar | _ | 65 | 50.8 | 39.0 (12.0) | 16 | 11.1 | _ |
|  | Olanzapine ODT | Schizophrenia, Schizoaffective, other psychotic disorder, Bipolar | _ | 84 | 57.1 | 39.0 (13.0) | 16 | 14.6 | _ |
| Katagiri 2012 (91) | Haloperidol | Bipolar I | _ | 20 | 55.0 | 49.3 (11.9) | 6 | 5.3 | _ |
|  | Olanzapine | Bipolar I | _ | 104 | 47.1 | 43.1 (12.0) | 6 | 13.3 | _ |
|  | Olanzapine | Bipolar I | _ | 104 | 47.1 | 43.1 (12.0) | 3 | 6.7 | _ |
|  | Placebo | Bipolar I | _ | 97 | 41.2 | 42.5 (10.9) | 3 | 1.0 | _ |
| Katila 2013 (92) | Placebo | MDD | _ | 172 | 51.0 | 71.2 (4.9) | 9 | 0.6 | _ |
|  | Quetiapine | MDD | _ | 166 | 49.0 | 71.3 (4.6) | 9 | 0.0 | _ |
| Kato 2020 (93) | Lurasidone 20-60 mg | Bipolar I | _ | 184 | 47.8 | 42.6 (12.9) | 6 | 1.1 | 1.1 |
|  | Lurasidone 80-120 mg | Bipolar I | _ | 169 | 47.9 | 43.2 (12.8) | 6 | 1.2 | 0.6 |
|  | Placebo | Bipolar I | _ | 172 | 45.0 | 41.3 (12.6) | 6 | 0.0 | 0.0 |
| Keck 2003 (94) | Aripiprazole | Bipolar I | _ | 130 | 45.4 | 40.5 (12.7) | 3 | 1.6 | _ |
|  | Placebo | Bipolar I | _ | 132 | 41.7 | 40.5 (11.8) | 3 | 0.0 | _ |
| Keck 2006 (95) | Aripiprazole | Bipolar I | _ | 78 | 38.0 | 39.0 (7.9) | 26 | 13.0 | _ |
|  | Placebo | Bipolar I | _ | 83 | 28.0 | 40.3 (10.9) | 26 | 0.0 | _ |
| Keck 2007 (96) | Aripiprazole | Bipolar I | _ | 78 | 38.0 | 39.0 (7.9) | 100 | 20.0 | _ |
|  | Placebo | Bipolar I | _ | 83 | 28.0 | 40.3 (10.9) | 100 | 5.0 | _ |
| Keks 2007 (97) | Olanzapine | Schizophrenia, Schizoaffective | _ | 300 | 58.0 | 35.2 (11.7) | 41 | 36.0 | 6.0 |
|  | Risperidone | Schizophrenia, Schizoaffective | _ | 247 | 56.0 | 35.1 (12.1) | 39 | 20.0 | 6.0 |
| Kerwin 2007 (98) | Aripiprazole | Schizophrenia | _ | 282 | 59.5 | 38.1 (10.8) | 26 | 7.3 | 15.8 |
|  | SGA | Schizophrenia | _ | 266 | 60.1 | 38.3 (11.1) | 26 | 21.2 | 9.1 |
| Ketter 2017 (99) | Asenapine 10 mg | Bipolar I | _ | 51 | 43.1 | 44.6 (10.7) | 26 | 16.3 | _ |
|  | Asenapine 5 mg | Bipolar I | _ | 53 | 47.2 | 44.8 (9.6) | 26 | 13.7 | _ |
| Kim 2007 (100) | Amisulpride | Schizophrenia | _ | 42 | 52.4 | 33.1 (8.7) | 12 | 9.5 | 9.5 |
|  | Risperidone | Schizophrenia | _ | 45 | 57.8 | 37.9 (8.4) | 12 | 2.2 | 4.4 |
| Kim 2012 (101) | Paliperidone ER | Schizophrenia | _ | 32 | 65.6 | 35.4 (8.3) | 12 | 12.5 | _ |
|  | Risperidone | Schizophrenia | _ | 26 | 65.4 | 32.5 (8.2) | 12 | 0.0 | _ |
| Kinon 2006 (102) | Ziprasidone | Schizophrenia, Schizoaffective | _ | 192 | - | - | 24 | 2.7 | _ |
|  | Olanzapine | Schizophrenia, Schizoaffective | _ | 202 | - | - | 24 | 15.5 | _ |
| Kinon 2008 (103) | Olanzapine 10 mg | Schizophrenia, Schizoaffective | _ | 199 | 67.3 | 41.2 (10.7) | 8 | 14.1 | _ |
|  | Olanzapine 20 mg | Schizophrenia, Schizoaffective | _ | 200 | 67.5 | 40.9 (10.8) | 8 | 18.6 | _ |
|  | Olanzapine 40 mg | Schizophrenia, Schizoaffective | _ | 200 | 69.5 | 41.6 (10.6) | 8 | 20.5 | _ |
| Kinon 2011 (104) | Olanzapine | Schizophrenia | _ | 62 | 54.8 | 41.7 (12.3) | 4 | 10.0 | _ |
|  | Placebo | Schizophrenia | _ | 122 | 57.4 | 38.9 (11.3) | 4 | 0.8 | _ |
| Kinoshita 2016 (105) | Asenapine 10 mg | Schizophrenia | _ | 175 | 42.9 | 41.4 (11.0) | 6 | 4.7 | _ |
|  | Asenapine 20 mg | Schizophrenia | _ | 181 | 54.7 | 41.7 (11.1) | 6 | 7.3 | _ |
|  | Placebo | Schizophrenia | _ | 174 | 46.6 | 41.1 (12.3) | 6 | 0.0 | _ |
| Kishi 2016(106) | Aripiprazole | Schizophrenia | yes | 22 | 31.8 | 42.4 (12.6) | 24 | 9.1 | _ |
|  | Blonanserin | Schizophrenia | yes | 22 | 50.0 | 36.5 (9.7) | 24 | 18.2 | _ |
| Krakowski 2009 (107) | Clozapine | Schizophrenia | _ | 34 | 85.3 | 35.2 (11.8) | 12 | 20.6 | _ |
|  | Haloperidol | Schizophrenia | _ | 28 | 85.7 | 31.3 (9.7) | 12 | 0.0 | _ |
|  | Olanzapine | Schizophrenia | _ | 31 | 77.4 | 35.4 (9.9) | 12 | 30.0 | _ |
| Kramer 2007 (108) | Paliperidone XR | Schizophrenia | _ | 104 | 56.0 | 39.0 (10.7) | 24 | 20.0 | _ |
|  | Placebo | Schizophrenia | _ | 101 | 62.0 | 37.5 (10.4) | 24 | 12.0 | _ |
| Kramer 2010 (109) | Paliperidone IM 50 mg eq. | Schizophrenia | _ | 79 | 65.0 | 40.0 (9.8) | 9 | 8.0 | 3.0 |
|  | Paliperidone IM 100 mg eq. | Schizophrenia | _ | 84 | 62.0 | 37.0 (10.4) | 9 | 6.0 | 1.0 |
|  | Placebo | Schizophrenia | _ | 84 | 59.0 | 40.0 (10.5) | 9 | 4.0 | 4.0 |
| Landbloom 2016 (110) | Asenapine 10 mg | Bipolar I | _ | 122 | 46.7 | 44.3 (10.8) | 3 | 6.4 | _ |
|  | Asenapine 20 mg | Bipolar I | _ | 119 | 46.2 | 42.5 (11.1) | 3 | 1.0 | _ |
|  | Placebo | Bipolar I | _ | 126 | 42.9 | 44.6 (11.5) | 3 | 0.0 | _ |
| Landbloom 2017 (111) | Asenapine 5 mg | Schizophrenia | _ | 97 | 59.8 | 41.5 (10.8) | 6 | 4.3 | _ |
|  | Asenapine 10 mg | Schizophrenia | _ | 113 | 61.1 | 39.1 (10.7) | 6 | 8.5 | _ |
|  | Olanzapine | Schizophrenia | _ | 46 | 60.9 | 40.8 (11.2) | 6 | 13.0 | _ |
|  | Placebo | Schizophrenia | _ | 101 | 53.5 | 41.4 (12.1) | 6 | 4.1 | _ |
| Langosch 2008 (112) | Quetiapine | Bipolar I II | _ | 21 | 29.0 | 45.4 (11.0) | 52 | 42.9 | 7.1 |
| Lauriello 2008 (113) | Olanzapine IM 420 mg | Schizophrenia | _ | 106 | 74.5 | 39.8 (10.8) | 8 | 23.6 | _ |
|  | Olanzapine IM 600 mg | Schizophrenia | _ | 100 | 72.0 | 41.5 (11.1) | 8 | 35.4 | _ |
|  | Olanzapine IM 405 mg | Schizophrenia | _ | 100 | 73.0 | 39.5 (11.4) | 8 | 27.0 | _ |
|  | Placebo | Schizophrenia | _ | 98 | 62.2 | 42.6 (11.2) | 8 | 12.4 | _ |
| Li 2014 (114) | Aripiprazole | Schizophrenia | _ | 139 | 48.9 | 33.6 (10.8) | 6 | 3.0 | _ |
|  | Risperidone | Schizophrenia | _ | 140 | 55.0 | 31.3 (10.6) | 6 | 12.0 | _ |
| Li 2015 (115) | Blonanserin | Schizophrenia | _ | 128 | 45.3 | 33.6 (11.0) | 4 | 13.8 | _ |
|  | Risperidone | Schizophrenia | _ | 133 | 56.4 | 35.0 (10.8) | 4 | 21.7 | _ |
| Li 2016 (116) | Placebo | Bipolar I II | _ | 147 | 47.9 | 32.8 (11.0) | 6 | 2.7 | _ |
|  | Quetiapine XR | Bipolar I II | _ | 147 | 48.2 | 33.4 (11.9) | 7 | 8.8 | _ |
| Lieberman 2003 (117) | Haloperidol | Schizophrenia, Schizoaffective, Schizofreniform | yes | 132 | 84.1 | 24.0 (4.9) | 12 | 22.7 | _ |
|  | Olanzapine | Schizophrenia, Schizoaffective, Schizofreniform | yes | 131 | 79.4 | 23.5 (4.6) | 12 | 61.5 | _ |
| Lieberman 2005 (118) | Olanzapine | Schizophrenia | _ | 336 | 72.6 | 40.8 (10.8) | 78 | 30.0 | _ |
|  | Perphenazine | Schizophrenia | _ | 261 | 76.2 | 40.0 (11.1) | 78 | 11.9 | _ |
|  | Quetiapine | Schizophrenia | _ | 337 | 75.7 | 40.9 (11.2) | 78 | 16.1 | _ |
|  | Risperidone | Schizophrenia | _ | 341 | 74.2 | 40.6 (11.3) | 78 | 14.0 | _ |
|  | Ziprasidone | Schizophrenia | _ | 185 | 69.7 | 40.1 (11.0) | 78 | 7.5 | _ |
| Loebel 2013 (119) | Lurasidone 80 mg | Schizophrenia | _ | 125 | 76.8 | 36.2 (10.9) | 6 | 4.3 | _ |
|  | Lurasidone 160 mg | Schizophrenia | _ | 121 | 67.8 | 37.9 (11.3) | 6 | 4.4 | _ |
|  | Placebo | Schizophrenia | _ | 121 | 63.6 | 37.4 (10.8) | 6 | 2.6 | _ |
|  | Quetiapine | Schizophrenia | _ | 119 | 64.7 | 37.4 (10.4) | 6 | 15.3 | _ |
| Loebel 2014 (120) | Lurasidone 20-60 mg | Bipolar I | _ | 161 | 43.5 | 41.3 (12.3) | 6 | 4.2 | _ |
|  | Lurasidone 80-120 mg | Bipolar I | _ | 162 | 39.5 | 42.0 (12.4) | 6 | 0.7 | _ |
|  | Placebo | Bipolar I | _ | 162 | 46.3 | 41.2 (12.5) | 6 | 0.7 | _ |
| MacFadden 2009 (121) | Placebo | Bipolar I | _ | 59 | 72.9 | 37.6 (12.0) | 52 | 31.0 | _ |
|  | Risperidone | Bipolar I | _ | 65 | 70.8 | 40.0 (11.8) | 52 | 28.1 | _ |
| Marder 2007 (122) | Olanzapine | Schizophrenia | _ | 105 | 80.0 | 40.5 (11.0) | 6 | 21.0 | _ |
|  | Paliperidone ER 12 mg | Schizophrenia | _ | 111 | 69.0 | 41.4 (10.7) | 6 | 18.0 | _ |
|  | Paliperidone ER 6 mg | Schizophrenia | _ | 111 | 68.0 | 42.1 (10.2) | 6 | 7.0 | _ |
|  | Placebo | Schizophrenia | _ | 105 | 78.0 | 42.3 (10.7) | 6 | 6.0 | _ |
| Martin 2002 (123) | Amisulpride | Schizophrenia | _ | 189 | 65.6 | 38.2 (12.5) | 7 | 14.3 | _ |
|  | Olanzapine | Schizophrenia | _ | 188 | 64.4 | 37.4 (11.6) | 7 | 25.5 | _ |
| McDonnell 2011 (124) | Olanzapine oral | Schizophrenia | _ | 322 | 65.1 | 38.9 (11.3) | 24 | 21.4 | _ |
|  | Olanzapine IM | Schizophrenia | _ | 599 | 65.1 | 38.9 (11.3) | 24 | 16.8 | _ |
| McElroy 2010 (125) | Placebo | Bipolar I II | _ | 124 | 33.1 | 38.7(-) | 8 | 4.1 | _ |
|  | Quetiapine 300 mg | Bipolar I II | _ | 243 | 38.4 | 38.4(-) | 8 | 9.0 | _ |
|  | Quetiapine 600 mg | Bipolar I II | _ | 244 | 39.2 | 38.5(-) | 8 | 11.3 | _ |
| McEvoy 2006 (126) | Clozapine | Schizophrenia | _ | 49 | 82.0 | 39.4 (9.9) | 24 | 20.0 | _ |
|  | Olanzapine | Schizophrenia | _ | 19 | 95.0 | 44.3 (10.5) | 24 | 13.0 | _ |
|  | Quetiapine | Schizophrenia | _ | 15 | 80.0 | 37.1 (11.8) | 24 | 15.0 | _ |
|  | Risperidone | Schizophrenia | _ | 16 | 63.0 | 37.7 (9.3) | 24 | 18.0 | _ |
| McEvoy 2007 (127) | Aripiprazole 10 mg | Schizophrenia | _ | 105 | 77.4 | 40.0 (11.3) | 6 | 9.0 | _ |
|  | Aripiprazole 15 mg | Schizophrenia | _ | 105 | 74.5 | 40.0 (11.3) | 6 | 10.0 | _ |
|  | Aripiprazole 20 mg | Schizophrenia | _ | 98 | 82.0 | 40.4 (11.0) | 6 | 5.0 | _ |
|  | Placebo | Schizophrenia | _ | 107 | 76.9 | 41.2 (11.4) | 6 | 4.0 | _ |
| McIntyre 2005 (128) | Haloperidol | Bipolar I | _ | 98 | 36.7 | 45.1(-) | 12 | 5.1 | _ |
|  | Placebo | Bipolar I | _ | 100 | 37.0 | 40.6(-) | 12 | 4.0 | _ |
|  | Quetiapine | Bipolar I | _ | 101 | 36.6 | 42.8(-) | 12 | 12.8 | _ |
| McIntyre 2009 (129) | Asenapine | Bipolar I | _ | 194 | 58.8 | 38.7 (11.9) | 3 | 6.0 | 1.8 |
|  | Olanzapine | Bipolar I | _ | 190 | 60.0 | 40.1 (11.3) | 3 | 12.9 | 1.2 |
|  | Placebo | Bipolar I | _ | 104 | 50.0 | 39.4 (12.0) | 3 | 0.0 | 2.3 |
| McIntyre 2010a (130) | Asenapine | Bipolar I | _ | 185 | 49.7 | 39.1 (12.3) | 3 | 7.2 | 1.8 |
|  | Olanzapine | Bipolar I | _ | 205 | 57.1 | 38.4 (10.8) | 3 | 19.0 | 0.0 |
|  | Placebo | Bipolar I | _ | 98 | 49.0 | 38.1 (12.5) | 3 | 1.2 | 1.2 |
| McIntyre 2010b (131) | Asenapine | Bipolar I | _ | 79 | 45.6 | 37.8 (13.3) | 40 | 39.2 | _ |
|  | Olanzapine | Bipolar I | _ | 107 | 63.9 | 38.7 (12.4) | 40 | 55.1 | _ |
| McQuade 2004 (132) | Aripiprazole | Schizophrenia | _ | 156 | 73.1 | 38.6 (10.6) | 26 | 13.0 | _ |
|  | Olanzapine | Schizophrenia | _ | 161 | 71.4 | 38.2 (11.0) | 26 | 33.0 | _ |
| Meltzer 2010 (133) | Clozapine | Schizophrenia | _ | 40 | 67.5 | 25.8 (5.8) | 6 | 28.0 | _ |
|  | Clozapine | Schizophrenia | _ | 40 | 67.5 | 25.8 (5.8) | 24 | 79.4 | _ |
|  | Clozapine | Schizophrenia | _ | 40 | 67.5 | 25.8 (5.8) | 48 | 72.7 | _ |
|  | Clozapine | Schizophrenia | _ | 40 | 67.5 | 25.8 (5.8) | 96 | 80.7 | _ |
|  | FGA | Schizophrenia | _ | 45 | 80.0 | 24.4 (5.0) | 6 | 6.7 | _ |
|  | FGA | Schizophrenia | _ | 45 | 80.0 | 24.4 (5.0) | 24 | 23.3 | _ |
|  | FGA | Schizophrenia | _ | 45 | 80.0 | 24.4 (5.0) | 48 | 41.9 | _ |
|  | FGA | Schizophrenia | _ | 45 | 80.0 | 24.4 (5.0) | 96 | 50.0 | _ |
| Meltzer 2011 (134) | Lurasidone 40 mg | Schizophrenia | _ | 119 | 78.2 | 37.7 (11.0) | 6 | 7.6 | _ |
|  | Lurasidone 120 mg | Schizophrenia | _ | 118 | 78.8 | 37.9 (11.2) | 6 | 4.2 | _ |
|  | Olanzapine | Schizophrenia | _ | 122 | 77.9 | 38.3 (10.2) | 6 | 34.4 | _ |
|  | Placebo | Schizophrenia | _ | 114 | 77.2 | 37.0 (11.3) | 6 | 7.0 | _ |
| Meltzer 2014 (135) | Risperidone IM 100 mg | Schizophrenia | _ | 78 | 71.8 | 41.0 (11.4) | 6 | 4.6 | _ |
|  | Risperidone IM 50 mg | Schizophrenia | _ | 82 | 73.2 | 39.2 (10.6) | 6 | 2.8 | _ |
|  | Risperidone IM 100 mg | Schizophrenia | _ | 78 | 71.8 | 41.0 (11.4) | 12 | 8.3 | _ |
|  | Risperidone IM 50 mg | Schizophrenia | _ | 82 | 73.2 | 39.2 (10.6) | 12 | 12.5 | _ |
|  | Risperidone IM 100 mg | Schizophrenia | _ | 78 | 71.8 | 41.0 (11.4) | 18 | 14.3 | _ |
|  | Risperidone IM 50 mg | Schizophrenia | _ | 82 | 73.2 | 39.2 (10.6) | 18 | 19.7 | _ |
|  | Risperidone IM 100 mg | Schizophrenia | _ | 78 | 71.8 | 41.0 (11.4) | 24 | 20.5 | _ |
|  | Risperidone IM 50 mg | Schizophrenia | _ | 82 | 73.2 | 39.2 (10.6) | 24 | 24.6 | _ |
| Merideth 2012 (136) | Placebo | GAD | _ | 214 | 36.0 | 36.6 (12.3) | 8 | 0.9 | _ |
|  | Quetiapine 150 mg | GAD | _ | 217 | 33.0 | 38.2 (11.5) | 8 | 3.8 | _ |
|  | Quetiapine 300 mg | GAD | _ | 206 | 29.0 | 39.0 (12.6) | 8 | 3.9 | _ |
| Mintzer 2007 (137) | Aripiprazole 2 mg | Alzheimer | _ | 118 | 19.0 | 83.0(-) | 10 | 7.0 | 10.0 |
|  | Aripiprazole 5 mg | Alzheimer | _ | 122 | 24.0 | 82.4(-) | 10 | 4.0 | 13.0 |
|  | Aripiprazole 10 mg | Alzheimer | _ | 126 | 24.0 | 82.3(-) | 10 | 4.0 | 11.0 |
|  | Placebo | Alzheimer | _ | 121 | 18.0 | 82.2(-) | 10 | 6.0 | 15.0 |
| Mitchell 2006 (138) | Olanzapine 20 mg | Schizophrenia, Schizoaffective, Bipolar I | _ | 12 | 75.0 | 40.6 (8.6) | 4 | 25.0 | _ |
|  | Olanzapine 30 mg | Schizophrenia, Schizoaffective, Bipolar I | _ | 11 | 54.5 | 37.9 (8.6) | 4 | 18.2 | _ |
|  | Olanzapine 40 mg | Schizophrenia, Schizoaffective, Bipolar I | _ | 14 | 78.6 | 39.4 (9.2) | 4 | 0.0 | _ |
| Moeller 2008 (139) | Quetiapine XR | Schizophrenia | _ | 331 | 50.9 | 39.8 (11.4) | 6 | 1.5 | _ |
|  | Quetiapine IR | Schizophrenia | _ | 166 | 57.8 | 39.9 (10.2) | 6 | 2.4 | _ |
| Mortimer 2004 (140) | Amisulpride | Schizophrenia | _ | 189 | 65.6 | 38.2 (12.5) | 24 | 20.6 | _ |
|  | Olanzapine | Schizophrenia | _ | 188 | 64.4 | 37.4 (11.6) | 24 | 35.1 | _ |
| Naber 2005 (141) | Clozapine | Schizophrenia | _ | 57 | 61.0 | 35.2 (10.8) | 14 | 52.0 | _ |
|  | Olanzapine | Schizophrenia | _ | 57 | 60.0 | 32.9 (10.4) | 16 | 34.0 | _ |
| Naber 2015(142) | Aripiprazole | Schizophrenia | _ | 144 | 59.7 | 42.6 (10.9) | 28 | 11.1 | 9.7 |
|  | Paliperidone | Schizophrenia | _ | 137 | 59.9 | 41.2 (10.7) | 28 | 14.6 | 5.8 |
| Nakamura 2009 (143) | Lurasidone | Schizophrenia | _ | 90 | 75.6 | 39.7 (9.9) | 6 | 6.7 | _ |
|  | Placebo | Schizophrenia | _ | 90 | 77.8 | 41.9 (9.8) | 6 | 7.8 | _ |
| Nasrallah 2013 (144) | Lurasidone 40 mg | Schizophrenia | _ | 122 | 67.2 | 40.3 (11.3) | 6 | 9.0 | 2.5 |
|  | Lurasidone 80 mg | Schizophrenia | _ | 119 | 63.9 | 38.6 (9.6) | 6 | 9.3 | 1.7 |
|  | Lurasidone 120 mg | Schizophrenia | _ | 124 | 74.2 | 37.6 (11.1) | 6 | 6.5 | 2.4 |
|  | Placebo | Schizophrenia | _ | 124 | 72.6 | 38.2 (9.9) | 6 | 3.2 | 0.8 |
| Nasrallah 2016 (145) | Aripiprazole IM 441 mg | Schizophrenia | _ | 207 | 68.1 | 39.9 (10.1) | 12 | 9.7 | 2.9 |
|  | Aripiprazole IM 882 mg | Schizophrenia | _ | 208 | 68.8 | 39.7 (11.1) | 12 | 8.7 | 3.4 |
|  | Placebo | Schizophrenia | _ | 208 | 66.8 | 39.5 (11.9) | 12 | 5.8 | 5.8 |
| Nasser 2016 (146) | Placebo | Schizophrenia | _ | 112 | 72.3 | - | 8 | 18.0 | _ |
|  | Risperidone IM 90 mg | Schizophrenia | _ | 111 | 83.8 | - | 8 | 32.7 | _ |
|  | Risperidone IM 120 mg | Schizophrenia | _ | 114 | 73.7 | - | 8 | 42.1 | _ |
| Newcomer 2008 (147) | Aripiprazole | Schizophrenia, Schizoaffective | _ | 88 | 56.8 | 39.7 (10.1) | 16 | 2.5 | 11.1 |
|  | Olanzapine | Schizophrenia, Schizoaffective | _ | 85 | 71.8 | 38.7 (10.1) | 16 | 9.1 | 2.6 |
| Niufan 2008 (148) | Olanzapine | Bipolar I | _ | 69 | 43.5 | 31.2 (12.6) | 4 | 16.2 | _ |
| Olié 2006 (149) | Amisulpride | Schizophrenia | _ | 63 | 60.3 | 38.2 (-) | 12 | 17.9 | 5.4 |
|  | Ziprasidone | Schizophrenia | _ | 60 | 68.3 | 39.4 (-) | 12 | 8.8 | 7.0 |
| Ou 2013 (150) | Olanzapine | Schizophrenia | yes | 130 | 56.9 | 27.7 (8.0) | 6 | 43.8 | _ |
|  | Ziprasidone | Schizophrenia | yes | 130 | 53.1 | 26.9 (7.8) | 6 | 0.0 | _ |
| Pandina 2010 (151) | Paliperidone IM 25 mg eq. | Schizophrenia | _ | 160 | 67.0 | 39.0(-) | 9 | 6.0 | _ |
|  | Paliperidone IM 100 mg eq. | Schizophrenia | _ | 165 | 67.0 | 39.0(-) | 9 | 8.0 | _ |
|  | Paliperidone IM 150 mg eq. | Schizophrenia | _ | 163 | 67.0 | 39.0(-) | 9 | 13.0 | _ |
|  | Placebo | Schizophrenia | _ | 164 | 67.0 | 39.0(-) | 8 | 5.0 | _ |
| Peluso 2013 (152) | FGA | Schizophrenia, Scizoaffective, Schizofreniform, Delusional Disorder | _ | 118 | - | - | 12 | 22.2 | _ |
|  | FGA | Schizophrenia, Scizoaffective, Schizofreniform, Delusional Disorder | _ | 118 | - | - | 24 | 36.4 | _ |
|  | FGA | Schizophrenia, Scizoaffective, Schizofreniform, Delusional Disorder | _ | 118 | - | - | 52 | 50.5 | _ |
|  | SGA | Schizophrenia, Scizoaffective, Schizofreniform, Delusional Disorder | _ | 109 | - | - | 12 | 20.5 | _ |
|  | SGA | Schizophrenia, Scizoaffective, Schizofreniform, Delusional Disorder | _ | 109 | - | - | 24 | 48.8 | _ |
|  | SGA | Schizophrenia, Scizoaffective, Schizofreniform, Delusional Disorder | _ | 109 | - | - | 52 | 34.2 | _ |
| Perez-Iglesias 2014 (153) | Aripiprazole | Schizophrenia, Schizophrenia Spectrum Disorder | yes | 78 | 48.7 | 32.6 (11.1) | 12 | 45.6 | _ |
|  | Quetiapine | Schizophrenia, Schizophrenia Spectrum Disorder | yes | 62 | 66.1 | 31.0 (9.2) | 12 | 34.0 | _ |
|  | Ziprasidone | Schizophrenia, Schizophrenia Spectrum Disorder | yes | 62 | 46.8 | 32.1 (10.5) | 12 | 23.5 | _ |
| Peuskens 1997 (154) | Chlorpromazine | Schizophrenia | _ | 100 | 66.0 | 34.0 (11.0) | 6 | 18.0 | _ |
|  | Quetiapine | Schizophrenia | _ | 101 | 62.0 | 32.0 (10.0) | 6 | 27.0 | _ |
| Pigott 2003 (155) | Aripiprazole | Schizophrenia | _ | 151 | 54.2 | 42.2 (11.1) | 26 | 6.0 | _ |
|  | Placebo | Schizophrenia | _ | 151 | 58.1 | 41.7 (10.9) | 26 | 4.0 | _ |
| Potkin 2003 (156) | Aripiprazole 20 mg | Schizophrenia, Schizoaffective | _ | 101 | 72.3 | 38.1(-) | 4 | 13.0 | _ |
|  | Aripiprazole 30 mg | Schizophrenia, Schizoaffective | _ | 101 | 65.3 | 40.2(-) | 4 | 9.0 | _ |
|  | Placebo | Schizophrenia, Schizoaffective | _ | 103 | 70.9 | 38.8(-) | 4 | 2.0 | _ |
|  | Risperidone | Schizophrenia, Schizoaffective | _ | 99 | 71.7 | 38.6(-) | 4 | 11.0 | _ |
| Potkin 2007 (157) | Asenapine | Schizophrenia | _ | 59 | 78.0 | 38.0(-) | 6 | 4.3 | _ |
|  | Placebo | Schizophrenia | _ | 62 | 79.0 | 42.0(-) | 6 | 1.9 | _ |
|  | Risperidone | Schizophrenia | _ | 59 | 61.0 | 43.0(-) | 6 | 17.0 | _ |
| Potkin 2015 (158) | Haloperidol | Schizophrenia | _ | 72 | 80.6 | 40.0 (10.5) | 6 | 4.3 | _ |
|  | Lurasidone 40 mg | Schizophrenia | _ | 67 | 68.7 | 42.0 (10.9) | 6 | 3.0 | _ |
|  | Lurasidone 80 mg | Schizophrenia | _ | 71 | 73.2 | 42.2 (8.3) | 6 | 5.7 | _ |
|  | Lurasidone 20 mg | Schizophrenia | _ | 71 | 71.8 | 40.7 (10.5) | 6 | 1.5 | _ |
|  | Placebo | Schizophrenia | _ | 72 | 76.4 | 41.0 (9.7) | 6 | 2.9 | _ |
| Quiroz 2010 (159) | Placebo | Bipolar I | _ | 149 | 54.4 | 39.0 (12.4) | 104 | 3.0 | _ |
|  | Risperidone | Bipolar I | _ | 154 | 48.7 | 39.0 (11.8) | 104 | 12.0 | _ |
| Ryckmans 2009 (160) | Aripiprazole | Schizophrenia | _ | 200 | 52.0 | 41.7 (11.5) | 12 | 4.0 | 11.0 |
|  | Aripiprazole | Schizophrenia | _ | 200 | 60.0 | 40.5 (11.3) | 12 | 3.0 | 10.0 |
| Sacchetti 2008 (161) | Olanzapine | Schizophrenia | _ | 25 | 72.0 | 35.0 (11.0) | 8 | 29.0 | _ |
|  | Quetiapine | Schizophrenia | _ | 25 | 56.0 | 39.0 (9.0) | 8 | 8.0 | _ |
|  | Risperidone | Schizophrenia | _ | 25 | 40.0 | 43.0 (13.0) | 8 | 8.0 | _ |
| Sachs 2006 (162) | Aripiprazole | Bipolar I | _ | 137 | 50.3 | 37.3 (10.5) | 3 | 0.8 | _ |
|  | Placebo | Bipolar I | _ | 135 | 46.7 | 40.4 (10.5) | 3 | 4.2 | _ |
| Sachs 2015 (163) | Cariprazine | Bipolar I | _ | 158 | 66.5 | 35.8 (11.4) | 3 | 2.0 | _ |
|  | Placebo | Bipolar I | _ | 154 | 61.7 | 36.7 (11.8) | 3 | 2.0 | _ |
| Savitz 2016 (164) | Paliperidone IM (414 mg eq./3 months) | Schizophrenia | _ | 483 | 51.1 | 39.2 (11.9) | 48 | 15.2 | 7.5 |
|  | Paliperidone IM (119 mg eq./ 1 month) | Schizophrenia | _ | 512 | 54.9 | 38.3 (12.2) | 48 | 16.4 | 4.3 |
| Schmidt 2012 (165) | JNJ-37822681 20 mg | Schizophrenia | _ | 99 | 52.5 | 40.2 (11.7) | 12 | 7.1 | _ |
|  | JNJ-37822681 40 mg | Schizophrenia | _ | 103 | 63.1 | 40.9 (10.8) | 12 | 7.9 | _ |
|  | JNJ-37822681 60 mg | Schizophrenia | _ | 98 | 57.1 | 39.4 (10.6) | 12 | 10.1 | _ |
|  | Olanzapine | Schizophrenia | _ | 93 | 52.7 | 38.6 (10.8) | 12 | 25.8 | _ |
| Schneider 2006 (166) | Olanzapine | Alzheimer | _ | 100 | 45.0 | 78.8 (7.3) | 36 | 11.1 | _ |
|  | Placebo | Alzheimer | _ | 142 | 43.0 | 77.3 (7.1) | 36 | 3.1 | _ |
|  | Quetiapine | Alzheimer | _ | 94 | 47.0 | 77.3 (8.7) | 36 | 6.0 | _ |
|  | Risperidone | Alzheimer | _ | 85 | 42.0 | 78.4 (7.1) | 36 | 10.7 | _ |
| Schoemaker 2010 (167) | Asenapine | Schizophrenia, Schizoaffective | _ | 908 | 52.0 | 36.8 (11.8) | 52 | 15.0 | 5.0 |
|  | Olanzapine | Schizophrenia, Schizoaffective | _ | 311 | 59.0 | 36.2 (12.4) | 52 | 36.0 | 4.0 |
| Schreiner 2012 (168) | Olanzapine | Schizophrenia | _ | 220 | 60.5 | 37.5 (11.4) | 26 | 28.5 | _ |
|  | Paliperidone | Schizophrenia | _ | 239 | 55.6 | 38.8 (11.1) | 26 | 18.3 | _ |
| Schreiner 2015 (169) | Aripiprazole | Schizophrenia | _ | 81 | 55.4 | 32.6 (10.1) | 104 | 23.5 | _ |
|  | Haloperidol | Schizophrenia | _ | 34 | 55.4 | 32.6 (10.1) | 104 | 11.8 | _ |
|  | Olanzapine | Schizophrenia | _ | 48 | 55.4 | 32.6 (10.1) | 104 | 37.5 | _ |
|  | Paliperidone IM | Schizophrenia | _ | 352 | 60.5 | 32.6 (10.1) | 104 | 20.6 | _ |
|  | Paliperidone oral | Schizophrenia | _ | 76 | 55.4 | 32.6 (10.1) | 104 | 19.7 | _ |
|  | Quetiapine | Schizophrenia | _ | 65 | 55.4 | 32.6 (10.1) | 104 | 15.4 | _ |
|  | Risperidone | Schizophrenia | _ | 56 | 55.4 | 32.6 (10.1) | 104 | 26.8 | _ |
| Schulz 2008 (170) | Olanzapine | Borderline | _ | 155 | 27.1 | 31.8 (9.5) | 12 | 34.2 | _ |
|  | Placebo | Borderline | _ | 159 | 30.8 | 31.8 (9.6) | 12 | 2.6 | _ |
| Sheehan 2009(171) | Placebo | Bipolar I II NOS | _ | 57 | 38.6 | 38.4 (12.8) | 8 | 0.0 | _ |
|  | Risperidone | Bipolar I II NOS | _ | 54 | 33.3 | 35.1 (12.4) | 8 | 10.0 | _ |
| Sheehan 2013 (172) | Placebo | Bipolar I II NOS | _ | 51 | 41.0 | 37.6 (11.6) | 8 | 4.0 | _ |
|  | Quetiapine XR | Bipolar I II NOS | _ | 49 | 41.0 | 41.4 (12.1) | 8 | 12.0 | _ |
| Shen 2014 (173) | Olanzapine | Schizophrenia | _ | 71 | 64.8 | 40.1 (10.5) | 6 | 29.4 | _ |
|  | Placebo | Schizophrenia | _ | 71 | 73.3 | 39.6 (10.4) | 6 | 10.8 | _ |
|  | Vabicaserin 200 mg | Schizophrenia | _ | 77 | 75.3 | 39.3 (10.2) | 6 | 6.0 | _ |
|  | Vabicaserin 400 mg | Schizophrenia | _ | 70 | 75.7 | 41.8 (10.2) | 6 | 4.6 | _ |
| Small 1997 (174) | Placebo | Schizophrenia | _ | 96 | 67.0 | 38.0 (10.0) | 4 | 5.0 | _ |
|  | Quetiapine high dose | Schizophrenia | _ | 96 | 69.0 | 36.0 (9.0) | 4 | 25.0 | _ |
|  | Quetiapine low dose | Schizophrenia | _ | 94 | 78.0 | 37.0 (9.0) | 4 | 16.0 | _ |
| Srivastava 2012 (175) | Olanzapine | Bipolar | _ | 23 | 65.2 | 38.1 (12.0) | 1 | 4.3 | _ |
|  | Placebo | Bipolar | _ | 22 | 72.7 | 42.9 (9.9) | 1 | 0.0 | _ |
| Streim 2008 (176) | Aripiprazole | Alzheimer | _ | 131 | 26.0 | 83.0(-) | 10 | 1.0 | 10.0 |
|  | Placebo | Alzheimer | _ | 125 | 22.0 | 83.0(-) | 10 | 8.0 | 3.0 |
| Suppes 2010 (177) | Placebo | Bipolar | _ | 137 | 37.2 | 39.9 (12.8) | 8 | 0.8 | _ |
|  | Quetiapine | Bipolar | _ | 133 | 33.8 | 39.0 (11.3) | 8 | 8.2 | _ |
| Szegedi 2018 (178) | Asenapine | Bipolar I | _ | 126 | 42.1 | 42.8 (12.7) | 26 | 7.9 | _ |
|  | Placebo | Bipolar I | _ | 126 | 48.4 | 40.9 (13.5) | 26 | 7.1 | _ |
| Thase 2006 (179) | Placebo | Bipolar I II | _ | 161 | 39.8 | 37.7 (11.8) | 8 | 2.8 | _ |
|  | Quetiapine 300 mg | Bipolar I II | _ | 155 | 44.5 | 37.2 (10.5) | 8 | 3.9 | _ |
|  | Quetiapine 600 mg | Bipolar I II | _ | 151 | 45.0 | 38.2 (11.0) | 8 | 8.6 | _ |
| Thase 2008 (180) | Aripiprazole (Study 1) | Bipolar I | _ | 186 | 38.0 | 39.0 (11.0) | 8 | 6.7 | _ |
|  | Aripiprazole (Study 2) | Bipolar I | _ | 187 | 40.0 | 41.0 (12.0) | 8 | 2.9 | _ |
|  | Placebo (Study 1) | Bipolar I | _ | 188 | 37.0 | 39.0 (13.0) | 8 | 3.5 | _ |
|  | Placebo (Study 2) | Bipolar I | _ | 188 | 40.0 | 40.0 (12.0) | 8 | 2.6 | _ |
| Tohen 2003a (181) | Olanzapine | Bipolar I | _ | 125 | 42.4 | 40.0 (12.1) | 47 | 23.6 | _ |
| Tohen 2003b (182) | Haloperidol | Bipolar I | _ | 219 | 42.9 | 40.0 (13.0) | 12 | 16.1 | _ |
|  | Olanzapine | Bipolar I | _ | 234 | 36.8 | 41.0 (13.0) | 12 | 39.7 | _ |
| Tohen 2003c (183) | Olanzapine | Bipolar I | _ | 370 | 37.6 | 42.2 (12.5) | 8 | 18.7 | _ |
|  | Placebo | Bipolar I | _ | 377 | 37.4 | 41.7 (12.4) | 8 | 0.3 | _ |
| Tohen 2005 (184) | Olanzapine | Bipolar | _ | 217 | 47.9 | 42.5 (13.1) | 52 | 29.8 | _ |
| Tohen 2006 (185) | Olanzapine | Bipolar I | _ | 225 | 38.7 | 41.1 (12.1) | 48 | 16.1 | _ |
|  | Placebo | Bipolar I | _ | 136 | 39.0 | 39.8 (11.5) | 48 | 2.3 | _ |
| Tohen 2008 (186) | Olanzapine | Bipolar I | _ | 215 | 45.8 | 39.5 (11.9) | 3 | 6.4 | _ |
|  | Olanzapine | Bipolar I | _ | 215 | 45.8 | 39.5 (11.9) | 12 | 18.8 | _ |
|  | Placebo | Bipolar I | _ | 105 | 53.5 | 40.6 (12.8) | 3 | 1.0 | _ |
| Tohen 2012 (187) | Olanzapine | Bipolar I | _ | 343 | 40.2 | 36.0 (11.1) | 6 | 23.2 | _ |
|  | Placebo | Bipolar I | _ | 171 | 44.4 | 35.0 (11.0) | 6 | 1.2 | _ |
| Tollefson 1997 (188) | Haloperidol | Schizophrenia, Schizoaffective, Schizofreniform | _ | 660 | - | 38.3 (11.1) | 6 | _ | 4.6 |
|  | Olanzapine | Schizophrenia, Schizoaffective, Schizofreniform | _ | 1336 | - | 38.7 (11.6) | 6 | _ | 2.5 |
| Vanelle 2006 (189) | Amisulpride | Schizophrenia, Depression | _ | 45 | 68.9 | 32.6 (7.9) | 8 | 8.9 | _ |
|  | Olanzapine | Schizophrenia, Depression | _ | 40 | 57.5 | 36.5 (8.0) | 8 | 15.0 | _ |
| Vieta 2010 (190) | Paliperidone | Bipolar I | _ | 190 | 57.9 | 40.0 (11.3) | 12 | 8.0 | _ |
|  | Quetiapine | Bipolar I | _ | 192 | 59.9 | 39.0 (11.0) | 12 | 17.0 | _ |
| Wang 2012 (191) | Placebo | MDD | _ | 155 | 32.7 | 39.7 (11.1) | 10 | 0.6 | _ |
|  | Quetiapine XR | MDD | _ | 157 | 28.6 | 40.1 (11.6) | 10 | 1.9 | _ |
| Weiden 2014 (192) | Iloperidone (gradual switch) | Schizophrenia | _ | 240 | 70.8 | 42.3 (11.0) | 12 | 9.6 | _ |
|  | Iloperidone (immediate switch) | Schizophrenia | _ | 260 | 63.5 | 44.2 (10.9) | 12 | 7.7 | _ |
| Weiden 2016 (193) | Iloperidone | Schizophrenia | _ | 151 | 62.7 | 38.4 (11.3) | 12 | 25.2 | 12.6 |
|  | Placebo | Schizophrenia | _ | 150 | 54.7 | 38.2 (11.1) | 12 | 20.0 | 10.7 |
| Weisler 2009 (194) | Placebo | MDD | _ | 178 | 36.5 | 40.3 (11.8) | 6 | 1.1 | _ |
|  | Quetiapine XR 50 mg | MDD | _ | 178 | 46.6 | 40.6 (11.1) | 6 | 0.6 | _ |
|  | Quetiapine XR 150 mg | MDD | _ | 168 | 38.1 | 41.5 (11.7) | 6 | 3.6 | _ |
|  | Quetiapine XR 300 mg | MDD | _ | 168 | 41.5 | 40.7 (12.2) | 6 | 4.5 | _ |
| Weisler 2011 (195) | Placebo | Bipolar I | _ | 404 | 52.0 | 40.0 (12.9) | 11 | 2.6 | _ |
|  | Quetiapine | Bipolar I | _ | 404 | 45.0 | 39.9 (12.3) | 23 | 10.6 | _ |
| Xiang 2011 (196) | Risperidone (dose reduction after 4 weeks) | Schizophrenia | _ | 125 | 40.8 | 31.3 (10.6) | 52 | 36.8 | _ |
|  | Risperidone (dose reduction after 26 weeks) | Schizophrenia | _ | 120 | 49.2 | 32.7 (9.8) | 52 | 37.5 | _ |
|  | Risperidone (no dose reduction) | Schizophrenia | _ | 129 | 48.1 | 33.8 (11.8) | 52 | 37.2 | _ |
| Yang 2010 (197) | Blonanserin | Schizophrenia | _ | 92 | 51.1 | 34.5 (10.4) | 6 | 3.9 | _ |
|  | Risperidone | Schizophrenia | _ | 91 | 47.3 | 36.0 (10.2) | 7 | 6.5 | _ |
| Yatham 2020 (198) | Cariprazine 0.5-0.75 mg | Bipolar I II | _ | 75 | 36.0 | 37.4 (10.8) | 8 | 5.3 | _ |
|  | Cariprazine 1.5-3 mg | Bipolar I II | _ | 75 | 32.0 | 38.9 (11.2) | 8 | 6.8 | _ |
|  | Placebo | Bipolar I II | _ | 77 | 40.3 | 40.6 (10.7) | 8 | 0.0 | _ |
| Young 2009(199) | Aripiprazole | Bipolar I | _ | 126 | 43.0 | 40.5 (12.6) | 3 | 1.9 | _ |
|  | Aripiprazole | Bipolar I | _ | 95 | 43.0 | 40.5 (12.6) | 12 | 5.1 | _ |
|  | Haloperidol | Bipolar I | _ | 121 | 44.0 | 41.6 (14.1) | 3 | 2.6 | _ |
|  | Haloperidol | Bipolar I | _ | 95 | 44.0 | 41.6 (14.1) | 12 | 5.8 | _ |
|  | Placebo | Bipolar I | _ | 109 | 46.0 | 40.2 (13.2) | 3 | 6.4 | _ |
|  | Placebo | Bipolar I | _ | 84 | 46.0 | 40.2 (13.2) | 12 | _ | _ |
| Young 2010 (200) | Quetiapine 300 mg | Bipolar I II | _ | 260 | 42.7 | 42.3(-) | 8 | 4.6 | _ |
|  | Placebo | Bipolar I II | _ | 131 | 45.7 | 41.5(-) | 8 | 3.3 | _ |
|  | Quetiapine 600 mg | Bipolar I II | _ | 267 | 36.5 | 42.8(-) | 8 | 8.3 | _ |
| Zanarini 2011 (201) | Olanzapine 2.5 mg | Borderline | _ | 150 | 27.3 | 32.6 (11.2) | 12 | 20.3 | _ |
|  | Olanzapine 5-10 mg | Borderline | _ | 148 | 28.4 | 32.8 (10.0) | 12 | 30.6 | _ |
|  | Placebo | Borderline | _ | 153 | 23.5 | 33.5 (11.3) | 12 | 4.8 | _ |
| Zhong 2006 (202) | Quetiapine | Schizophrenia | _ | 338 | 77.1 | 40.2 (10.8) | 8 | 10.4 | _ |
|  | Risperidone | Schizophrenia | _ | 335 | 74.4 | 39.6 (10.8) | 8 | 10.5 | _ |

## Appendix 3.2: References of included studies

1. Adams DH, Kinon BJ, Baygani S, Millen BA, Velona I, Kollack-Walker S, et al. A long-term, phase 2, multicenter, randomized, open-label, comparative safety study of pomaglumetad methionil (LY2140023 monohydrate) versus atypical antipsychotic standard of care in patients with schizophrenia. BMC Psychiatry. 2013;13:143.

2. Adams DH, Zhang L, Millen BA, Kinon BJ, Gomez JC. Pomaglumetad methionil (ly2140023 monohydrate) and aripiprazole in patients with schizophrenia: A phase 3, multicenter, double-blind comparison. Schizophrenia Research and Treatment. 2014:758212.

3. Addington DE, Pantelis C, Dineen M, Benattia I, Romano SJ. Efficacy and tolerability of ziprasidone versus risperidone in patients with acute exacerbation of schizophrenia or schizoaffective disorder: an 8-week, double-blind, multicenter trial. J Clin Psychiatry. 2004;65(12):1624-33.

4. Alphs L, Benson C, Cheshire-Kinney K, Lindenmayer JP, Mao L, Rodriguez SC, et al. Real-world outcomes of paliperidone palmitate compared to daily oral antipsychotic therapy in schizophrenia: a randomized, open-label, review board-blinded 15-month study. J Clin Psychiatry. 2015;76(5):554-61.

5. Alvarez E, Bernardo M, Casares JRG, Montejo AL. Ziprasidone versus olanzapine in the weight gain associated with the treatment of schizophrenia: A six-month double-blind randomized parallel group study. European Journal of Psychiatry. 2012;26(4):248-59.

6. Arvanitis LA, Miller BG. Multiple fixed doses of "Seroquel" (quetiapine) in patients with acute exacerbation of schizophrenia: a comparison with haloperidol and placebo. The Seroquel Trial 13 Study Group. Biol Psychiatry. 1997;42(4):233-46.

7. Ascher-Svanum H, Stensland MD, Zhao Z, Kinon BJ. Acute weight gain, gender, and therapeutic response to antipsychotics in the treatment of patients with schizophrenia. BMC Psychiatry. 2005;5:3.

8. Bauer M, Dell'Osso L, Kasper S, Pitchot W, Vansvik ED, Kohler J, et al. Extended-release quetiapine fumarate (quetiapine XR) monotherapy and quetiapine XR or lithium as add-on to antidepressants in patients with treatment-resistant major depressive disorder. J Affect Disorders. 2013;151(1):209-19.

9. Berwaerts J, Xu H, Nuamah I, Lim P, Hough D. Evaluation of the efficacy and safety of paliperidone extended-release in the treatment of acute mania: a randomized, double-blind, dose-response study. J Affect Disord. 2012;136(1-2):e51-e60.

10. Berwaerts J, Melkote R, Nuamah I, Lim P. A randomized, placebo- and active-controlled study of paliperidone extended-release as maintenance treatment in patients with bipolar I disorder after an acute manic or mixed episode. J Affect Disord. 2012;138(3):247-58.

11. Berwaerts J, Liu Y, Gopal S, Nuamah I, Xu H, Savitz A, et al. Efficacy and Safety of the 3-Month Formulation of Paliperidone Palmitate vs Placebo for Relapse Prevention of Schizophrenia: A Randomized Clinical Trial. JAMA Psychiatry. 2015;72(8):830-9.

12. Borison RL, Arvanitis LA, Miller BG. ICI 204,636, an atypical antipsychotic: efficacy and safety in a multicenter, placebo-controlled trial in patients with schizophrenia. U.S. SEROQUEL Study Group. J Clin Psychopharmacol. 1996;16(2):158-69.

13. Bortnick B, El-Khalili N, Banov M, Adson D, Datto C, Raines S, et al. Efficacy and tolerability of extended release quetiapine fumarate (quetiapine XR) monotherapy in major depressive disorder: A placebo-controlled, randomized study. J Affect Disorders. 2011;128(1-2):83-94.

14. Buchanan RW, Panagides J, Zhao J, Phiri P, den Hollander W, Ha X, et al. Asenapine Versus Olanzapine in People With Persistent Negative Symptoms of Schizophrenia. Journal of Clinical Psychopharmacology. 2012;32(1):36-45.

15. Bushe C, Sniadecki J, Bradley AJ, Hoffmann VP. Comparison of metabolic and prolactin variables from a six-month randomised trial of olanzapine and quetiapine in schizophrenia. Journal of Psychopharmacology. 2010;24(7):1001-9.

16. Calabrese JR, Keck PE, Jr., Macfadden W, Minkwitz M, Ketter TA, Weisler RH, et al. A randomized, double-blind, placebo-controlled trial of quetiapine in the treatment of bipolar I or II depression. Am J Psychiatry. 2005;162(7):1351-60.

17. Calabrese JR, Sanchez R, Jin N, Amatniek J, Cox K, Johnson B, et al. Efficacy and Safety of Aripiprazole Once-Monthly in the Maintenance Treatment of Bipolar I Disorder: A Double-Blind, Placebo-Controlled, 52-Week Randomized Withdrawal Study. J Clin Psychiatry. 2017;78(3):324-31.

18. Cantillon M, Prakash A, Alexander A, Ings R, Sweitzer D, Bhat L. Dopamine serotonin stabilizer RP5063: A randomized, double-blind, placebo-controlled multicenter trial of safety and efficacy in exacerbation of schizophrenia or schizoaffective disorder. Schizophr Res. 2017;189:126-33.

19. Canuso CM, Lindenmayer JP, Kosik-Gonzalez C, Turkoz I, Carothers J, Bossie CA, et al. A randomized, double-blind, placebo-controlled study of 2 dose ranges of paliperidone extended-release in the treatment of subjects with schizoaffective disorder. J Clin Psychiatry. 2010;71(5):587-98.

20. Canuso CM, Schooler N, Carothers J, Turkoz I, Kosik-Gonzalez C, Bossie CA, et al. Paliperidone Extended-Release in Schizoaffective Disorder. Journal of Clinical Psychopharmacology. 2010;30(5):487-95.

21. Casey DE, Carson WH, Saha AR, Liebeskind A, Ali MW, Jody D, et al. Switching patients to aripiprazole from other antipsychotic agents: a multicenter randomized study. Psychopharmacology (Berl). 2003;166(4):391-9.

22. Casey DE, Sands EE, Heisterberg J, Yang HM. Efficacy and safety of bifeprunox in patients with an acute exacerbation of schizophrenia: results from a randomized, double-blind, placebo-controlled, multicenter, dose-finding study. Psychopharmacology (Berl). 2008;200(3):317-31.

23. Chan HY, Lin WW, Lin SK, Hwang TJ, Su TP, Chiang SC, et al. Efficacy and safety of aripiprazole in the acute treatment of schizophrenia in Chinese patients with risperidone as an active control: a randomized trial. J Clin Psychiatry. 2007;68(1):29-36.

24. Chan HY, Chang CJ, Chiang SC, Chen JJ, Chen CH, Sun HJ, et al. A randomised controlled study of risperidone and olanzapine for schizophrenic patients with neuroleptic-induced acute dystonia or parkinsonism. Journal of Psychopharmacology. 2010;24(1):91-8.

25. Chen EY, Hui CL, Lam MM, Chiu CP, Law CW, Chung DW, et al. Maintenance treatment with quetiapine versus discontinuation after one year of treatment in patients with remitted first episode psychosis: randomised controlled trial. BMJ. 2010;341:c4024.

26. Chen Y, Bobo WV, Watts K, Jayathilake K, Tang T, Meltzer HY. Comparative effectiveness of switching antipsychotic drug treatment to aripiprazole or ziprasidone for improving metabolic profile and atherogenic dyslipidemia: a 12-month, prospective, open-label study. J Psychopharmacol. 2012;26(9):1201-10.

27. Chen JJ, Chan HY, Chen CH, Gau SS, Hwu HG. Risperidone and olanzapine versus another first generation antipsychotic in patients with schizophrenia inadequately responsive to first generation antipsychotics. Pharmacopsychiatry. 2012;45(2):64-71.

28. Cheng Z, Yuan Y, Han X, Yang L, Cai S, Yang F, et al. An open-label randomised comparison of aripiprazole, olanzapine and risperidone for the acute treatment of first-episode schizophrenia: Eight-week outcomes. J Psychopharmacol. 2019;33(10):1227-36.

29. Chengappa KN, Turkin SR, Schlicht PJ, Murphy SL, Brar JS, Fagiolini A, et al. A Pilot, 15-month, randomised effectiveness trial of Risperidone long-acting injection (RLAI) versus oral atypical antipsychotic agents (AAP) in persons with bipolar disorder. Acta Neuropsychiatr. 2010;22(2):68-80.

30. Chrzanowski WK, Marcus RN, Torbeyns A, Nyilas M, McQuade RD. Effectiveness of long-term aripiprazole therapy in patients with acutely relapsing or chronic, stable schizophrenia: a 52-week, open-label comparison with olanzapine. Psychopharmacology. 2006;189(2):259-66.

31. Citrome L, Cucchiaro J, Sarma K, Phillips D, Silva R, Tsuchiya S, et al. Long-term safety and tolerability of lurasidone in schizophrenia. International Clinical Psychopharmacology. 2012;27(3):165-76.

32. Conley RR, Mahmoud R. A randomized double-blind study of risperidone and olanzapine in the treatment of schizophrenia or schizoaffective disorder. Am J Psychiatry. 2001;158(5):765-74.

33. Coppola D, Melkote R, Lannie C, Singh J, Nuamah I, Gopal S, et al. Efficacy and safety of paliperidone extended release 1.5 mg/day-A Double-Blind, placebo- and active-Controlled, study in the treatment of patients with schizophrenia. Psychopharmacology Bulletin. 2011;44(2).

34. Correll CU, Skuban A, Ouyang J, Hobart M, Pfister S, McQuade RD, et al. Efficacy and Safety of Brexpiprazole for the Treatment of Acute Schizophrenia: A 6-Week Randomized, Double-Blind, Placebo-Controlled Trial. Am J Psychiatry. 2015;172(9):870-80.

35. Correll CU, Davis RE, Weingart M. Efficacy and Safety of Lumateperone for Treatment of Schizophrenia: A Randomized Clinical Trial (vol 13, pg 512, 2020). Jama Psychiatry. 2020;77(4):438-.

36. Correll CU, Newcomer JW, Silverman B, DiPetrillo L, Graham C, Jiang Y, et al. Effects of Olanzapine Combined With Samidorphan on Weight Gain in Schizophrenia: A 24-Week Phase 3 Study. Am J Psychiatry. 2020;177(12):1168-78.

37. Cutler AJ, Kalali AH, Weiden PJ, Hamilton J, Wolfgang CD. Four-week, double-blind, placebo- and ziprasidone-controlled trial of iloperidone in patients with acute exacerbations of schizophrenia. J Clin Psychopharmacol. 2008;28(2 Suppl 1):S20-8.

38. Cutler AJ, Montgomery SA, Feifel D, Lazarus A, Aström M, Brecher M. Extended release quetiapine fumarate monotherapy in major depressive disorder: a placebo- and duloxetine-controlled study. J Clin Psychiatry. 2009;70(4):526-39.

39. Cutler AJ, Datto C, Nordenhem A, Minkwitz M, Acevedo L, Darko D. Extended-release quetiapine as monotherapy for the treatment of adults with acute mania: A randomized, double-blind, 3-week trial. Clinical Therapeutics. 2011;33(11):1643-58.

40. Cutler AJ, Tran-Johnson T, Kalali A, Astrom M, Brecher M, Meulien D. A failed 6-week, randomized, double-blind, placebo-controlled study of once-daily extended release quetiapine fumarate in patients with acute schizophrenia: Lessons learned. Psychopharmacology Bulletin. 2011;44(4):37-69.

41. Davidson M, Emsley R, Kramer M, Ford L, Pan G, Lim P, et al. Efficacy, safety and early response of paliperidone extended-release tablets (paliperidone ER): Results of a 6-week, randomized, placebo-controlled study(vol 93, pg 117, 2007). Schizophr Res. 2007;96(1-3):273-4.

42. De Deyn P, Jeste DV, Swanink R, Kostic D, Breder C. Aripiprazole for the treatment of psychosis in patients with Alzheimer's disease: A randomized, placebo-controlled study. Journal of Clinical Psychopharmacology. 2005;25(5):463-7.

43. De Hert M, Mittoux A, He Y, Peuskens J. Metabolic parameters in the short- and long-term treatment of schizophrenia with sertindole or risperidone. Eur Arch Psychiatry Clin Neurosci. 2011;261(4):231-9.

44. Deberdt WG, Dysken MW, Rappaport SA, Feldman PD, Young CA, Hay DP, et al. Comparison of Olanzapine and Risperidone in the Treatment of Psychosis and Associated Behavioral Disturbances in Patients With Dementia. The American Journal of Geriatric Psychiatry. 2005;13(8):722-30.

45. Detke HC, Weiden PJ, Llorca PM, Choukour M, Watson SB, Brunner E, et al. Comparison of olanzapine long-acting injection and oral olanzapine: a 2-year, randomized, open-label study in outpatients with schizophrenia. J Clin Psychopharmacol. 2014;34(4):426-34.

46. Dossenbach M, Treuer T, Kryzhanovskaya L, Saylan M, Dominguez S, Huang X. Olanzapine versus chlorpromazine in the treatment of schizophrenia: a pooled analysis of four 6-week, randomized, open-label studies in the Middle East and North Africa. J Clin Psychopharmacol. 2007;27(4):329-37.

47. Dubovsky SL, Frobose C, Phiri P, de Greef R, Panagides J. Short-term safety and pharmacokinetic profile of asenapine in older patients with psychosis. Int J Geriatr Psychiatry. 2012;27(5):472-82.

48. Durgam S, Starace A, Li D, Migliore R, Ruth A, Nemeth G, et al. An evaluation of the safety and efficacy of cariprazine in patients with acute exacerbation of schizophrenia: a phase II, randomized clinical trial. Schizophr Res. 2014;152(2-3):450-7.

49. Durgam S, Starace A, Li D, Migliore R, Ruth A, Nemeth G, et al. The efficacy and tolerability of cariprazine in acute mania associated with bipolar I disorder: a phase II trial. Bipolar Disord. 2015;17(1):63-75.

50. Durgam S, Cutler AJ, Lu K, Migliore R, Ruth A, Laszlovszky I, et al. Cariprazine in acute exacerbation of schizophrenia: a fixed-dose, phase 3, randomized, double-blind, placebo- and active-controlled trial. J Clin Psychiatry. 2015;76(12):e1574-82.

51. Durgam S, Earley W, Lipschitz A, Guo H, Laszlovszky I, Nemeth G, et al. An 8-Week Randomized, Double-Blind, Placebo-Controlled Evaluation of the Safety and Efficacy of Cariprazine in Patients With Bipolar I Depression. Am J Psychiatry. 2016;173(3):271-81.

52. Durgam S, Earley W, Li R, Li D, Lu K, Laszlovszky I, et al. Long-term cariprazine treatment for the prevention of relapse in patients with schizophrenia: A randomized, double-blind, placebo-controlled trial. Schizophr Res. 2016;176(2-3):264-71.

53. Durgam S, Landbloom RP, Mackle M, Wu X, Mathews M, Nasrallah HA. Exploring the long-term safety of asenapine in adults with schizophrenia in a double-blind, fixed-dose, extension study. Neuropsychiatr Dis Treat. 2017;13:2021-35.

54. Earley WR, Burgess MV, Khan B, Rekeda L, Suppes T, Tohen M, et al. Efficacy and safety of cariprazine in bipolar I depression: A double-blind, placebo-controlled phase 3 study. Bipolar Disorders. 2019.

55. Fleischhacker WW, McQuade RD, Marcus RN, Archibald D, Swanink R, Carson WH. A double-blind, randomized comparative study of aripiprazole and olanzapine in patients with schizophrenia. Biol Psychiatry. 2009;65(6):510-7.

56. Fleischhacker WW, Gopal S, Lane R, Gassmann-Mayer C, Lim P, Hough D, et al. A randomized trial of paliperidone palmitate and risperidone long-acting injectable in schizophrenia. Int J Neuropsychoph. 2012;15(1):107-18.

57. Fleischhacker WW, Sanchez R, Perry PP, Jin N, Peters-Strickland T, Johnson BR, et al. Aripiprazole once-monthly for treatment of schizophrenia: double-blind, randomised, non-inferiority study. Brit J Psychiat. 2014;205(2):135-44.

58. Fleischhacker WW, Hobart M, Ouyang J, Forbes A, Pfister S, McQuade RD, et al. Efficacy and Safety of Brexpiprazole (OPC-34712) as Maintenance Treatment in Adults with Schizophrenia: a Randomized, Double-Blind, Placebo-Controlled Study. Int J Neuropsychopharmacol. 2017;20(1):11-21.

59. Fu DJ, Turkoz I, Bruce Simonson R, Walling DP, Schooler NR, Lindenmayer JP, et al. Paliperidone Palmitate once-monthly reduces risk of relapse of psychotic, depressive, and manic symptoms and maintains functioning in a double-blind, randomized study of schizoaffective disorder. Journal of Clinical Psychiatry. 2015;76(3):253-62.

60. Garcia E, Robert M, Peris F, Nakamura H, Sato N, Terazawa Y. The efficacy and safety of blonanserin compared with haloperidol in acute-phase schizophrenia: a randomized, double-blind, placebo-controlled, multicentre study. CNS Drugs. 2009;23(7):615-25.

61. Gopal S, Hough DW, Xu HY, Lull JM, Gassmann-Mayer C, Remmerie BM, et al. Efficacy and safety of paliperidone palmitate in adult patients with acutely symptomatic schizophrenia: a randomized, double-blind, placebo-controlled, dose-response study. International Clinical Psychopharmacology. 2010;25(5):247-56.

62. Green AI, Lieberman JA, Hamer RM, Glick ID, Gur RE, Kahn RS, et al. Olanzapine and haloperidol in first episode psychosis: two-year data. Schizophr Res. 2006;86(1-3):234-43.

63. Grootens KP, van Veelen NM, Peuskens J, Sabbe BG, Thys E, Buitelaar JK, et al. Ziprasidone vs olanzapine in recent-onset schizophrenia and schizoaffective disorder: results of an 8-week double-blind randomized controlled trial. Schizophr Bull. 2011;37(2):352-61.

64. Grossberg GT, Kohegyi E, Mergel V, Josiassen MK, Meulien D, Hobart M, et al. Efficacy and Safety of Brexpiprazole for the Treatment of Agitation in Alzheimer's Dementia: Two 12-Week, Randomized, Double-Blind, Placebo-Controlled Trials. Am J Geriat Psychiat. 2020;28(4):383-400.

65. Higuchi T, Ishigooka J, Iyo M, Yeh CB, Ebenezer EG, Liang KY, et al. Lurasidone in the treatment of schizophrenia: Results of a double-blind, placebo-controlled trial in Asian patients. Asia Pac Psychiatry. 2019;11(2):e12352.

66. Higuchi T, Iyo M, Kwon JS, Chou YH, Chen HK, Chen JY, et al. Randomized, double-blind, placebo, and risperidone-controlled study of lurasidone in the treatment of schizophrenia: Results of an inconclusive 6-week trial. Asia-Pacific Psychiatry. 2019;11(3):e12354.

67. Hill AL, Sun B, Karagianis JL, Watson SB, McDonnell DP. Dose-associated changes in safety and efficacy parameters observed in a 24-week maintenance trial of olanzapine long-acting injection in patients with schizophrenia. BMC Psychiatry. 2011;11:28.

68. Hobart M, Skuban A, Zhang P, Josiassen MK, Hefting N, Augustine C, et al. Efficacy and safety of flexibly dosed brexpiprazole for the adjunctive treatment of major depressive disorder: a randomized, active-referenced, placebo-controlled study. Current Medical Research and Opinion. 2018;34(4):633-42.

69. Hobart M, Skuban A, Zhang P, Augustine C, Brewer C, Hefting N, et al. A randomized, placebo-controlled study of the efficacy and safety of fixed-dose brexpiprazole 2 mg/d as adjunctive treatment of adults with major depressive disorder. Journal of Clinical Psychiatry. 2018;79(4):17m12058.

70. Honer WG, MacEwan GW, Gendron A, Stip E, Labelle A, Williams R, et al. A randomized, double-blind, placebo-controlled study of the safety and tolerability of high-dose quetiapine in patients with persistent symptoms of schizophrenia or schizoaffective disorder. J Clin Psychiatry. 2012;73(1):13-20.

71. Hough D, Gopal S, Vijapurkar U, Lim P, Morozova M, Eerdekens M. Paliperidone palmitate maintenance treatment in delaying the time-to-relapse in patients with schizophrenia: a randomized, double-blind, placebo-controlled study. Schizophr Res. 2010;116(2-3):107-17.

72. Ishigooka J, Iwashita S, Tadori Y. Efficacy and safety of brexpiprazole for the treatment of acute schizophrenia in Japan: A 6-week, randomized, double-blind, placebo-controlled study. Psychiat Clin Neuros. 2018;72(9):692-700.

73. Iyo M, Ishigooka J, Nakamura M, Sakaguchi R, Okamoto K, Mao Y, et al. Efficacy and safety of lurasidone in acutely psychotic patients with schizophrenia: A 6-week, randomized, double-blind, placebo-controlled study. Psychiatry and Clinical Neurosciences. 2021.

74. Jeste DV, Barak Y, Madhusoodanan S, Grossman F, Gharabawi G. International multisite double-blind trial of the atypical antipsychotics risperidone and olanzapine in 175 elderly patients with chronic schizophrenia. Am J Geriat Psychiat. 2003;11(6):638-47.

75. Jindal KC, Singh GP, Munjal V. Aripiprazole versus olanzapine in the treatment of schizophrenia: a clinical study from India. Int J Psychiatry Clin Pract. 2013;17(1):21-9.

76. Kamijima K, Higuchi T, Ishigooka J, Ohmori T, Ozaki N, Kanba S, et al. Aripiprazole augmentation to antidepressant therapy in Japanese patients with major depressive disorder: A randomized, double-blind, placebo-controlled study (ADMIRE study). J Affect Disorders. 2013;151(3):899-905.

77. Kanba S, Kawasaki H, Ishigooka J, Sakamoto K, Kinoshita T, Kuroki T. A placebo-controlled, double-blind study of the efficacy and safety of aripiprazole for the treatment of acute manic or mixed episodes in Asian patients with bipolar I disorder (the AMAZE study). World J Biol Psychiatry. 2014;15(2):113-21.

78. Kane JM, Carson WH, Saha AR, McQuade RD, Ingenito GG, Zimbroff DL, et al. Efficacy and safety of aripiprazole and haloperidol versus placebo in patients with schizophrenia and schizoaffective disorder. J Clin Psychiatry. 2002;63(9):763-71.

79. Kane JM, Khanna S, Rajadhyaksha S, Giller E. Efficacy and tolerability of ziprasidone in patients with treatment-resistant schizophrenia. Int Clin Psychopharmacol. 2006;21(1):21-8.

80. Kane J, Canas F, Kramer M, Ford L, Gassmann-Mayer C, Lim P, et al. Treatment of schizophrenia with paliperidone extended-release tablets: a 6-week placebo-controlled trial. Schizophr Res. 2007;90(1-3):147-61.

81. Kane JM, Kim E, Kan HJ, Guo Z, Bates JA, Whitehead R, et al. Comparative utility of aripiprazole and haloperidol in schizophrenia: post hoc analysis of two 52-week, randomized, controlled trials. Appl Health Econ Health Policy. 2009;7(2):109-19.

82. Kane JM, Osuntokun O, Kryzhanovskaya LA, Xu W, Stauffer VL, Watson SB, et al. A 28-week, randomized, double-blind study of olanzapine versus aripiprazole in the treatment of schizophrenia. J Clin Psychiatry. 2009;70(4):572-81.

83. Kane JM, Cohen M, Zhao J, Alphs L, Panagides J. Efficacy and safety of asenapine in a placebo- and haloperidol-controlled trial in patients with acute exacerbation of schizophrenia. J Clin Psychopharmacol. 2010;30(2):106-15.

84. Kane JM, Mackle M, Snow-Adami L, Zhao J, Szegedi A, Panagides J. A randomized placebo-controlled trial of asenapine for the prevention of relapse of schizophrenia after long-term treatment. J Clin Psychiatry. 2011;72(3):349-55.

85. Kane JM, Potkin SG, Daniel DG, Buckley PF. A double-blind, randomized study comparing the efficacy and safety of sertindole and risperidone in patients with treatment-resistant schizophrenia. J Clin Psychiatry. 2011;72(2):194-204.

86. Kane JM, Sanchez R, Perry PP, Jin N, Johnson BR, Forbes RA, et al. Aripiprazole intramuscular depot as maintenance treatment in patients with schizophrenia: a 52-week, multicenter, randomized, double-blind, placebo-controlled study. J Clin Psychiatry. 2012;73(5):617-24.

87. Kane JM, Peters-Strickland T, Baker RA, Hertel P, Eramo A, Jin N, et al. Aripiprazole Once-Monthly in the Acute Treatment of Schizophrenia. The Journal of Clinical Psychiatry. 2014;75(11):1254-60.

88. Kane JM, Zukin S, Wang Y, Lu K, Ruth A, Nagy K, et al. Efficacy and Safety of Cariprazine in Acute Exacerbation of Schizophrenia: Results From an International, Phase III Clinical Trial. J Clin Psychopharmacol. 2015;35(4):367-73.

89. Kane JM, Skuban A, Ouyang J, Hobart M, Pfister S, McQuade RD, et al. A multicenter, randomized, double-blind, controlled phase 3 trial of fixed-dose brexpiprazole for the treatment of adults with acute schizophrenia. Schizophr Res. 2015;164(1-3):127-35.

90. Karagianis J, Grossman L, Landry J, Reed VA, de Haan L, Maguire GA, et al. A randomized controlled trial of the effect of sublingual orally disintegrating olanzapine versus oral olanzapine on body mass index: the PLATYPUS Study. Schizophr Res. 2009;113(1):41-8.

91. Katagiri H, Takita Y, Tohen M, Higuchi T, Kanba S, Takahashi M. Efficacy and safety of olanzapine in the treatment of Japanese patients with bipolar I disorder in a current manic or mixed episode: a randomized, double-blind, placebo- and haloperidol-controlled study. J Affect Disord. 2012;136(3):476-84.

92. Katila H, Mezhebovsky I, Mulroy A, Berggren L, Eriksson H, Earley W, et al. Randomized, double-blind study of the efficacy and tolerability of extended release quetiapine fumarate (quetiapine XR) monotherapy in elderly patients with major depressive disorder. Am J Geriat Psychiat. 2013;21(8):769-84.

93. Kato T, Ishigooka J, Miyajima M, Watabe K, Fujimori T, Masuda T, et al. Double-blind, placebo-controlled study of lurasidone monotherapy for the treatment of bipolar I depression. Psychiat Clin Neuros. 2020;74(12):635-44.

94. Keck PE, Jr., Marcus R, Tourkodimitris S, Ali M, Liebeskind A, Saha A, et al. A placebo-controlled, double-blind study of the efficacy and safety of aripiprazole in patients with acute bipolar mania. Am J Psychiatry. 2003;160(9):1651-8.

95. Keck Jr PE, Calabrese JR, McQuade RD, Carson WH, Carlson BX, Rollin LM, et al. A randomized, double-blind, placebo-controlled 26-week trial of aripiprazole in recently manic patients with bipolar I disorder. Journal of Clinical Psychiatry. 2006;67(4):626-37.

96. Keck PE, Jr., Calabrese JR, McIntyre RS, McQuade RD, Carson WH, Eudicone JM, et al. Aripiprazole monotherapy for maintenance therapy in bipolar I disorder: a 100-week, double-blind study versus placebo. J Clin Psychiatry. 2007;68(10):1480-91.

97. Keks NA, Ingham M, Khan A, Karcher K. Long-acting injectable risperidone v. olanzapine tablets for schizophrenia or schizoaffective disorder. Randomised, controlled, open-label study. Br J Psychiatry. 2007;191:131-9.

98. Kerwin R, Millet B, Herman E, Banki CM, Lublin H, Pans M, et al. A multicentre, randomized, naturalistic, open-label study between aripiprazole and standard of care in the management of community-treated schizophrenic patients Schizophrenia Trial of Aripiprazole: (STAR) study. European Psychiatry. 2007;22(7):433-43.

99. Ketter TA, Durgam S, Landbloom R, Mackle M, Wu X, Mathews M. Long-term safety and tolerability of asenapine: A double-blind, uncontrolled, long-term extension trial in adults with an acute manic or mixed episode associated with bipolar I disorder. J Affect Disord. 2017;207:384-92.

100. Kim SW, Shin IS, Kim JM, Lee SH, Lee JH, Yoon BH, et al. Amisulpride versus risperidone in the treatment of depression in patients with schizophrenia: a randomized, open-label, controlled trial. Prog Neuropsychopharmacol Biol Psychiatry. 2007;31(7):1504-9.

101. Kim SW, Chung YC, Lee YH, Lee JH, Kim SY, Bae KY, et al. Paliperidone ER versus risperidone for neurocognitive function in patients with schizophrenia: a randomized, open-label, controlled trial. Int Clin Psychopharmacol. 2012;27(5):267-74.

102. Kinon BJ, Lipkovich I, Edwards SB, Adams DH, Ascher-Svanum H, Siris SG. A 24-week randomized study of olanzapine versus ziprasidone in the treatment of schizophrenia or schizoaffective disorder in patients with prominent depressive symptoms. J Clin Psychopharmacol. 2006;26(2):157-62.

103. Kinon BJ, Volavka J, Stauffer V, Edwards SE, Liu-Seifert H, Chen L, et al. Standard and higher dose of olanzapine in patients with schizophrenia or schizoaffective disorder: A randomized, double-blind, fixed-dose study. Journal of Clinical Psychopharmacology. 2008;28(4):392-400.

104. Kinon BJ, Zhang L, Millen BA, Osuntokun OO, Williams JE, Kollack-Walker S, et al. A multicenter, inpatient, phase 2, double-blind, placebo-controlled dose-ranging study of LY2140023 monohydrate in patients with DSM-IV schizophrenia. Journal of Clinical Psychopharmacology. 2011;31(3):349-55.

105. Kinoshita T, Bai Y-M, Kim J-H, Miyake M, Oshima N. Efficacy and safety of asenapine in Asian patients with an acute exacerbation of schizophrenia: A multicentre, randomized, double-blind, 6-week, placebo-controlled study. Psychopharmacology. 2016;233(14):2663-74.

106. Kishi T, Matsuda Y, Matsunaga S, Mukai T, Moriwaki M, Tabuse H, et al. A randomized trial of aripiprazole vs blonanserin for the treatment of acute schizophrenia and related disorders. Neuropsychiatric Disease and Treatment. 2016;12.

107. Krakowski M, Czobor P, Citrome L. Weight gain, metabolic parameters, and the impact of race in aggressive inpatients randomized to double-blind clozapine, olanzapine or haloperidol. Schizophr Res. 2009;110(1-3):95-102.

108. Kramer M, Simpson G, Maciulis V, Kushner S, Vijapurkar U, Lim P, et al. Paliperidone extended-release tablets for prevention of symptom recurrence in patients with schizophrenia: a randomized, double-blind, placebo-controlled study. J Clin Psychopharmacol. 2007;27(1):6-14.

109. Kramer M, Litman R, Hough D, Lane R, Lim P, Liu YN, et al. Paliperidone palmitate, a potential long-acting treatment for patients with schizophrenia. Results of a randomized, double-blind, placebo-controlled efficacy and safety study. Int J Neuropsychoph. 2010;13(5):635-47.

110. Landbloom RL, Mackle M, Wu X, Kelly L, Snow-Adami L, McIntyre RS, et al. Asenapine: Efficacy and safety of 5 and 10mg bid in a 3-week, randomized, double-blind, placebo-controlled trial in adults with a manic or mixed episode associated with bipolar I disorder. J Affect Disord. 2016;190:103-10.

111. Landbloom R, Mackle M, Wu X, Kelly L, Snow-Adami L, McIntyre RS, et al. Asenapine for the treatment of adults with an acute exacerbation of schizophrenia: results from a randomized, double-blind, fixed-dose, placebo-controlled trial with olanzapine as an active control. CNS Spectr. 2017;22(4):333-41.

112. Langosch JM, Drieling T, Biedermann NC, Born C, Sasse J, Bauer H, et al. Efficacy of quetiapine monotherapy in rapid-cycling bipolar disorder in comparison with sodium valproate. J Clin Psychopharmacol. 2008;28(5):555-60.

113. Lauriello J, Lambert T, Andersen S, Lin D, Taylor CC, McDonnell D. An 8-week, double-blind, randomized, placebo-controlled study of olanzapine long-acting injection in acutely ill patients with schizophrenia. J Clin Psychiatry. 2008;69(5):790-9.

114. Li H, Luo J, Wang C, Xie S, Xu X, Wang X, et al. Efficacy and safety of aripiprazole in Chinese Han schizophrenia subjects: a randomized, double-blind, active parallel-controlled, multicenter clinical trial. Schizophr Res. 2014;157(1-3):112-9.

115. Li H, Yao C, Shi J, Yang F, Qi S, Wang L, et al. Comparative study of the efficacy and safety between blonanserin and risperidone for the treatment of schizophrenia in Chinese patients: A double-blind, parallel-group multicenter randomized trial. Journal of Psychiatric Research. 2015;69:102-9.

116. Li H, Gu N, Wang G, Tan Q, Yang F, Ning Y, et al. Efficacy and safety of quetiapine extended release monotherapy in bipolar depression: A multi-center, randomized, double-blind, placebo-controlled trial. Psychopharmacology. 2016;233(7):1289-97.

117. Lieberman JA, Tollefson G, Tohen M, Green AI, Gur RE, Kahn R, et al. Comparative efficacy and safety of atypical and conventional antipsychotic drugs in first-episode psychosis: a randomized, double-blind trial of olanzapine versus haloperidol. Am J Psychiatry. 2003;160(8):1396-404.

118. Lieberman JA, Scott Stroup T, McEvoy JP, Swartz MS, Rosenheck RA, Perkins DO, et al. Effectiveness of antipsychotic drugs in patients with chronic schizophrenia. New England Journal of Medicine. 2005;353(12):1209-23.

119. Loebel A, Cucchiaro J, Sarma K, Xu L, Hsu C, Kalali AH, et al. Efficacy and safety of lurasidone 80 mg/day and 160 mg/day in the treatment of schizophrenia: a randomized, double-blind, placebo- and active-controlled trial. Schizophr Res. 2013;145(1-3):101-9.

120. Loebel A, Cucchiaro J, Silva R, Kroger H, Hsu J, Sarma K, et al. Lurasidone Monotherapy in the Treatment of Bipolar I Depression: A Randomized, Double-Blind, Placebo-Controlled Study. American Journal of Psychiatry. 2014;171(2):160-8.

121. Macfadden W, Alphs L, Haskins JT, Turner N, Turkoz I, Bossie C, et al. A randomized, double-blind, placebo-controlled study of maintenance treatment with adjunctive risperidone long-acting therapy in patients with bipolar I disorder who relapse frequently. Bipolar Disorders. 2009;11(8):827-39.

122. Marder SR, Kramer M, Ford L, Eerdekens E, Lim P, Eerdekens M, et al. Efficacy and safety of paliperidone extended-release tablets: results of a 6-week, randomized, placebo-controlled study. Biol Psychiatry. 2007;62(12):1363-70.

123. Martin S, Lĵo H, Peuskens J, Thirumalai S, Giudicelli A, Fleurot O, et al. A double-blind, randomised comparative trial of amisulpride versus olanzapine in the treatment of schizophrenia: short-term results at two months. Curr Med Res Opin. 2002;18(6):355-62.

124. McDonnell DP, Kryzhanovskaya LA, Zhao F, Detke HC, Feldman PD. Comparison of metabolic changes in patients with schizophrenia during randomized treatment with intramuscular olanzapine long-acting injection versus oral olanzapine. Hum Psychopharmacol. 2011;26(6):422-33.

125. McElroy SL, Weisler RH, Chang W, Olausson B, Paulsson B, Brecher M, et al. A Double-Blind, Placebo-Controlled Study of Quetiapine and Paroxetine as Monotherapy in Adults With Bipolar Depression (EMBOLDEN II). Journal of Clinical Psychiatry. 2010;71(2):163-74.

126. McEvoy JP, Lieberman JA, Stroup TS, Davis SM, Meltzer HY, Rosenheck RA, et al. Effectiveness of clozapine versus olanzapine, quetiapine, and risperidone in patients with chronic schizophrenia who did not respond to prior atypical antipsychotic treatment. Am J Psychiatry. 2006;163(4):600-10.

127. McEvoy JP, Daniel DG, Carson Jr WH, McQuade RD, Marcus RN. A randomized, double-blind, placebo-controlled, study of the efficacy and safety of aripiprazole 10, 15 or 20 mg/day for the treatment of patients with acute exacerbations of schizophrenia. Journal of Psychiatric Research. 2007;41(11):895-905.

128. McIntyre RS, Brecher M, Paulsson B, Huizar K, Mullen J. Quetiapine or haloperidol as monotherapy for bipolar mania—a 12-week, double-blind, randomised, parallel-group, placebo-controlled trial. European Neuropsychopharmacology. 2005;15(5):573-85.

129. McIntyre RS, Cohen M, Zhao J, Alphs L, Macek TA, Panagides J. A 3-week, randomized, placebo-controlled trial of asenapine in the treatment of acute mania in bipolar mania and mixed states. Bipolar Disord. 2009;11(7):673-86.

130. McIntyre RS, Cohen M, Zhao J, Alphs L, Macek TA, Panagides J. Asenapine in the treatment of acute mania in bipolar I disorder: a randomized, double-blind, placebo-controlled trial. J Affect Disord. 2010;122(1-2):27-38.

131. McIntyre RS, Cohen M, Zhao J, Alphs L, Macek TA, Panagides J. Asenapine for long-term treatment of bipolar disorder: a double-blind 40-week extension study. J Affect Disord. 2010;126(3):358-65.

132. McQuade RD, Stock E, Marcus R, Jody D, Gharbia NA, Vanveggel S, et al. A comparison of weight change during treatment with olanzapine or aripiprazole: results from a randomized, double-blind study. J Clin Psychiatry. 2004;65 Suppl 18:47-56.

133. Meltzer HY, Bobo WV, Lee MA, Cola P, Jayathilake K. A randomized trial comparing clozapine and typical neuroleptic drugs in non-treatment-resistant schizophrenia. Psychiatry Res. 2010;177(3):286-93.

134. Meltzer HY, Cucchiaro J, Silva R, Ogasa M, Phillips D, Xu J, et al. Lurasidone in the treatment of schizophrenia: a randomized, double-blind, placebo- and olanzapine-controlled study. Am J Psychiatry. 2011;168(9):957-67.

135. Meltzer HY, Lindenmayer JP, Kwentus J, Share DB, Johnson R, Jayathilake K. A six month randomized controlled trial of long acting injectable risperidone 50 and 100mg in treatment resistant schizophrenia. Schizophr Res. 2014;154(1-3):14-22.

136. Merideth C, Cutler AJ, She F, Eriksson H. Efficacy and tolerability of extended release quetiapine fumarate monotherapy in the acute treatment of generalized anxiety disorder: a randomized, placebo controlled and active-controlled study. Int Clin Psychopharmacol. 2012;27(1):40-54.

137. Mintzer JE, Tune LE, Breder CD, Swanink R, Marcus RN, McQuade RD, et al. Aripiprazole for the treatment of psychoses in institutionalized patients with Alzheimer dementia: a multicenter, randomized, double-blind, placebo-controlled assessment of three fixed doses. Am J Geriatr Psychiatry. 2007;15(11):918-31.

138. Mitchell M, Riesenberg R, Bari MA, Marquez E, Kurtz D, Falk D, et al. A double-blind, randomized trial to evaluate the pharmacokinetics and tolerability of 30 or 40 mg/d oral olanzapine relative to 20 mg/d oral olanzapine in stable psychiatric subjects. Clin Ther. 2006;28(6):881-92.

139. Moller HJ, Johnson S, Mateva T, Brecher M, Svensson O, Miller F, et al. Evaluation of the feasibility of switching from immediate release quetiapine to extended release quetiapine fumarate in stable outpatients with schizophrenia. Int Clin Psychopharmacol. 2008;23(2):95-105.

140. Mortimer A, Martin S, Lôo H, Peuskens J. A double-blind, randomized comparative trial of amisulpride versus olanzapine for 6 months in the treatment of schizophrenia. Int Clin Psychopharmacol. 2004;19(2):63-9.

141. Naber D, Riedel M, Klimke A, Vorbach EU, Lambert M, Kuhn KU, et al. Randomized double blind comparison of olanzapine vs. clozapine on subjective well-being and clinical outcome in patients with schizophrenia. Acta Psychiatr Scand. 2005;111(2):106-15.

142. Naber D, Hansen K, Forray C, Baker RA, Sapin C, Beillat M, et al. Qualify: a randomized head-to-head study of aripiprazole once-monthly and paliperidone palmitate in the treatment of schizophrenia. Schizophr Res. 2015;168(1-2):498-504.

143. Nakamura M, Ogasa M, Guarino J, Phillips D, Severs J, Cucchiaro J, et al. Lurasidone in the treatment of acute schizophrenia: a double-blind, placebo-controlled trial. J Clin Psychiatry. 2009;70(6):829-36.

144. Nasrallah HA, Silva R, Phillips D, Cucchiaro J, Hsu J, Xu J, et al. Lurasidone for the treatment of acutely psychotic patients with schizophrenia: a 6-week, randomized, placebo-controlled study. J Psychiatr Res. 2013;47(5):670-7.

145. Nasrallah HA, Newcomer JW, Risinger R, Du Y, Zummo J, Bose A, et al. Effect of Aripiprazole Lauroxil on Metabolic and Endocrine Profiles and Related Safety Considerations Among Patients With Acute Schizophrenia. J Clin Psychiatry. 2016;77(11):1519-25.

146. Nasser AF, Henderson DC, Fava M, Fudala PJ, Twumasi-Ankrah P, Kouassi A, et al. Efficacy, Safety, and Tolerability of RBP-7000 Once-Monthly Risperidone for the Treatment of Acute Schizophrenia: An 8-Week, Randomized, Double-Blind, Placebo-Controlled, Multicenter Phase 3 Study. J Clin Psychopharmacol. 2016;36(2):130-40.

147. Newcomer JW, Campos JA, Marcus RN, Breder C, Berman RM, Kerselaers W, et al. A multicenter, randomized, double-blind study of the effects of aripiprazole in overweight subjects with schizophrenia or schizoaffective disorder switched from olanzapine. J Clin Psychiatry. 2008;69(7):1046-56.

148. Niufan G, Tohen M, Qiuqing A, Fude Y, Pope E, McElroy H, et al. Olanzapine versus lithium in the acute treatment of bipolar mania: a double-blind, randomized, controlled trial. J Affect Disord. 2008;105(1-3):101-8.

149. Olié J-P, Spina E, Murray S, Yang R. Ziprasidone and amisulpride effectively treat negative symptoms of schizophrenia: Results of a 12-week, double-blind study. International Clinical Psychopharmacology. 2006;21(3):143-51.

150. Ou JJ, Xu Y, Chen HH, Fan X, Gao K, Wang J, et al. Comparison of metabolic effects of ziprasidone versus olanzapine treatment in patients with first-episode schizophrenia. Psychopharmacology (Berl). 2013;225(3):627-35.

151. Pandina GJ, Lindenmayer JP, Lull J, Lim P, Gopal S, Herben V, et al. A randomized, placebo-controlled study to assess the efficacy and safety of 3 doses of paliperidone palmitate in adults with acutely exacerbated schizophrenia. Journal of Clinical Psychopharmacology. 2010;30(3):235-44.

152. Peluso MJ, Lewis SW, Barnes TR, Jones PB. Non-neurological and metabolic side effects in the Cost Utility of the Latest Antipsychotics in Schizophrenia Randomised Controlled Trial (CUtLASS-1). Schizophr Res. 2013;144(1-3):80-6.

153. Perez-Iglesias R, Ortiz-Garcia de la Foz V, Martinez Garcia O, Amado JA, Garcia-Unzueta MT, Ayesa-Arriola R, et al. Comparison of metabolic effects of aripiprazole, quetiapine and ziprasidone after 12 weeks of treatment in first treated episode of psychosis. Schizophr Res. 2014;159(1):90-4.

154. Peuskens J, Link CG. A comparison of quetiapine and chlorpromazine in the treatment of schizophrenia. Acta Psychiatr Scand. 1997;96(4):265-73.

155. Pigott TA, Carson WH, Saha AR, Torbeyns AF, Stock EG, Ingenito GG. Aripiprazole for the prevention of relapse in stabilized patients with chronic schizophrenia: a placebo-controlled 26-week study. J Clin Psychiatry. 2003;64(9):1048-56.

156. Potkin SG, Saha AR, Kujawa MJ, Carson WH, Ali M, Stock E, et al. Aripiprazole, an antipsychotic with a novel mechanism of action, and risperidone vs placebo in patients with schizophrenia and schizoaffective disorder. Arch Gen Psychiatry. 2003;60(7):681-90.

157. Potkin SG, Cohen M, Panagides J. Efficacy and tolerability of asenapine in acute schizophrenia: a placebo- and risperidone-controlled trial. J Clin Psychiatry. 2007;68(10):1492-500.

158. Potkin SG, Kimura T, Guarino J. A 6-week, double-blind, placebo- and haloperidol-controlled, phase II study of lurasidone in patients with acute schizophrenia. Therapeutic Advances in Psychopharmacology. 2015;5(6):322-31.

159. Quiroz JA, Yatham LN, Palumbo JM, Karcher K, Kushner S, Kusumakar V. Risperidone long-acting injectable monotherapy in the maintenance treatment of bipolar I disorder. Biol Psychiatry. 2010;68(2):156-62.

160. Ryckmans V, Kahn JP, Modell S, Werner C, McQuade RD, Kerselaers W, et al. Switching to aripiprazole in outpatients with schizophrenia experiencing insufficient efficacy and/or safety/tolerability issues with risperidone: a randomized, multicentre, open-label study. Pharmacopsychiatry. 2009;42(3):114-21.

161. Sacchetti E, Valsecchi P, Parrinello G. A randomized, flexible-dose, quasi-naturalistic comparison of quetiapine, risperidone, and olanzapine in the short-term treatment of schizophrenia: the QUERISOLA trial. Schizophr Res. 2008;98(1-3):55-65.

162. Sachs G, Sanchez R, Marcus R, Stock E, McQuade R, Carson W, et al. Aripiprazole in the treatment of acute manic or mixed episodes in patients with bipolar I disorder: a 3-week placebo-controlled study. J Psychopharmacol. 2006;20(4):536-46.

163. Sachs GS, Greenberg WM, Starace A, Lu K, Ruth A, Laszlovszky I, et al. Cariprazine in the treatment of acute mania in bipolar I disorder: A double-blind, placebo-controlled, Phase III trial. J Affect Disorders. 2015;174:296-302.

164. Savitz AJ, Xu H, Gopal S, Nuamah I, Ravenstijn P, Janik A, et al. Efficacy and Safety of Paliperidone Palmitate 3-Month Formulation for Patients with Schizophrenia: A Randomized, Multicenter, Double-Blind, Noninferiority Study. Int J Neuropsychopharmacol. 2016;19(7).

165. Schmidt ME, Kent JM, Daly E, Janssens L, Van Osselaer N, Hüsken G, et al. A double-blind, randomized, placebo-controlled study with JNJ-37822681, a novel, highly selective, fast dissociating D₂ receptor antagonist in the treatment of acute exacerbation of schizophrenia. European Neuropsychopharmacology. 2012;22(10):721-33.

166. Schneider LS, Tariot PN, Dagerman KS, Davis SM, Hsiao JK, Ismail MS, et al. Effectiveness of atypical antipsychotic drugs in patients with Alzheimer's disease. New England Journal of Medicine. 2006;355(15):1525-38.

167. Schoemaker J, Naber D, Vrijland P, Panagides J, Emsley R. Long-term assessment of Asenapine vs. Olanzapine in patients with schizophrenia or schizoaffective disorder. Pharmacopsychiatry. 2010;43(4):138-46.

168. Schreiner A, Niehaus D, Shuriquie NA, Aadamsoo K, Korcsog P, Salinas R, et al. Metabolic effects of paliperidone extended release versus oral olanzapine in patients with schizophrenia: a prospective, randomized, controlled trial. J Clin Psychopharmacol. 2012;32(4):449-57.

169. Schreiner A, Aadamsoo K, Altamura AC, Franco M, Gorwood P, Neznanov NG, et al. Paliperidone palmitate versus oral antipsychotics in recently diagnosed schizophrenia. Schizophr Res. 2015;169(1-3):393-9.

170. Schulz SC, Zanarini MC, Bateman A, Bohus M, Detke HC, Trzaskoma Q, et al. Olanzapine for the treatment of borderline personality disorder: variable dose 12-week randomised double-blind placebo-controlled study. Br J Psychiatry. 2008;193(6):485-92.

171. Sheehan DV, McElroy SL, Harnett-Sheehan K, Keck Jr PE, Janavs J, Rogers J, et al. Randomized, placebo-controlled trial of risperidone for acute treatment of bipolar anxiety. J Affect Disorders. 2009;115(3):376-85.

172. Sheehan DV, Harnett-Sheehan K, Hidalgo RB, Janavs J, McElroy SL, Amado D, et al. Randomized, placebo-controlled trial of quetiapine XR and divalproex ER monotherapies in the treatment of the anxious bipolar patient. J Affect Disorders. 2013;145(1):83-94.

173. Shen JH, Zhao Y, Rosenzweig-Lipson S, Popp D, Williams JB, Giller E, et al. A 6-week randomized, double-blind, placebo-controlled, comparator referenced trial of vabicaserin in acute schizophrenia. J Psychiatr Res. 2014;53:14-22.

174. Small JG, Hirsch SR, Arvanitis LA, Miller BG, Link CG. Quetiapine in patients with schizophrenia. A high- and low-dose double-blind comparison with placebo. Seroquel Study Group. Arch Gen Psychiatry. 1997;54(6):549-57.

175. Srivastava S, Wang PW, Hill SJ, Childers ME, Keller KL, Ketter TA. Pilot study of the efficacy of double-blind, placebo-controlled one-week olanzapine stabilization therapy in heterogeneous symptomatic bipolar disorder patients. J Psychiatr Res. 2012;46(7):920-6.

176. Streim JE, Porsteinsson AP, Breder CD, Swanink R, Marcus R, McQuade R, et al. A randomized, double-blind, placebo-controlled study of aripiprazole for the treatment of psychosis in nursing home patients with Alzheimer disease. Am J Geriatr Psychiatry. 2008;16(7):537-50.

177. Suppes T, Datto C, Minkwitz M, Nordenhem A, Walker C, Darko D. Effectiveness of the extended release formulation of quetiapine as monotherapy for the treatment of acute bipolar depression. J Affect Disord. 2010;121(1-2):106-15.

178. Szegedi A, Durgam S, Mackle M, Yun Yu S, Wu X, Mathews M, et al. Randomized, double-blind, placebo-controlled trial of asenapine maintenance therapy in adults with an acute manic or mixed episode associated with bipolar i disorder. American Journal of Psychiatry. 2018;175(1):71-9.

179. Thase ME, Macfadden W, Weisler RH, Chang W, Paulsson B, Khan A, et al. Efficacy of quetiapine monotherapy in bipolar I and II depression: a double-blind, placebo-controlled study (the BOLDER II study). J Clin Psychopharmacol. 2006;26(6):600-9.

180. Thase ME, Jonas A, Khan A, Bowden CL, Wu X, McQuade RD, et al. Aripiprazole monotherapy in nonpsychotic bipolar I depression: results of 2 randomized, placebo-controlled studies. J Clin Psychopharmacol. 2008;28(1):13-20.

181. Tohen M, Ketter TA, Zarate CA, Suppes T, Frye M, Altshuler L, et al. Olanzapine versus divalproex sodium for the treatment of acute mania and maintenance of remission: a 47-week study. Am J Psychiatry. 2003;160(7):1263-71.

182. Tohen M, Goldberg JF, Gonzalez-Pinto Arrillaga AM, Azorin JM, Vieta E, Hardy-Bayle MC, et al. A 12-week, double-blind comparison of olanzapine vs haloperidol in the treatment of acute mania. Arch Gen Psychiatry. 2003;60(12):1218-26.

183. Tohen M, Vieta E, Calabrese J, Ketter TA, Sachs G, Bowden C, et al. Efficacy of olanzapine and olanzapine-fluoxetine combination in the treatment of bipolar I depression. Arch Gen Psychiatry. 2003;60(11):1079-88.

184. Tohen M, Greil W, Calabrese JR, Sachs GS, Yatham LN, Oerlinghausen BM, et al. Olanzapine versus lithium in the maintenance treatment of bipolar disorder: a 12-month, randomized, double-blind, controlled clinical trial. Am J Psychiatry. 2005;162(7):1281-90.

185. Tohen M, Calabrese JR, Sachs GS, Banov MD, Detke HC, Risser R, et al. Randomized, placebo-controlled trial of olanzapine as maintenance therapy in patients with bipolar I disorder responding to acute treatment with olanzapine. Am J Psychiatry. 2006;163(2):247-56.

186. Tohen M, Vieta E, Goodwin GM, Sun B, Amsterdam JD, Banov M, et al. Olanzapine versus divalproex versus placebo in the treatment of mild to moderate mania: a randomized, 12-week, double-blind study. J Clin Psychiatry. 2008;69(11):1776-89.

187. Tohen M, McDonnell DP, Case M, Kanba S, Ha K, Fang YR, et al. Randomised, double-blind, placebo-controlled study of olanzapine in patients with bipolar I depression. Br J Psychiatry. 2012;201(5):376-82.

188. Tollefson GD, Beasley CM, Jr., Tran PV, Street JS, Krueger JA, Tamura RN, et al. Olanzapine versus haloperidol in the treatment of schizophrenia and schizoaffective and schizophreniform disorders: results of an international collaborative trial. Am J Psychiatry. 1997;154(4):457-65.

189. Vanelle JM, Douki S. A double-blind randomised comparative trial of amisulpride versus olanzapine for 2 months in the treatment of subjects with schizophrenia and comorbid depression. Eur Psychiatry. 2006;21(8):523-30.

190. Vieta E, Nuamah IF, Lim P, Yuen EC, Palumbo JM, Hough DW, et al. A randomized, placebo- and active-controlled study of paliperidone extended release for the treatment of acute manic and mixed episodes of bipolar I disorder. Bipolar Disord. 2010;12(3):230-43.

191. Wang G, McIntyre A, Earley WR, Raines S, Eriksson H. A randomized, double-blind study of the efficacy and tolerability of extended release quetiapine fumarate (quetiapine XR) monotherapy in patients with major depressive disorder. Psychopharmacology Bulletin. 2012;45(1):5-30.

192. Weiden PJ, Citrome L, Alva G, Brams M, Glick ID, Jackson R, et al. A trial evaluating gradual- or immediate-switch strategies from risperidone, olanzapine, or aripiprazole to iloperidone in patients with schizophrenia. Schizophr Res. 2014;153(1-3):160-8.

193. Weiden PJ, Manning R, Wolfgang CD, Ryan JM, Mancione L, Han G, et al. A Randomized Trial of Iloperidone for Prevention of Relapse in Schizophrenia: The REPRIEVE Study. CNS Drugs. 2016;30(8):735-47.

194. Weisler R, Joyce JM, McGill L, Lazarus A, Szamosi J, Eriksson H. Extended release quetiapine fumarate monotherapy for major depressive disorder: Results of a double-blind, randomized, placebo-controlled study. CNS Spectrums. 2009;14(6):299-313.

195. Weisler RH, Nolen WA, Neijber A, Hellqvist A, Paulsson B. Continuation of quetiapine versus switching to placebo or lithium for maintenance treatment of bipolar I disorder (Trial 144: A randomized controlled study). Journal of Clinical Psychiatry. 2011;72(11):1452-64.

196. Xiang YT, Wang CY, Ungvari GS, Kreyenbuhl JA, Chiu HF, Lai KY, et al. Weight changes and their associations with demographic and clinical characteristics in risperidone maintenance treatment for schizophrenia. Pharmacopsychiatry. 2011;44(4):135-41.

197. Yang J, Bahk W-M, Cho H-S, Jeon Y-W, Jon D-I, Jung H-Y, et al. Efficacy and tolerability of blonanserin in the patients with schizophrenia: A randomized, double-blind, risperidone-compared trial. Clinical Neuropharmacology. 2010;33(4):169-75.

198. Yatham LN, Vieta E, Earley W. Evaluation of cariprazine in the treatment of bipolar i and II depression: A randomized, double-blind, placebo-controlled, phase 2 trial. International Clinical Psychopharmacology. 2020:147-56.

199. Young AH, Oren DA, Lowy A, McQuade RD, Marcus RN, Carson WH, et al. Aripiprazole monotherapy in acute mania: 12-week randomised placebo- and haloperidol-controlled study. Br J Psychiatry. 2009;194(1):40-8.

200. Young AH, McElroy SL, Bauer M, Philips N, Chang W, Olausson B, et al. A Double-Blind, Placebo-Controlled Study of Quetiapine and Lithium Monotherapy in Adults in the Acute Phase of Bipolar Depression (EMBOLDEN I). Journal of Clinical Psychiatry. 2010;71(2):150-62.

201. Zanarini MC, Schulz SC, Detke HC, Tanaka Y, Zhao F, Lin D, et al. A dose comparison of olanzapine for the treatment of borderline personality disorder: a 12-week randomized, double-blind, placebo-controlled study. J Clin Psychiatry. 2011;72(10):1353-62.

202. Zhong KX, Sweitzer DE, Hamer RM, Lieberman JA. Comparison of quetiapine and risperidone in the treatment of schizophrenia: A randomized, double-blind, flexible-dose, 8-week study. J Clin Psychiatry. 2006;67(7):1093-103.

# Appendix 4: Excluded studies

## Appendix 4.1: Table of excluded studies after full text assessment

| **Author (year)** | **Reason for Exclusion** | **Reference** |
| --- | --- | --- |
| Ader 2008 | No 7% weight data | (203) |
| Albaugh 2011 | No 7% weight data | (204) |
| Altamura 2003 | No 7% weight data | (205) |
| Alvarez 2006 | No ITT analysis | (206) |
| Amore 2011 | No 7% weight data | (207) |
| Apiquian 2003 | No 7% weight data | (208) |
| Apiquian 2005 | No 7% weight data | (209) |
| Arango 2009 | No 7% weight data | (210) |
| Arato 2002 | No 7% weight data | (211) |
| Atmaca 2003 | No 7% weight data | (212) |
| Azorin 2006 | No 7% weight data | (213) |
| Ballon 2018 | No ITT analysis | (214) |
| Baptista 1997 | No 7% weight data | (215) |
| Baptista 2007 | No 7% weight data | (216) |
| Barnett 2002 | No 7% weight data | (217) |
| Baymiller 2002 | No 7% weight data | (218) |
| Baymiller 2003 | No 7% weight data | (219) |
| Beasley 1997 | No 7% weight data | (220) |
| Beasley 2003 | No 7% weight data | (221) |
| Berger 2008 | No 7% weight data | (222) |
| Bhowmick 2010 | No 7% weight data | (223) |
| Bingham 2018 | No 7% weight data | (224) |
| Bisol 2008 | No ITT analysis | (225) |
| Bitter 2004 | No 7% weight data | (226) |
| Bitter 2010 | No 7% weight data | (227) |
| Black 2014 | No 7% weight data | (228) |
| Bobo 2011a | No 7% weight data | (229) |
| Bobo 2011b | No 7% weight data | (230) |
| Bobo 2011c | No 7% weight data | (231) |
| Bogenschutz 2004 | No 7% weight data | (232) |
| Boidi 2007 | No 7% weight data | (233) |
| Bonaccorso 2015 | No 7% weight data | (234) |
| Bowden 2005 | No 7% weight data | (235) |
| Boyer 1999 | No 7% weight data | (236) |
| Bozzatello 2017 | No 7% weight data | (237) |
| Breier 2002 | No 7% weight data | (238) |
| Breier 2005 | No 7% weight data | (239) |
| Brunette 2011 | No 7% weight data | (240) |
| Buchanan 2005 | No 7% weight data | (241) |
| Bustillo 1996 | No 7% weight data | (242) |
| Butterfield 2001 | No 7% weight data | (243) |
| Canuso 2010 | No 7% weight data | (244) |
| Carey 2012 | No 7% weight data | (245) |
| Chaichan 2004 | No 7% weight data | (246) |
| Chan 2013 | No 7% weight data | (247) |
| Chaudhry 2007 | No 7% weight data | (248) |
| Chiu 2006 | No 7% weight data | (249) |
| Chowdhury 1999 | No 7% weight data | (250) |
| Citrome 2009 | No ITT analysis | (251) |
| Citrome 2015 | Posthoc / subgroup analysis | (252) |
| Citrome 2016 | No 7% weight data | (253) |
| Ciudad 2006 | No ITT analysis | (254) |
| Ciudad 2007 | Language | (255) |
| Cohen-Mansfield 1999 | No 7% weight data | (256) |
| Collins 1967 | No 7% weight data | (257) |
| Cookson 1986 | No 7% weight data | (258) |
| Cooper 2000 | No 7% weight data | (259) |
| Corazza 1996 | No 7% weight data | (260) |
| Correll 2020 | No 7% weight data | (261) |
| Covell 2004 | No 7% weight data | (262) |
| Covell 2012 | No 7% weight data | (263) |
| Crespo-Facorro 2006 | No 7% weight data | (264) |
| Crespo-Facorro 2011 | No 7% weight data | (265) |
| Crespo-Facorro 2012 | No 7% weight data | (266) |
| Crespo-Facorro 2013a | No 7% weight data | (267) |
| Crespo-Facorro 2013b | No 7% weight data | (268) |
| Crespo-Facorro 2014 | No 7% weight data | (269) |
| Csernansky 2002 | No 7% weight data | (270) |
| Czobor 2002 | No 7% weight data | (271) |
| Daly 2013 | Posthoc / subgroup analysis | (272) |
| Daniel 1996 | No 7% weight data | (273) |
| Daniel 1999 | No 7% weight data | (274) |
| Daurignac 2015 | No 7% weight data | (275) |
| de Arce Cordón 2012 | No 7% weight data | (276) |
| De Deyn 2004 | No 7% weight data | (277) |
| de Leon 2007 | No 7% weight data | (278) |
| De Lima 2005 | No 7% weight data | (279) |
| Deepak 2015 | No 7% weight data | (280) |
| Di Fiorino 2014 | No 7% weight data | (281) |
| Dossenbach 2004 | No 7% weight data | (282) |
| Downing 2014 | No 7% weight data | (283) |
| Dufresne 1993 | No 7% weight data | (284) |
| Durif 2004 | No 7% weight data | (285) |
| Earley 2019 | Study already included | (286) |
| Ehrlich 2012 | No 7% weight data | (287) |
| El-Mallakh 2012 | No ITT analysis | (288) |
| Emsley 2004 | No 7% weight data | (289) |
| Emsley 2005 | No 7% weight data | (290) |
| Endicott 2007 | No 7% weight data | (291) |
| Essock 2000 | No 7% weight data | (292) |
| Essock 2011 | No 7% weight data | (293) |
| Fava 2012 | No 7% weight data | (294) |
| Feng 2019 | No 7% weight data | (295) |
| Fleischhacker 2013a | No 7% weight data | (296) |
| Fleischhacker 2013b | No 7% weight data | (297) |
| Fong 2008 | No 7% weight data | (298) |
| Fountaine 2010 | No 7% weight data | (299) |
| Frank 2015 | No 7% weight data | (300) |
| Friedman 1999 | No 7% weight data | (301) |
| Fu 2014 | No 7% weight data | (302) |
| Fukushi 2020 | No 7% weight data | (303) |
| Gaebel 2010 | No 7% weight data | (304) |
| Gafoor 2010 | No 7% weight data | (305) |
| Ganguli 2008 | No 7% weight data | (306) |
| Gao 2018 | No 7% weight data | (307) |
| Gareri 2004 | No 7% weight data | (308) |
| Garriga 2017 | No 7% weight data | (309) |
| Gattaz 2004 | No 7% weight data | (310) |
| Geffen 2012 | No 7% weight data | (311) |
| Godleski 2003 | No 7% weight data | (312) |
| Godleski 2005 | No 7% weight data | (313) |
| Gomez-Revuelta 2018 | No 7% weight data | (314) |
| Gomez-Revuelta 2020 | No 7% weight data | (315) |
| Gureje 2003 | No 7% weight data | (316) |
| Hamilton 2009 | No 7% weight data | (317) |
| Hard 2017 | No 7% weight data | (318) |
| Hard 2019 | No 7% weight data | (319) |
| Hardy 2011 | No 7% weight data | (320) |
| Harvey 2019 | No 7% weight data | (321) |
| Harvey 2020 | No 7% weight data | (322) |
| Hatta 2009 | No 7% weight data | (323) |
| Hinze-Selch 2000 | No 7% weight data | (324) |
| Hirsch 2002 | No 7% weight data | (325) |
| Hirschfeld 2004 | No 7% weight data | (326) |
| Hough 2009 | No 7% weight data | (327) |
| Howanitz 1999 | No 7% weight data | (328) |
| Hu 2013 | No 7% weight data | (329) |
| Huang 2018 | No 7% weight data | (330) |
| Huttunen 1995 | No 7% weight data | (331) |
| Hwang 2001 | No 7% weight data | (332) |
| Hwang 2003 | No 7% weight data | (333) |
| Hwang 2015 | No 7% weight data | (334) |
| Ingole 2009 | No 7% weight data | (335) |
| Ionescu 2010 | No 7% weight data | (336) |
| Ishigooka 2015 | No 7% weight data | (337) |
| Jena 2019 | No 7% weight data | (338) |
| Johnsen 2010 | No 7% weight data | (339) |
| Johnsen 2020 | No 7% weight data | (340) |
| Jokinen 1984 | No 7% weight data | (341) |
| Jones 2006 | No 7% weight data | (342) |
| Jus 1974 | No 7% weight data | (343) |
| Kahn 2008 | No ITT analysis | (344) |
| Kane 2003 | No 7% weight data | (345) |
| Kane 2007 | No 7% weight data | (346) |
| Kane 2010 | No ITT analysis | (347) |
| Kane 2020 | No 7% weight data | (348) |
| Kasper 2003 | No 7% weight data | (349) |
| Keck 1998 | No 7% weight data | (350) |
| Keck 2003 | No 7% weight data | (351) |
| Keck 2009 | No ITT analysis | (352) |
| Keefe 2006 | No 7% weight data | (353) |
| Keefe 2004 | No 7% weight data | (354) |
| Kelly 2008 | Posthoc / subgroup analysis | (355) |
| Kennedy 2003 | Posthoc / subgroup analysis | (356) |
| Kennedy 2005 | No 7% weight data | (357) |
| Khanna 2005 | No 7% weight data | (358) |
| Kim 2009 | No 7% weight data | (359) |
| Kingstone 1970 | No 7% weight data | (360) |
| Kinon 2006 | No 7% weight data | (361) |
| Kinon 2010 | No 7% weight data | (362) |
| Kluge 2007 | No 7% weight data | (363) |
| Kluge 2009 | No 7% weight data | (364) |
| Kolotkin 2008 | No 7% weight data | (365) |
| Krakowski 2011 | No 7% weight data | (366) |
| Krystal 2011 | No 7% weight data | (367) |
| Kusumi 2012 | No 7% weight data | (368) |
| Kwon 2012 | No 7% weight data | (369) |
| Kwon 2015 | No 7% weight data | (370) |
| Lecrubier 2006 | No 7% weight data | (371) |
| Lee 2002 | No 7% weight data | (372) |
| Lee 2012 | No 7% weight data | (373) |
| Lencz 2010 | No 7% weight data | (374) |
| Li 2012 | No 7% weight data | (375) |
| Lieberman 2003 | No 7% weight data | (376) |
| Lieberman 2016 | No ITT analysis | (377) |
| Liebowitz 2010 | No 7% weight data | (378) |
| Lin 2010 | No 7% weight data | (379) |
| Lin 2013 | No 7% weight data | (380) |
| Lin 2017 | No 7% weight data | (381) |
| Lindenmayer 2007 | No 7% weight data | (382) |
| Lindenmayer 2011 | No 7% weight data | (383) |
| Littlewood 2015 | No 7% weight data | (384) |
| Liu 2014 | No 7% weight data | (385) |
| Liu 2016 | Language | (386) |
| Loebel 2013 | No ITT analysis | (387) |
| Loebl 2008 | No 7% weight data | (388) |
| Lofwall 2014 | No 7% weight data | (389) |
| Lohoff 2010 | No 7% weight data | (390) |
| MacFadden 2010 | No 7% weight data | (391) |
| Maguire 2004 | No 7% weight data | (392) |
| Maina 2008 | No 7% weight data | (393) |
| Maitra 2020 | No 7% weight data | (394) |
| Mamo 2004 | No 7% weight data | (395) |
| Marni 2019 | No 7% weight data | (396) |
| Marra 2002 | No 7% weight data | (397) |
| Martin 2020 | No 7% weight data | (398) |
| Mazza 2008 | No 7% weight data | (399) |
| McDougle 1998 | No 7% weight data | (400) |
| McEvoy 2007 | No ITT analysis | (401) |
| McEvoy 2013 | No 7% weight data | (402) |
| McEvoy 2014 | No 7% weight data | (403) |
| McGlashan 2006 | No 7% weight data | (404) |
| McIntyre 2011 | No ITT analysis | (405) |
| McLaren 1992 | No 7% weight data | (406) |
| Mearin 2004 | No 7% weight data | (407) |
| Meltzer 2008 | No 7% weight data | (408) |
| Meltzer 2015 | No 7% weight data | (409) |
| Miceli 2000 | No 7% weight data | (410) |
| Min 1993 | No 7% weight data | (411) |
| Moretti 2005 | No 7% weight data | (412) |
| Moretti 2004 | No 7% weight data | (413) |
| Mosolov 2011 | No 7% weight data | (414) |
| Mousavi 2013 | No 7% weight data | (415) |
| Murasaki 2018 | No 7% weight data | (416) |
| Naber 2013 | No 7% weight data | (417) |
| Nagesh 2017 | No 7% weight data | (418) |
| Nasrallah 2010 | No 7% weight data | (419) |
| Navari 2016 | No 7% weight data | (420) |
| Nemeth 2017 | No 7% weight data | (421) |
| Newcomer 2009 | No 7% weight data | (422) |
| Nistico 1974 | No 7% weight data | (423) |
| Noordsy 2017 | No 7% weight data | (424) |
| Ohlmeier 2007 | No 7% weight data | (425) |
| Ogasa 2013 | No 7% weight data | (426) |
| Okugawa 2009 | No 7% weight data | (427) |
| Ondo 2002 | No 7% weight data | (428) |
| Ozguven 2011 | No 7% weight data | (429) |
| Pae 2007 | No 7% weight data | (430) |
| Pae 2009 | No 7% weight data | (431) |
| Paleacu 2008 | No 7% weight data | (432) |
| Papakostas 2012 | No 7% weight data | (433) |
| Papakostas 2015 | No 7% weight data | (434) |
| Park 2013 | No 7% weight data | (435) |
| Patil 2007 | No 7% weight data | (436) |
| Patkar 2012 | No 7% weight data | (437) |
| Perez-Iglesias 2007 | No ITT analysis | (438) |
| Perez-Iglesias 2008a | No 7% weight data | (439) |
| Perez-Iglesias 2008b | No ITT analysis | (440) |
| Perez-Iglesias 2009 | No 7% weight data | (441) |
| Perez-Iglesias 2014 | No ITT analysis | (442) |
| Perkins 2008 | No 7% weight data | (443) |
| Perlis 2006 | No 7% weight data | (444) |
| Peuskens 1999 | No 7% weight data | (445) |
| Peuskens 2007 | Study already included | (446) |
| Pivac 2004 | No 7% weight data | (447) |
| Popovic 2007 | No 7% weight data | (448) |
| Potkin 2011 | No 7% weight data | (449) |
| Potkin 2019 | No 7% weight data | (450) |
| Pu 2019 | No 7% weight data | (451) |
| Purdon 2001 | No 7% weight data | (452) |
| Quednow 2006 | No 7% weight data | (453) |
| Raison 2018 | No 7% weight data | (454) |
| Ramasubbu 2016 | No 7% weight data | (455) |
| Ramerman 2019 | No 7% weight data | (456) |
| Ramsey 2014 | No 7% weight data | (457) |
| Raoufinia 2017 | No 7% weight data | (458) |
| Ravenstijn 2016 | No 7% weight data | (459) |
| Reeves 2008 | No 7% weight data | (460) |
| Revicki 2003 | No 7% weight data | (461) |
| Rezayat 2014 | No 7% weight data | (462) |
| Riesenberg 2012 | No 7% weight data | (463) |
| Ritchie 2003 | No 7% weight data | (464) |
| Ritchie 2006 | No 7% weight data | (465) |
| Robinson 2006 | No 7% weight data | (466) |
| Roerig 2005 | No 7% weight data | (467) |
| Roerig 2008 | No 7% weight data | (468) |
| Rosenheck 2003 | No 7% weight data | (469) |
| Rothschild 2004 | No 7% weight data | (470) |
| Rui 2014 | No 7% weight data | (471) |
| Ryan 2004 | No 7% weight data | (472) |
| Sacchetti 2004 | No 7% weight data | (473) |
| Sacchetti 2009 | No 7% weight data | (474) |
| Sacher 2008 | No 7% weight data | (475) |
| Saddichha 2007 | No 7% weight data | (476) |
| Saddichha 2008 | No 7% weight data | (477) |
| Safa 2008 | No 7% weight data | (478) |
| Sajatovic 2001 | No 7% weight data | (479) |
| Sajeev Kumar 2017 | No 7% weight data | (480) |
| San 2012 | No 7% weight data | (481) |
| Sanz-Fuentenebro 2013 | No 7% weight data | (482) |
| Sathirakul 2003 | No 7% weight data | (483) |
| Schoemaker 2012 | No 7% weight data | (484) |
| Schooler 2005 | No 7% weight data | (485) |
| Schooler 2016 | No 7% weight data | (486) |
| Sechter 2002 | No ITT analysis | (487) |
| Sevy 2011 | No 7% weight data | (488) |
| Shafti 2016 | No 7% weight data | (489) |
| Shafti 2019 | No 7% weight data | (490) |
| Sherwood Brown 2014 | No 7% weight data | (491) |
| Shoja Shafti 2014 | No 7% weight data | (492) |
| Shoja Shafti 2015a | No 7% weight data | (493) |
| Shoja Shafti 2015b | No 7% weight data | (494) |
| Silva de Lima 2005 | No 7% weight data | (495) |
| Simpson 2004 | No 7% weight data | (496) |
| Simpson 2005 | No 7% weight data | (497) |
| Singh 2016 | No 7% weight data | (498) |
| Smeraldi 1998 | No 7% weight data | (499) |
| Smith 2009 | No 7% weight data | (500) |
| Smith 2010 | No 7% weight data | (501) |
| Smith 2012 | No 7% weight data | (502) |
| Sowell 2002 | No 7% weight data | (503) |
| Sowell 2003 | No 7% weight data | (504) |
| Spivak 1999 | No 7% weight data | (505) |
| Stone 2012 | No 7% weight data | (506) |
| Su 2021 | No 7% weight data | (507) |
| Sumiyoshi 2003 | No 7% weight data | (508) |
| Suppes 2014 | No 7% weight data | (509) |
| Suresh Kumar 2016 | No 7% weight data | (510) |
| Swadi 2010 | No 7% weight data | (511) |
| Swartz 2018 | No 7% weight data | (512) |
| Takekita 2013 | No 7% weight data | (513) |
| Tandon 2016 | No 7% weight data | (514) |
| Tapp 2015 | No 7% weight data | (515) |
| Teff 2013 | No 7% weight data | (516) |
| Thase 2007 | No 7% weight data | (517) |
| Tohen 1999 | No 7% weight data | (518) |
| Tohen 2002 | No 7% weight data | (519) |
| Tohen 2000 | No 7% weight data | (520) |
| Tollefson 2001 | No 7% weight data | (521) |
| Tran 1997 | No 7% weight data | (522) |
| Tran 1999 | No 7% weight data | (523) |
| Tunis 2006 | No 7% weight data | (524) |
| Tybura 2014 | No 7% weight data | (525) |
| Tyrer 2009 | No 7% weight data | (526) |
| Tzimos 2008 | No 7% weight data | (527) |
| van Bruggen 2003 | No ITT analysis | (528) |
| Vazquez-Bourgon 2018 | No 7% weight data | (529) |
| Vazquez-Bourgon 2020 | No 7% weight data | (530) |
| Verhey 2006 | No 7% weight data | (531) |
| Vidarsdottir 2010 | No 7% weight data | (532) |
| Vieta 2005 | No 7% weight data | (533) |
| Volavka 2002 | No 7% weight data | (534) |
| Voruganti 2007 | No 7% weight data | (535) |
| Wampers 2012 | No 7% weight data | (536) |
| Wang 2006 | No 7% weight data | (537) |
| Wang 2010 | No 7% weight data | (538) |
| Wang 2014 | No 7% weight data | (539) |
| Wang 2017 | No 7% weight data | (540) |
| Weiden 2020a | No 7% weight data | (541) |
| Weiden 2020b | No 7% weight data | (542) |
| Werapongset 1998 | No 7% weight data | (543) |
| Wilner 2000 | No 7% weight data | (544) |
| Woods 2003 | No 7% weight data | (545) |
| Wu 2007 | No 7% weight data | (546) |
| Xu 2015 | No 7% weight data | (547) |
| Xue 2018 | No 7% weight data | (548) |
| Yatham 2007 | No 7% weight data | (549) |
| Yung 2011 | No 7% weight data | (550) |
| Zajecka 2002 | No 7% weight data | (551) |
| Zanardi 2006 | No 7% weight data | (552) |
| Zanarini 2001 | No 7% weight data | (553) |
| Zanarini 2004 | No 7% weight data | (554) |
| Zarcone 2001 | No 7% weight data | (555) |
| Zhang 2012 | No 7% weight data | (556) |
| Zimbroff 2007 | No 7% weight data | (557) |

## Appendix 4.2: References of excluded studies

203. Ader M, Garvey WT, Phillips LS, Nemeroff CB, Gharabawi G, Mahmoud R, et al. Ethnic heterogeneity in glucoregulatory function during treatment with atypical antipsychotics in patients with schizophrenia. J Psychiatr Res. 2008;42(13):1076-85.

204. Albaugh VL, Singareddy R, Mauger D, Lynch CJ. A double blind, placebo-controlled, randomized crossover study of the acute metabolic effects of olanzapine in healthy volunteers. PLoS ONE. 2011;6(8):e22662.

205. Altamura AC, Salvadori D, Madaro D, Santini A, Mundo E. Efficacy and tolerability of quetiapine in the treatment of bipolar disorder: Preliminary evidence from a 12-month open-label study. J Affect Disorders. 2003;76(1-3):267-71.

206. Alvarez E, Ciudad A, Olivares JM, Bousoño M, Gómez JC. A randomized, 1-year follow-up study of olanzapine and risperidone in the treatment of negative symptoms in outpatients with schizophrenia. J Clin Psychopharmacol. 2006;26(3):238-49.

207. Amore M, Bertelli M, Villani D, Tamborini S, Rossi M. Olanzapine vs. risperidone in treating aggressive behaviours in adults with intellectual disability: A single blind study. Journal of Intellectual Disability Research. 2011;55(2):210-8.

208. Apiquian R, Fresan A, Herrera K, Ulloa RE, Loyzaga C, De La Fuente-Sandoval C, et al. Minimum effective doses of haloperidol for the treatment of first psychotic episode: A comparative study with risperidone and olanzapine. Int J Neuropsychoph. 2003;6(4):403-8.

209. Apiquian R, Fresan A, Ulloa RE, de la Fuente-Sandoval C, Herrera-Estrella M, Vazquez A, et al. Amoxapine as an atypical antipsychotic: a comparative study vs risperidone. Neuropsychopharmacology. 2005;30(12):2236-44.

210. Arango C, Robles O, Parellada M, Fraguas D, Ruiz-Sancho A, Medina O, et al. Olanzapine compared to quetiapine in adolescents with a first psychotic episode. Eur Child Adolesc Psychiatry. 2009;18(7):418-28.

211. Arato M, O'Connor R, Meltzer HY. A 1-year, double-blind, placebo-controlled trial of ziprasidone 40, 80 and 160 mg/day in chronic schizophrenia: the Ziprasidone Extended Use in Schizophrenia (ZEUS) study. Int Clin Psychopharmacol. 2002;17(5):207-15.

212. Atmaca M, Kuloglu M, Tezcan E, Ustundag B. Serum leptin and triglyceride levels in patients on treatment with atypical antipsychotics. Journal of Clinical Psychiatry. 2003;64(5):598-604.

213. Azorin JM, Strub N, Loft H. A double-blind, controlled study of sertindole versus risperidone in the treatment of moderate-to-severe schizophrenia. International Clinical Psychopharmacology. 2006;21(1):49-56.

214. Ballon JS, Pajvani UB, Mayer LES, Freyberg Z, Freyberg R, Contreras I, et al. Pathophysiology of drug induced weight and metabolic effects: findings from an RCT in healthy volunteers treated with olanzapine, iloperidone, or placebo. Journal of Psychopharmacology. 2018;32(5):533-40.

215. Baptista T, Molina MG, Martinez JL, De Quijada M, Calanche De Cuesta I, Acosta A, et al. Effects of the antipsychotic drug sulpiride on reproductive hormones in healthy premenopausal women: Relationship with body weight regulation. Pharmacopsychiatry. 1997;30(6):256-62.

216. Baptista T, Martinez M, Lacruz A, Arellano A, Mendoza S, Beaulieu S, et al. Insulin resistance index and counter-regulatory factors during olanzapine or risperidone administration in subjects with schizophrenia. Schizophr Res. 2007;89(1-3):350-2.

217. Barnett SD, Kramer ML, Casat CD, Connor KM, Davidson JRT. Efficacy of olanzapine in social anxiety disorder: A pilot study. Journal of Psychopharmacology. 2002;16(4):365-8.

218. Baymiller SP, Ball P, McMahon RP, Buchanan RW. Weight and blood pressure change during clozapine treatment. Clin Neuropharmacol. 2002;25(4):202-6.

219. Baymiller SP, Ball P, McMahon RP, Buchanan RW. Serum glucose and lipid changes during the course of clozapine treatment: the effect of concurrent beta-adrenergic antagonist treatment. Schizophr Res. 2003;59(1):49-57.

220. Beasley CM, Jr., Hamilton SH, Crawford AM, Dellva MA, Tollefson GD, Tran PV, et al. Olanzapine versus haloperidol: acute phase results of the international double-blind olanzapine trial. Eur Neuropsychopharmacol. 1997;7(2):125-37.

221. Beasley Jr CM, Sutton VK, Hamilton SH, Walker DJ, Dossenbach M, Taylor CC, et al. A Double-Blind, Randomized, Placebo-Controlled Trial of Olanzapine in the Prevention of Psychotic Relapse. Journal of Clinical Psychopharmacology. 2003;23(6):582-94.

222. Berger GE, Proffitt TM, McConchie M, Kerr M, Markulev C, Yuen HP, et al. Dosing quetiapine in drug-naive first-episode psychosis: A controlled, double-blind, randomized, single-center study investigating efficacy, tolerability, and safety of 200 mg/day vs. 400 mg/day of quetiapine fumarate in 141 patients aged 15 to 25 years. Journal of Clinical Psychiatry. 2008;69(11):1702-14.

223. Bhowmick S, Hazra A, Ghosh M. Amisulpride versus olanzapine in the treatment of schizophrenia in Indian patients: randomized controlled trial. Aust N Z J Psychiatry. 2010;44(3):237-42.

224. Bingham KS, Meyers BS, Mulsant BH, Rothschild AJ, Whyte EM, Banerjee S, et al. Stabilization treatment of remitted psychotic depression: The STOP-PD study. Acta Psychiatrica Scandinavica. 2018;138(3):267-73.

225. Bisol LW, Brunstein MG, Ottoni GL, Ramos FLP, Borba DL, Daltio CS, et al. Is flunarizine a long-acting oral atypical antipsychotic? A randomized clinical trial versus haloperidol for the treatment of schizophrenia. Journal of Clinical Psychiatry. 2008;69(10):1572-9.

226. Bitter I, Dossenbach MRK, Brook S, Feldman PD, Metcalfe S, Gagiano CA, et al. Olanzapine versus clozapine in treatment-resistant or treatment-intolerant schizophrenia. Progress in Neuro-Psychopharmacology and Biological Psychiatry. 2004;28(1):173-80.

227. Bitter I, Treuer T, Dilbaz N, Oyffe I, Ciorabai EM, Gonzalez SL, et al. Patients' preference for olanzapine orodispersible tablet compared with conventional oral tablet in a multinational, randomized, crossover study. World J Biol Psychiatry. 2010;11(7):894-903.

228. Black DW, Zanarini MC, Romine A, Shaw M, Allen J, Schulz SC. Comparison of low and moderate dosages of extended-release quetiapine in borderline personality disorder: A randomized, double-blind, placebo-controlled trial. American Journal of Psychiatry. 2014;171(11):1174-82.

229. Bobo WV, Bonaccorso S, Jayathilake K, Meltzer HY. Prediction of long-term metabolic effects of olanzapine and risperidone treatment from baseline body mass index in schizophrenia and bipolar disorder. Psychiatry Research. 2011;189(2):200-7.

230. Bobo WV, Epstein RA, Lynch A, Patton TD, Bossaller NA, Shelton RC. A Randomized open comparison of long-acting injectable risperidone and treatment as usual for prevention of relapse, rehospitalization, and urgent care referral in community-treated patients with rapid cycling bipolar disorder. Clinical Neuropharmacology. 2011;34(6):224-33.

231. Bobo WV, Epstein RA, Jr., Shelton RC. Effects of orally disintegrating vs regular olanzapine tablets on body weight, eating behavior, glycemic and lipid indices, and gastrointestinal hormones: a randomized, open comparison in outpatients with bipolar depression. Ann Clin Psychiatry. 2011;23(3):193-201.

232. Bogenschutz MP, George Nurnberg H. Olanzapine versus placebo in the treatment of borderline personality disorder. J Clin Psychiatry. 2004;65(1):104-9.

233. Boidi G, Ferro M. Rapid dose initiation of quetiapine for the treatment of acute schizophrenia and schizoaffective disorder: A randomised, multicentre, parallel-group, open study. Human Psychopharmacology. 2007;22(5):299-306.

234. Bonaccorso S, Sodhi M, Li J, Bobo WV, Chen Y, Tumuklu M, et al. The brain-derived neurotrophic factor (BDNF) Val66Met polymorphism is associated with increased body mass index and insulin resistance measures in bipolar disorder and schizophrenia. Bipolar Disorders. 2015;17(5):528-35.

235. Bowden CL, Grunze H, Mullen J, Brecher M, Paulsson B, Jones M, et al. A randomized, double-blind, placebo-controlled efficacy and safety study of quetiapine or lithium as monotherapy for mania in bipolar disorder. J Clin Psychiatry. 2005;66(1):111-21.

236. Boyer P, Lecrubier Y, Stalla-Bourdillon A, Fleurot O. Amisulpride versus amineptine and placebo for the treatment of dysthymia. Neuropsychobiology. 1999;39(1):25-32.

237. Bozzatello P, Rocca P, Uscinska M, Bellino S. Efficacy and Tolerability of Asenapine Compared with Olanzapine in Borderline Personality Disorder: An Open-Label Randomized Controlled Trial. CNS Drugs. 2017;31(9):809-19.

238. Breier A, Sutton VK, Feldman PD, Kadam DL, Ferchland I, Wright P, et al. Olanzapine in the treatment of dopamimetic-induced psychosis in patients with Parkinson's disease. Biol Psychiatry. 2002;52(5):438-45.

239. Breier A, Berg PH, Thakore JH, Naber D, Gattaz WF, Cavazzoni P, et al. Olanzapine versus ziprasidone: Results of a 23-week double-blind study in patients with schizophrenia. American Journal of Psychiatry. 2005;162(10):1879-87.

240. Brunette MF, Dawson R, O'Keefe CD, Narasimhan M, Noordsy DL, Wojcik J, et al. A randomized trial of clozapine versus other antipsychotics for cannabis use disorder in patients with schizophrenia. Journal of Dual Diagnosis. 2011;7(1-2):50-63.

241. Buchanan RW, Ball MP, Weiner E, Kirkpatrick B, Gold JM, McMahon RP, et al. Olanzapine treatment of residual positive and negative symptoms. Am J Psychiatry. 2005;162(1):124-9.

242. Bustillo JR, Buchanan RW, Irish D, Breier A. Differential effect of clozapine on weight: a controlled study. Am J Psychiatry. 1996;153(6):817-9.

243. Butterfield MI, Becker ME, Connor KM, Sutherland S, Churchill LE, Davidson JRT. Olanzapine in the treatment of post-traumatic stress disorder: A pilot study. International Clinical Psychopharmacology. 2001;16(4):197-203.

244. Canuso CM, Grinspan A, Kalali A, Damaraju CV, Merriman U, Alphs L, et al. Medication satisfaction in schizophrenia: A blinded-initiation study of paliperidone extended release in patients suboptimally responsive to risperidone. International Clinical Psychopharmacology. 2010;25(3):155-64.

245. Carey P, Suliman S, Ganesan K, Seedat S, Stein DJ. Olanzapine monotherapy in posttraumatic stress disorder: efficacy in a randomized, double-blind, placebo-controlled study. Hum Psychopharmacol. 2012;27(4):386-91.

246. Chaichan W. Olanzapine plus fluvoxamine and olanzapine alone for the treatment of an acute exacerbation of schizophrenia. Psychiat Clin Neuros. 2004;58(4):364-8.

247. Chan HY, Lin AS, Chen KP, Cheng JS, Chen YY, Tsai CJ. An open-label, randomized, controlled trial of zotepine and risperidone for acutely ill, hospitalized, schizophrenic patients with symptoms of agitation. Journal of Clinical Psychopharmacology. 2013;33(6):747-52.

248. Chaudhry IB, Husain N, Khan S, Badshah S, Deakin B, Kapur S. Amoxapine as an antipsychotic: comparative study versus haloperidol. J Clin Psychopharmacol. 2007;27(6):575-81.

249. Chiu CC, Chen KP, Liu HC, Lu ML. The early effect of olanzapine and risperidone on insulin secretion in atypical-naive schizophrenic patients. Journal of Clinical Psychopharmacology. 2006;26(5):504-7.

250. Chowdhury AN, Mukherjee A, Ghosh K, Chowdhury S, Das Sen K. Horizon of a new hope: Recovery of schizophrenia in India. International Medical Journal. 1999;6(3):181-5.

251. Citrome L, Stauffer VL, Chen L, Kinon BJ, Kurtz DL, Jacobson JG, et al. Olanzapine plasma concentrations after treatment with 10, 20, and 40 mg/d in patients with schizophrenia: An analysis of correlations with efficacy, weight gain, and prolactin concentration. Journal of Clinical Psychopharmacology. 2009;29(3):278-83.

252. Citrome L, Weiden PJ, Alva G, Glick ID, Jackson R, Mattingly G, et al. Switching to iloperidone: An omnibus of clinically relevant observations from a 12-week, open-label, randomized clinical trial in 500 persons with schizophrenia. Clin Schizophr Relat Psychoses. 2015;8(4):183-95.

253. Citrome L, Ota A, Nagamizu K, Perry P, Weiller E, Baker RA. The effect of brexpiprazole (OPC-34712) and aripiprazole in adult patients with acute schizophrenia: results from a randomized, exploratory study. Int Clin Psychopharmacol. 2016;31(4):192-201.

254. Ciudad A, Olivares JM, Bousono M, Gomez JC, Alvarez E. Improvement in social functioning in outpatients with schizophrenia with prominent negative symptoms treated with olanzapine or risperidone in a 1 year randomized, open-label trial. Progress in Neuro-Psychopharmacology and Biological Psychiatry. 2006;30(8):1515-22.

255. Ciudad A, Alvarez E, Bousoño M, Olivares JM, Gómez JC. [Safety and tolerability of olanzapine versus risperidone: a one-year randomized study in outpatients with schizophrenia with prominent negative symptoms]. Actas Esp Psiquiatr. 2007;35(2):105-14.

256. Cohen-Mansfield J, Lipson S, Werner P, Billig N, Taylor L, Woosley R. Withdrawal of haloperidol, thioridazine, and lorazepam in the nursing home: A controlled, double-blind study. Archives of Internal Medicine. 1999;159(15):1733-40.

257. Collins AD, Dundas J. A double-blind trial of amitriptyline/perphenazine, perphenazine and placebo in chronic withdrawn inert schizophrenics. Br J Psychiatry. 1967;113(505):1425-9.

258. Cookson JC, Kennedy NM, Gribbon D. Weight gain and prolactin levels in patients on long-term antipsychotic medication: a double-blind comparative trial of haloperidol decanoate and fluphenazine decanoate. Int Clin Psychopharmacol. 1986;1 Suppl 1:41-51.

259. Cooper SJ, Butler A, Tweed J, Welch C, Raniwalla J. Zotepine in the prevention of recurrence: A randomised, double-blind, placebo-controlled study for chronic schizophrenia. Psychopharmacology. 2000;150(3):237-43.

260. Corazza GR, Biagi F, Albano O, Bianchi Porro G, Cheli R, Mazzacca G, et al. Levosulpiride in functional dyspepsia: A multicentric, double-blind, controlled trial. Italian Journal of Gastroenterology. 1996;28(6):317-23.

261. Correll CU, Litman RE, Filts Y, Llaudo J, Naber D, Torres F, et al. Efficacy and safety of once-monthly Risperidone ISM<sup></sup> in schizophrenic patients with an acute exacerbation. npj Schizophrenia. 2020;6(1).

262. Covell NH, Weissman EM, Essock SM. Weight gain with clozapine compared to first generation antipsychotic medications. Schizophr Bull. 2004;30(2):229-40.

263. Covell NH, McEvoy JP, Schooler NR, Stroup TS, Jackson CT, Rojas IA, et al. Effectiveness of switching from long-acting injectable fluphenazine or haloperidol decanoate to long-acting injectable risperidone microspheres: an open-label, randomized controlled trial. J Clin Psychiatry. 2012;73(5):669-75.

264. Crespo-Facorro B, Pérez-Iglesias R, Ramirez-Bonilla M, Martínez-García O, Llorca J, Luis Vázquez-Barquero J. A practical clinical trial comparing haloperidol, risperidone, and olanzapine for the acute treatment of first-episode nonaffective psychosis. J Clin Psychiatry. 2006;67(10):1511-21.

265. Crespo-Facorro B, Perez-Iglesias R, Mata I, Ramirez-Bonilla M, Martinez-Garcia O, Pardo-Garcia G, et al. Effectiveness of haloperidol, risperidone and olanzapine in the treatment of first-episode non-affective psychosis: Results of a randomized, flexible-dose, open-label 1-year follow-up comparison. Journal of Psychopharmacology. 2011;25(6):744-54.

266. Crespo-Facorro B, Perez-Iglesias R, Mata I, Martinez-Garcia O, Ortiz V, Pelayo-Teran JM, et al. Long-term (3-year) effectiveness of haloperidol, risperidone and olanzapine: Results of a randomized, flexible-dose, open-label comparison in first-episode nonaffective psychosis. Psychopharmacology. 2012;219(1):225-33.

267. Crespo-Facorro B, Ortiz-Garcia de la Foz V, Mata I, Ayesa-Arriola R, Suarez-Pinilla P, Valdizan EM, et al. Aripiprazole, Ziprasidone and Quetiapine in the treatment of first-episode nonaffective psychosis: A 12-week randomized, flexible-dose, open-label trial. Schizophr Res. 2013;147(2-3):375-82.

268. Crespo-Facorro B, Perez-Iglesias R, Mata I, Ortiz-Garcia De La Foz V, Martinez-Garcia O, Valdizan EM, et al. Aripiprazole, ziprasidone, and quetiapine in the treatment of first-episode nonaffective psychosis: Results of a 6-week, randomized, flexible-dose, open-label comparison. Journal of Clinical Psychopharmacology. 2013;33(2):215-20.

269. Crespo-Facorro B, Ortiz-Garcia De La Foz V, Mata I, Ayesa-Arriola R, Suarez-Pinilla P, Valdizan EM, et al. Treatment of first-episode non-affective psychosis: A randomized comparison of aripiprazole, quetiapine and ziprasidone over 1 year. Psychopharmacology. 2014;231(2):357-66.

270. Csernansky JG, Mahmoud R, Brenner R, Risperidone USASG. A comparison of risperidone and haloperidol for the prevention of relapse in patients with schizophrenia. N Engl J Med. 2002;346(1):16-22.

271. Czobor P, Volavka J, Sheitman B, Lindenmayer JP, Citrome L, McEvoy J, et al. Antipsychotic-induced weight gain and therapeutic response: a differential association. J Clin Psychopharmacol. 2002;22(3):244-51.

272. Daly EJ, Kent JM, Janssens L, Newcomer JW, Husken G, Boer PD, et al. Metabolic and body mass parameters after treatment with JNJ-37822681, a novel fast-dissociating D2 receptor antagonist, vs olanzapine in patients with schizophrenia. Annals of Clinical Psychiatry. 2013;25(3):173-83.

273. Daniel DG, Goldberg TE, Weinberger DR, Kleinman JE, Pickar D, Lubick LJ, et al. Different side effect profiles of risperidone and clozapine in 20 outpatients with schizophrenia or schizoaffective disorder: a pilot study. Am J Psychiatry. 1996;153(3):417-9.

274. Daniel DG, Zimbroff DL, Potkin SG, Reeves KR, Harrigan EP, Lakshminarayanan M. Ziprasidone 80 mg/day and 160 mg/day in the acute exacerbation of schizophrenia and schizoaffective disorder: a 6-week placebo-controlled trial. Ziprasidone Study Group. Neuropsychopharmacology. 1999;20(5):491-505.

275. Daurignac E, Leonard KE, Dubovsky SL. Increased lean body mass as an early indicator of olanzapine-induced weight gain in healthy men. Int Clin Psychopharmacol. 2015;30(1):23-8.

276. de Arce Cordon R, Eding E, Marques-Teixeira J, Milanova V, Rancans E, Schreiner A. Descriptive analyses of the aripiprazole arm in the risperidone long-acting injectable versus quetiapine relapse prevention trial (ConstaTRE). Eur Arch Psychiatry Clin Neurosci. 2012;262(2):139-49.

277. De Deyn PP, Carrasco MM, Deberdt W, Jeandel C, Hay DP, Feldman PD, et al. Olanzapine versus placebo in the treatment of psychosis with or without associated behavioral disturbances in patients with Alzheimer's disease. Int J Geriatr Psychiatry. 2004;19(2):115-26.

278. de Leon J, Diaz FJ, Josiassen RC, Cooper TB, Simpson GM. Weight gain during a double-blind multidosage clozapine study. J Clin Psychopharmacol. 2007;27(1):22-7.

279. De Lima MS, De Jesus Mari J, Breier A, Costa AM, De Sena EP, Hotopf M. Quality of life in schizophrenia: A multicenter, randomized, naturalistic, controlled trial comparing olanzapine to first-generation antipsychotics. Journal of Clinical Psychiatry. 2005;66(7):831-8.

280. Deepak TS, Raveesh BN, Parashivamurthy BM, Narendra Kumar MS, Majgi SM, Nagesh HN. Clinical assessment of weight gain with atypical antipsychotics - Blonanserin vs amisulpride. Journal of Clinical and Diagnostic Research. 2015;9(6):FC07-FC10.

281. Di Fiorino M, Montagnani G, Trespi G, Kasper S. Extended-release quetiapine fumarate (quetiapine XR) versus risperidone in the treatment of depressive symptoms in patients with schizoaffective disorder or schizophrenia: A randomized, open-label, parallel-group, flexible-dose study. International Clinical Psychopharmacology. 2014;29(3):166-76.

282. Dossenbach MR, Folnegovic-Smalc V, Hotujac L, Uglesic B, Tollefson GD, Grundy SL, et al. Double-blind, randomized comparison of olanzapine versus fluphenazine in the long-term treatment of schizophrenia. Prog Neuropsychopharmacol Biol Psychiatry. 2004;28(2):311-8.

283. Downing ACM, Kinon BJ, Millen BA, Zhang L, Liu L, Morozova MA, et al. A double-blind, placebo-controlled comparator study of LY2140023 monohydrate in patients with schizophrenia. BMC Psychiatry. 2014;14(1):351.

284. Dufresne RL, Valentino D, Kass DJ. Thioridazine improves affective symptoms in schizophrenic patients. Psychopharmacol Bull. 1993;29(2):249-55.

285. Durif F, Debilly B, Galitzky M, Morand D, Viallet F, Borg M, et al. Clozapine improves dyskinesias in Parkinson disease A double-blind, placebo-controlled study. Neurology. 2004;62(3):381-8.

286. Earley W, Burgess MV, Rekeda L, Dickinson R, Szatmári B, Németh G, et al. Cariprazine Treatment of Bipolar Depression: A Randomized Double-Blind Placebo-Controlled Phase 3 Study. Am J Psychiatry. 2019;176(6):439-48.

287. Ehrlich S, Leopold K, Merle JV, Theophil I, Haag W, Lautenschlager M, et al. Trajectories of agouti-related protein and leptin levels during antipsychotic-associated weight gain in patients with schizophrenia. J Clin Psychopharmacol. 2012;32(6):767-72.

288. El-Mallakh RS, Marcus R, Baudelet C, McQuade R, Carson WH, Owen R. A 40-week double-blind aripiprazole versus lithium follow-up of a 12-week acute phase study (total 52 weeks) in bipolar I disorder. J Affect Disord. 2012;136(3):258-66.

289. Emsley R, Turner HJ, Schronen J, Botha K, Smit R, Oosthuizen PP. A single-blind, randomized trial comparing quetiapine and haloperidol in the treatment of tardive dyskinesia. J Clin Psychiatry. 2004;65(5):696-701.

290. Emsley R, Turner HJ, Schronen J, Botha K, Smit R, Oosthuizen PP. Effects of quetiapine and haloperidol on body mass index and glycaemic control: a long-term, randomized, controlled trial. Int J Neuropsychopharmacol. 2005;8(2):175-82.

291. Endicott J, Rajagopalan K, Minkwitz M, Macfadden W. A randomized, double-blind, placebo-controlled study of quetiapine in the treatment of bipolar I and II depression: Improvements in quality of life. International Clinical Psychopharmacology. 2007;22(1):29-37.

292. Essock SM, Frisman LK, Covell NH, Hargreaves WA. Cost-effectiveness of clozapine compared with conventional antipsychotic medication for patients in state hospitals. Arch Gen Psychiatry. 2000;57(10):987-94.

293. Essock SM, Schooler NR, Stroup TS, McEvoy JP, Rojas I, Covell NH, et al. Effectiveness of switching from antipsychotic polypharmacy to monotherapy. American Journal of Psychiatry. 2011;168(7):702-8.

294. Fava M, Mischoulon D, Iosifescu D, Witte J, Pencina M, Flynn M, et al. A double-blind, placebo-controlled study of aripiprazole adjunctive to antidepressant therapy among depressed outpatients with inadequate response to prior antidepressant therapy (ADAPT-A Study). Psychother Psychosom. 2012;81(2):87-97.

295. Feng Y, Shi J, Wang L, Zhang X, Tan Y, Zhao J, et al. Randomized, double-blind, 6-week non-inferiority study of lurasidone and risperidone for the treatment of schizophrenia. Psychiat Clin Neuros. 2019.

296. Fleischhacker WW, Sanchez R, Johnson B, Jin N, Forbes RA, McQuade R, et al. Long-term safety and tolerability of aripiprazole once-monthly in maintenance treatment of patients with schizophrenia. Int Clin Psychopharmacol. 2013;28(4):171-6.

297. Fleischhacker WW, Siu CO, Bodén R, Pappadopulos E, Karayal ON, Kahn RS. Metabolic risk factors in first-episode schizophrenia: Baseline prevalence and course analysed from the European First-Episode Schizophrenia Trial. Int J Neuropsychoph. 2013;16(5):987-95.

298. Fong T, Kalechstein A, Bernhard B, Rosenthal R, Rugle L. A double-blind, placebo-controlled trial of olanzapine for the treatment of video poker pathological gamblers. Pharmacology Biochemistry and Behavior. 2008;89(3):298-303.

299. Fountaine RJ, Taylor AE, Mancuso JP, Greenway FL, Byerley LO, Smith SR, et al. Increased food intake and energy expenditure following administration of olanzapine to healthy men. Obesity (Silver Spring). 2010;18(8):1646-51.

300. Frank E, Wallace ML, Hall M, Hasler B, Levenson JC, Janney CA, et al. An Integrated Risk Reduction Intervention can reduce body mass index in individuals being treated for bipolar I disorder: Results from a randomized trial. Bipolar Disorders. 2015;17(4):424-37.

301. Friedman JH. Low-dose clozapine for the treatment of drug-induced psychosis in Parkinson's disease. New England Journal of Medicine. 1999;340(10):757-63.

302. Fu DJ, Bossie CA, Sliwa JK, Ma YW, Alphs L. Paliperidone palmitate versus oral risperidone and risperidone long-acting injection in patients with recently diagnosed schizophrenia: a tolerability and efficacy comparison. Int Clin Psychopharmacol. 2014;29(1):45-55.

303. Fukushi R, Nomura Y, Katashima M, Komatsu K, Sato Y, Takada A. Population Pharmacokinetics Analysis of Quetiapine Extended-release Formulation in Japanese Patients with Bipolar Depression. Clin Ther. 2020;42(6):1067-76.e2.

304. Gaebel W, Schreiner A, Bergmans P, de Arce R, Rouillon F, Cordes J, et al. Relapse prevention in schizophrenia and schizoaffective disorder with risperidone long-acting injectable vs quetiapine: results of a long-term, open-label, randomized clinical trial. Neuropsychopharmacology. 2010;35(12):2367-77.

305. Gafoor R, Landau S, Craig TKJ, Elanjithara T, Power P, McGuire P. Esquire trial: Efficacy and adverse effects of quetiapine versus risperidone in first-episode schizophrenia. Journal of Clinical Psychopharmacology. 2010;30(5):600-6.

306. Ganguli R, Brar JS, Mahmoud R, Berry SA, Pandina GJ. Assessment of strategies for switching patients from olanzapine to risperidone: a randomized, open-label, rater-blinded study. BMC Med. 2008;6:17.

307. Gao K, Goto T, Yuan C, Brownrigg B, Conroy C, Chan PK, et al. A pilot study of the effectiveness of lithium versus quetiapine immediate release monotherapy in patients with bipolar spectrum disorders. Journal of Clinical Psychopharmacology. 2018;38(5):422-34.

308. Gareri P, Cotroneo A, Lacava R, Seminara G, Marigliano N, Loiacono A, et al. Comparison of the efficacy of new and conventional antipsychotic drugs in the treatment of behavioral and psychological symptoms of dementia (BPSD). Archives of Gerontology and Geriatrics. 2004;38(SUPPL.):207-15.

309. Garriga M, Solé E, González-Pinto A, Selva-Vera G, Arranz B, Amann BL, et al. Efficacy of quetiapine XR vs. placebo as concomitant treatment to mood stabilizers in the control of subthreshold symptoms of bipolar disorder: Results from a pilot, randomized controlled trial. Eur Neuropsychopharmacol. 2017;27(10):959-69.

310. Gattaz WF, Diehl A, Geuppert MS, Hubrich P, Schmitt A, Linde I, et al. Olanzapine versus flupenthixol in the treatment of inpatients with schizophrenia: a randomized double-blind trial. Pharmacopsychiatry. 2004;37(6):279-85.

311. Geffen Y, Keefe R, Rabinowitz J, Anand R, Davidson M. BL-1020, a new gamma-aminobutyric acid-enhanced antipsychotic: Results of 6-week, randomized, double-blind, controlled, efficacy and safety study. Journal of Clinical Psychiatry. 2012;73(9):e1168-e74.

312. Godleski LS, Goldsmith LJ, Vieweg WV, Zettwoch NC, Stikovac DM, Lewis SJ. Switching from depot antipsychotic drugs to olanzapine in patients with chronic schizophrenia. J Clin Psychiatry. 2003;64(2):119-22.

313. Godleski LS, Goldsmith LJ, Vieweg WV, Zettwoch N, Stikovac D, Lewis S. Switching depot antipsychotic drug responders to oral olanzapine. Prog Neuropsychopharmacol Biol Psychiatry. 2005;29(1):141-4.

314. Gomez-Revuelta M, Pelayo-Teran JM, Juncal-Ruiz M, Ortiz-Garcia de la Foz V, Vazquez-Bourgon J, Gonzalez-Pinto A, et al. Long-term antipsychotic effectiveness in first episode of psychosis: A 3-year follow-up randomized clinical trial comparing aripiprazole, quetiapine, and ziprasidone. Int J Neuropsychoph. 2018;21(12):1090-101.

315. Gomez-Revuelta M, Pelayo-Teran JM, Juncal-Ruiz M, Vazquez-Bourgon J, Suarez-Pinilla P, Romero-Jimenez R, et al. Antipsychotic Treatment Effectiveness in First Episode of Psychosis: PAFIP 3-Year Follow-Up Randomized Clinical Trials Comparing Haloperidol, Olanzapine, Risperidone, Aripiprazole, Quetiapine, and Ziprasidone. The international journal of neuropsychopharmacology. 2020;23(4):217-29.

316. Gureje O, Miles W, Keks N, Grainger D, Lambert T, McGrath J, et al. Olanzapine vs risperidone in the management of schizophrenia: A randomized double-blind trial in Australia and New Zealand. Schizophr Res. 2003;61(2-3):303-14.

317. Hamilton JD, Nguyen QX, Gerber RM, Rubio NB. Olanzapine in cocaine dependence: A double-blind, placebo-controlled trial. American Journal on Addictions. 2009;18(1):48-52.

318. Hard ML, Mills RJ, Sadler BM, Wehr AY, Weiden PJ, von Moltke L. Pharmacokinetic Profile of a 2-Month Dose Regimen of Aripiprazole Lauroxil: A Phase I Study and a Population Pharmacokinetic Model. CNS Drugs. 2017;31(7):617-24.

319. Hard ML, Wehr A, von Moltke L, Du Y, Farwick S, Walling DP, et al. Pharmacokinetics and safety of deltoid or gluteal injection of aripiprazole lauroxil NanoCrystal Dispersion used for initiation of the long-acting antipsychotic aripiprazole lauroxil. Therapeutic Advances in Psychopharmacology. 2019;9.

320. Hardy TA, Henry RR, Forrester TD, Kryzhanovskaya LA, Campbell GM, Marks DM, et al. Impact of olanzapine or risperidone treatment on insulin sensitivity in schizophrenia or schizoaffective disorder. Diabetes Obes Metab. 2011;13(8):726-35.

321. Harvey PD, Nakamura H, Murasaki M. Blonanserin versus haloperidol in Japanese patients with schizophrenia: A phase 3, 8-week, double-blind, multicenter, randomized controlled study. Neuropsychopharmacology Reports. 2019;39(3):173-82.

322. Harvey PD, Nakamura H, Miura S. Blonanserin vs risperidone in Japanese patients with schizophrenia: A post hoc analysis of a phase 3, 8-week, multicenter, double-blind, randomized controlled study. Neuropsychopharmacology Reports. 2020;40(1):63-72.

323. Hatta K, Sato K, Hamakawa H, Takebayashi H, Kimura N, Ochi S, et al. Effectiveness of second-generation antipsychotics with acute-phase schizophrenia. Schizophr Res. 2009;113(1):49-55.

324. Hinze-Selch D, Deuschle M, Weber B, Heuser I, Pollmächer T. Effect of coadministration of clozapine and fluvoxamine versus clozapine monotherapy on blood cell counts, plasma levels of cytokines and body weight. Psychopharmacology (Berl). 2000;149(2):163-9.

325. Hirsch SR, Kissling W, Bäuml J, Power A, O'Connor R. A 28-week comparison of ziprasidone and haloperidol in outpatients with stable schizophrenia. J Clin Psychiatry. 2002;63(6):516-23.

326. Hirschfeld RMA, Keck Jr PE, Kramer M, Karcher K, Canuso C, Eerdekens M, et al. Rapid antimanic effect of risperidone monotherapy: A 3-week multicenter, double-blind, placebo-controlled trial. American Journal of Psychiatry. 2004;161(6):1057-65.

327. Hough D, Lindenmayer JP, Gopal S, Melkote R, Lim P, Herben V, et al. Safety and tolerability of deltoid and gluteal injections of paliperidone palmitate in schizophrenia. Prog Neuropsychopharmacol Biol Psychiatry. 2009;33(6):1022-31.

328. Howanitz E, Pardo M, Smelson DA, Engelhart C, Eisenstein N, Stern RG, et al. The efficacy and safety of clozapine versus chlorpromazine in geriatric schizophrenia. J Clin Psychiatry. 1999;60(1):41-4.

329. Hu S, Yao M, Peterson BS, Xu D, Hu J, Tang J, et al. A randomized, 12-week study of the effects of extended-release paliperidone (paliperidone ER) and olanzapine on metabolic profile, weight, insulin resistance, and beta-cell function in schizophrenic patients. Psychopharmacology. 2013;230(1):3-13.

330. Huang M, Yu L, Pan F, Lu S, Hu S, Hu J, et al. A randomized, 13-week study assessing the efficacy and metabolic effects of paliperidone palmitate injection and olanzapine in first-episode schizophrenia patients. Prog Neuropsychopharmacol Biol Psychiatry. 2018;81:122-30.

331. Huttunen MO, Piepponen T, Rantanen H, Larmo I, Nyholm R, Raitasuo V. Risperidone versus zuclopenthixol in the treatment of acute schizophrenic episodes: a double-blind parallel-group trial. Acta Psychiatr Scand. 1995;91(4):271-7.

332. Hwang TJ, Lin SK, Lin HN. Efficacy and safety of zotepine for the treatment of Taiwanese schizophrenic patients: a double-blind comparison with haloperidol. J Formos Med Assoc. 2001;100(12):811-6.

333. Hwang TJ, Lee SM, Sun HJ, Lin HN, Tsai SJ, Lee YC, et al. Amisulpride versus risperidone in the treatment of schizophrenic patients: a double-blind pilot study in Taiwan. J Formos Med Assoc. 2003;102(1):30-6.

334. Hwang TJ, Lo WM, Chan HY, Lin CF, Hsieh MH, Liu CC, et al. Fast Versus Slow Strategy of Switching Patients With Schizophrenia to Aripiprazole From Other Antipsychotics. J Clin Psychopharmacol. 2015;35(6):635-44.

335. Ingole S, Belorkar NR, Waradkar P, Shrivastava M. Comparison of effects of olanzapine and risperidone on body mass index and blood sugar level in schizophrenic patients. Indian J Physiol Pharmacol. 2009;53(1):47-54.

336. Ionescu D, Dehelean C, Funar-Timofei S, Dragan S, Galca E. Efficacy and tolerability of risperidone in the treatment of depressive disorders. Farmacia. 2010;58(4):494-501.

337. Ishigooka J, Nakamura J, Fujii Y, Iwata N, Kishimoto T, Iyo M, et al. Efficacy and safety of aripiprazole once-monthly in Asian patients with schizophrenia: a multicenter, randomized, double-blind, non-inferiority study versus oral aripiprazole. Schizophr Res. 2015;161(2-3):421-8.

338. Jena M, Ranjan R, Mishra BR, Mishra A, Nath S, Sahu P, et al. Effect of lurasidone vs olanzapine on neurotrophic biomarkers in unmedicated schizophrenia: A randomized controlled trial. Journal of Psychiatric Research. 2019;112:1-6.

339. Johnsen E, Kroken RA, Wentzel-Larsen T, Jorgensen HA. Effectiveness of second-generation antipsychotics: A naturalistic, randomized comparison of olanzapine, quetiapine, risperidone, and ziprasidone. BMC Psychiatry. 2010;10:26.

340. Johnsen E, Kroken RA, Løberg EM, Rettenbacher M, Joa I, Larsen TK, et al. Amisulpride, aripiprazole, and olanzapine in patients with schizophrenia-spectrum disorders (BeSt InTro): a pragmatic, rater-blind, semi-randomised trial. Lancet Psychiat. 2020;7(11):945-54.

341. Jokinen K, Koskinen T, Selonen R. Flupenthixol versus diazepam in the treatment of psychosomatic disorders: a double-blind, multi-centre trial in general practice. Pharmatherapeutica. 1984;3(9):573-81.

342. Jones PB, Barnes TR, Davies L, Dunn G, Lloyd H, Hayhurst KP, et al. Randomized controlled trial of the effect on Quality of Life of second- vs first-generation antipsychotic drugs in schizophrenia: Cost Utility of the Latest Antipsychotic Drugs in Schizophrenia Study (CUtLASS 1). Arch Gen Psychiatry. 2006;63(10):1079-87.

343. Jus A, Pineau R, Jus K, Villeneuve A, Gautier J, Pires P, et al. Penfluridol: a long-acting oral neuroleptic as therapeutic agent in chronic schizophrenia. Curr Ther Res Clin Exp. 1974;16(10):1041-58.

344. Kahn RS, Fleischhacker WW, Boter H, Davidson M, Vergouwe Y, Keet IP, et al. Effectiveness of antipsychotic drugs in first-episode schizophrenia and schizophreniform disorder: an open randomised clinical trial. The Lancet. 2008;371(9618):1085-97.

345. Kane JM, Eerdekens M, Lindenmayer JP, Keith SJ, Lesem M, Karcher K. Long-acting injectable risperidone: efficacy and safety of the first long-acting atypical antipsychotic. Am J Psychiatry. 2003;160(6):1125-32.

346. Kane JM, Meltzer HY, Carson Jr WH, McQuade RD, Marcus RN, Sanchez R. Aripiprazole for treatment-resistant schizophrenia: Results of a multicenter, randomized, double-blind, comparison study versus perphenazine. Journal of Clinical Psychiatry. 2007;68(2):213-23.

347. Kane JM, Detke HC, Naber D, Sethuraman G, Lin DY, Bergstrom RF, et al. Olanzapine long-acting injection: a 24-week, randomized, double-blind trial of maintenance treatment in patients with schizophrenia. Am J Psychiatry. 2010;167(2):181-9.

348. Kane JM, Schooler NR, Marcy P, Correll CU, Achtyes ED, Gibbons RD, et al. Effect of Long-Acting Injectable Antipsychotics vs Usual Care on Time to First Hospitalization in Early-Phase Schizophrenia: A Randomized Clinical Trial. JAMA Psychiatry. 2020;77(12):1217-24.

349. Kasper S, Lerman MN, McQuade RD, Saha A, Carson WH, Ali M, et al. Efficacy and safety of aripiprazole vs. haloperidol for long-term maintenance treatment following acute relapse of schizophrenia. Int J Neuropsychoph. 2003;6(4):325-37.

350. Keck Jr P, Buffenstein A, Ferguson J, Feighner J, Jaffe W, Harrigan EP, et al. Ziprasidone 40 and 120 mg/day in the acute exacerbation of schizophrenia and schizoaffective disorder: A 4-week placebo-controlled trial. Psychopharmacology. 1998;140(2):173-84.

351. Keck PE, Jr., Versiani M, Potkin S, West SA, Giller E, Ice K. Ziprasidone in the treatment of acute bipolar mania: a three-week, placebo-controlled, double-blind, randomized trial. Am J Psychiatry. 2003;160(4):741-8.

352. Keck PE, Orsulak PJ, Cutler AJ, Sanchez R, Torbeyns A, Marcus RN, et al. Aripiprazole monotherapy in the treatment of acute bipolar I mania: a randomized, double-blind, placebo- and lithium-controlled study. J Affect Disord. 2009;112(1-3):36-49.

353. Keefe RSE, Young CA, Rock SL, Purdon SE, Gold JM, Breier A. One-year double-blind study of the neurocognitive efficacy of olanzapine, risperidone, and haloperidol in schizophrenia. Schizophr Res. 2006;81(1):1-5.

354. Keefe RS, Seidman LJ, Christensen BK, Hamer RM, Sharma T, Sitskoorn MM, et al. Comparative effect of atypical and conventional antipsychotic drugs on neurocognition in first-episode psychosis: a randomized, double-blind trial of olanzapine versus low doses of haloperidol. Am J Psychiatry. 2004;161(6):985-95.

355. Kelly DL, Conley RR, Love RC, Morrison JA, McMahon RP. Metabolic risk with second-generation antipsychotic treatment: A double-blind randomized 8-week trial of risperidone and olanzapine. Annals of Clinical Psychiatry. 2008;20(2):71-8.

356. Kennedy JS, Jeste D, Kaiser CJ, Golshan S, Maguire GA, Tollefson G, et al. Olanzapine vs haloperidol in geriatric schizophrenia: Analysis of data from a double-blind controlled trial. International Journal of Geriatric Psychiatry. 2003;18(11):1013-20.

357. Kennedy J, Deberdt W, Siegal A, Micca J, Degenhardt E, Ahl J, et al. Olanzapine does not enhance cognition in non-agitated and non-psychotic patients with mild to moderate Alzheimer's dementia. International Journal of Geriatric Psychiatry. 2005;20(11):1020-7.

358. Khanna S, Vieta E, Lyons B, Grossman F, Eerdekens M, Kramer M. Risperidone in the treatment of acute mania: Double-blind, placebo-controlled study. Brit J Psychiat. 2005;187(SEPT.):229-34.

359. Kim CY, Chung S, Lee JN, Kwon JS, Kim DH, Kim CE, et al. A 12-week, naturalistic switch study of the efficacy and tolerability of aripiprazole in stable outpatients with schizophrenia or schizoaffective disorder. International Clinical Psychopharmacology. 2009;24(4):181-8.

360. Kingstone E, Kolivakis T, Kossatz I. Double blind study of clopenthixol and chlorpromazine in acute hospitalized schizophrenics. Int Z Klin Pharmakol Ther Toxikol. 1970;3(1):41-5.

361. Kinon BJ, Noordsy DL, Liu-Seifert H, Gulliver AH, Ascher-Svanum H, Kollack-Walker S. Randomized, double-blind 6-month comparison of olanzapine and quetiapine in patients with schizophrenia or schizoaffective disorder with prominent negative symptoms and poor functioning. Journal of Clinical Psychopharmacology. 2006;26(5):453-61.

362. Kinon BJ, Chen L, Ascher-Svanum H, Stauffer VL, Kollack-Walker S, Zhou W, et al. Early response to antipsychotic drug therapy as a clinical marker of subsequent response in the treatment of schizophrenia. Neuropsychopharmacology. 2010;35(2):581-90.

363. Kluge M, Schuld A, Himmerich H, Dalal M, Schacht A, Wehmeier PM, et al. Clozapine and olanzapine are associated with food craving and binge eating: results from a randomized double-blind study. J Clin Psychopharmacol. 2007;27(6):662-6.

364. Kluge M, Schuld A, Schacht A, Himmerich H, Dalal MA, Wehmeier PM, et al. Effects of clozapine and olanzapine on cytokine systems are closely linked to weight gain and drug-induced fever. Psychoneuroendocrinology. 2009;34(1):118-28.

365. Kolotkin RL, Corey-Lisle PK, Crosby RD, Kan HJ, McQuade RD. Changes in weight and weight-related quality of life in a multicentre, randomized trial of aripiprazole versus standard of care. Eur Psychiatry. 2008;23(8):561-6.

366. Krakowski M, Czobor P. Cholesterol and cognition in schizophrenia: A double-blind study of patients randomized to clozapine, olanzapine and haloperidol. Schizophr Res. 2011;130(1-3):27-33.

367. Krystal JH, Rosenheck RA, Cramer JA, Vessicchio JC, Jones KM, Vertrees JE, et al. Adjunctive risperidone treatment for antidepressant-resistant symptoms of chronic military service-related PTSD: a randomized trial. Jama. 2011;306(5):493-502.

368. Kusumi I, Honda M, Uemura K, Sugawara Y, Kohsaka M, Tochigi A, et al. Effect of olanzapine orally disintegrating tablet versus oral standard tablet on body weight in patients with schizophrenia: A randomized open-label trial. Progress in Neuro-Psychopharmacology and Biological Psychiatry. 2012;36(2):313-7.

369. Kwon JS, Mittoux A, Hwang JY, Ong A, Cai ZJ, Su TP. The efficacy and safety of 12 weeks of treatment with sertindole or olanzapine in patients with chronic schizophrenia who did not respond successfully to their previous treatments: A randomized, double-blind, parallel-group, flexible-dose study. International Clinical Psychopharmacology. 2012;27(6):326-35.

370. Kwon JS, Kim SN, Han J, Lee SI, Chang JS, Choi JS, et al. Satisfaction of immediate or delayed switch to paliperidone palmitate in patients unsatisfied with current oral atypical antipsychotics. International Clinical Psychopharmacology. 2015;30(6):320-8.

371. Lecrubier Y, Quintin P, Bouhassira M, Perrin E, Lancrenon S. The treatment of negative symptoms and deficit states of chronic schizophrenia: Olanzapine compared to amisulpride and placebo in a 6-month double-blind controlled clinical trial. Acta Psychiatrica Scandinavica. 2006;114(5):319-27.

372. Lee CT, Conde BJ, Mazlan M, Visanuyothin T, Wang A, Wong MM, et al. Switching to olanzapine from previous antipsychotics: a regional collaborative multicenter trial assessing 2 switching techniques in Asia Pacific. J Clin Psychiatry. 2002;63(7):569-76.

373. Lee SJ, Lee JH, Jung SW, Koo BH, Choi TY, Lee KH. A 6-week, randomized, multicentre, open-label study comparing efficacy and tolerability of amisulpride at a starting dose of 400 mg/day versus 800 mg/day in patients with acute exacerbations of schizophrenia. Clin Drug Investig. 2012;32(11):735-45.

374. Lencz T, Robinson DG, Napolitano B, Sevy S, Kane JM, Goldman D, et al. DRD2 promoter region variation predicts antipsychotic-induced weight gain in first episode schizophrenia. Pharmacogenet Genomics. 2010;20(9):569-72.

375. Li YM, Zhao JP, Ou JJ, Wu RR. Efficacy and tolerability of ziprasidone vs olanzapine in naive first-episode schizophrenia: A 6-week, randomized, open-label, flexible-dose study. Pharmacopsychiatry. 2012;45(5):177-81.

376. Lieberman JA, Phillips M, Gu H, Stroup S, Zhang P, Kong L, et al. Atypical and conventional antipsychotic drugs in treatment-naive first-episode schizophrenia: a 52-week randomized trial of clozapine vs chlorpromazine. Neuropsychopharmacology. 2003;28(5):995-1003.

377. Lieberman JA, Davis RE, Correll CU, Goff DC, Kane JM, Tamminga CA, et al. ITI-007 for the Treatment of Schizophrenia: A 4-Week Randomized, Double-Blind, Controlled Trial. Biological Psychiatry. 2016;79(12):952-61.

378. Liebowitz M, Lam RW, Lepola U, Datto C, Sweitzer D, Eriksson H. Efficacy and tolerability of extended release quetiapine fumarate monotherapy as maintenance treatment of major depressive disorder: A randomized, placebo-controlled trial. Depression and Anxiety. 2010;27(10):964-76.

379. Lin CH, Kuo CC, Chou LS, Chen YH, Chen CC, Huang KH, et al. A randomized, double-blind comparison of risperidone versus low-dose risperidone plus low-dose haloperidol in treating schizophrenia. J Clin Psychopharmacol. 2010;30(5):518-25.

380. Lin CH, Wang FC, Lin SC, Huang YH, Chen CC, Lane HY. Antipsychotic combination using low-dose antipsychotics is as efficacious and safe as, but cheaper, than optimal-dose monotherapy in the treatment of schizophrenia: A randomized, double-blind study. International Clinical Psychopharmacology. 2013;28(5):267-74.

381. Lin CH, Wang FC, Lin SC, Huang YH, Chen CC. A randomized, double-blind, comparison of the efficacy and safety of low-dose olanzapine plus low-dose trifluoperazine versus full-dose olanzapine in the acute treatment of schizophrenia. Schizophr Res. 2017;185:80-7.

382. Lindenmayer JP, Khan A, Iskander A, Abad MT, Parker B. A randomized controlled trial of olanzapine versus haloperidol in the treatment of primary negative symptoms and neurocognitive deficits in schizophrenia. J Clin Psychiatry. 2007;68(3):368-79.

383. Lindenmayer JP, Citrome L, Khan A, Kaushik S. A randomized, double-blind, parallel-group, fixed-dose, clinical trial of quetiapine at 600 versus 1200 mg/d for patients with treatment-resistant schizophrenia or schizoaffective disorder. Journal of Clinical Psychopharmacology. 2011;31(2):160-8.

384. Littlewood RA, Claus ED, Arenella P, Bogenschutz M, Karoly H, Ewing SWF, et al. Dose specific effects of olanzapine in the treatment of alcohol dependence. Psychopharmacology. 2015;232(7):1261-8.

385. Liu J, Sun J, Shen X, Guo W, Zhi S, Song G, et al. Randomized controlled trial comparing changes in serum prolactin and weight among female patients with first-episode schizophrenia over 12 months of treatment with risperidone or quetiapine. Shanghai Archives of Psychiatry. 2014;26(2):88-94.

386. Liu Q, Zhang HY, Cao QJ, Shuang M, Yang FD, Wang CY, et al. Efficacy and tolerability of domestic blonanserin tablets in acute schizophrenic: a randomized, double-blind, risperidone-controlled trial. Chinese Journal of New Drugs. 2016;25(7):779-86.

387. Loebel A, Cucchiaro J, Xu J, Sarma K, Pikalov A, Kane JM. Effectiveness of lurasidone vs quetiapine XR for relapse prevention in schizophrenia: A 12-month, double-blind, noninferiority study. Schizophr Res. 2013;147(1):95-102.

388. Loebl T, Angarita GA, Pachas GN, Huang KL, Lee SH, Nino J, et al. A randomized, double-blind, placebo-controlled trial of long-acting risperidone in cocaine-dependent men. J Clin Psychiatry. 2008;69(3):480-6.

389. Lofwall MR, Nuzzo PA, Campbell C, Walsh SL. Aripiprazole effects on self-administration and pharmacodynamics of intravenous cocaine and cigarette smoking in humans. Experimental and Clinical Psychopharmacology. 2014;22(3):238-47.

390. Lohoff FW, Etemad B, Mandos LA, Gallop R, Rickels K. Ziprasidone treatment of refractory generalized anxiety disorder: A placebo-controlled, double-blind study. Journal of Clinical Psychopharmacology. 2010;30(2):185-9.

391. MacFadden W, Ma YW, Thomas Haskins J, Bossie CA, Alphs L. A prospective study comparing the long-term effectiveness of injectable risperidone long-acting therapy and oral aripiprazole in patients with schizophrenia. Psychiatry (Edgemont). 2010;7(11):23-31.

392. Maguire GA, Riley GD, Franklin DL, Maguire ME, Nguyen CT, Brojeni PH. Olanzapine in the treatment of developmental stuttering: A double-blind, placebo-controlled trial. Annals of Clinical Psychiatry. 2004;16(2):63-7.

393. Maina G, Pessina E, Albert U, Bogetto F. 8-week, single-blind, randomized trial comparing risperidone versus olanzapine augmentation of serotonin reuptake inhibitors in treatment-resistant obsessive-compulsive disorder. Eur Neuropsychopharmacol. 2008;18(5):364-72.

394. Maitra A, Bhattacharyya S, Mukhopadhyay S, Mallick AK, Biswas S, Singh OP. A Randomized Controlled Trial to Compare the Efficacy, Safety and Tolerability of Asenapine versus Olanzapine in Management of Schizophrenia. Clinical Psychopharmacology and Neuroscience. 2020;18(4):587-98.

395. Mamo D, Kapur S, Shammi CM, Papatheodorou G, Mann S, Therrien F, et al. A PET Study of Dopamine D2 and Serotonin 5-HT2 Receptor Occupancy in Patients with Schizophrenia Treated with Therapeutic Doses of Ziprasidone. American Journal of Psychiatry. 2004;161(5):818-25.

396. Marni T, Loebis B, Camellia V, Effendy E, Nasution NM. The difference of fasting blood sugar of male patients with schizophrenia treated with flexible dose between aripiprazole and risperidone in medan, Indonesia. Open Access Macedonian Journal of Medical Sciences. 2019;7(9):1446-51.

397. Marra D, Warot D, Berlin I, Hispard E, Notides C, Tilikete S, et al. Amisulpride does not prevent relapse in primary alcohol dependence: Results of a pilot randomized, placebo-controlled trial. Alcoholism: Clinical and Experimental Research. 2002;26(10):1545-52.

398. Martin W, Graham C, Morrow L, Beysen C, Toledo FGS, Bajorunas D, et al. 184 Insulin Sensitivity and Glucose Metabolism of Olanzapine and Combination Olanzapine and Samidorphan: A Phase 1 Exploratory Study in Healthy Volunteers. CNS spectrums. 2020;25(2):316.

399. Mazza M, Squillacioti MR, Pecora RD, Janiri L, Bria P. Beneficial acute antidepressant effects of aripiprazole as an adjunctive treatment or monotherapy in bipolar patients unresponsive to mood stabilizers: results from a 16-week open-label trial. Expert Opin Pharmacother. 2008;9(18):3145-9.

400. McDougle CJ, Holmes JP, Carlson DC, Pelton GH, Cohen DJ, Price LH. A double-blind, placebo-controlled study of risperidone in adults with autistic disorder and other pervasive developmental disorders. Arch Gen Psychiatry. 1998;55(7):633-41.

401. McEvoy JP, Lieberman JA, Perkins DO, Hamer RM, Gu H, Lazarus A, et al. Efficacy and tolerability of olanzapine, quetiapine, and risperidone in the treatment of early psychosis: a randomized, double-blind 52-week comparison. Am J Psychiatry. 2007;164(7):1050-60.

402. McEvoy JP, Citrome L, Hernandez D, Cucchiaro J, Hsu J, Pikalov A, et al. Effectiveness of lurasidone in patients with schizophrenia or schizoaffective disorder switched from other antipsychotics: a randomized, 6-week, open-label study. J Clin Psychiatry. 2013;74(2):170-9.

403. McEvoy JP, Byerly M, Hamer RM, Dominik R, Swartz MS, Rosenheck RA, et al. Effectiveness of paliperidone palmitate vs haloperidol decanoate for maintenance treatment of schizophrenia: a randomized clinical trial. Jama. 2014;311(19):1978-87.

404. McGlashan TH, Zipursky RB, Perkins D, Addington J, Miller T, Woods SW, et al. Randomized, double-blind trial of olanzapine versus placebo in patients prodromally symptomatic for psychosis. Am J Psychiatry. 2006;163(5):790-9.

405. McIntyre RS, McElroy SL, Eudicone JM, Forbes RA, Carlson BX, Baker RA. A 52-week, double-blind evaluation of the metabolic effects of aripiprazole and lithium in bipolar I disorder. Primary Care Companion to the Journal of Clinical Psychiatry. 2011;13(6).

406. McLaren S, Cookson JC, Silverstone T. Positive and negative symptoms, depression and social disability in chronic schizophrenia: a comparative trial of bromperidol and fluphenazine decanoates. Int Clin Psychopharmacol. 1992;7(2):67-72.

407. Mearin F, Rodrigo L, Pérez-Mota A, Balboa A, Jiménez I, Sebastián JJ, et al. Levosulpiride and cisapride in the treatment of dysmotility-like functional dyspepsia: a randomized, double-masked trial. Clin Gastroenterol Hepatol. 2004;2(4):301-8.

408. Meltzer HY, Bobo WV, Roy A, Jayathilake K, Chen Y, Ertugrul A, et al. A randomized, double-blind comparison of clozapine and high-dose olanzapine in treatment-resistant patients with schizophrenia. J Clin Psychiatry. 2008;69(2):274-85.

409. Meltzer HY, Risinger R, Nasrallah HA, Du Y, Zummo J, Corey L, et al. A randomized, double-blind, placebo-controlled trial of aripiprazole lauroxil in acute exacerbation of schizophrenia. Journal of Clinical Psychiatry. 2015;76(8):1085-90.

410. Miceli JJ, Wilner KD, Hansen RA, Johnson AC, Apseloff G, Gerber N. Single- and multiple-dose pharmacokinetics of ziprasidone under non-fasting conditions in healthy male volunteers. Br J Clin Pharmacol. 2000;49 Suppl 1(Suppl 1):5s-13s.

411. Min SK, Rhee CS, Kim CE, Kang DY. Risperidone versus haloperidol in the treatment of chronic schizophrenic patients: a parallel group double-blind comparative trial. Yonsei Med J. 1993;34(2):179-90.

412. Moretti R, Torre P, Antonello RM, Cattaruzza T, Cazzato G. Olanzapine as a possible treatment of behavioral symptoms in vascular dementia: Risks of cerebrovascular events - A controlled, open-label study. Journal of Neurology. 2005;252(10):1186-93.

413. Moretti R, Torre P, Antonello RM, Cattaruzza T, Cazzato G, Bava A. Olanzapine as a possible treatment for anxiety due to vascular dementia: An open study. American Journal of Alzheimer's Disease and other Dementias. 2004;19(2):81-8.

414. Mosolov SN, Smulevich AB, Neznanov NG, Tochilov VA, Andreev BV, Avedisova AS, et al. mGlu 2/3 agonists—A new approach to the treatment of Schizophrenia: Results of a randomized double-blind trial. Neuroscience and Behavioral Physiology. 2011;41(6):559-66.

415. Mousavi S, Rostami H, Sharbafchi M, Boroujeni A, Mahaki B. Onset of action of atypical and typical antipsychotics in the treatment of acute psychosis. Journal of Research in Pharmacy Practice. 2013;2(4):138-44.

416. Murasaki M, Koyama T, Kanba S, Takeuchi M, Shimizu Y, Arita E, et al. Multi-center, randomized, double-blind, placebo-controlled study of quetiapine extended-release formulation in Japanese patients with bipolar depression. Psychopharmacology (Berl). 2018;235(10):2859-69.

417. Naber D, Peuskens J, Schwarzmann N, Goltz M, Kruger H, Lambert M, et al. Subjective well-being in schizophrenia: A randomised controlled open-label 12-month non-inferiority study comparing quetiapine XR with risperidone (RECOVER). European Neuropsychopharmacology. 2013;23(10):1257-69.

418. Nagesh HN, Nagaraj AK, Kishore MS, Narendra Kumar MS. A randomized prospective comparative study of weight gain between asenapine and iloperidone in patients with psychosis. National Journal of Physiology, Pharmacy and Pharmacology. 2017;7(1):94-8.

419. Nasrallah HA, Gopal S, Gassmann-Mayer C, Quiroz JA, Lim P, Eerdekens M, et al. A controlled, evidence-based trial of paliperidone palmitate, a long-acting injectable antipsychotic, in schizophrenia. Neuropsychopharmacology. 2010;35(10):2072-82.

420. Navari RM, Nagy CK, Le-Rademacher J, Loprinzi CL. Olanzapine versus fosaprepitant for the prevention of concurrent chemotherapy radiotherapy-induced nausea and vomiting. Journal of Community and Supportive Oncology. 2016;14(4):141-7.

421. Nemeth G, Laszlovszky I, Czobor P, Szalai E, Szatmari B, Harsanyi J, et al. Cariprazine versus risperidone monotherapy for treatment of predominant negative symptoms in patients with schizophrenia: a randomised, double-blind, controlled trial. The Lancet. 2017;389(10074):1103-13.

422. Newcomer JW, Ratner RE, Eriksson JW, Emsley R, Meulien D, Miller F, et al. A 24-week, multicenter, open-label, randomized study to compare changes in glucose metabolism in patients with schizophrenia receiving treatment with olanzapine, quetiapine, or risperidone. The Journal of Clinical Psychiatry. 2009;70(4):487-99.

423. Nistico G, Ragozzine D, Marano V. A comparative study of penfluridol and flupentixol in the treatment of chronic schizophrenia. J Clin Pharmacol. 1974;14(8):476-82.

424. Noordsy DL, Glynn SM, Sugar CA, O'Keefe CD, Marder SR. Risperidone versus olanzapine among patients with schizophrenia participating in supported employment: Eighteen-month outcomes. J Psychiatr Res. 2017;95:299-307.

425. Ohlmeier MD, Jahn K, Wilhelm-Gossling C, Godecke-Koch T, Hoffmann J, Seifert J, et al. Perazine and carbamazepine in comparison to olanzapine in schizophrenia. Neuropsychobiology. 2007;55(2):81-8.

426. Ogasa M, Kimura T, Nakamura M, Guarino J. Lurasidone in the treatment of schizophrenia: a 6-week, placebo-controlled study. Psychopharmacology (Berl). 2013;225(3):519-30.

427. Okugawa G, Kato M, Wakeno M, Koh J, Morikawa M, Matsumoto N, et al. Randomized clinical comparison of perospirone and risperidone in patients with schizophrenia: Kansai psychiatric multicenter study. Psychiat Clin Neuros. 2009;63(3):322-8.

428. Ondo WG, Levy JK, Vuong KD, Hunter C, Jankovic J. Olanzapine treatment for dopaminergic-induced hallucinations. Movement Disorders. 2002;17(5):1031-5.

429. Ozguven HD, Baskak B, Oner O, Atbasoglu C. Metabolic effects of olanzapine and quetiapine: A six-week randomized, single blind, controlled study. Open Neuropsychopharmacology Journal. 2011;4(1):10-7.

430. Pae CU, Kim JJ, Lee CU, Lee SJ, Lee C, Patkar AA, et al. Rapid versus conventional initiation of quetiapine in the treatment of schizophrenia: a randomized, parallel-group trial. J Clin Psychiatry. 2007;68(3):399-405.

431. Pae CU, Serretti A, Chiesa A, Mandelli L, Lee C, Kim J, et al. Immediate versus gradual suspension of previous treatments during switch to aripiprazole: Results of a randomized, open label study. European Neuropsychopharmacology. 2009;19(8):562-70.

432. Paleacu D, Barak Y, Mirecky I, Mazeh D. Quetiapine treatmdnt for behavioural and psychological symptoms of dementia in alzheimer's disease patients: A 6-week, double-blind, placebo-controlled study. International Journal of Geriatric Psychiatry. 2008;23(4):393-400.

433. Papakostas GI, Vitolo OV, IsHak WW, Rapaport MH, Zajecka JM, Kinrys G, et al. A 12-week, randomized, double-blind, placebo-controlled, sequential parallel comparison trial of ziprasidone as monotherapy for major depressive disorder. Journal of Clinical Psychiatry. 2012;73(12):1541-7.

434. Papakostas GI, Fava M, Baer L, Swee MB, Jaeger A, Bobo WV, et al. Ziprasidone augmentation of escitalopram for major depressive disorder: Efficacy results from a randomized, Double-Blind, Placebo-Controlled Study. American Journal of Psychiatry. 2015;172(12):1251-8.

435. Park S, Yi KK, Kim MS, Hong JP. Effects of ziprasidone and olanzapine on body composition and metabolic parameters: An open-label comparative pilot study. Behavioral and Brain Functions. 2013;9(1):27.

436. Patil ST, Zhang L, Martenyi F, Lowe SL, Jackson KA, Andreev BV, et al. Activation of mGlu2/3 receptors as a new approach to treat schizophrenia: a randomized Phase 2 clinical trial. Nat Med. 2007;13(9):1102-7.

437. Patkar A, Gilmer W, Pae CU, Vöhringer PA, Ziffra M, Pirok E, et al. A 6 week randomized double-blind placebo-controlled trial of ziprasidone for the acute depressive mixed state. PLoS One. 2012;7(4):e34757.

438. Perez-Iglesias R, Crespo-Facorro B, Amado JA, Garcia-Unzueta MT, Ramirez-Bonilla ML, Gonzalez-Blanch C, et al. A 12-week randomized clinical trial to evaluate metabolic changes in drug-naive, first-episode psychosis patients treated with haloperidol, olanzapine, or risperidone. J Clin Psychiatry. 2007;68(11):1733-40.

439. Perez-Iglesias R, Vazquez-Barquero JL, Amado JA, Berja A, Garcia-Unzueta MT, Pelayo-Terán JM, et al. Effect of antipsychotics on peptides involved in energy balance in drug-naive psychotic patients after 1 year of treatment. J Clin Psychopharmacol. 2008;28(3):289-95.

440. Perez-Iglesias R, Crespo-Facorro B, Martinez-Garcia O, Ramirez-Bonilla ML, Alvarez-Jimenez M, Pelayo-Teran JM, et al. Weight gain induced by haloperidol, risperidone and olanzapine after 1 year: findings of a randomized clinical trial in a drug-naive population. Schizophr Res. 2008;99(1-3):13-22.

441. Perez-Iglesias R, Mata I, Pelayo-Teran JM, Amado JA, Garcia-Unzueta MT, Berja A, et al. Glucose and lipid disturbances after 1 year of antipsychotic treatment in a drug-naive population. Schizophr Res. 2009;107(2-3):115-21.

442. Perez-Iglesias R, Martinez-Garcia O, Pardo-Garcia G, Amado JA, Garcia-Unzueta MT, Tabares-Seisdedos R, et al. Course of weight gain and metabolic abnormalities in first treated episode of psychosis: the first year is a critical period for development of cardiovascular risk factors. Int J Neuropsychopharmacol. 2014;17(1):41-51.

443. Perkins DO, Gu H, Weiden PJ, McEvoy JP, Hamer RM, Lieberman JA. Predictors of treatment discontinuation and medication nonadherence in patients recovering from a first episode of schizophrenia, schizophreniform disorder, or schizoaffective disorder: A randomized, double-blind, flexible-dose, multicenter study. Journal of Clinical Psychiatry. 2008;69(1):106-13.

444. Perlis RH, Baker RW, Zarate Jr CA, Brown EB, Schuh LM, Jamal HH, et al. Olanzapine versus risperidone in the treatment of manic or mixed states in bipolar I disorder: A randomized, double-blind trial. Journal of Clinical Psychiatry. 2006;67(11):1747-53.

445. Peuskens J, Bech P, Moller HJ, Bale R, Fleurot O, Rein W. Amisulpride vs. risperidone in the treatment of acute exacerbations of schizophrenia. Psychiatry Research. 1999;88(2):107-17.

446. Peuskens J, De Hert M, Mortimer A. Metabolic control in patients with schizophrenia treated with amisulpride or olanzapine. Int Clin Psychopharmacol. 2007;22(3):145-52.

447. Pivac N, Kozaric-Kovacic D, Muck-Seler D. Olanzapine versus fluphenazine in an open trial in patients with psychotic combat-related post-traumatic stress disorder. Psychopharmacology. 2004;175(4):451-6.

448. Popovic V, Doknic M, Maric N, Pekic S, Damjanovic A, Miljic D, et al. Changes in neuroendocrine and metabolic hormones induced by atypical antipsychotics in normal-weight patients with schizophrenia. Neuroendocrinology. 2007;85(4):249-56.

449. Potkin SG, Ogasa M, Cucchiaro J, Loebel A. Double-blind comparison of the safety and efficacy of lurasidone and ziprasidone in clinically stable outpatients with schizophrenia or schizoaffective disorder. Schizophr Res. 2011;132(2-3):101-7.

450. Potkin SG, Kunovac J, Silverman BL, Simmons A, Jiang Y, DiPetrillo L, et al. 26 A Phase 3 Study to Determine the Antipsychotic Efficacy and Safety of ALKS 3831 in Adult Patients with Acute Exacerbation of Schizophrenia. CNS spectrums. 2019;24(1):187-8.

451. Pu ZP, Li GR, Zou ZP, Tao F, Hu SH. A Randomized, 8-Week Study of the Effects of Extended-Release Paliperidone and Olanzapine on Heart Rate Variability in Patients with Schizophrenia. Journal of Clinical Psychopharmacology. 2019;39(3):243-8.

452. Purdon SE, Malla A, Labelle A, Lit W. Neuropsychological change in patients with schizophrenia after treatment with quetiapine or haloperidol. Journal of Psychiatry and Neuroscience. 2001;26(2):137-49.

453. Quednow BB, Wagner M, Westheide J, Beckmann K, Bliesener N, Maier W, et al. Sensorimotor gating and habituation of the startle response in schizophrenic patients randomly treated with amisulpride or olanzapine. Biol Psychiatry. 2006;59(6):536-45.

454. Raison CL, Pikalov A, Siu C, Tsai J, Koblan K, Loebel A. C-reactive protein and response to lurasidone in patients with bipolar depression. Brain, Behavior, and Immunity. 2018;73:717-24.

455. Ramasubbu R, Burgess A, Gaxiola-Valdez I, Cortese F, Clark D, Kemp A, et al. Amygdala responses to quetiapine XR and citalopram treatment in major depression: The role of 5-HTTLPR-S/Lg polymorphisms. Human Psychopharmacology. 2016;31(2):144-55.

456. Ramerman L, de Kuijper G, Scheers T, Vink M, Vrijmoeth P, Hoekstra PJ. Is risperidone effective in reducing challenging behaviours in individuals with intellectual disabilities after 1 year or longer use? A placebo-controlled, randomised, double-blind discontinuation study. Journal of Intellectual Disability Research. 2019;63(5):418-28.

457. Ramsey TL, Brennan MD. Glucagon-like peptide 1 receptor (GLP1R) haplotypes correlate with altered response to multiple antipsychotics in the CATIE trial. Schizophr Res. 2014;160(1-3):73-9.

458. Raoufinia A, Peters-Strickland T, Nylander AG, Baker RA, Eramo A, Jin N, et al. Aripiprazole once-monthly 400 mg: Comparison of pharmacokinetics, tolerability, and safety of deltoid versus gluteal administration. Int J Neuropsychoph. 2017;20(4):295-304.

459. Ravenstijn P, Remmerie B, Savitz A, Samtani MN, Nuamah I, Chang CT, et al. Pharmacokinetics, safety, and tolerability of paliperidone palmitate 3-month formulation in patients with schizophrenia: A phase-1, single-dose, randomized, open-label study. Journal of Clinical Pharmacology. 2016;56(3):330-9.

460. Reeves H, Batra S, May RS, Zhang R, Dahl DC, Li X. Efficacy of risperidone augmentation to antidepressants in the management of suicidality in major depressive disorder: A randomized, double-blind, placebo-controlled pilot study. Journal of Clinical Psychiatry. 2008;69(8):1228-36.

461. Revicki DA, Paramore LC, Sommerville KW, Swann AC, Zajecka JM. Divalproex sodium versus olanzapine in the treatment of acute mania in bipolar disorder: Health-related quality of life and medical cost outcomes. Journal of Clinical Psychiatry. 2003;64(3):288-94.

462. Rezayat AA, Hebrani P, Behdani F, Salaran M, Marvast MN. Comparison the effectiveness of aripiprazole and risperidone for the treatment of acute bipolar mania. Journal of Research in Medical Sciences. 2014;19(8):733-8.

463. Riesenberg RA, Baldytcheva I, Datto C. Self-reported sedation profile of quetiapine extended-release and quetiapine immediate-release during 6-day initial dose escalation in bipolar depression: a multicenter, randomized, double-blind, phase IV study. Clin Ther. 2012;34(11):2202-11.

464. Ritchie CW, Chiu E, Harrigan S, Hall K, Hassett A, MacFarlane S, et al. The impact upon extra-pyramidal side effects, clinical symptoms and quality of life of a switch from conventional to atypical antipsychotics (risperidone or olanzapine) in elderly patients with schizophrenia. International Journal of Geriatric Psychiatry. 2003;18(5):432-40.

465. Ritchie CW, Chiu E, Harrigan S, McFarlane S, Mastwyk M, Halliday G, et al. A comparison of the efficacy and safety of olanzapine and risperidone in the treatment of elderly patients with schizophrenia: An open study of six months duration. International Journal of Geriatric Psychiatry. 2006;21(2):171-9.

466. Robinson DG, Woerner MG, Napolitano B, Patel RC, Sevy SM, Gunduz-Bruce H, et al. Randomized comparison of olanzapine versus risperidone for the treatment of first-episode schizophrenia: 4-month outcomes. Am J Psychiatry. 2006;163(12):2096-102.

467. Roerig JL, Mitchell JE, de Zwaan M, Crosby RD, Gosnell BA, Steffen KJ, et al. A comparison of the effects of olanzapine and risperidone versus placebo on eating behaviors. J Clin Psychopharmacol. 2005;25(5):413-8.

468. Roerig JL, Steffen KJ, Mitchell JE, Crosby RD, Gosnell BA. A comparison of the effects of olanzapine and risperidone versus placebo on ghrelin plasma levels. J Clin Psychopharmacol. 2008;28(1):21-6.

469. Rosenheck R, Perlick D, Bingham S, Liu-Mares W, Collins J, Warren S, et al. Effectiveness and cost of olanzapine and haloperidol in the treatment of schizophrenia: a randomized controlled trial. Jama. 2003;290(20):2693-702.

470. Rothschild AJ, Williamson DJ, Tohen MF, Schatzberg A, Andersen SW, Van Campen LE, et al. A double-blind, randomized study of olanzapine and olanzapine/fluoxetine combination for major depression with psychotic features. Journal of Clinical Psychopharmacology. 2004;24(4):365-73.

471. Rui Q, Wang Y, Liang S, Liu Y, Wu Y, Wu Q, et al. Relapse prevention study of paliperidone extended-release tablets in Chinese patients with schizophrenia. Prog Neuropsychopharmacol Biol Psychiatry. 2014;53:45-53.

472. Ryan MCM, Flanagan S, Kinsella U, Keeling F, Thakore JH. The effects of atypical antipsychotics on visceral fat distribution in first episode, drug-naive patients with schizophrenia. Life Sciences. 2004;74(16):1999-2008.

473. Sacchetti E, Panariello A, Regini C, Valsecchi P. Quetiapine in hospitalized patients with schizophrenia refractory to treatment with first-generation antipsychotics: a 4-week, flexible-dose, single-blind, exploratory, pilot trial. Schizophr Res. 2004;69(2-3):325-31.

474. Sacchetti E, Galluzzo A, Valsecchi P, Romeo F, Gorini B, Warrington L. Ziprasidone vs clozapine in schizophrenia patients refractory to multiple antipsychotic treatments: the MOZART study. Schizophr Res. 2009;113(1):112-21.

475. Sacher J, Mossaheb N, Spindelegger C, Klein N, Geiss-Granadia T, Sauermann R, et al. Effects of olanzapine and ziprasidone on glucose tolerance in healthy volunteers. Neuropsychopharmacology. 2008;33(7):1633-41.

476. Saddichha S, Manjunatha N, Ameen S, Akhtar S. Effect of olanzapine, risperidone, and haloperidol treatment on weight and body mass index in first-episode schizophrenia patients in India: a randomized, double-blind, controlled, prospective study. J Clin Psychiatry. 2007;68(11):1793-8.

477. Saddichha S, Ameen S, Akhtar S. Predictors of antipsychotic-induced weight gain in first-episode psychosis: conclusions from a randomized, double-blind, controlled prospective study of olanzapine, risperidone, and haloperidol. J Clin Psychopharmacol. 2008;28(1):27-31.

478. Safa M, Sadr S, Delfan B, Saki M, Javad Tarrahi M. Metabolic effects of olanzapine and risperidone in patients with psychotic disorders. International Journal of Psychiatry in Clinical Practice. 2008;12(4):299-302.

479. Sajatovic M, Brescan DW, Perez DE, DiGiovanni SK, Hattab H, Ray JB, et al. Quetiapine alone and added to a mood stabilizer for serious mood disorders. J Clin Psychiatry. 2001;62(9):728-32.

480. Sajeev Kumar PB, Pandey RS, Thirthalli J, Siva Kumar PT, Naveen Kumar C. A comparative study of short term efficacy of aripiprazole and risperidone in schizophrenia. Current Neuropharmacology. 2017;15(8):1073-84.

481. San L, Arranz B, Perez V, Safont G, Corripio I, Ramirez N, et al. One-year, randomized, open trial comparing olanzapine, quetiapine, risperidone and ziprasidone effectiveness in antipsychotic-naive patients with a first-episode psychosis. Psychiatry Research. 2012;200(2-3):693-701.

482. Sanz-Fuentenebro J, Taboada D, Palomo T, Aragues M, Ovejero S, Del Alamo C, et al. Randomized trial of clozapine vs. risperidone in treatment-naive first-episode schizophrenia: Results after one year. Schizophr Res. 2013;149(1-3):156-61.

483. Sathirakul K, Chan C, Teng L, Bergstrom RF, Yeo KP, Wise SD. Olanzapine pharmacokinetics are similar in Chinese and Caucasian subjects. Br J Clin Pharmacol. 2003;56(2):184-7.

484. Schoemaker J, Stet L, Vrijland P, Naber D, Panagides J, Emsley R. Long-term efficacy and safety of asenapine or olanzapine in patients with schizophrenia or schizoaffective disorder: an extension study. Pharmacopsychiatry. 2012;45(5):196-203.

485. Schooler N, Rabinowitz J, Davidson M, Emsley R, Harvey PD, Kopala L, et al. Risperidone and haloperidol in first-episode psychosis: a long-term randomized trial. Am J Psychiatry. 2005;162(5):947-53.

486. Schooler NR, Marder SR, Chengappa KNR, Petrides G, Ames D, Wirshing WC, et al. Clozapine and risperidone in moderately refractory schizophrenia: A 6-Month randomized double-blind comparison. Journal of Clinical Psychiatry. 2016;77(5):628-34.

487. Sechter D, Peuskens J, Fleurot O, Rein W, Lecrubier Y. Amisulpride vs. risperidone in chronic schizophrenia: results of a 6-month double-blind study. Neuropsychopharmacology. 2002;27(6):1071-81.

488. Sevy S, Robinson DG, Sunday S, Napolitano B, Miller R, McCormack J, et al. Olanzapine vs. risperidone in patients with first-episode schizophrenia and a lifetime history of cannabis use disorders: 16-week clinical and substance use outcomes. Psychiatry Res. 2011;188(3):310-4.

489. Shafti SS, Kaviani H. A comparison between quetiapine and aripiprazole for treatment of schizophrenia: A double blind contrast. Current Psychopharmacology. 2016;5(1):13-9.

490. Shafti SS, Kaviani H. Adjunctive quetiapine may help fluvoxamine-resistant obsessive-compulsive disorder among female in-patients: A randomized-controlled study. Psychiatry and Clinical Psychopharmacology. 2019;29(2):171-7.

491. Sherwood Brown E, Davila D, Nakamura A, Carmody TJ, John Rush A, Lo A, et al. A randomized, double-blind, placebo-controlled trial of quetiapine in patients with bipolar disorder, mixed or depressed phase, and alcohol dependence. Alcoholism: Clinical and Experimental Research. 2014;38(7):2113-8.

492. Shoja Shafti S, Fallah Jahromi P. Olanzapine induced Q-Tc shortening. Therapeutic Advances in Psychopharmacology. 2014;4(6):240-6.

493. Shoja Shafti S, Kaviani H. Quetiapine versus aripiprazole in the management of schizophrenia. Therapeutic Advances in Psychopharmacology. 2015;5(3):166-71.

494. Shoja Shafti S, Kaviani H. Aripiprazole versus quetiapine in treatment-resistant obsessive-compulsive disorder: a double-blind clinical trial. Therapeutic Advances in Psychopharmacology. 2015;5(1):32-7.

495. Silva de Lima M, de Jesus Mari J, Breier A, Maria Costa A, Ponde de Sena E, Hotopf M. Quality of life in schizophrenia: a multicenter, randomized, naturalistic, controlled trial comparing olanzapine to first-generation antipsychotics. J Clin Psychiatry. 2005;66(7):831-8.

496. Simpson GM, Glick ID, Weiden PJ, Romano SJ, Siu CO. Randomized, controlled, double-blind multicenter comparison of the efficacy and tolerability of ziprasidone and olanzapine in acutely ill inpatients with schizophrenia or schizoaffective disorder. Am J Psychiatry. 2004;161(10):1837-47.

497. Simpson GM, Weiden P, Pigott T, Murray S, Siu CO, Romano SJ. Six-month, blinded, multicenter continuation study of ziprasidone versus olanzapine in schizophrenia. Am J Psychiatry. 2005;162(8):1535-8.

498. Singh S, Chandra S, Kapoor AK, Singh HK, Kant R. Metabolic effects of olanzapine versus iloperidone: A 24 weeks randomized, prospective, interventional study. Internet Journal of Medical Update. 2016;11(2):17-24.

499. Smeraldi E. Amisulpride versus fluoxetine in patients with dysthymia or major depression in partial remission. A double-blind, comparative study. J Affect Disorders. 1998;48(1):47-56.

500. Smith RC, Lindenmayer JP, Davis JM, Kelly E, Viviano TF, Cornwell J, et al. Effects of olanzapine and risperidone on glucose metabolism and insulin sensitivity in chronic schizophrenic patients with long-term antipsychotic treatment: a randomized 5-month study. J Clin Psychiatry. 2009;70(11):1501-13.

501. Smith RC, Lindenmayer JP, Hu Q, Kelly E, Viviano TF, Cornwell J, et al. Effects of olanzapine and risperidone on lipid metabolism in chronic schizophrenic patients with long-term antipsychotic treatment: a randomized five month study. Schizophr Res. 2010;120(1-3):204-9.

502. Smith RC, Rachakonda S, Dwivedi S, Davis JM. Olanzapine and risperidone effects on appetite and ghrelin in chronic schizophrenic patients. Psychiatry Res. 2012;199(3):159-63.

503. Sowell MO, Mukhopadhyay N, Cavazzoni P, Shankar S, Steinberg HO, Breier A, et al. Hyperglycemic clamp assessment of insulin secretory responses in normal subjects treated with olanzapine, risperidone, or placebo. J Clin Endocrinol Metab. 2002;87(6):2918-23.

504. Sowell M, Mukhopadhyay N, Cavazzoni P, Carlson C, Mudaliar S, Chinnapongse S, et al. Evaluation of insulin sensitivity in healthy volunteers treated with olanzapine, risperidone, or placebo: a prospective, randomized study using the two-step hyperinsulinemic, euglycemic clamp. J Clin Endocrinol Metab. 2003;88(12):5875-80.

505. Spivak B, Musin E, Mester R, Gonen N, Talmon Y, Guy N, et al. The effect of long-term antipsychotic treatment on the body weight of patients suffering from chronic schizophrenia: clozapine versus classical antipsychotic agents. Int Clin Psychopharmacol. 1999;14(4):229-32.

506. Stone WS, Hsi X, Giuliano AJ, Tan L, Zhu S, Li L, et al. Are neurocognitive, clinical and social dysfunctions in schizotaxia reversible pharmacologically? Results from the Changsha study. Asian Journal of Psychiatry. 2012;5(1):73-82.

507. Su Y, Yan H, Guo L, Lu T, Zhang D, Yue W. Association of MTHFR C677T Polymorphism With Antipsychotic-Induced Change of Weight and Metabolism Index. Frontiers in Psychiatry. 2021;12 (no pagination).

508. Sumiyoshi T, Jayathilake K, Meltzer HY. A comparison of two doses of melperone, an atypical antipsychotic drug, in the treatment of schizophrenia. Schizophr Res. 2003;62(1-2):65-72.

509. Suppes T, McElroy SL, Sheehan DV, Hidalgo RB, Cosgrove VE, Gwizdowski IS, et al. A randomized, double-blind, placebo-controlled study of ziprasidone monotherapy in bipolar disorder with co-occurring lifetime panic or generalized anxiety disorder. Journal of Clinical Psychiatry. 2014;75(1):77-84.

510. Suresh Kumar PN, Anish PK, Rajmohan V. Olanzapine has better efficacy compared to risperidone for treatment of negative symptoms in schizophrenia. Indian Journal of Psychiatry. 2016;58(3):311-6.

511. Swadi HS, Craig BJ, Pirwani NZ, Black VC, Buchan JC, Bobier CM. A trial of quetiapine compared with risperidone in the treatment of first onset psychosis among 15- to 18-year-old adolescents. Int Clin Psychopharmacol. 2010;25(1):1-6.

512. Swartz HA, Rucci P, Thase ME, Wallace M, Carretta E, Celedonia KL, et al. Psychotherapy Alone and Combined With Medication as Treatments for Bipolar II Depression: A Randomized Controlled Trial. J Clin Psychiatry. 2018;79(2).

513. Takekita Y, Kato M, Wakeno M, Sakai S, Suwa A, Nishida K, et al. A 12-week randomized, open-label study of perospirone versus aripiprazole in the treatment of Japanese schizophrenia patients. Prog Neuropsychopharmacol Biol Psychiatry. 2013;40:110-4.

514. Tandon R, Cucchiaro J, Phillips D, Hernandez D, Mao Y, Pikalov A, et al. A double-blind, placebo-controlled, randomized withdrawal study of lurasidone for the maintenance of efficacy in patients with schizophrenia. J Psychopharmacol. 2016;30(1):69-77.

515. Tapp A, Wood AE, Kennedy A, Sylvers P, Kilzieh N, Saxon AJ. Quetiapine for the treatment of cocaine use disorder. Drug and Alcohol Dependence. 2015;149:18-24.

516. Teff KL, Rickels MR, Grudziak J, Fuller C, Nguyen HL, Rickels K. Antipsychotic-induced insulin resistance and postprandial hormonal dysregulation independent of weight gain or psychiatric disease. Diabetes. 2013;62(9):3232-40.

517. Thase ME, Corya SA, Osuntokun O, Case M, Henley DB, Sanger TM, et al. A randomized, double-blind comparison of olanzapine/fluoxetine combination, olanzapine, and fluoxetine in treatment-resistant major depressive disorder. J Clin Psychiatry. 2007;68(2):224-36.

518. Tohen M, Sanger TM, McElroy SL, Tollefson GD, Chengappa KN, Daniel DG, et al. Olanzapine versus placebo in the treatment of acute mania. Olanzapine HGEH Study Group. Am J Psychiatry. 1999;156(5):702-9.

519. Tohen M, Baker RW, Altshuler LL, Zarate CA, Suppes T, Ketter TA, et al. Olanzapine versus divalproex in the treatment of acute mania. Am J Psychiatry. 2002;159(6):1011-7.

520. Tohen M, Jacobs TG, Grundy SL, McElroy SL, Banov MC, Janicak PG, et al. Efficacy of olanzapine in acute bipolar mania: a double-blind, placebo-controlled study. The Olanzipine HGGW Study Group. Arch Gen Psychiatry. 2000;57(9):841-9.

521. Tollefson GD, Birkett MA, Kiesler GM, Wood AJ. Double-blind comparison of olanzapine versus clozapine in schizophrenic patients clinically eligible for treatment with clozapine. Biol Psychiatry. 2001;49(1):52-63.

522. Tran PV, Hamilton SH, Kuntz AJ, Potvin JH, Andersen SW, Beasley Jr C, et al. Double-blind comparison of olanzapine versus risperidone in the treatment of schizophrenia and other psychotic disorders. Journal of Clinical Psychopharmacology. 1997;17(5):407-18.

523. Tran PV, Tollefson GD, Sanger TM, Lu Y, Berg PH, Beasley CM, Jr. Olanzapine versus haloperidol in the treatment of schizoaffective disorder. Acute and long-term therapy. Br J Psychiatry. 1999;174:15-22.

524. Tunis SL, Faries DE, Nyhuis AW, Kinon BJ, Ascher-Svanum H, Aquila R. Cost-effectiveness of olanzapine as first-line treatment for schizophrenia: Results from a randomized, open-label, I-year trial. Value in Health. 2006;9(2):77-89.

525. Tybura P, Trześniowska-Drukała B, Bienkowski P, Beszlej A, Frydecka D, Mierzejewski P, et al. Pharmacogenetics of adverse events in schizophrenia treatment: comparison study of ziprasidone, olanzapine and perazine. Psychiatry Res. 2014;219(2):261-7.

526. Tyrer P, Oliver-Africano P, Romeo R, Knapp M, Dickens S, Bouras N, et al. Neuroleptics in the treatment of aggressive challenging behaviour for people with intellectual disabilities: A randomised controlled trial (NACHBID). Health Technology Assessment. 2009;13(21):1-54.

527. Tzimos A, Samokhvalov V, Kramer M, Ford L, Gassmann-Mayer C, Lim P, et al. Safety and tolerability of oral paliperidone extended-release tablets in elderly patients with schizophrenia: a double-blind, placebo-controlled study with six-month open-label extension. Am J Geriatr Psychiatry. 2008;16(1):31-43.

528. van Bruggen J, Tijssen J, Dingemans P, Gersons B, Linszen D. Symptom response and side-effects of olanzapine and risperidone in young adults with recent onset schizophrenia. Int Clin Psychopharmacol. 2003;18(6):341-6.

529. Vázquez-Bourgon J, Pérez-Iglesias R, Ortiz-García de la Foz V, Suárez Pinilla P, Díaz Martínez Á, Crespo-Facorro B. Long-term metabolic effects of aripiprazole, ziprasidone and quetiapine: A pragmatic clinical trial in drug-naïve patients with a first-episode of non-affective psychosis. Psychopharmacology. 2018;235(1):245-55.

530. Vazquez-Bourgon J, Ibanez Alario M, Mayoral-van Son J, Gomez Revuelta M, Ayesa Arriola R, Juncal Ruiz M, et al. A 3-year prospective study on the metabolic effect of aripiprazole, quetiapine and ziprasidone: A pragmatic clinical trial in first episode psychosis patients. European Neuropsychopharmacology. 2020;39:46-55.

531. Verhey FRJ, Verkaaik M, Lousberg R. Olanzapine versus haloperidol in the treatment of agitation in elderly patients with dementia: Results of a randomized controlled double-blind trial. Dementia and Geriatric Cognitive Disorders. 2006;21(1):1-8.

532. Vidarsdottir S, de Leeuw van Weenen JE, Frolich M, Roelfsema F, Romijn JA, Pijl H. Effects of olanzapine and haloperidol on the metabolic status of healthy men. J Clin Endocrinol Metab. 2010;95(1):118-25.

533. Vieta E, Bourin M, Sanchez R, Marcus R, Stock E, McQuade R, et al. Effectiveness of aripiprazole v. haloperidol in acute bipolar mania: double-blind, randomised, comparative 12-week trial. Br J Psychiatry. 2005;187:235-42.

534. Volavka J, Czobor P, Sheitman B, Lindenmayer J-P, Citrome L, McEvoy JP, et al. Clozapine, olanzapine, risperidone, and haloperidol in the treatment of patients with chronic schizophrenia and schizoaffective disorder. The American Journal of Psychiatry. 2002;159(2):255-62.

535. Voruganti LP, Awad AG, Parker G, Forrest C, Usmani Y, Fernando MLD, et al. Cognition, functioning and quality of life in schizophrenia treatment: Results of a one-year randomized controlled trial of olanzapine and quetiapine. Schizophr Res. 2007;96(1-3):146-55.

536. Wampers M, Hanssens L, van Winkel R, Heald A, Collette J, Peuskens J, et al. Differential effects of olanzapine and risperidone on plasma adiponectin levels over time: results from a 3-month prospective open-label study. Eur Neuropsychopharmacol. 2012;22(1):17-26.

537. Wang X, Savage R, Borisov A, Rosenberg J, Woolwine B, Tucker M, et al. Efficacy of risperidone versus olanzapine in patients with schizophrenia previously on chronic conventional antipsychotic therapy: a switch study. J Psychiatr Res. 2006;40(7):669-76.

538. Wang CY, Xiang YT, Cai ZJ, Weng YZ, Bo QJ, Zhao JP, et al. Risperidone maintenance treatment in schizophrenia: A randomized, controlled trial. American Journal of Psychiatry. 2010;167(6):676-85.

539. Wang M, Tong JH, Huang DS, Zhu G, Liang GM, Du H. Efficacy of olanzapine monotherapy for treatment of bipolar I depression: a randomized, double-blind, placebo controlled study. Psychopharmacology (Berl). 2014;231(14):2811-8.

540. Wang HH, Cai M, Wang HN, Chen YC, Zhang RG, Wang Y, et al. An assessor-blinded, randomized comparison of efficacy and tolerability of switching from olanzapine to ziprasidone and the combination of both in schizophrenia spectrum disorders. J Psychiatr Res. 2017;85:59-65.

541. Weiden PJ, Claxton A, Kunovac J, Walling DP, Du Y, Yao B, et al. Efficacy and safety of a 2-month formulation of aripiprazole lauroxil with 1-day initiation in patients hospitalized for acute schizophrenia transitioned to outpatient care: Phase 3, randomized, double-blind, active-control ALPINE study. Journal of Clinical Psychiatry. 2020;81(3).

542. Weiden PJ, Du Y, von Moltke L, Wehr A, Hard M, Marandi M, et al. Pharmacokinetics, Safety, and Tolerability of a 2-Month Dose Interval Regimen of the Long-Acting Injectable Antipsychotic Aripiprazole Lauroxil: Results From a 44-Week Phase I Study. CNS Drugs. 2020;34(9):961-72.

543. Werapongset W, Chaisirikul S, Chrujiporn W, Visanuyothin T, Kessawai D, Charisilp C, et al. Efficacy and tolerability of risperidone in chronic schizophrenic Thai patients. J Med Assoc Thai. 1998;81(5):324-8.

544. Wilner KD, Tensfeldt TG, Baris B, Smolarek TA, Turncliff RZ, Colburn WA, et al. Single- and multiple-dose pharmacokinetics of ziprasidone in healthy young and elderly volunteers. Br J Clin Pharmacol. 2000;49 Suppl 1:15s-20s.

545. Woods SW, Breier A, Zipursky RB, Perkins DO, Addington J, Miller TJ, et al. Randomized trial of olanzapine versus placebo in the symptomatic acute treatment of the schizophrenic prodrome. Biol Psychiatry. 2003;54(4):453-64.

546. Wu R-R, Zhao J-P, Zhai J-G, Guo X-F, Guo W-B. Sex difference in effects of typical and atypical antipsychotics on glucose-insulin homeostatic and lipid metabolism in first-episode schizophrenia. Journal of Clinical Psychopharmacology. 2007;27(4):374-9.

547. Xu L, Lu Y, Yang Y, Zheng Y, Chen F, Lin Z. Olanzapine-valproate combination versus olanzapine or valproate monotherapy in the treatment of bipolar imania: A randomized controlled study in a chinese population group. Neuropsychiatric Disease and Treatment. 2015;11:1265-71.

548. Xue X, Song Y, Yu X, Fan Q, Tang J, Chen X. Olanzapine and haloperidol for the treatment of acute symptoms of mental disorders induced by amphetamine-type stimulants. Medicine (United States). 2018;97(8):e9786.

549. Yatham LN, Fallu A, Binder CE. A 6-month randomized open-label comparison of continuation of oral atypical antipsychotic therapy or switch to long acting injectable risperidone in patients with bipolar disorder. Acta Psychiatr Scand Suppl. 2007(434):50-6.

550. Yung AR, Phillips LJ, Nelson B, Francey SM, PanYuen H, Simmons MB, et al. Randomized controlled trial of interventions for young people at ultra high risk for psychosis: 6-Month analysis. Journal of Clinical Psychiatry. 2011;72(4):430-40.

551. Zajecka JM, Weisler R, Sachs G, Swann AC, Wozniak P, Sommerville KW. A comparison of the efficacy, safety, and tolerability of divalproex sodium and olanzapine in the treatment of bipolar disorder. J Clin Psychiatry. 2002;63(12):1148-55.

552. Zanardi R, Smeraldi E. A double-blind, randomised, controlled clinical trial of acetyl-l-carnitine vs. amisulpride in the treatment of dysthymia. European Neuropsychopharmacology. 2006;16(4):281-7.

553. Zanarini MC, Frankenburg FR. Olanzapine treatment of female borderline personality disorder patients: a double-blind, placebo-controlled pilot study. J Clin Psychiatry. 2001;62(11):849-54.

554. Zanarini MC, Frankenburg FR, Parachini EA. A preliminary, randomized trial of fluoxetine, olanzapine, and the olanzapine-fluoxetine combination in women with borderline personality disorder. J Clin Psychiatry. 2004;65(7):903-7.

555. Zarcone JR, Hellings JA, Crandall K, Matthew Reese R, Marquis J, Fleming K, et al. Effects of risperidone on aberrant behavior of persons with developmental disabilities: I. A double-blind crossover study using multiple measures. American Journal on Mental Retardation. 2001;106(6):525-38.

556. Zhang Y, Dai G. Efﬁcacy and metabolic influence of paliperidone ER, aripiprazole and ziprasidone to patients with first-episode schizophrenia through 52 weeks follow-up in China. Hum Psychopharmacol. 2012;27(6):605-14.

557. Zimbroff D, Warrington L, Loebel A, Yang R, Siu C. Comparison of ziprasidone and aripiprazole in acutely ill patients with schizophrenia or schizoaffective disorder: a randomized, double-blind, 4-week study. Int Clin Psychopharmacol. 2007;22(6):363-70.

# Appendix 5: CRWG

## Appendix 5.1: Results meta-analysis CRWG

| **Antipsychotic** | **Time (weeks)** | **N studies** | **n** | **% CRWG** | **95% CI** | **I^22^** | **Significance test Z** | ***p*** |
| --- | --- | --- | --- | --- | --- | --- | --- | --- |
| Amisulpride | 6-16 wk | 4 | 339 | 13.3 | 9.8 - 17.2 | 0.00% | 11.719 | <0.001 |
| Amisulpride | 16-38 wk | 1 | 189 | 20.6 | 15.1 - 27.1 |  | 12.035 | <0.001 |
| Aripiprazole | <6 wk | 19 | 2171 | 5.5 | 3.7 - 7.5 | 70.13% | 9.524 | <0.001 |
| Aripiprazole | 6-16 wk | 19 | 2683 | 6.7 | 4.1 – 10.0 | 89.07% | 7.657 | <0.001 |
| Aripiprazole | 16-38 wk | 12 | 2257 | 11.7 | 8.7 - 14.9 | 79.37% | 12.725 | <0.001 |
| Aripiprazole | >38 wk | 7 | 1843 | 17.2 | 11.0 - 24.3 | 91.44% | 8.742 | <0.001 |
| Asenapine | <6 wk | 13 | 1587 | 4.7 | 3.3 - 6.3 | 43.01% | 10.607 | <0.001 |
| Asenapine | 16-38 wk | 8 | 982 | 8.0 | 5.6 - 10.7 | 44.93% | 10.182 | <0.001 |
| Asenapine | >38 wk | 2 | 987 | 16.4 | 14.2 - 18.8 |  | 23.833 | <0.001 |
| Blonanserin | <6 wk | 3 | 183 | 1.7 | 0.0 - 5.0 |  | 2.095 | 0.036 |
| Blonanserin | 6-16 wk | 2 | 220 | 9.4 | 5.8 - 13.7 |  | 8.037 | <0.001 |
| Blonanserin | 16-38 wk | 1 | 22 | 18.2 | 5.2 - 40.3 |  | 3.345 | 0.001 |
| Brexpiprazole | <6 wk | 10 | 1584 | 7.2 | 5.2 - 9.4 | 60.20% | 11.752 | <0.001 |
| Brexpiprazole | 6-16 wk | 3 | 430 | 1.9 | 0.7 - 3.4 |  | 4.412 | <0.001 |
| Brexpiprazole | >38 wk | 1 | 264 | 5.3 | 2.9 - 8.7 |  | 6.679 | <0.001 |
| Cariprazine | <6 wk | 11 | 1650 | 5.3 | 3.4 - 7.5 | 69.72% | 8.835 | <0.001 |
| Cariprazine | 6-16 wk | 5 | 583 | 4.8 | 3.0 - 6.9 | 12.38% | 8.165 | <0.001 |
| Cariprazine | 16-38 wk | 1 | 101 | 26.7 | 18.4 -36.5 |  | 10.001 | <0.001 |
| Chlorpromazine | <6 wk | 2 | 137 | 19.5 | 13.2 - 26.7 |  | 9.320 | <0.001 |
| Chlorpromazine | 6-16 wk | 1 | 154 | 13.6 | 8.6 - 20.1 |  | 8.487 | <0.001 |
| Clozapine | <6 wk | 1 | 40 | 27.5 | 14.6 - 43.9 |  | 6.105 | <0.001 |
| Clozapine | 6-16 wk | 2 | 91 | 40.0 | 30.1 - 50.4 |  | 11.729 | <0.001 |
| Clozapine | 16-38 wk | 2 | 89 | 47.0 | 36.6 - 57.5 |  | 12.925 | <0.001 |
| Clozapine | >38 wk | 2 | 80 | 76.3 | 66.2 - 85.2 |  | 17.587 | <0.001 |
| FGA | <6 wk | 1 | 45 | 6.7 | 1.4 - 18.3 |  | 2.763 | 0.006 |
| FGA | 6-16 wk | 2 | 134 | 17.6 | 11.3 - 24.8 |  | 8.208 | <0.001 |
| FGA | 16-38 wk | 2 | 163 | 32.3 | 25.3 - 39.8 |  | 13.976 | <0.001 |
| FGA | >38 wk | 3 | 208 | 49.0 | 42.2 - 55.9 |  | 20.550 | <0.001 |
| Haloperidol | <6 wk | 8 | 1166 | 3.6 | 2.3 - 5.2 | 18.35% | 7.970 | <0.001 |
| Haloperidol | 6-16 wk | 5 | 572 | 9.1 | 3.2 - 17.3 | 86.83% | 4.231 | <0.001 |
| Haloperidol | >38 wk | 3 | 599 | 22.8 | 7.8 - 42.5 |  | 4.164 | <0.001 |
| Iloperidone | <6 wk | 1 | 300 | 21.0 | 16.5 - 26.1 |  | 15.545 | <0.001 |
| Iloperidone | 6-16 wk | 2 | 500 | 8.6 | 6.3 -11.2 |  | 12.021 | <0.001 |
| Iloperidone | >38 wk | 1 | 151 | 25.2 | 18.5 – 32.9 |  | 11.983 | <0.001 |
| Lurasidone | <6 wk | 20 | 2628 | 3.6 | 2.5 – 4.9 | 58.02% | 10.207 | <0.001 |
| Lurasidone | >38 wk | 1 | 419 | 7.4 | 5.1 - 10.3 |  | 10.363 | <0.001 |
| Olanzapine | <6 wk | 23 | 3533 | 17.5 | 13.8 - 21.5 | 85.96% | 14.619 | <0.001 |
| Olanzapine | 6-16 wk | 29 | 3691 | 25.2 | 20.6 - 30.1 | 90.51% | 17.116 | <0.001 |
| Olanzapine | 16-38 wk | 19 | 3954 | 23.8 | 18.7 - 29.2 | 92.73% | 14.862 | <0.001 |
| Olanzapine | >38 wk | 14 | 2829 | 36.9 | 30.4 - 43.6 | 92.35% | 17.681 | <0.001 |
| Paliperidone | <6 wk | 16 | 1822 | 6.6 | 4.6 - 8.9 | 69.92% | 10.176 | <0.001 |
| Paliperidone | 6-16 wk | 10 | 1094 | 8.4 | 6.8 - 10.2 | 0.00% | 16.368 | <0.001 |
| Paliperidone | 16-38 wk | 6 | 991 | 15.5 | 9.4 - 22.8 | 88.31% | 7.836 | <0.001 |
| Paliperidone | >38 wk | 7 | 2192 | 18.3 | 14.2 - 22.7 | 84.34% | 14.563 | <0.001 |
| Quetiapine | <6 wk | 22 | 2634 | 9.5 | 6.4 - 13.1 | 87.94% | 9.373 | <0.001 |
| Quetiapine | 6-16 wk | 25 | 4042 | 7.0 | 5.0 - 9.2 | 84.27% | 11.177 | <0.001 |
| Quetiapine | 16-38 wk | 4 | 688 | 10.1 | 7.6 - 12.8 | 7.68% | 12.739 | <0.001 |
| Quetiapine | >38 wk | 4 | 512 | 17.0 | 9.9 - 25.4 | 71.04% | 7.119 | <0.001 |
| Risperidone | <6 wk | 10 | 973 | 10.6 | 7.6 - 13.9 | 58.36% | 11.109 | <0.001 |
| Risperidone | 6-16 wk | 19 | 2020 | 12.4 | 7.6 - 18.1 | 91.42% | 7.746 | <0.001 |
| Risperidone | 16-38 wk | 6 | 421 | 17.4 | 13.1 - 22.1 | 28.14% | 12.583 | <0.001 |
| Risperidone | >38 wk | 12 | 1960 | 23.0 | 17.5 - 28.9 | 88.14% | 13.178 | <0.001 |
| Sertindole | 6-16 wk | 1 | 205 | 24.9 | 19.1 - 31.4 |  | 14.012 | <0.001 |
| Sertindole | >38 wk | 1 | 131 | 16.8 | 10.8 - 24.3 |  | 8.761 | <0.001 |
| SGA | 6-16 wk | 1 | 109 | 20.2 | 13.1 - 28.9 |  | 8.822 | <0.001 |
| SGA | 16-38 wk | 3 | 506 | 29.5 | 15.0 - 46.4 |  | 6.005 | <0.001 |
| SGA | >38 wk | 2 | 134 | 37.1 | 28.9 - 45.6 |  | 13.477 | <0.001 |
| Ziprasidone | <6 wk | 2 | 282 | 2.5 | 0.9 - 4.8 |  | 4.283 | <0.001 |
| Ziprasidone | 6-16 wk | 5 | 462 | 8.7 | 3.8 - 15.1 | 75.32% | 5.065 | <0.001 |
| Ziprasidone | 16-38 wk | 2 | 219 | 2.8 | 0.8 - 5.7 |  | 3.595 | <0.001 |
| Ziprasidone | >38 wk | 1 | 185 | 7.6 | 4.2 - 12.4 |  | 6.707 | <0.001 |
| Placebo | <6 wk | 59 | 7270 | 2.0 | 1.4 - 2.6 | 62.29% | 11.086 | <0.001 |
| Placebo | 6-16 wk | 37 | 5597 | 2.8 | 1.9 - 3.8 | 74.48% | 9.866 | <0.001 |
| Placebo | 16-38 wk | 10 | 1333 | 7.4 | 2.4 - 14.5 | 94.42% | 4.070 | <0.001 |
| Placebo | >38 wk | 9 | 1057 | 6.7 | 3.2 - 11.2 | 84.79% | 5.541 | <0.001 |
|  |  |  |  |  |  |  |  |  |

## Appendix 5.2: Forest plots of CRWG

### Supplementary file 5.2.1: Amisulpride

### Supplementary file 5.2.2: Aripiprazole

### Supplementary file 5.2.3: Asenapine

### Supplementary file 5.2.4: Blonanserin

### Supplementary file 5.2.5: Brexpiprazole

### Supplementary file 5.2.6: Cariprazine

### Supplementary file 5.2.7: Chlorpromazine

### Supplementary file 5.2.8: Clozapine

### Supplementary file 5.2.9: FGA

### Supplementary file 5.2.10: Haloperidol

### Supplementary file 5.2.11: Iloperidone

### Supplementary file 5.2.12: Lurasidone

### Supplementary file 5.2.13: Olanzapine

### Supplementary file 5.2.14: Paliperidone

### Supplementary file 5.2.15: Placebo

### Supplementary file 5.2.16: Quetiapine

### Supplementary file 5.2.17: Risperidone

### Supplementary file 5.2.18: Sertindole

### Supplementary file 5.2.19: SGA

### Supplementary file 5.2.20: Ziprasidone

# Appendix 6: CRWL

## Appendix 6.1: Results meta-analysis CRWL

| **Antipsychotic** | **Time (weeks)** | **N studies** | **n** | **% CRWL** | **95% CI** | **I^2^** | **Significance test Z** | ***p*** |
| --- | --- | --- | --- | --- | --- | --- | --- | --- |
| Aripiprazole | <6 wk | 1 | 122 | 4.9 | 1.8 -10.4 |  | 4.132 | <0.001 |
| Aripiprazole | 6-16 wk | 12 | 1708 | 8.8 | 6.5 - 11.5 | 68.52% | 12.097 | <0.001 |
| Aripiprazole | 16-38 wk | 7 | 1326 | 9.7 | 6.1- 14.0 | 82.90% | 8.273 | <0.001 |
| Aripiprazole | >38 wk | 2 | 157 | 9.1 | 4.8 - 14.4 |  | 6.153 | <0.001 |
| Asenapine | <6 wk | 4 | 599 | 1.5 | 0.6 - 2.7 | 0.00% | 4.511 | <0.001 |
| Asenapine | 16-38 wk | 3 | 679 | 5.8 | 1.9 - 11.6 |  | 4.100 | <0.001 |
| Asenapine | >38 wk | 1 | 908 | 5.0 | 3.6 - 6.6 |  | 12.602 | <0.001 |
| Lurasidone | <6 wk | 5 | 718 | 1.4 | 0.6 - 2.5 | 0.00% | 4.874 | <0.001 |
| Lurasidone | >38 wk | 1 | 419 | 12.6 | 9.6 - 16.2 |  | 13.950 | <0.001 |
| Olanzapine | <6 wk | 5 | 1985 | 1.2 | 0.2 - 2.6 | 67.84% | 3.236 | 0.001 |
| Olanzapine | 6-16 wk | 2 | 274 | 0.3 | 0.0 - 1.5 |  | 1.061 | 0.289 |
| Olanzapine | 16-38 wk | 6 | 1234 | 4.8 | 3.3 - 6.7 | 47.66% | 9.559 | <0.001 |
| Olanzapine | >38 wk | 4 | 1135 | 10.8 | 4.1 - 20.0 | 94.86% | 4.691 | <0.001 |
| Paliperidone | <6 wk | 8 | 855 | 2.0 | 1.1 - 3.1 | 0.00% | 6.261 | <0.001 |
| Paliperidone | 6-16 wk | 2 | 163 | 1.8 | 0.1 - 4.7 |  | 2.515 | 0.012 |
| Paliperidone | 16-38 wk | 2 | 241 | 4.9 | 2.5 – 8.1 |  | 5.803 | <0.001 |
| Paliperidone | >38 wk | 2 | 995 | 5.7 | 4.4 - 7.3 |  | 13.961 | <0.001 |
| Quetiapine | 16-38 wk | 1 | 175 | 9.1 | 5.3 - 14.4 |  | 7.245 | <0.001 |
| Quetiapine | >38 wk | 1 | 21 | 4.8 | 0.1 - 23.8 |  | 1.420 | 0.156 |
| Risperidone | <6 wk | 1 | 116 | 0.0 | 0.0 - 3.1 |  | 0.000 | 1.000 |
| Risperidone | 6-16 wk | 3 | 380 | 1.4 | 0.0 - 5.9 |  | 1.423 | 0.155 |
| Risperidone | >38 wk | 2 | 449 | 5.8 | 3.8 - 8.2 |  | 9.062 | <0.001 |
| Ziprasidone | 6-16 wk | 3 | 361 | 8.5 | 5.7 - 11.6 |  | 9.614 | <0.001 |
| Ziprasidone | >38 wk | 1 | 28 | 28.6 | 13.2 - 48.7 |  | 5.109 | <0.001 |
| Placebo | <6 wk | 11 | 1311 | 2.3 | 0.7 – 4.5 | 78.79% | 3.898 | <0.001 |
| Placebo | 6-16 wk | 6 | 811 | 5.3 | 2.8 – 8.6 | 69.34% | 6.119 | <0.001 |
| Placebo | 16-38 wk | 4 | 526 | 11.1 | 8.5 – 14.0 | 2.28% | 13.549 | <0.001 |
| Placebo | >38 wk | 1 | 133 | 12.0 | 7.0 - 18.8 |  | 7.285 | <0.001 |
|  |  |  |  |  |  |  |  |  |

## Appendix 6.2: Forest plots of CRWL

### Supplementary file 6.2.1: Aripiprazole

### Supplementary file 6.2.2: Asenapine

### Supplementary file 6.2.3: Lurasidone

### Supplementary file 6.2.4: Olanzapine

### Supplementary file 6.2.5: Paliperidone

### Supplementary file 6.2.6: Placebo

### Supplementary file 6.2.7: Quetiapine

### Supplementary file 6.2.8: Risperidone

### Supplementary file 6.2.9: Ziprasidone

# Appendix 7. Results for AP with data for only 1 period

## Supplementary table 7.1. CRWG for AP with data for only 1 period

| **Antipsychotic** | **Time (weeks)** | **N studies** | **n** | **% CRWG** | **95% CI** | **I^2^** | **Significance test Z** | ***p*** |
| --- | --- | --- | --- | --- | --- | --- | --- | --- |
| LY2140023 | 16-38 wk | 2 | 641 | 4.8 | 3.2 – 6.6 |  | 9.673 | <0.001 |

Time is in weeks. N= number of studies. n = number of patients included in the study.

## Supplementary table 7.2. CRWL for AP with data for only 1 period

| **Antipsychotic** | **Time (weeks)** | **N studies** | **n** | **% CRWL** | **95% CI** | **I^2^** | **Significance test Z** | ***p*** |
| --- | --- | --- | --- | --- | --- | --- | --- | --- |
| Amisulpride | 6-16 wk | 2 | 105 | 6.4 | 2.3 – 12.2 |  | 4.180 | <0.001 |
| Haloperidol | <6 wk | 2 | 775 | 3.4 | 2.2 – 4.8 |  | 8.665 | <0.001 |
| LY2140023 | 16-38 wk | 2 | 641 | 13.3 | 10.8 – 16.1 |  | 17.325 | <0.001 |
| SGA | 16-38 wk | 2 | 397 | 6.4 | 4.1 – 9.0 |  | 8.870 | <0.001 |

Time is in weeks. N= number of studies. n = number of patients included in the study.

# Appendix 8: Diagnosis

## Supplementary table 8.1: Overview psychiatric diagnoses of included studies and grouped diagnoses

| **Study diagnosis** | **Group diagnosis** |
| --- | --- |
| Alzheimer | Dementia |
| Dementia | Dementia |
| Bipolar | Bipolar Disorder |
| Bipolar I | Bipolar Disorder |
| Bipolar I II | Bipolar Disorder |
| Bipolar I II NOS | Bipolar Disorder |
| Borderline | Other diagnosis |
| GAD | Other diagnosis |
| MDD | Other diagnosis |
| Schizophrenia | Schizophrenia |
| Schizoaffective Disorder | Schizophrenia Spectrum |
| Schizophrenia, Depression | Schizophrenia Spectrum |
| Schizophrenia, Schizoaffective Disorder | Schizophrenia Spectrum |
| Schizophrenia, Schizoaffective Disorder, other psychotic disorder, Bipolar | Schizophrenia Spectrum |
| Schizophrenia, Schizoaffective, Bipolar | Schizophrenia Spectrum |
| Schizophrenia, Schizoaffective, Bipolar I | Schizophrenia Spectrum |
| Schizophrenia, Schizoaffective, Schizofreniform | Schizophrenia Spectrum |
| Schizophrenia, Schizophrenia Spectrum Disorder | Schizophrenia Spectrum |
| Schizophrenia, Schizoaffective, Schizofreniform, Delusional Disorder | Schizophrenia Spectrum |

# Appendix 9: Sensitivity analyses

## Supplementary table 9.1: Sensitivity meta-analysis CRWG without AP-naive population

| **Antipsychotic** | **Time (wk)** | **N studies** | **n** | **% CRWG** | **95% CI** | **I^2^** | **Significance test Z** | ***p*** |
| --- | --- | --- | --- | --- | --- | --- | --- | --- |
| Amisulpride | 6–16 wk | 4 | 339 | 13.3 | 9.8 – 17.2 | 0.00% | 11.719 | <0.001 |
| Amisulpride | 16–38 wk | 1 | 189 | 20.6 | 15.1 – 27.1 |  | 12.035 | <0.001 |
| **Aripiprazole** | **<6 wk** | **18** | **2010** | **5.3** | **3.5 – 7.4** | **70.83%** | **8.876** | **<0.001** |
| **Aripiprazole** | **6–16 wk** | **17** | **2452** | **5.1** | **3.3 – 7.2** | **77.54%** | **8.906** | **<0.001** |
| **Aripiprazole** | **16–38 wk** | **11** | **2235** | **11.8** | **8.8 – 15.2** | **81.21%** | **12.742** | **<0.001** |
| Aripiprazole | >38 wk | 7 | 1843 | 17.2 | 11.0 – 24.3 | 91.44% | 8.742 | <0.001 |
| Asenapine | <6 wk | 13 | 1587 | 4.7 | 3.3 – 6.3 | 43.01% | 10.607 | <0.001 |
| Asenapine | 16–38 wk | 8 | 982 | 8.0 | 5.6 – 10.7 | 44.93% | 10.182 | <0.001 |
| Asenapine | >38 wk | 2 | 987 | 16.4 | 14.2 – 18.8 |  | 23.833 | <0.001 |
| Blonanserin | <6 wk | 3 | 183 | 1.7 | 0.0 – 5.0 |  | 2.095 | 0.036 |
| Blonanserin | 6–16 wk | 2 | 220 | 9.4 | 5.8– 13.7 |  | 8.037 | <0.001 |
| **Blonanserin** | **16–38 wk** | **0** |  |  |  |  |  |  |
| Brexpiprazole | <6 wk | 10 | 1584 | 7.2 | 5.2 – 9.4 | 60.20% | 11.752 | <0.001 |
| Brexpiprazole | 6–16 wk | 3 | 430 | 1.9 | 0.7 – 3.4 |  | 4.412 | <0.001 |
| Brexpiprazole | >38 wk | 1 | 264 | 5.3 | 2.9– 8.7 |  | 6.679 | <0.001 |
| Cariprazine | <6 wk | 11 | 1650 | 5.3 | 3.4 – 7.5 | 69.72% | 8.835 | <0.001 |
| Cariprazine | 6–16 wk | 5 | 583 | 4.8 | 3.0 – 6.9 | 12.38% | 8.165 | <0.001 |
| Cariprazine | 16–38 wk | 1 | 101 | 26.7 | 18.4 – 36.5 |  | 10.001 | <0.001 |
| Chlorpromazine | <6 wk | 2 | 137 | 19.5 | 13.2 – 26.7 |  | 9.320 | <0.001 |
| Chlorpromazine | 6–16 wk | 1 | 154 | 13.6 | 8.6 – 20.1 |  | 8.487 | <0.001 |
| Clozapine | <6 wk | 1 | 40 | 27.5 | 14.6 – 43.9 |  | 6.105 | <0.001 |
| Clozapine | 6–16 wk | 2 | 91 | 40.0 | 30.1 – 50.4 |  | 11.729 | <0.001 |
| Clozapine | 16–38 wk | 2 | 89 | 47.0 | 36.6 – 57.5 |  | 12.925 | <0.001 |
| Clozapine | >38 wk | 2 | 80 | 76.3 | 66.2 – 85.2 |  | 17.587 | <0.001 |
| FGA | <6 wk | 1 | 45 | 6.7 | 1.4 – 18.3 |  | 2.763 | 0.006 |
| FGA | 6–16 wk | 2 | 134 | 17.6 | 11.3 – 24.8 |  | 8.208 | <0.001 |
| FGA | 16–38 wk | 2 | 163 | 32.3 | 25.3 – 39.8 |  | 13.976 | <0.001 |
| FGA | >38 wk | 3 | 208 | 49.0 | 42.2 – 55.9 |  | 20.550 | <0.001 |
| Haloperidol | <6 wk | 8 | 1166 | 3.6 | 2.3 – 5.2 | 18.35% | 7.970 | <0.001 |
| **Haloperidol** | **6–16 wk** | **4** | **440** | **6.4** | **1.4 – 14.0** | **82.77%** | **3.294** | **0.001** |
| **Haloperidol** | **>38 wk** | **2** | **467** | **15.9** | **12.6 – 19.4** |  | **15.332** | **<0.001** |
| Iloperidone | <6 wk | 1 | 300 | 21.0 | 16.5 – 26.1 |  | 15.545 | <0.001 |
| Iloperidone | 6–16 wk | 2 | 500 | 8.6 | 6.3 – 11.2 |  | 12.021 | <0.001 |
| Iloperidone | 16–38 wk | 1 | 151 | 25.2 | 18.5 – 32.9 |  | 11.983 | <0.001 |
| Lurasidone | <6 wk | 20 | 2628 | 3.6 | 2.5 – 4.9 | 58.02% | 10.207 | <0.001 |
| Lurasidone | >38 wk | 1 | 419 | 7.4 | 5.1 – 10.3 |  | 10.363 | <0.001 |
| **Olanzapine** | **<6 wk** | **21** | **3343** | **16.0** | **12.7 – 19.6** | **80.39%** | **15.114** | **<0.001** |
| **Olanzapine** | **6–16 wk** | **26** | **3377** | **22.0** | **18.4 – 25.8** | **83.77%** | **19.487** | **<0.001** |
| Olanzapine | 16–38 wk | 19 | 3954 | 23.8 | 18.7 – 29.2 | 92.73% | 14.862 | <0.001 |
| **Olanzapine** | **>38 wk** | **13** | **2698** | **34.3** | **29.1 – 39.6** | **87.53%** | **20.925** | **<0.001** |
| Paliperidone | <6 wk | 16 | 1822 | 6.6 | 4.6 – 8.9 | 69.92% | 10.176 | <0.001 |
| Paliperidone | 6–16 wk | 10 | 1094 | 8.4 | 6.8 – 10.4 | 0.00% | 16.368 | <0.001 |
| Paliperidone | 16–38 wk | 6 | 991 | 15.5 | 9.4 – 22.8 | 88.31% | 7.836 | <0.001 |
| Paliperidone | >38 wk | 7 | 2192 | 18.3 | 14.2 – 22.7 | 84.34% | 14.563 | <0.001 |
| Quetiapine | <6 wk | 22 | 2634 | 9.5 | 6.4 – 13.1 | 87.94% | 9.373 | <0.001 |
| **Quetiapine** | **6–16 wk** | **24** | **3980** | **6.3** | **4.6 – 8.3** | **80.94%** | **11.583** | **<0.001** |
| Quetiapine | 16–38 wk | 4 | 688 | 10.1 | 7.6 – 12.8 | 7.68% | 12.739 | <0.001 |
| Quetiapine | >38 wk | 4 | 512 | 17.0 | 9.9 – 25.4 | 71.04% | 7.119 | <0.001 |
| **Risperidone** | **<6 wk** | **8** | **812** | **10.2** | **6.9 – 14.0** | **61.95%** | **9.488** | **<0.001** |
| **Risperidone** | **6–16 wk** | **18** | **1868** | **11.5** | **6.9 – 17.1** | **90.61%** | **7.410** | **<0.001** |
| Risperidone | 16–38 wk | 6 | 421 | 17.4 | 13.1 – 22.1 | 28.14% | 12.583 | <0.001 |
| Risperidone | >38 wk | 12 | 1960 | 23.0 | 17.5 – 28.9 | 88.14% | 13.178 | <0.001 |
| SGA | 6–16 wk | 1 | 109 | 20.2 | 13.1 – 28.9 |  | 8.822 | <0.001 |
| SGA | 16–38 wk | 3 | 506 | 29.5 | 15.0 – 46.4 |  | 6.005 | <0.001 |
| SGA | >38 wk | 2 | 134 | 37.1 | 28.9 – 45.6 |  | 13.477 | <0.001 |
| Sertindole | 6–16 wk | 1 | 205 | 24.9 | 19.1 – 31.4 |  | 14.012 | <0.001 |
| Sertindole | >38 wk | 1 | 131 | 16.8 | 10.8 – 24.3 |  | 8.761 | <0.001 |
| **Ziprasidone** | **<6 wk** | **1** | **150** | **7.3** | **3.7– 12.7** |  | **5.859** | **<0.001** |
| **Ziprasidone** | **6–16 wk** | **3** | **361** | **6.8** | **4.3 – 9.7** |  | **8.448** | **<0.001** |
| Ziprasidone | 16–38 wk | 2 | 219 | 2.8 | 0.8 – 5.7 |  | 3.595 | <0.001 |
| Ziprasidone | >38 wk | 1 | 185 | 7.6 | 4.2 – 12.4 |  | 6.707 | <0.001 |
| Placebo | <6 wk | 59 | 7270 | 2.0 | 1.4 – 2.6 | 62.29% | 11.086 | <0.001 |
| Placebo | 6–16 wk | 37 | 5597 | 2.8 | 1.9 – 3.8 | 74.48% | 9.866 | <0.001 |
| Placebo | 16–38 wk | 10 | 1333 | 7.4 | 2.4 – 14.5 | 94.42% | 4.070 | <0.001 |
| Placebo | >38 wk | 9 | 1057 | 6.7 | 3.2 – 11.2 | 84.79% | 5.541 | <0.001 |
|  |  |  |  |  |  |  |  |  |

Time is in weeks. N= number of studies. n = number of patients included in the study.

Differences with original analyses in **bold.**

## Supplementary table 9.2: Sensitivity meta-analysis CRWG without population >65 years

| **Antipsychotic** | **Time (wk)** | **N studies** | **n** | **% CRWG** | **95% CI** | **I^2^** | **Significance test Z** | ***p*** |
| --- | --- | --- | --- | --- | --- | --- | --- | --- |
| Amisulpride | 6–16 wk | 4 | 339 | 13.3 | 9.8 – 17.2 | 0.00% | 11.719 | <0.001 |
| Amisulpride | 16–38 wk | 1 | 189 | 20.6 | 15.1 – 27.1 |  | 12.035 | <0.001 |
| Aripiprazole | <6 wk | 19 | 2171 | 5.5 | 3.7 – 7.5 | 70.13% | 9.524 | <0.001 |
| **Aripiprazole** | **6–16 wk** | **13** | **1927** | **7.4** | **3.9 – 11.9** | **90.96%** | **6.228** | **<0.001** |
| Aripiprazole | 16–38 wk | 12 | 2257 | 11.7 | 8.7 – 14.9 | 79.37% | 12.725 | <0.001 |
| Aripiprazole | >38 wk | 7 | 1843 | 17.2 | 11.0 – 24.3 | 91.44% | 8.742 | <0.001 |
| **Asenapine** | **<6 wk** | **11** | **1465** | **5.3** | **4.0 – 6.8** | **25.04%** | **12.786** | **<0.001** |
| Asenapine | 16–38 wk | 8 | 982 | 8.0 | 5.6 – 10.7 | 44.93% | 10.182 | <0.001 |
| Asenapine | >38 wk | 2 | 987 | 16.4 | 14.2 – 18.8 |  | 23.833 | <0.001 |
| Blonanserin | <6 wk | 3 | 183 | 1.7 | 0.0 – 5.0 |  | 2.095 | 0.036 |
| Blonanserin | 6–16 wk | 2 | 220 | 9.4 | 5.8– 13.7 |  | 8.037 | <0.001 |
| Blonanserin | 16–38 wk | 1 | 22 | 18.2 | 5.2 – 40.3 |  | 3.345 | 0.001 |
| Brexpiprazole | <6 wk | 10 | 1584 | 7.2 | 5.2 – 9.4 | 60.20% | 11.752 | <0.001 |
| **Brexpiprazole** | **6–16 wk** | **0** |  |  |  |  |  |  |
| Brexpiprazole | >38 wk | 1 | 264 | 5.3 | 2.9– 8.7 |  | 6.679 | <0.001 |
| Cariprazine | <6 wk | 11 | 1650 | 5.3 | 3.4 – 7.5 | 69.72% | 8.835 | <0.001 |
| Cariprazine | 6–16 wk | 5 | 583 | 4.8 | 3.0 – 6.9 | 12.38% | 8.165 | <0.001 |
| Cariprazine | 16–38 wk | 1 | 101 | 26.7 | 18.4 –36.5 |  | 10.001 | <0.001 |
| Chlorpromazine | <6 wk | 2 | 137 | 19.5 | 13.2 – 26.7 |  | 9.320 | <0.001 |
| Chlorpromazine | 6–16 wk | 1 | 154 | 13.6 | 8.6 – 20.1 |  | 8.487 | <0.001 |
| Clozapine | <6 wk | 1 | 40 | 27.5 | 14.6 – 43.9 |  | 6.105 | <0.001 |
| Clozapine | 6–16 wk | 2 | 91 | 40.0 | 30.1 – 50.4 |  | 11.729 | <0.001 |
| Clozapine | 16–38 wk | 2 | 89 | 47.0 | 36.6 – 57.5 |  | 12.925 | <0.001 |
| Clozapine | >38 wk | 2 | 80 | 76.3 | 66.2 – 85.2 |  | 17.587 | <0.001 |
| FGA | <6 wk | 1 | 45 | 6.7 | 1.4 – 18.3 |  | 2.763 | 0.006 |
| FGA | 6–16 wk | 2 | 134 | 17.6 | 11.3 – 24.8 |  | 8.208 | <0.001 |
| FGA | 16–38 wk | 2 | 163 | 32.3 | 25.3 – 39.8 |  | 13.976 | <0.001 |
| FGA | >38 wk | 3 | 208 | 49.0 | 42.2 – 55.9 |  | 20.550 | <0.001 |
| Haloperidol | <6 wk | 8 | 1166 | 3.6 | 2.3 – 5.2 | 18.35% | 7.970 | <0.001 |
| Haloperidol | 6–16 wk | 5 | 572 | 9.1 | 3.2 – 17.3 | 86.83% | 4.231 | <0.001 |
| Haloperidol | >38 wk | 3 | 599 | 22.8 | 7.8 – 42.5 |  | 4.164 | <0.001 |
| Iloperidone | <6 wk | 1 | 300 | 21.0 | 16.5 – 26.1 |  | 15.545 | <0.001 |
| Iloperidone | 6–16 wk | 2 | 500 | 8.6 | 6.3 –11.2 |  | 12.021 | <0.001 |
| Iloperidone | 16–38 wk | 1 | 151 | 25.2 | 18.5 – 32.9 |  | 11.983 | <0.001 |
| Lurasidone | <6 wk | 20 | 2628 | 3.6 | 2.5 – 4.9 | 58.02% | 10.207 | <0.001 |
| Lurasidone | >38 wk | 1 | 419 | 7.4 | 5.1 – 10.3 |  | 10.363 | <0.001 |
| Olanzapine | <6 wk | 23 | 3533 | 17.5 | 13.8 – 21.5 | 85.96% | 14.619 | <0.001 |
| **Olanzapine** | **6–16 wk** | **27** | **3399** | **26.6** | **22.0 – 31.4** | **88.79%** | **18.311** | **<0.001** |
| **Olanzapine** | **16–38 wk** | **18** | **3854** | **24.6** | **19.4 – 30.1** | **92.80%** | **14.875** | **<0.001** |
| Olanzapine | >38 wk | 14 | 2829 | 36.9 | 30.4 – 43.6 | 92.35% | 17.681 | <0.001 |
| Paliperidone | <6 wk | 16 | 1822 | 6.6 | 4.6 – 8.9 | 69.92% | 10.176 | <0.001 |
| Paliperidone | 6–16 wk | 10 | 1094 | 8.4 | 6.8 – 10.4 | 0 | 16.368 | <0.001 |
| Paliperidone | 16–38 wk | 6 | 991 | 15.5 | 9.4 – 22.8 | 88.31% | 7.836 | <0.001 |
| Paliperidone | >38 wk | 7 | 2192 | 18.3 | 14.2 – 22.7 | 84.34% | 14.563 | <0.001 |
| Quetiapine | <6 wk | 22 | 2634 | 9.5 | 6.4 – 13.1 | 87.94% | 9.373 | <0.001 |
| **Quetiapine** | **6–16 wk** | **24** | **3876** | **7.5** | **5.6 – 9.6** | **80.12%** | **12.759** | **<0.001** |
| **Quetiapine** | **16–38 wk** | **3** | **594** | **10.7** | **8.2 – 13.4** |  | **13.140** | **<0.001** |
| Quetiapine | >38 wk | 4 | 512 | 17.0 | 9.9 – 25.4 | 71.04% | 7.119 | <0.001 |
| Risperidone | <6 wk | 10 | 973 | 10.6 | 7.6 – 13.9 | 58.36% | 11.109 | <0.001 |
| **Risperidone** | **6–16 wk** | **17** | **1737** | **14.4** | **9.7 – 19.8** | **87.29%** | **9.404** | **<0.001** |
| **Risperidone** | **16–38 wk** | **5** | **336** | **19.3** | **15.1 – 23.8** | **0.00%** | **14.301** | **<0.001** |
| Risperidone | >38 wk | 12 | 1960 | 23.0 | 17.5 – 28.9 | 88.14% | 13.178 | <0.001 |
| SGA | 6–16 wk | 1 | 109 | 20.2 | 13.1 – 28.9 |  | 8.822 | <0.001 |
| SGA | 16–38 wk | 3 | 506 | 29.5 | 15.0 – 46.4 |  | 6.005 | <0.001 |
| SGA | >38 wk | 2 | 134 | 37.1 | 28.9 – 45.6 |  | 13.477 | <0.001 |
| Sertindole | 6–16 wk | 1 | 205 | 24.9 | 19.1 – 31.4 |  | 14.012 | <0.001 |
| Sertindole | >38 wk | 1 | 131 | 16.8 | 10.8 – 24.3 |  | 8.761 | <0.001 |
| Ziprasidone | <6 wk | 2 | 282 | 2.5 | 0.9 – 4.8 | 75.32% | 4.283 | <0.001 |
| Ziprasidone | 6–16 wk | 5 | 462 | 8.7 | 3.8 – 15.1 |  | 5.065 | <0.001 |
| Ziprasidone | 16–38 wk | 2 | 219 | 2.8 | 0.8 – 5.7 |  | 3.595 | <0.001 |
| Ziprasidone | >38 wk | 1 | 185 | 7.6 | 4.2 – 12.4 |  | 6.707 | <0.001 |
| Placebo | <6 wk | 59 | 7270 | 2.0 | 1.4 – 2.6 | 62.29% | 11.086 | <0.001 |
| **Placebo** | **6–16 wk** | **30** | **4710** | **2.9** | **1.9 – 4.8** | **76.29%** | **8.861** | **<0.001** |
| **Placebo** | **16–38 wk** | **9** | **1191** | **8.0** | **2.4 – 16.2** | **94.86%** | **3.864** | **0.001** |
| Placebo | >38 wk | 9 | 1057 | 6.7 | 3.2 – 11.2 | 84.79% | 5.541 | <0.001 |
|  |  |  |  |  |  |  |  |  |

Time is in weeks. N= number of studies. n = number of patients included in the study.

Differences with original analyses in **bold.**

## Supplementary table 9.3: Sensitivity analysis meta-regression CRWG without AP-naive population and population >65 years

|  |  | Metaregression CRWG duration | | | Sensitivity analysis without AP-naive | | | Sensitivity analysis without 65+ years | | |
| --- | --- | --- | --- | --- | --- | --- | --- | --- | --- | --- |
| **Antipsychotic** | **Period** | **B** | **95% CI** | ***p*** | **B** | **95% CI** | ***p*** | **B** | **95% CI** | ***p*** |
| Amisulpride | 6-16 wk | 0 | 0 | 0 | 0 | 0 | 0 | 0 | 0 | 0 |
|  | 16-38 wk | 0.47 | - 0.29 – 1.23 | 0.145 | 0.47 | - 0.29 – 1.23 | 0.145 | 0.47 | - 0.29 – 1.23 | 0.145 |
|  |  |  |  |  |  |  |  |  |  |  |
| Aripiprazole | <6 wk | 0 | 0 | 0 | 0 | 0 | 0 | 0 | 0 | 0 |
|  | 6-16 wk | 0.09 | - 0.43 – 0.62 | 0.725 | -1.15 | - 0.63 – 0.34 | 0.547 | 0.24 | - 0.32 – 0.81 | 0.393 |
|  | 16-38 wk | 0.69 | 0.12 – 1.26 | **0.019** | 0.70 | 0.20 – 1.20 | **0.007** | 0.69 | 0.12 – 1.27 | **0.020** |
|  | >38 wk | 1.14 | 0.47 –1.80 | **0.001** | 1.13 | 0.57 – 1.69 | **<0.001** | 1.14 | 0.47 – 1.80 | **0.001** |
|  |  |  |  |  |  |  |  |  |  |  |
| Asenapine | <6 wk | 0 | 0 | 0 | 0 | 0 | 0 | 0 | 0 | 0 |
|  | 16-38 wk | 0.50 | - 0.02 – 1.01 | 0.057 | 0.50 | - 0.02 – 1.01 | 0.057 | 0.48 | - 0.05 – 1.00 | 0.072 |
|  | >38 wk | 1.67 | 0.98 – 2.36 | **<0.001** | 1.67 | 0.98 – 2.36 | **<0.001** | 1.65 | 0.95 – 2.35 | **<0.001** |
|  |  |  |  |  |  |  |  |  |  |  |
| Blonanserin | <6 wk | 0 | 0 | 0 |  | insufficient data |  | 0 | 0 | 0 |
|  | 6-16 wk | 1.03 | - 2.65 – 4.71 | 0.353 |  | insufficient data |  | 1.03 | - 2.65 – 4.71 | 0.353 |
|  | 16-38 wk | 1.91 | - 2.73 – 6.54 | 0.219 |  | insufficient data |  | 1.91 | - 2.73 – 6.54 | 0.219 |
|  |  |  |  |  |  |  |  |  |  |  |
| Brexpiprazole | <6 wk | 0 | 0 | 0 | 0 | 0 | 0 | 0 | 0 | 0 |
|  | 6-16 wk | -1.47 | - 2.41 – - 0.53 | **0.006** | -1.47 | - 2.41 – - 0.53 | **0.006** |  | no data |  |
|  | >38 wk | -0.40 | - 1.38 – 0.57 | 0.384 | -0.40 | - 1.38 – 0.57 | 0.384 | -0.39 | - 1.49 – 0.71 | 0.441 |
|  |  |  |  |  |  |  |  |  |  |  |
| Cariprazine | <6 wk | 0 | 0 | 0 | 0 | 0 | 0 | 0 | 0 | 0 |
|  | 6-16 wk | -0.11 | - 0.86 – 0.63 | 0.751 | -0.11 | - 0.86 – 0.63 | 0.751 | -0.11 | - 0.86 – 0.63 | 0.751 |
|  | 16-38 wk | 1.82 | 0.63 – 3.01 | **0.006** | 1.82 | 0.62 – 3.01 | **0.006** | 1.82 | 0.63 – 3.01 | **0.006** |
|  |  |  |  |  |  |  |  |  |  |  |
| Clozapine | <6 wk | 0 | 0 | 0 | 0 | 0 | 0 | 0 | 0 | 0 |
|  | 6-16 wk | 0.33 | - 4.61 – 5.27 | 0.843 | 0.33 | - 4.61 – 5.27 | 0.843 | 0.33 | - 4.61 – 5.27 | 0.843 |
|  | 16-38 wk | 0.92 | - 4.04 – 5.87 | 0.598 | 0.92 | - 4.04 – 5.87 | 0.598 | 0.92 | - 4.04 – 5.87 | 0.598 |
|  | >38 wk | 2.15 | -2.81 – 7.10 | 0.262 | 2.15 | -2.81 – 7.10 | 0.262 | 2.15 | -2.81 – 7.10 | 0.262 |
|  |  |  |  |  |  |  |  |  |  |  |
| FGA | <6 wk | 0 | 0 | 0 | 0 | 0 | 0 | 0 | 0 | 0 |
|  | 6-16 wk | 1.38 | -0.76 – 3.52 | 0.133 | 1.38 | -0.76 – 3.52 | 0.133 | 1.38 | -0.76 – 3.52 | 0.133 |
|  | 16-38 wk | 1.92 | - 016 – 4.00 | 0.061 | 1.92 | - 016 – 4.00 | 0.061 | 1.92 | - 016 – 4.00 | 0.061 |
|  | >38 wk | 2.57 | 0.52 – 4.62 | **0.028** | 2.57 | 0.51 – 4.62 | **0.028** | 2.57 | 0.51 – 4.62 | **0.028** |
|  |  |  |  |  |  |  |  |  |  |  |
| Haloperidol | <6 wk | 0 | 0 | 0 | 0 | 0 | 0 | 0 | 0 | 0 |
|  | 6-16 wk | 1.07 | 0.08 – 2.06 | **0.036** | 0.79 | -0.18 – 1.71 | 0.084 | 1.07 | 0.08 – 2.06 | **0.036** |
|  | >38 wk | 1.88 | 0.82 – 2.95 | **0.002** | 1.36 | 0.33 – 2.39 | **0.015** | 1.88 | 0.82 – 2.95 | **0.002** |
|  |  |  |  |  |  |  |  |  |  |  |
| Lurasidone | <6 wk | 0 | 0 | 0 | 0 | 0 | 0 | 0 | 0 | 0 |
|  | >38 wk | 0.59 | - 0.53 – 1.70 | 0.287 | 0.59 | - 0.53 – 1.70 | 0.287 | 0.59 | - 0.53 – 1.70 | 0.287 |
|  |  |  |  |  |  |  |  |  |  |  |
| Olanzapine | <6 wk | 0 | 0 | 0 | 0 | 0 | 0 | 0 | 0 | 0 |
|  | 6-16 wk | 0.41 | 0.02 – 0.78 | **0.038** | 0.34 | -0.01 – 0.68 | 0.058 | 0.48 | 0.11 – 0.86 | **0.012** |
|  | 16-38 wk | 0.33 | - 0.08 – 0.75 | 0.116 | 0.43 | -0.06 – 0.79 | **0.024** | 0.38 | - 0.03 – 0.79 | 0.069 |
|  | >38 wk | 0.96 | 0.51 – 1.41 | **<0.001** | 0.94 | 0.54 – 1.33 | **<0.001** | 0.96 | 0.53 – 1.39 | **<0.001** |
|  |  |  |  |  |  |  |  |  |  |  |
| Paliperidone | <6 wk | 0 | 0 | 0 | 0 | 0 | 0 | 0 | 0 | 0 |
|  | 6-16 wk | 0.24 | - 0.24 – 0.72 | 0.322 | 0.24 | - 0.24 – 0.72 | 0.322 | 0.24 | - 0.24 – 0.72 | 0.322 |
|  | 16-38 wk | 0.87 | 0.36 – 1.38 | **0.002** | 0.87 | 0.36 – 1.38 | **0.002** | 0.87 | 0.36 – 1.38 | **0.002** |
|  | >38 wk | 1.07 | 0.59 – 1.55 | **<0.001** | 1.07 | 0.59 – 1.55 | **<0.001** | 1.07 | 0.59 – 1.55 | **<0.001** |
|  |  |  |  |  |  |  |  |  |  |  |
| Quetiapine | <6 wk | 0 | 0 | 0 | 0 | 0 | 0 | 0 | 0 | 0 |
|  | 6-16 wk | -0.24 | -0.73 – 0.25 | 0.327 | -0.34 | -0.80 – 0.13 | 0.156 | -0.24 | -0.74 – 0.25 | 0.330 |
|  | 16-38 wk | 0.14 | -0.76 – 1.05 | 0.751 | 0.13 | -0.72 – 0.98 | 0.758 | 0.32 | -0.70 – 1.35 | 0.528 |
|  | >38 wk | 0.78 | -0.10 – 1.66 | 0.082 | 0.76 | -0.07 – 1.60 | 0.070 | 0.78 | -0.11 – 1.67 | 0.083 |
|  |  |  |  |  |  |  |  |  |  |  |
| Risperidone | <6 wk | 0 | 0 | 0 | 0 | 0 | 0 | 0 | 0 | 0 |
|  | 6-16 wk | 0.33 | -0.26 – 0.92 | 0.361 | 0.28 | -0.34 – 0.91 | 0.361 | 0.47 | -0.07 – 1.00 | 0.085 |
|  | 16-38 wk | 0.58 | -0.18 – 1.34 | 0.118 | 0.61 | -0.16 – 1.39 | 0.118 | 0.69 | -0.03 – 1.41 | 0.059 |
|  | >38 wk | 0.90 | 0.29 – 1.51 | **0.005** | 0.93 | 0.30 – 1.57 | **0.005** | 0.89 | 0.35 – 1.43 | **0.002** |
|  |  |  |  |  |  |  |  |  |  |  |
| SGA | 6-16 wk | 0 | 0 | 0 | 0 | 0 | 0 | 0 | 0 | 0 |
|  | 16-38 wk | 0.47 | -1.97 – 2.90 | 0.586 | 0.47 | -1.97 – 2.90 | 0.586 | 0.47 | -1.97 – 2.90 | 0.586 |
|  | >38 wk | 0.99 | -1.65 – 3.62 | 0.319 | 0.99 | -1.65 – 3.62 | 0.319 | 0.99 | -1.65 – 3.62 | 0.319 |
|  |  |  |  |  |  |  |  |  |  |  |
| Ziprasidone | <6 wk | 0 | 0 | 0 | 0 | 0 | 0 | 0 | 0 | 0 |
|  | 6-16 wk | 0.26 | -2.00 – 2.52 | 0.778 | 0.22 | -1.87 – 1.91 | 0.973 | 0.26 | -2.00 – 2.52 | 0.778 |
|  | 16-38 wk | -0.33 | -2.93 – 2.28 | 0.759 | -0.42 | -2.63 – 1.80 | 0.592 | -0.33 | -2.93 – 2.28 | 0.759 |
|  | >38 wk | 0.07 | -2.77 – 2.92 | 0.949 | 0.07 | -2.13 – 2.28 | 0.921 | 0.07 | -2.77 – 2.92 | 0.949 |
|  |  |  |  |  |  |  |  |  |  |  |
| Placebo | <6 wk | 0 | 0 | 0 | 0 | 0 | 0 | 0 | 0 | 0 |
|  | 6-16 wk | 0.09 | - 0.31 – 0.49 | 0.644 | 0.09 | - 0.31 – 0.49 | 0.644 | 0.13 | - 0.29 – 0.55 | 0.542 |
|  | 16-38 wk | 1.14 | 0.54 – 1.74 | **<0.001** | 1.14 | 0.54 – 1.74 | **<0.001** | 1.27 | 0.65 – 1.89 | **<0.001** |
|  | >38 wk | 0.85 | 0.24 – 1.45 | **0.007** | 0.85 | 0.24 – 1.45 | **0.007** | 0.85 | 0.25 – 1.45 | **0.006** |
|  |  |  |  |  |  |  |  |  |  |  |

* Period < 6 weeks was the reference period.

§ Only outcomes that could be analyzed are noted here.

Significant p-values in **bold.**

## Supplementary table 9.4: Sensitivity meta-analysis CRWL without population > 65 years

| **Antipsychotic** | **Time (weeks)** | **N studies** | **n** | **% CRWL** | **95% CI** | **I^2^** | **Significance test Z** | ***p*** |
| --- | --- | --- | --- | --- | --- | --- | --- | --- |
| Aripiprazole | <6 wk | 1 | 122 | 4.9 | 1.8 – 10.4 |  | 4.132 | <0.001 |
| **Aripiprazole** | **6–16 wk** | **8** | **1211** | **7.9** | **5.0 – 11.4** | **75.56%** | **8.383** | **<0.001** |
| Aripiprazole | 16–38 wk | 7 | 1326 | 9.7 | 6.1 – 14.0 | 82.90% | 8.273 | <0.001 |
| Aripiprazole | >38 wk | 2 | 157 | 9.1 | 4.8 – 14.4 |  | 6.153 | <0.001 |
| Asenapine | <6 wk | 4 | 599 | 1.5 | 0.6 – 2.7 | 0.00% | 4.511 | <0.001 |
| Asenapine | 16–38 wk | 3 | 679 | 5.8 | 1.9 – 11.6 |  | 4.100 | <0.001 |
| Asenapine | >38 wk | 1 | 908 | 5.0 | 3.6 – 6.6 |  | 12.602 | <0.001 |
| Lurasidone | <6 wk | 5 | 718 | 1.4 | 0.6 – 2.5 | 0.00% | 4.874 | <0.001 |
| Lurasidone | >38 wk | 1 | 419 | 12.6 | 9.6 – 16.2 |  | 13.950 | <0.001 |
| Olanzapine | <6 wk | 5 | 1985 | 1.2 | 0.2 – 2.6 | 67.84% | 3.236 | 0.001 |
| Olanzapine | 6–16 wk | 2 | 274 | 0.3 | 0.0 – 1.5 |  | 1.061 | 0.289 |
| Olanzapine | 16–38 wk | 6 | 1234 | 4.8 | 3.3 – 6.7 | 47.66% | 9.559 | <0.001 |
| Olanzapine | >38 wk | 4 | 1135 | 10.8 | 4.1 – 20.0 | 94.86% | 4.691 | <0.001 |
| Paliperidone | <6 wk | 8 | 855 | 2.0 | 1.1 – 3.1 | 0.00% | 6.261 | <0.001 |
| Paliperidone | 6–16 wk | 2 | 163 | 1.8 | 0.1 – 4.7 |  | 2.515 | 0.012 |
| Paliperidone | 16–38 wk | 2 | 241 | 4.9 | 2.5 – 8.1 |  | 5.803 | <0.001 |
| Paliperidone | >38 wk | 2 | 995 | 5.7 | 4.4 – 7.3 |  | 13.961 | <0.001 |
| Quetiapine | 16–38 wk | 1 | 175 | 9.1 | 5.3 – 14.4 |  | 7.245 | <0.001 |
| Quetiapine | >38 wk | 1 | 21 | 4.8 | 0.1 – 23.8 |  | 1.420 | 0.156 |
| Risperidone | <6 wk | 1 | 116 | 0.0 | 0.0 – 3.1 |  | 0.000 | 1.000 |
| Risperidone | 6–16 wk | 3 | 380 | 1.4 | 0.0 – 5.9 |  | 1.423 | 0.155 |
| Risperidone | >38 wk | 2 | 449 | 5.8 | 3.8 – 8.2 |  | 9.062 | <0.001 |
| Ziprasidone | 6–16 wk | 3 | 361 | 8.5 | 5.7 – 11.6 |  | 9.614 | <0.001 |
| Ziprasidone | >38 wk | 1 | 28 | 28.6 | 13.2 – 48.7 |  | 5.109 | <0.001 |
| Placebo | <6 wk | 11 | 1311 | 2.3 | 0.7 – 4.5 | 78.79% | 3.898 | <0.001 |
| **Placebo** | **6–16 wk** | **2** | **292** | **5.0** | **2.7 – 7.9** |  | **6.470** | **<0.001** |
| Placebo | 16–38 wk | 4 | 526 | 11.1 | 8.5 – 14.0 |  | 13.549 | <0.001 |
| Placebo | >38 wk | 1 | 133 | 12.0 | 7.0 – 18.8 |  | 7.285 | <0.001 |
|  |  |  |  |  |  |  |  |  |

Time is in weeks. N= number of studies. n = number of patients included in the study.

Differences with original analyses in **bold.**

## Supplementary table 9.5: Sensitivity analysis meta-regression CRWL without population > 65 years

|  |  | Metaregression CRWL | |  | Sensitivity analysis without +65 years | | |
| --- | --- | --- | --- | --- | --- | --- | --- |
| **Antipsychotic** | **Period** | **B** | **95% CI** | ***p*** | **B** | **95% CI** | ***p*** |
| Aripiprazole | <6 wk | 0 | 0 | 0 | 0 | 0 | 0 |
|  | 6–16 wk | 0.64 | -0.74 – 2.01 | 0.345 | 0.05 | -1.07 – 2.07 | 0.504 |
|  | 16–38 wk | 0.80 | -0.61 – 2.20 | 0.248 | 0.76 | -0.82 – 2.33 | 0.321 |
|  | >38 wk | 0.64 | -1.02 – 2.31 | 0.427 | 0.64 | -1.22 – 2.50 | 0.475 |
|  |  |  |  |  |  |  |  |
| Asenapine | <6 wk | 0 | 0 | 0 | 0 | 0 | 0 |
|  | 16–38 wk | 1.29 | -0.08 – 2.67 | 0.061 | 1.29 | -0.08 – 2.67 | 0.061 |
|  | >38 wk | 1.12 | -0.60 – 2.84 | 0.155 | 1.12 | -0.60 – 2.84 | 0.155 |
|  |  |  |  |  |  |  |  |
| Lurasidone | <6 wk | 0 | 0 | 0 | 0 | 0 | 0 |
|  | >38 wk | 2.11 | 1.17 – 3.04 | **0.003** | 2.11 | 1.17 – 3.04 | **0.003** |
|  |  |  |  |  |  |  |  |
| Olanzapine | <6 wk | 0 | 0 | 0 | 0 | 0 | 0 |
|  | 6–16 wk | 0.37 | -1.76 – 2.50 | 0.709 | 0.37 | -1.76 – 2.50 | 0.709 |
|  | 16–38 wk | 1.05 | -0.00 – 2.10 | **0.050** | 1.05 | -0.00 – 2.10 | **0.050** |
|  | >38 wk | 1.82 | 0.73 – 2.92 | **0.004** | 1.82 | 0.73 – 2.92 | **0.004** |
|  |  |  |  |  |  |  |  |
| Paliperidone | <6 wk | 0 | 0 | 0 | 0 | 0 | 0 |
|  | 6–16 wk | -0.18 | -1.64 – 1.28 | 0.786 | -0.18 | -1.64 – 1.28 | 0.786 |
|  | 16–38 wk | 0.67 | -0.28 – 1.63 | 0.145 | 0.67 | -0.28 – 1.63 | 0.145 |
|  | >38 wk | 0.83 | 0.09 – 1.57 | **0.032** | 0.83 | 0.09 – 1.57 | **0.032** |
|  |  |  |  |  |  |  |  |
| Placebo | <6 wk | 0 | 0 | 0 | 0 | 0 | 0 |
|  | 6–16 wk | 0.30 | -0.50 – 1.11 | 0.435 | 0.17 | -0.90 – 1.25 | 0.730 |
|  | 16–8 wk | 1.14 | 0.29 – 1.98 | **0.011** | 1.08 | 0.31 – 1.86 | **0.010** |
|  | >38 wk | 1.17 | -0.21 – 2.55 | 0.092 | 1.12 | -0.13 – 2.37 | 0.076 |
|  |  |  |  |  |  |  |  |

* Period < 6 weeks was the reference period.

§ Only outcomes that could be analyzed are noted here.

Significant p-values in **bold.**

## Supplementary table 9.6: Sensitivity meta-analysis CRWG without studies with high Risk of Bias

| **Antipsychotic** | **Time (wk)** | **N studies** | **n** | **% CRWG** | **95% CI** | **I^2^** | **Significance test Z** | ***p*** |
| --- | --- | --- | --- | --- | --- | --- | --- | --- |
| Amisulpride | 6–16 wk | 4 | 339 | 13.3 | 9.8 – 17.2 | 0.00% | 11.719 | <0.001 |
| Amisulpride | 16–38 wk | 1 | 189 | 20.6 | 15.1 – 27.1 |  | 12.035 | <0.001 |
| **Aripiprazole** | **<6 wk** | **18** | **2141** | **5.5** | **3.7 – 7.6** | **71.87%** | **9.338** | **<0.001** |
| Aripiprazole | 6–16 wk | 19 | 2683 | 6.7 | 4.1 – 10.0 | 89.07% | 7.657 | <0.001 |
| Aripiprazole | 16–38 wk | 12 | 2257 | 11.7 | 8.7 – 14.9 | 79.37% | 12.725 | <0.001 |
| **Aripiprazole** | **>38 wk** | **6** | **1762** | **16.3** | **9.7 – 24.1** | **92.78%** | **7.670** | **<0.001** |
| Asenapine | <6 wk | 13 | 1586 | 4.7 | 3.3 – 6.3 | 43.01% | 10.607 | <0.001 |
| Asenapine | 16–38 wk | 8 | 982 | 8.0 | 5.6 – 10.7 | 44.93% | 10.182 | <0.001 |
| Asenapine | >38 wk | 2 | 987 | 16.4 | 14.2 – 18.8 |  | 23.833 | <0.001 |
| Blonanserin | <6 wk | 3 | 183 | 1.7 | 0.0 – 5.0 |  | 2.095 | 0.036 |
| Blonanserin | 6–16 wk | 2 | 220 | 9.4 | 5.8– 13.7 |  | 8.037 | <0.001 |
| Blonanserin | 16–38 wk | 1 | 22 | 18.2 | 5.2 – 40.3 |  | 3.345 | 0.001 |
| Brexpiprazole | <6 wk | 10 | 1584 | 7.2 | 5.2 – 9.4 | 60.20% | 11.752 | <0.001 |
| Brexpiprazole | 6–16 wk | 3 | 430 | 1.9 | 0.7 – 3.4 |  | 4.412 | <0.001 |
| Brexpiprazole | >38 wk | 1 | 264 | 5.3 | 2.9– 8.7 |  | 6.679 | <0.001 |
| Cariprazine | <6 wk | 11 | 1650 | 5.3 | 3.4 – 7.5 | 69.72% | 8.835 | <0.001 |
| Cariprazine | 6–16 wk | 5 | 583 | 4.8 | 3.0 – 6.9 | 12.38% | 8.165 | <0.001 |
| Cariprazine | 16–38 wk | 1 | 101 | 26.7 | 18.4 –36.5 |  | 10.001 | <0.001 |
| Chlorpromazine | <6 wk | 2 | 137 | 19.5 | 13.2 – 26.7 |  | 9.320 | <0.001 |
| Chlorpromazine | 6–16 wk | 1 | 154 | 13.6 | 8.6 – 20.1 |  | 8.487 | <0.001 |
| Clozapine | <6 wk | 1 | 40 | 27.5 | 14.6 – 43.9 |  | 6.105 | <0.001 |
| Clozapine | 6–16 wk | 2 | 91 | 40.0 | 30.1 – 50.4 |  | 11.729 | <0.001 |
| Clozapine | 16–38 wk | 2 | 89 | 47.0 | 36.6 – 57.5 |  | 12.925 | <0.001 |
| Clozapine | >38 wk | 2 | 80 | 76.3 | 66.2 – 85.2 |  | 17.587 | <0.001 |
| FGA | <6 wk | 1 | 45 | 6.7 | 1.4 – 18.3 |  | 2.763 | 0.006 |
| FGA | 6–16 wk | 2 | 134 | 17.6 | 11.3 – 24.8 |  | 8.208 | <0.001 |
| FGA | 16–38 wk | 2 | 163 | 32.3 | 25.3 – 39.8 |  | 13.976 | <0.001 |
| FGA | >38 wk | 3 | 208 | 49.0 | 42.2 – 55.9 |  | 20.550 | <0.001 |
| Haloperidol | <6 wk | 8 | 1166 | 3.6 | 2.3 – 5.2 | 18.35% | 7.970 | <0.001 |
| Haloperidol | 6–16 wk | 5 | 572 | 9.1 | 3.2 – 17.3 | 86.83% | 4.231 | <0.001 |
| **Haloperidol** | **>38 wk** | **2** | **565** | **21.8** | **18.4 – 25.3** |  | **21.497** | **<0.001** |
| **Iloperidone** | **<6 wk** | **0** |  |  |  |  |  |  |
| Iloperidone | 6–16 wk | 2 | 500 | 8.6 | 6.3 –11.2 |  | 12.021 | <0.001 |
| Iloperidone | 16–38 wk | 1 | 151 | 25.2 | 18.5 – 32.9 |  | 11.983 | <0.001 |
| Lurasidone | <6 wk | 20 | 2628 | 3.6 | 2.5 – 4.9 | 58.02% | 10.207 | <0.001 |
| Lurasidone | >38 wk | 1 | 419 | 7.4 | 5.1 – 10.3 |  | 10.363 | <0.001 |
| **Olanzapine** | **<6 wk** | **22** | **3608** | **17.3** | **13.5 – 21.4** | **86.52%** | **14.244** | **<0.001** |
| Olanzapine | 6–16 wk | 29 | 3691 | 25.2 | 20.6 – 30.1 | 90.51% | 17.116 | <0.001 |
| Olanzapine | 16–38 wk | 19 | 3954 | 23.8 | 18.7 – 29.2 | 92.73% | 14.862 | <0.001 |
| **Olanzapine** | **>38 wk** | **13** | **2781** | **36.9** | **30.1 – 43.8** | **92.93%** | **17.026** | **<0.001** |
| Paliperidone | <6 wk | 16 | 1822 | 6.6 | 4.6 – 8.9 | 69.92% | 10.176 | <0.001 |
| Paliperidone | 6–16 wk | 10 | 1094 | 8.4 | 6.8 – 10.4 | 0.00% | 16.368 | <0.001 |
| Paliperidone | 16–38 wk | 6 | 991 | 15.5 | 9.4 – 22.8 | 88.31% | 7.836 | <0.001 |
| **Paliperidone** | **>38 wk** | **5** | **1764** | **17.6** | **12.5 – 23.4** | **88.65%** | **11.171** | **<0.001** |
| Quetiapine | <6 wk | 22 | 2634 | 9.5 | 6.4 – 13.1 | 87.94% | 9.373 | <0.001 |
| Quetiapine | 6–16 wk | 25 | 4042 | 7.0 | 5.0 – 9.2 | 84.27% | 11.177 | <0.001 |
| Quetiapine | 16–38 wk | 4 | 688 | 10.1 | 7.6 – 12.8 | 7.68% | 12.739 | <0.001 |
| **Quetiapine** | **>38 wk** | **2** | **426** | **14.6** | **11.4 – 18.2** |  | **14.626** | **<0.001** |
| **Risperidone** | **<6 wk** | **9** | **717** | **10.2** | **7.0 – 14.0** | **62.30%** | **9.698** | **<0.001** |
| Risperidone | 6–16 wk | 19 | 2020 | 12.4 | 7.6 – 18.1 | 91.42% | 7.746 | <0.001 |
| Risperidone | 16–38 wk | 6 | 421 | 17.4 | 13.1 – 22.1 | 28.14% | 12.583 | <0.001 |
| **Risperidone** | **>38 wk** | **11** | **1904** | **22.7** | **17.0 – 28.9** | **89.01%** | **12.492** | **<0.001** |
| SGA | 6–16 wk | 1 | 109 | 20.2 | 13.1 – 28.9 |  | 8.822 | <0.001 |
| SGA | 16–38 wk | 3 | 506 | 29.5 | 15.0 – 46.4 |  | 6.005 | <0.001 |
| SGA | >38 wk | 2 | 134 | 37.1 | 28.9 – 45.6 |  | 13.477 | <0.001 |
| Sertindole | 6–16 wk | 1 | 205 | 24.9 | 19.1 – 31.4 |  | 14.012 | <0.001 |
| Sertindole | >38 wk | 1 | 131 | 16.8 | 10.8 – 24.3 |  | 8.761 | <0.001 |
| **Ziprasidone** | **<6 wk** | **1** | **132** | **0.0** | **0.0 – 2.8** |  | **0.000** | **1.000** |
| Ziprasidone | 6–16 wk | 5 | 462 | 8.7 | 3.8 – 15.1 |  | 5.065 | <0.001 |
| Ziprasidone | 16–38 wk | 2 | 219 | 2.8 | 0.8 – 5.7 |  | 3.595 | <0.001 |
| Ziprasidone | >38 wk | 1 | 185 | 7.6 | 4.2 – 12.4 |  | 6.707 | <0.001 |
| **Placebo** | **<6 wk** | **57** | **7009** | **2.0** | **1.5 – 2.7** | **61.07%** | **10.856** | **<0.001** |
| Placebo | 6–16 wk | 37 | 5597 | 2.8 | 1.9 – 3.8 | 74.48% | 9.866 | <0.001 |
| Placebo | 16–38 wk | 10 | 1333 | 7.4 | 2.4 – 14.5 | 94.42% | 4.070 | <0.001 |
| Placebo | >38 wk | 9 | 1057 | 6.7 | 3.2 – 11.2 | 84.79% | 5.541 | <0.001 |
|  |  |  |  |  |  |  |  |  |

Time is in weeks. N= number of studies. n = number of patients included in the study.

Differences with original analyses in **bold.**

## Supplementary table 9.7: Sensitivity analysis meta-regression CRWG without studies with high Risk of Bias

|  |  | Metaregression CRWG duration | | | Sensitivity analysis without high RoB | | |
| --- | --- | --- | --- | --- | --- | --- | --- |
| **Antipsychotic** | **Period** | **B** | **95% CI** | ***p*** | **B** | **95% CI** | ***p*** |
| Amisulpride | 6–16 wk | 0 | 0 | 0 | 0 | 0 | 0 |
|  | 16–38 wk | 0.47 | -0.29 – 1.23 | 0.145 | 0.47 | -0.29 – 1.23 | 0.145 |
|  |  |  |  |  |  |  |  |
| Aripiprazole | <6 wk | 0 | 0 | 0 | 0 | 0 | 0 |
|  | 6–16 wk | 0.09 | -0.43 – 0.62 | 0.725 | 0.10 | -0.44 – 0.65 | 0.704 |
|  | 16–38 wk | 0.69 | 0.12 – 1.26 | **0.019** | 0.70 | 0.12 – 1.29 | **0.020** |
|  | >38 wk | 1.14 | -0.47 – 1.80 | **0.001** | 1.08 | 0.37 – 1.79 | **0.004** |
|  |  |  |  |  |  |  |  |
| Asenapine | <6 wk | 0 | 0 | 0 | 0 | 0 | 0 |
|  | 16–38 wk | 0.50 | -0.02 – 1.01 | 0.057 | 0.50 | -0.02 – 1.01 | 0.057 |
|  | >38 wk | 1.67 | 0.98 – 2.36 | **<0.001** | 1.67 | 0.98 – 2.36 | **<0.001** |
|  |  |  |  |  |  |  |  |
| Blonanserin | <6 wk | 0 | 0 | 0 | 0 | 0 | 0 |
|  | 6–16 wk | 1.03 | -2.65 – 4.71 | 0.353 | 1.03 | -2.65 – 4.71 | 0.353 |
|  | 16–38 wk | 1.91 | -2.73 – 6.54 | 0.219 | 1.91 | -2.73 – 6.54 | 0.219 |
|  |  |  |  |  |  |  |  |
| Brexpiprazole | <6 wk | 0 | 0 | 0 | 0 | 0 | 0 |
|  | 6–16 wk | -1.47 | -2.41 – -0.53 | **0.006** | -1.47 | -2.41 – -0.53 | **0.006** |
|  | >38 wk | -0.40 | -1.38 – 0.57 | 0.384 | -0.40 | -1.38 – 0.57 | 0.384 |
|  |  |  |  |  |  |  |  |
| Cariprazine | <6 wk | 0 | 0 | 0 | 0 | 0 | 0 |
|  | 6–16 wk | -0.11 | -0.86 – 0.63 | 0.751 | -0.11 | -0.86 – 0.63 | 0.751 |
|  | 16–38 wk | 1.82 | 0.63 – 3.01 | **0.006** | 1.82 | 0.63 – 3.01 | **0.006** |
|  |  |  |  |  |  |  |  |
| Clozapine | <6 wk | 0 | 0 | 0 | 0 | 0 | 0 |
|  | 6–16 wk | 0.33 | -4.61 – 5.27 | 0.843 | 0.33 | -4.61 – 5.27 | 0.843 |
|  | 16–38 wk | 0.92 | -4.04 – 5.87 | 0.598 | 0.92 | -4.04 – 5.87 | 0.598 |
|  | >38 wk | 2.15 | -2.81 – 7.10 | 0.262 | 2.15 | -2.81 – 7.10 | 0.262 |
|  |  |  |  |  |  |  |  |
| FGA | <6 wk | 0 | 0 | 0 | 0 | 0 | 0 |
|  | 6–16 wk | 1.38 | -0.76 – 3.52 | 0.133 | 1.38 | -0.76 – 3.52 | 0.133 |
|  | 16–38 wk | 1.92 | -016 – 4.00 | 0.061 | 1.92 | -016 – 4.00 | 0.061 |
|  | >38 wk | 2.57 | 0.52 – 4.62 | **0.028** | 2.57 | 0.52 – 4.62 | **0.028** |
|  |  |  |  |  |  |  |  |
| Haloperidol | <6 wk | 0 | 0 | 0 | 0 | 0 | 0 |
|  | 6–16 wk | 1.07 | 0.08 – 2.06 | **0.036** | 1.07 | 0.08 – 2.07 | **0.036** |
|  | >38 wk | 1.88 | 0.82 – 2.95 | **0.002** | 2.12 | 0.96 – 3.29 | **0.002** |
|  |  |  |  |  |  |  |  |
| Lurasidone | <6 wk | 0 | 0 | 0 | 0 | 0 | 0 |
|  | >38 wk | 0.59 | -0.53 – 1.70 | 0.287 | 0.59 | -0.53 – 1.70 | 0.287 |
|  |  |  |  |  |  |  |  |
| Olanzapine | <6 wk | 0 | 0 | 0 | 0 | 0 | 0 |
|  | 6–16 wk | 0.41 | 0.02 – 0.78 | **0.038** | 0.42 | 0.03 – 0.81 | **0.037** |
|  | 16–38 wk | 0.33 | -0.08 – 0.75 | 0.116 | 0.35 | -0.08 – 0.77 | 0.112 |
|  | >38 wk | 0.96 | 0.51 – 1.41 | **<0.001** | 0.97 | 0.51 – 1.43 | **<0.001** |
|  |  |  |  |  |  |  |  |
| Paliperidone | <6 wk | 0 | 0 | 0 | 0 | 0 | 0 |
|  | 6–16 wk | 0.24 | -0.24 – 0.72 | 0.322 | 0.24 | -0.25 – 0.74 | 0.325 |
|  | 16–38 wk | 0.87 | 0.36 – 1.38 | **0.002** | 0.87 | 0.34 – 1.41 | **0.002** |
|  | >38 wk | 1.07 | 0.59 – 1.55 | **<0.001** | 1.03 | 0.49 – 1.58 | **<0.001** |
|  |  |  |  |  |  |  |  |
| Quetiapine | <6 wk | 0 | 0 | 0 | 0 | 0 | 0 |
|  | 6–16 wk | -0.24 | -0.73 – 0.25 | 0.327 | -0.24 | -0.73 – 0.25 | 0.324 |
|  | 16–38 wk | 0.14 | -0.76 – 1.05 | 0.751 | 0.14 | -0.76 – 1.04 | 0.752 |
|  | >38 wk | 0.78 | -0.10 – 1.66 | 0.082 | 0.38 | -0.77 – 1.53 | 0.509 |
|  |  |  |  |  |  |  |  |
| Risperidone | <6 wk | 0 | 0 | 0 | 0 | 0 | 0 |
|  | 6–16 wk | 0.33 | -0.26 – 0.92 | 0.361 | 0.36 | -0.28 – 0.99 | 0.261 |
|  | 16–38 wk | 0.58 | -0.18 – 1.34 | 0.118 | 0.62 | -0.18 – 1.42 | 0.128 |
|  | >38 wk | 0.90 | 0.29 – 1.51 | **0.005** | 0.92 | 0.26 – 1.58 | **0.008** |
|  |  |  |  |  |  |  |  |
| SGA | 6–16 wk | 0 | 0 | 0 | 0 | 0 | 0 |
|  | 16–38 wk | 0.47 | -1.97 – 2.90 | 0.586 | 0.47 | -1.97 – 2.90 | 0.586 |
|  | >38 wk | 0.99 | -1.65 – 3.62 | 0.319 | 0.99 | -1.65 – 3.62 | 0.319 |
|  |  |  |  |  |  |  |  |
| Ziprasidone | <6 wk | 0 | 0 | 0 | 0 | 0 | 0 |
|  | 6–16 wk | 0.26 | -2.00 – 2.52 | 0.778 | 0.19 | -2.03 – 2.41 | 0.837 |
|  | 16–38 wk | -0.33 | -2.93 – 2.28 | 0.759 | -0.40 | -2.98 – 2.17 | 0.704 |
|  | >38 wk | 0.07 | -2.77 – 2.92 | 0.949 |  | no data |  |
|  |  |  |  |  |  |  |  |
| Placebo | <6 wk | 0 | 0 | 0 | 0 | 0 | 0 |
|  | 6–16 wk | 0.09 | -0.31 – 0.49 | 0.644 | 0.10 | -0.31 – 0.51 | 0.628 |
|  | 16–38 wk | 1.14 | 0.54 –1.74 | **<0.001** | 1.14 | 0.54 –1.76 | **<0.001** |
|  | >38 wk | 0.85 | 0.24 – 1.45 | **0.007** | 0.86 | 0.24 – 1.47 | **0.007** |
|  |  |  |  |  |  |  |  |

* Period < 6 weeks was the reference period.

§ Only outcomes that could be analyzed are noted here.

Significant p-values in **bold.**

## Supplementary table 9.8: Sensitivity meta-analysis CRWL without studies with high Risk of Bias

| **Antipsychotic** | **Time (weeks)** | **N studies** | **n** | **% CRWL** | **95% CI** | **I^2^** | **Significance test Z** | ***p*** |
| --- | --- | --- | --- | --- | --- | --- | --- | --- |
| Aripiprazole | <6 wk | 1 | 122 | 4.9 | 1.8 –10.4 |  | 4.132 | <0.001 |
| Aripiprazole | 6–16 wk | 12 | 1708 | 8.8 | 6.5 – 11.5 | 68.52% | 12.097 | <0.001 |
| Aripiprazole | 16–38 wk | 7 | 1326 | 9.7 | 6.1– 14.0 | 82.90% | 8.273 | <0.001 |
| Aripiprazole | >38 wk | 2 | 157 | 9.1 | 4.8 – 14.4 |  | 6.153 | <0.001 |
| Asenapine | <6 wk | 4 | 599 | 1.5 | 0.6 – 2.7 | 0.00% | 4.511 | <0.001 |
| Asenapine | 16–38 wk | 3 | 679 | 5.8 | 1.9 – 11.6 |  | 4.100 | <0.001 |
| Asenapine | >38 wk | 1 | 908 | 5.0 | 3.6 – 6.6 |  | 12.602 | <0.001 |
| Lurasidone | <6 wk | 5 | 718 | 1.4 | 0.6 – 2.5 |  | 4.874 | <0.001 |
| Lurasidone | >38 wk | 1 | 419 | 12.6 | 9.6 – 16.2 |  | 13.950 | <0.001 |
| Olanzapine | <6 wk | 5 | 1985 | 1.2 | 0.2 – 2.6 | 67.84% | 3.236 | 0.001 |
| Olanzapine | 6–16 wk | 2 | 274 | 0.3 | 0.0 – 1.5 |  | 1.061 | 0.289 |
| Olanzapine | 16–38 wk | 6 | 1234 | 4.8 | 3.3 – 6.7 | 47.66% | 9.559 | <0.001 |
| Olanzapine | >38 wk | 4 | 1135 | 10.8 | 4.1 – 20.0 | 94.86% | 4.691 | <0.001 |
| Paliperidone | <6 wk | 8 | 855 | 2.0 | 1.1 – 3.1 | 0.00% | 6.261 | <0.001 |
| Paliperidone | 6–16 wk | 2 | 163 | 1.8 | 0.1 – 4.7 |  | 2.515 | 0.012 |
| Paliperidone | 16–38 wk | 2 | 241 | 4.9 | 2.5 – 8.1 |  | 5.803 | <0.001 |
| Paliperidone | >38 wk | 2 | 995 | 5.7 | 4.4 – 7.3 |  | 13.961 | <0.001 |
| Quetiapine | 16–38 wk | 1 | 175 | 9.1 | 5.3 – 14.4 |  | 7.245 | <0.001 |
| **Quetiapine** | **>38 wk** | **0** |  |  |  |  |  |  |
| **Risperidone** | **<6 wk** | **0** |  |  |  |  |  |  |
| Risperidone | 6–16 wk | 3 | 380 | 1.4 | 0.0 – 5.9 |  | 1.423 | 0.155 |
| Risperidone | >38 wk | 2 | 449 | 5.8 | 3.8 – 8.2 |  | 9.062 | <0.001 |
| Ziprasidone | 6–16 wk | 3 | 361 | 8.5 | 5.7 – 11.6 |  | 9.614 | <0.001 |
| Ziprasidone | >38 wk | 1 | 28 | 28.6 | 13.2 – 48.7 |  | 5.109 | <0.001 |
| **Placebo** | **<6 wk** | **10** | **1197** | **2.3** | **0.6 – 4.8** | **80.86%** | **3.509** | **<0.001** |
| Placebo | 6–16 wk | 6 | 811 | 5.3 | 2.8 – 8.6 | 69.34% | 6.119 | <0.001 |
| Placebo | 16–38 wk | 4 | 526 | 11.1 | 8.5 – 14.0 |  | 13.549 | <0.001 |
| Placebo | >38 wk | 1 | 133 | 12.0 | 7.0 – 18.8 |  | 7.285 | <0.001 |
|  |  |  |  |  |  |  |  |  |

Time is in weeks. N= number of studies. n = number of patients included in the study.

Differences with original analyses in **bold.**

## Supplementary table 9.9: Sensitivity analysis meta-regression CRWL without studies with high Risk of Bias

|  |  | Metaregression CRWL | |  | Sensitivity analysis without high RoB | | |
| --- | --- | --- | --- | --- | --- | --- | --- |
| **Antipsychotic** | **Period** | **B** | **95% CI** | ***p*** | **B** | **95% CI** | ***p*** |
| Aripiprazole | <6 wk | 0 | 0 | 0 | 0 | 0 | 0 |
|  | 6–16 wk | 0.64 | -0.74 – 2.01 | 0.345 | 0.64 | -0.74 – 2.01 | 0.345 |
|  | 16–38 wk | 0.80 | -0.61 – 2.20 | 0.248 | 0.80 | -0.61 – 2.20 | 0.248 |
|  | >38 wk | 0.64 | -1.02 – 2.31 | 0.427 | 0.64 | -1.02 – 2.31 | 0.427 |
|  |  |  |  |  |  |  |  |
| Asenapine | <6 wk | 0 | 0 | 0 | 0 | 0 | 0 |
|  | 16–38 wk | 1.29 | -0.08 –2.67 | 0.061 | 1.29 | -0.08 – 2.67 | 0.061 |
|  | >38 wk | 1.12 | -0.60 – 2.84 | 0.155 | 1.12 | -0.60 – 2.84 | 0.155 |
|  |  |  |  |  |  |  |  |
| Lurasidone | <6 wk | 0 | 0 | 0 | 0 | 0 | 0 |
|  | >38 wk | 2.11 | 1.17 – 3.04 | **0.003** | 2.11 | 1.17 – 3.04 | **0.003** |
|  |  |  |  |  |  |  |  |
| Olanzapine | <6 wk | 0 | 0 | 0 | 0 | 0 | 0 |
|  | 6–16 wk | 0.37 | -1.76 – 2.50 | 0.709 | 0.37 | -1.76 – 2.50 | 0.709 |
|  | 16–38 wk | 1.05 | -0.00 – 2.10 | **0.05** | 1.05 | -0.00 – 2.10 | **0.05** |
|  | >38 wk | 1.82 | 0.73 –2.92 | **0.004** | 1.82 | 0.73 – 2.92 | **0.004** |
|  |  |  |  |  |  |  |  |
| Paliperidone | <6 wk | 0 | 0 | 0 | 0 | 0 | 0 |
|  | 6–16 wk | -0.18 | -1.64 – 1.28 | 0.786 | -0.18 | -1.64 – 1.28 | 0.786 |
|  | 16–38 wk | 0.67 | -0.28 – 1.63 | 0.145 | 0.67 | -0.28 – 1.63 | 0.145 |
|  | >38 wk | 0.83 | 0.09 – 1.57 | **0.032** | 0.83 | 0.09 – 1.57 | **0.032** |
|  |  |  |  |  |  |  |  |
| Placebo | <6 wk | 0 | 0 | 0 | 0 | 0 | 0 |
|  | 6–16 wk | 0.30 | -0.50 – 1.11 | 0.435 | 0.27 | -0.57 – 1.11 | 0.512 |
|  | 16–38 wk | 1.14 | 0.29 – 1.98 | **0.011** | 1.10 | 0.22 – 1.99 | **0.018** |
|  | >38 wk | 1.17 | -0.21 – 2.55 | 0.092 | 1.13 | -0.29 –2.56 | 0.110 |
|  |  |  |  |  |  |  |  |

* Period < 6 weeks was the reference period.

§ Only outcomes that could be analyzed are noted here.

Significant p-values in **bold.**

# Appendix 10: Publication Bias

## Supplementary file 10.1: Funnel plots CRWG stratified by AP and study duration

## Supplementary file10.2: Funnel plots CRWL stratified by AP and study duration

## Supplementary file 10.3: CRWG Egger tests coefficient and p-value and added studies by trim-and-fill procedure

|  | Duration | Egger test CRWG | Trim & fill^1^ | pooled estimate (95% CI)^2^ | pooled estimate t&f (95% CI)^3^ |
| --- | --- | --- | --- | --- | --- |
| Amisulpride | <6 weeks |  |  |  |  |
|  | 6-16 weeks | -1.32 p=0.364 | 0 |  |  |
|  | 16-38 weeks |  | 0 |  |  |
|  | >38 weeks |  |  |  |  |
| Aripiprazole | <6 weeks | -2.90 p<0.001 | 0 |  |  |
|  | 6-16 weeks | -6.27 p<0.001 | 0 |  |  |
|  | 16-38 weeks | -4.08 p=0.004 | 0 |  |  |
|  | >38 weeks | -3.74 p=0.070 | 0 |  |  |
| Asenapine | <6 weeks | -2.43 p<0.001 | 0 |  |  |
|  | 6-16 weeks |  |  |  |  |
|  | 16-38 weeks | 0.61 p=0.718 | 0 |  |  |
|  | >38 weeks |  | 1 | 0.164  (0.142 - 0.188) | 0.176  (0.056 - 0.558) |
| Blonanserin | <6 weeks |  | 0 |  |  |
|  | 6-16 weeks |  | 0 |  |  |
|  | 16-38 weeks |  | 0 |  |  |
|  | >38 weeks |  |  |  |  |
| Brexpiprazole | <6 weeks | -4.85 p<0.001 | 0 |  |  |
|  | 6-16 weeks | -2.27 p=0.238 | 0 |  |  |
|  | 16-38 weeks |  |  |  |  |
|  | >38 weeks |  | 0 |  |  |
| Cariprazine | <6 weeks | -5.72 p<0.001 | 0 |  |  |
|  | 6-16 weeks | -3.02 p=0.177 | 0 |  |  |
|  | 16-38 weeks |  | 0 |  |  |
|  | >38 weeks |  |  |  |  |
| Chlorpromazine | <6 weeks |  | 1 | 0.195  (0.132 - 0.267) | 0.220  (0.152 - 0.317) |
|  | 6-16 weeks |  | 0 |  |  |
|  | 16-38 weeks |  |  |  |  |
|  | >38 weeks |  |  |  |  |
| Clozapine | <6 weeks |  | 0 |  |  |
|  | 6-16 weeks |  | 0 |  |  |
|  | 16-38 weeks |  | 1 | 0.470  (0.366 - 0.575) | - |
|  | >38 weeks |  | 1 | 0.763  (0.662 - 0.852) | - |
| FGA | <6 weeks |  | 0 |  |  |
|  | 6-16 weeks |  | 0 |  |  |
|  | 16-38 weeks |  | 0 |  |  |
|  | >38 weeks | -1.60 p=0.542 | 0 |  |  |
| Haloperidol | <6 weeks | 0.04 p=0.971 | 0 |  |  |
|  | 6-16 weeks | -5.15 p=0.114 | 0 |  |  |
|  | 16-38 weeks |  |  |  |  |
|  | >38 weeks | 0.29 p=0.978 | 0 |  |  |
| Iloperidone | <6 weeks |  | 0 |  |  |
|  | 6-16 weeks |  | 0 |  |  |
|  | 16-38 weeks |  |  |  |  |
|  | >38 weeks |  |  |  |  |
| Lurasidone | <6 weeks | -3.79 p<0.001 | 0 |  |  |
|  | 6-16 weeks |  |  |  |  |
|  | 16-38 weeks |  |  |  |  |
|  | >38 weeks |  | 0 |  |  |
| Olanzapine | <6 weeks | -0.10 p=0.921 | 0 |  |  |
|  | 6-16 weeks | -1.98 p=0.240 | 0 |  |  |
|  | 16-38 weeks | -3.08 p=0.171 | 0 |  |  |
|  | >38 weeks | -0.11 p=0.974 | 0 |  |  |
| Paliperidone | <6 weeks | -4.69 p<0.001 | 0 |  |  |
|  | 6-16 weeks | -1.34 p=0.204 | 0 |  |  |
|  | 16-38 weeks | -9.33 p=0.077 | 0 |  |  |
|  | >38 weeks | -0.57 p=0.898 | 0 |  |  |
| Quetiapine | <6 weeks | -6.28 p<0.001 | 0 |  |  |
|  | 6-16 weeks | -3.47 p=0.003 | 0 |  |  |
|  | 16-38 weeks | -0.30 p=0.849 | 0 |  |  |
|  | >38 weeks | 1.23 p=0.690 | 0 |  |  |
| Risperidone | <6 weeks | -2.99 p=0.006 | 0 |  |  |
|  | 6-16 weeks | -3.63 p=0.010 | 0 |  |  |
|  | 16-38 weeks | -1.84 p=0.395 | 0 |  |  |
|  | >38 weeks | 2.51 p=0.466 | 1 | 0.230  (0.175 - 0.289) | 0.281  (0.201 - 0.392) |
| Sertindole | <6 weeks |  |  |  |  |
|  | 6-16 weeks |  | 0 |  |  |
|  | 16-38 weeks |  |  |  |  |
|  | >38 weeks |  | 0 |  |  |
| SGA | <6 weeks |  |  |  |  |
|  | 6-16 weeks |  | 0 |  |  |
|  | 16-38 weeks | 7.72 p=0.766 | 0 |  |  |
|  | >38 weeks |  | 1 | 0.371  (0.289 - 0.456) | 0.520  (0.283 - 0.954) |
| Ziprasidone | <6 weeks |  | 0 |  |  |
|  | 6-16 weeks | -2.94 p=0.405 | 0 |  |  |
|  | 16-38 weeks |  | 1 | 0.028  (0.019 - 0.038) | 0.028  (0.006 - 0.129) |
|  | >38 weeks |  | 0 |  |  |
| Placebo | <6 weeks | -2.76 p<0.001 | 0 |  |  |
|  | 6-16 weeks | -4.19 p<0.001 | 0 |  |  |
|  | 16-38 weeks | -6.64 p=0.001 | 0 |  |  |
|  | >38 weeks | -5.81 p=0.017 | 0 |  |  |

^1^ added studies using trim and fill ^2^ pooled estimate from metaprop analysis ^3^ pooled estimate from trim-and-fill analysis

## Supplementary file 10.4: CRWL Egger tests coefficient and p-value and added studies by trim-and-fill procedure

|  | Duration | Egger test CRWL | Trim & fill^1^ | pooled estimate (95% CI)^2^ | pooled estimate t&f (95% CI)^3^ |
| --- | --- | --- | --- | --- | --- |
| Aripiprazole | <6 weeks |  | 0 |  |  |
|  | 6-16 weeks | -5.56 p=0.008 | 2 | 0.088  (0.065 - 0.115) | 0.089  (0.068 - 0.117) |
|  | 16-38 weeks | -4.20 p=0.007 | 0 |  |  |
|  | >38 weeks |  | 0 |  |  |
| Asenapine | <6 weeks | -1.21 p=0.230 | 1 | 0.015  (0.006 - 0.027) | 0.017  (0.010 - 0.030) |
|  | 6-16 weeks |  |  |  |  |
|  | 16-38 weeks | -7.38 p=0.130 | 0 |  |  |
|  | >38 weeks |  | 0 |  |  |
| Lurasidone | <6 weeks | -3.62 p=0.017 | 0 |  |  |
|  | 6-16 weeks |  |  |  |  |
|  | 16-38 weeks |  |  |  |  |
|  | >38 weeks |  | 0 |  |  |
| Olanzapine | <6 weeks | -1.13 p=0.075 | 0 |  |  |
|  | 6-16 weeks |  | 0 |  |  |
|  | 16-38 weeks | -5.57 p=0.175 | 0 |  |  |
|  | >38 weeks | -13.80 p=0.004 | 0 |  |  |
| Paliperidone | <6 weeks | -6.50 p=0.013 | 0 |  |  |
|  | 6-16 weeks |  | 0 |  |  |
|  | 16-38 weeks |  | 0 |  |  |
|  | >38 weeks |  | 0 |  |  |
| Quetiapine | <6 weeks |  |  |  |  |
|  | 6-16 weeks |  |  |  |  |
|  | 16-38 weeks |  | 0 |  |  |
|  | >38 weeks |  | 0 |  |  |
| Risperidone | <6 weeks |  |  |  |  |
|  | 6-16 weeks |  | 1 | 0.014  (0.000 - 0.059) | 0.025  (0.012 - 0.051) |
|  | 16-38 weeks |  |  |  |  |
|  | >38 weeks |  | 0 |  |  |
| Ziprasidone | <6 weeks |  |  |  |  |
|  | 6-16 weeks | -1.66 p=0.504 | 0 |  |  |
|  | 16-38 weeks |  |  |  |  |
|  | >38 weeks |  | 0 |  |  |
| Placebo | <6 weeks | -3.86 p<0.001 | 0 |  |  |
|  | 6-16 weeks | -5.23 p=0.011 | 0 |  |  |
|  | 16-38 weeks | 4.79 p=0.477 | 1 | 0.111  (0.085 - 0.140) | 0.131  (0.099 - 0.173) |
|  | >38 weeks |  | 0 |  |  |

^1^ added studies using trim and fill ^2^ pooled estimate from metaprop analysis ^3^ pooled estimate from trim-and-fill analysis

# Appendix 11: Risk of Bias Assessment

## Supplementary table 11.1: Risk of Bias assessment for the individual domains

## Supplementary table 11.2: Risk of Bias assessment for the individual studies

| **Study** | **Random sequence generation** | **Allocation concealment** | **Blinding of participants and personnel** | **Blinding of outcome assessment** | **Incomplete outcome data addressed** | **Selective reporting** |
| --- | --- | --- | --- | --- | --- | --- |
| Adams 2013 BMC Psychiatry | + | ? | + | + | + | + |
| Adams 2014 Schizophr Res Treatment | + | ? | + | + | + | + |
| Addington 2004 J Clin Psychiatry | + | ? | + | + | + | + |
| Alphs 2015 J Clin Psychiatry | + | + | + | + | + | + |
| Alvarez 2012 Eur J Psychiatry | + | ? | + | + | + | + |
| Arvanitis 1997 Biol Psychiatry | ? | ? | + | + | + | + |
| Ascher-Svanum 2005 BMC Psychiatry | + | ? | + | + | + |  |
| Bauer 2013 J Affect Disord | + | + | + | + | + | + |
| Berwaerts 2012a J Affect Disord | + | + | + | + | + | + |
| Berwaerts 2012b J Affect Disord | + | + | + | + | + | + |
| Berwaerts 2015 JAMA Psychiatry | + | + | + | + | + | + |
| Borison 1996 J Clin Psychopharmacol | ? | ? | + | + | + | + |
| Bortnick 2011 J Affect Disord | + | ? | + | + | + | + |
| Buchanan 2012 J Clin Psychopharmacol | ? | ? | + | + | + | + |
| Bushe 2010 J Pyschopharmacol | + | + | + | + | + |  |
| Calabrese 2005 Am J Psychiatry | + | + | + | + | + | + |
| Calabrese 2017 J Clin Psychiatry | + | + | + | + | + | + |
| Cantillon 2017 Schizophr Res | + | ? | + | + | + | + |
| Canuso 2010 J Clin Psychiatry | + | ? | + | + | + | + |
| Canuso 2010 J Clin Psychopharmacol | + | + | + | + | + | + |
| Casey 2003 Psychopharmacology | + | + | + | + | + | + |
| Casey 2008 Psychopharmacology | + | ? | + | + | + | - |
| Chan 2007 J Clin Psychiatry | + | + | + | + | + | + |
| Chan 2010 J Psychopharmacology | + | ? | + | + | + | + |
| Chen 2010 BMJ | + | + | + | + | + | + |
| Chen 2012 J Pyschopharmacol | + | + | + | + | + | + |
| Chen 2012 Pharmacopsychiatry | + | + | + | + | + | + |
| Cheng 2019 J Psychopharmacol | + | + | + | + | + | + |
| Chengappa 2010 Act Neuropsychiatr | ? | ? | + | + | + | + |
| Chrzanowski 2006 Psychopharmacology | ? | ? | + | + | + | + |
| Citrome 2012 Int Clin Psychopharmacol | + | ? | + | + | + | + |
| Conley 2001 Am J Psychiatry | ? | ? | + | + | + | + |
| Coppola 2011 Psychopharmacol Bull | + | + | + | + | ? | + |
| Correll 2015 Am J Psychiatry | + | ? | + | + | + | + |
| Correll 2020 Am J Psychiatry | + | ? | + | + | + | + |
| Correll 2020 JAMA Psychiatry | + | + | + | + | + | + |
| Cutler 2008 J Clin Psychopharmacol | + | + | + | + | - | + |
| Cutler 2009 J Clin Psychiatry | + | + | + | + | + | + |
| Cutler 2011 Clin Ther | + | + | + | + | + | + |
| Cutler 2011 Psychopharmacol Bull | ? | ? | + | + | + | + |
| Davidson 2007 Schizophr Res | ? | + | + | + | + | + |
| De Deyn 2005 J Clin Psychopharmacol | ? | ? | + | + | ? | + |
| De Hert 2011 Eur Arch Psychiatry Clin Neurosci | ? | ? | + | + | + | + |
| Deberdt 2005 Am J Geriatr Psychiatry | + | ? | + | + | + | + |
| Detke 2014 J Clin Psychopharmacol | ? | ? | + | + | + | + |
| Dossenbach 2007 J Clin Psychopharmacol | ? | ? | + | + | + | + |
| Dubovsky 2012 Int J Geriatr Psychiatry | ? | ? | + | + | + | + |
| Durgam 2014 Schizophr Res | + | ? | + | + | + | + |
| Durgam 2015 Bipolar Disord | + | ? | + | + | + | + |
| Durgam 2015 J Clin Psychiatry | + | ? | + | + | + | + |
| Durgam 2016 Am J Psychiatry | + | + | + | + | + | + |
| Durgam 2016 Schizophr Res | + | + | + | + | + | + |
| Durgam 2017 Neuropsychiatr Dis Treat | + | + | + | + | + | + |
| Earley 2019 Bipolar Disord | + | + | + | + | + | + |
| Fleischhacker 2009 Biol Psychiatry | + | + | + | + | + | + |
| Fleischhacker 2012 Int J Neuropsychopharmacol | + | + | + | + | + | + |
| Fleischhacker 2014 Br J Psychiatry | + | + | + | + | + | + |
| Fleischhacker 2017 Int J Neuropsychopharmacol | + | + | + | + | + | + |
| Fu 2015 J Clin Psychiatry | + | ? | + | + | + | + |
| Garcia 2009 CNS Drugs | + | + | + | + | + | ? |
| Gopal 2010 Int Clin Psychopharmacol | + | + | + | + | ? | ? |
| Green 2006 Schizophr Res | ? | ? | + | + | + | + |
| Grootens 2011 Schizophr Bull | + | ? | + | + | + | + |
| Grossberg 2020 Am J Geriatr Psychiatry | + | + | + | + | + | + |
| Higuchi 2019a Asia Pac Psychiatry | + | + | + | + | + | + |
| Higuchi 2019b Asia Pac Psychiatry | + | ? | + | + | ? | ? |
| Hill 2011 BMC Psychiatry | + | ? | + | + | + | + |
| Hobart 2018 Curr Med Res Opin | + | + | + | + | + | + |
| Hobart 2018 J Clin Psychiatry | + | + | + | + | + | + |
| Honer 2012 J Clin Psychiatry | + | + | + | + | + | + |
| Hough 2010 Schizophr Res | + | + | + | + | + | + |
| Ishigooka 2018 Psychiatry Clin Neurosci | + | + | + | + | + | + |
| Iyo 2021 Psychiatry Clin Neurosci | + | + | + | + | + | + |
| Jeste 2003 Am J Geriatr Psychiatry | ? | ? | + | + | + | + |
| Jindal 2013 Int J Psychiatry | + | ? | + | + | - | + |
| Kamijima 2013 J Affect Disord | + | ? | + | + | + | + |
| Kanba 2014 World J Biol Psychiatry | ? | ? | + | + | + | + |
| Kane 2002 J Clin Psychiatry | ? | ? | + | + | + | + |
| Kane 2006 Int Clin Psychopharmacol | ? | ? | + | + | + | + |
| Kane 2007 Schizophr Res | ? | ? | + | + | + | + |
| Kane 2009 Appl Health Econ Health Policy | + | ? | + | + | + | ? |
| Kane 2009 J Clin Psychiatry | ? | ? | + | + | + | + |
| Kane 2010 J Clin Pyschopharmacol | ? | ? | + | + | + | + |
| Kane 2011a J Clin Psychiatry | ? | ? | + | + | + | + |
| Kane 2011b J Clin Psychiatry | + | ? | + | + | + | ? |
| Kane 2012 J Clin Psychiatry | + | ? | + | + | + | + |
| Kane 2014 J Clin Psychiatry | + | + | + | + | + | + |
| Kane 2015 J Clin Psychopharmacol | + | ? | + | + | + | + |
| Kane 2015 Schizophr Res | + | + | + | + | + | + |
| Karagianis 2009 Schizophr Res | + | ? | + | + | + | + |
| Katagiri 2012 J Affect Disord | ? | ? | + | + | + | + |
| Katila 2013 Am J Geriatr Psychiatry | + | + | + | + | + | + |
| Kato 2020 Psychiatry Clin Neurosci | + | + | + | + | + | + |
| Keck 2003 Am J Psychiatry | ? | ? | + | + | + | + |
| Keck 2006 J Clin Psychiatry | ? | ? | + | + | ? | + |
| Keck 2007 J Clin Psychiatry | + | ? | + | + | + | + |
| Keks 2007 Br J Psychiatry | + | + | + | + | ? | + |
| Kerwin 2007 Eur Psychiatry | + | ? | + | + | + | + |
| Ketter 2017 J Affect Disord | + | + | + | + | + | + |
| Kim 2007 Prog Neuropsychopharmacol Biol Psychiatry | ? | ? | + | + | + | + |
| Kim 2012 Int Clin Psychopharmacol | ? | ? | + | + | + | + |
| Kinon 2006 J Clin Psychopharmacol | ? | ? | + | + | ? | + |
| Kinon 2008 J Clin Psychopharmacol | + | ? | + | + | + | + |
| Kinon 2011 J Clin Psychopharmacol | + | ? | + | + | + | + |
| Kinoshita 2016 Pyschopharmacology | + | + | + | + | + | + |
| Kishi 2016 Neuropsych Dis Treat | + | + | + | + | + | + |
| Krakowski 2009 Schizophr Res | ? | ? | + | + | + | + |
| Kramer 2007 J Clin Psychopharmacol | + | + | + | + | + | + |
| Kramer 2010 Int J Neuropsychopharmacol | + | + | + | + | + | + |
| Landbloom 2016 J Affect Disord | + | + | + | + | + | + |
| Landbloom 2017 CNS Spectr | + | + | + | + | + | + |
| Langosch 2008 J Clin Psychopharmacol | + | - | + | + | ? | + |
| Lauriello 2008 J Clin Psychiatry | + | ? | + | + | + | + |
| Li 2014 Schizophr Res | ? | + | + | + | ? | ? |
| Li 2015 J Psychiatr Res | + | + | + | + | + | + |
| Li 2016 Psychopharmacology | + | + | + | + | + | + |
| Lieberman 2003 Am J Psychiatry | ? | ? | + | + | + | + |
| Lieberman 2005 N Engl J Med | ? | ? | + | + | + | + |
| Loebel 2013 Schizophr Res | + | + | + | + | + | + |
| Loebel 2014 Am J Psychiatry | + | + | + | + | + | + |
| MacFadden 2009 Bipolar Disord | + | + | + | + | + | + |
| Marder 2007 Biol Psychiatry | + | + | + | + | + | + |
| Martin 2002 Curr Med Res Opin | ? | ? | + | + | + | + |
| McDonnell 2011 Hum Psychopharmacol | + | + | + | + | ? | + |
| McElroy 2010 J Clin Psychiatry | + | + | + | + | + | + |
| McEvoy 2006 Am J Psychiatry | ? | ? | + | + | + | + |
| McEvoy 2007 J Psychiatr Res | + | ? | + | + | + | + |
| McIntyre 2005 Eur Neuropsychopharmacol | + | ? | + | + | + | + |
| McIntyre 2009 Bipolar Disord | + | + | + | + | + | + |
| McIntyre 2010a J Affect Disord | + | + | + | + | + | + |
| McIntyre 2010b J Affect Disord | + | + | + | + | + | + |
| McQuade 2004 J Clin Psychiatry | ? | ? | + | + | ? | + |
| Meltzer 2010 Psychiatry Res | + | ? | + | + | ? | + |
| Meltzer 2011 Am J Psychiatry | + | + | + | + | + | + |
| Meltzer 2014 Schizophr Res | ? | ? | + | + | ? | + |
| Merideth 2012 Int Clin Psychopharmacol | + | + | + | + | + | + |
| Mintzer 2007 Am J Geriatr Psychiatry | ? | ? | + | + | + | + |
| Mitchel 2006 Clin Ther | ? | ? | + | + | ? | + |
| Moeller 2008 Int Clin Psychopharmacol | ? | ? | + | + | + | + |
| Mortimer 2004 Int Clin Psychopharmacol | + | + | + | + | + | + |
| Naber 2005 Acta Psychiatr Scand | ? | ? | + | + | + | + |
| Naber 2015 Schizophr Res | ? | ? | + | + | + | + |
| Nakamura 2009 J Clin Psychiatry | + | ? | + | + | + | + |
| Nasrallah 2013 Psychiatr Res | + | + | + | + | + | + |
| Nasrallah 2016 J Clin Psychiatry | + | ? | + | + | + | + |
| Nasser 2016 J Clin Psychopharmacol | + | + | + | + | + | + |
| Newcomer 2008 J Clin Psychiatry | ? | ? | + | + | + | + |
| Niufan 2008 J Affect Disord | ? | ? | + | + | ? | + |
| Olie 2006 Int Clin Psychopharmacol | + | ? | + | + | ? | + |
| Ou 2013 Psychopharmacology | + | ? | + | + | + | + |
| Pandina 2010 J Clin Psychopharmacol | + | + | + | + | + | + |
| Peluso 2013 Schizophr Res | + | + | + | + | ? | + |
| Perez-Iglesias 2014 Schizophr Res | + | ? | + | + | + | + |
| Peuskens 1997 Acta Pchychiatr Scand | ? | ? | + | + | ? | + |
| Pigott 2003 J Clin Psychiatry | ? | ? | + | + | + | + |
| Potkin 2003 Arch Gen Psychiatry | ? | ? | + | + | + | + |
| Potkin 2007 J Clin Psychiatry | ? | ? | + | + | + | + |
| Potkin 2015 TPP | + | + | + | + | + | + |
| Quiroz 2010 Biol Psychiatry | + | + | + | + | ? | + |
| Ryckmans 2009 Pharmacopsychiatry | + | ? | + | + | + | + |
| Sacchetti 2008 Schizophr Res | + | ? | + | + | + | ? |
| Sachs 2006 J Psychopharmacol | + | ? | + | + | + | + |
| Sachs 2015 J Affect Disord | + | ? | + | + | + | + |
| Savitz 2016 Int J Neuropsychopharmacol | + | + | + | + | + | + |
| Schmidt 2012 Eur Neuropsychopharmacol | + | + | + | + | + | + |
| Schneider 2006 N Eng J Med | + | ? | + | + | + | + |
| Schoemaker 2010 Pharmacopsychiatry | + | ? | + | + | + | + |
| Schreiner 2012 J Clin Psychopharmacol | + | + | + | + | + | + |
| Schreiner 2015 Schizophr Res | + | - | + | + | + | + |
| Schulz 2008 Br J Psychiatry | + | + | + | + | + | + |
| Sheehan 2009 J Affect Disord | + | ? | + | + | ? | + |
| Sheehan 2013 J Affect Disord | + | ? | + | + | + | + |
| Shen 2014 J Psychiatr Res | + | + | + | + | ? | + |
| Small 1997 Arch Gen Psychiatry | + | + | + | + | + | + |
| Srivastava 2012 J Psychiatr Res | ? | ? | + | + | + | + |
| Streim 2008 Am J Geriatr Psychiatry | ? | + | + | + | + | + |
| Suppes 2010 J Affect Disord | + | ? | + | + | + | + |
| Szegedi 2018 Am J Psychiatry | + | + | + | + | + | + |
| Thase 2006 J Clin Psychopharmacol | + | + | + | + | + | + |
| Thase 2008 J Clin Psychopharmacol | + | + | + | + | + | + |
| Tohen 2003 Am J Psychiatry | ? | ? | + | + | + | + |
| Tohen 2003a Arch Gen Psychiatry | + | + | + | + | + | + |
| Tohen 2003b Arch Gen Psychiatry | + | + | + | + | + | + |
| Tohen 2005 Am J Psychiatry | + | + | + | + | + | + |
| Tohen 2006 Am J Psychiatry | + | ? | + | + | + | + |
| Tohen 2008 J Clin Psychiatry | + | + | + | + | + | + |
| Tohen 2012 Br J Psychiatry | + | + | + | + | + | + |
| Tollefson 1997 Am J Psychiatry | ? | ? | + | + | ? | ? |
| Vanelle 2006 Eur Psychiatry | + | + | + | + | + | + |
| Vieta 2010 Bipolar Disord | + | + | + | + | + | + |
| Wang 2012 Psychopharmacol Bull | + | + | + | + | + | + |
| Weiden 2014 Schizophr Res | + | ? | + | + | + | + |
| Weiden 2016 CNS Drugs | + | + | + | + | + | + |
| Weisler 2009 CNS Spectr | + | + | + | + | + | + |
| Weisler 2011 J Clin Psychiatry | + | + | + | + | + | + |
| Xiang 2011 Pharmacopsychiatry | ? | ? | + | + | + | ? |
| Yang 2010 Clin Neuropharmacol | + | ? | + | + | + | + |
| Yatham 2020 Int Clin Psychopharmacol | + | + | + | + | + | + |
| Young 2009 Br J Psychiatry | + | ? | + | + | + | ? |
| Young 2010 J Clin Psychiatry | + | ? | + | + | + | ? |
| Zanarini 2011 J Clin Psychiatry | + | + | + | + | + | + |
| Zhong 2006 J Clin Psychiatry | ? | ? | + | + | + | + |

## Supplementary table 11.3: Certainty of evidence assessment (GRADE)

| **№ of studies** | **GRADE Certainty assessment** | | | | | | **Certainty** |
| --- | --- | --- | --- | --- | --- | --- | --- |
|  | **Study design** | **Risk of bias** | **Inconsistency** | **Indirectness** | **Imprecision** | **Other considerations** |  |
|  |  |  |  |  |  |  |  |
| Clinically relevant weight gain associated with antipsychotic use (follow-up: range < 6 weeks to > 38 weeks; assessed with: % of patients with CRWG; meta-regression) | | | | | | | |
| 200 | randomised trials | not serious | serious^a^ | not serious | not serious | publication bias strongly suspected  strong association^b,c^ | ⨁⨁⨁◯  Moderate |
| Clinically relevant weight loss associated with antipsychotic use (follow-up: range < 6 weeks to > 38 weeks; assessed with: % of patients with CRWL; meta-regression) | | | | | | | |
| 45 | randomised trials | not serious | serious^d^ | not serious | not serious | publication bias strongly suspected^c^ | ⨁⨁◯◯  Low |
| Clinically relevant weight gain in antipsychotic naive patients versus switch patients (follow-up: range < 6 weeks to > 38 weeks; assessed with: meta-regression) | | | | | | | |
| 200 | randomised trials | not serious | serious^a^ | not serious | not serious | publication bias strongly suspected  strong association^c,e^ | ⨁⨁⨁◯  Moderate |
| Clinically relevant weight gain and association with diagnosis (follow-up: range < 6 weeks to > 38 weeks; assessed with: meta-regression) | | | | | | | |
| 200 | randomised trials | not serious | serious^a^ | not serious | not serious | publication bias strongly suspected^c^ | ⨁⨁◯◯  Low |

#### **Explanations**

a. The I-square of the included studies ranged from 0.0% to 94.16%, indicating no heterogeneity to strong heterogeneity.

b. The proportion CRWG was significant in all AP and in placebo and varied between 1.7% (blonanserin <6 weeks) 1.9% (brexpiprazole 6-16 weeks) and 2.0% (placebo <6 week) on the one hand and 47% (clozapine 16-38 weeks), 49% (FGA > 38 weeks), 76% (clozapine > 38 weeks) on the other.

c. Funnel plots and Egger tests showed some evidence of publication bias, specifically for AP with fewer studies. Pooled estimate and p-value of trim-and-fill analyses were similar to the original analyses.

d. The I-square of the included studies ranged from 0.0% to 94.86% indicating little to strong heterogeneity.

e. AP-naive patients using aripiprazole, olanzapine and quetiapine showed significantly more CRWG than switch patients.

#### **Reference**

GRADEpro GDT: GRADEpro Guideline Development Tool [Software]. McMaster University and Evidence Prime, 2021. Available from [gradepro.org](https://gradepro.org/gradepro.org).
